# Reactions of diborenes with terminal alkynes: mechanisms of ligand-controlled *anti*-selective hydroalkynylation, cycloaddition and C≡C triple bond scission†

**DOI:** 10.1039/d1sc02081a

**Published:** 2021-06-21

**Authors:** Lukas Englert, Uwe Schmidt, Michael Dömling, Max Passargus, Tom E. Stennett, Alexander Hermann, Merle Arrowsmith, Marcel Härterich, Jonas Müssig, Alexandra Phillipps, Dominic Prieschl, Anna Rempel, Felix Rohm, Krzysztof Radacki, Fabian Schorr, Torsten Thiess, J. Oscar C. Jiménez-Halla, Holger Braunschweig

**Affiliations:** Institute for Inorganic Chemistry, Julius-Maximilians-Universität Würzburg Am Hubland 97074 Würzburg Germany h.braunschweig@uni-wuerzburg.de; Institute for Sustainable Chemistry & Catalysis with Boron, Julius-Maximilians-Universität Würzburg Am Hubland 97074 Würzburg Germany; Departamento de Química, Universidad de Guanajuato Noria Alta S/N 36050 Guanajuato Mexico jjimenez@ugto.mx

## Abstract

The reactions of terminal acetylenes with doubly Lewis base-stabilised diborenes resulted in different outcomes depending on the nature of the ligands at boron and the conformation of the diborene (cyclic *versus* acyclic). N-heterocyclic carbene (NHC)-stabilised diborenes tended to undergo *anti*-selective hydroalkynylation at room temperature, whereas [2 + 2] cycloaddition was observed at higher temperatures, invariably followed by a C–N bond activation at one NHC ligand, leading to the ring-expansion of the initially formed BCBC ring and formation of novel boron-containing heterocycles. For phosphine-stabilised diborenes only [2 + 2] cycloaddition was observed, followed by a rearrangement of the resulting 1,2-dihydro-1,2-diborete to the corresponding 1,3-isomer, which amounts to complete scission of both the B=B double and C≡C triple bonds of the reactants. The elusive 1,2-isomer was finally trapped by using a cyclic phosphine-stabilised diborene, which prevented rearrangement to the 1,3-isomer. Extensive density functional theory (DFT) calculations provide a rationale for the selectivity observed.

## Abstract 2

The outcome of reactions between diborenes and terminal alkynes can be tuned by varying the stabilising Lewis base and/or reaction conditions, to yield either the *anti*-hydroalkynylation product or [2 + 2] cycloaddition-derived boron heterocycles.

## Graphical abstract

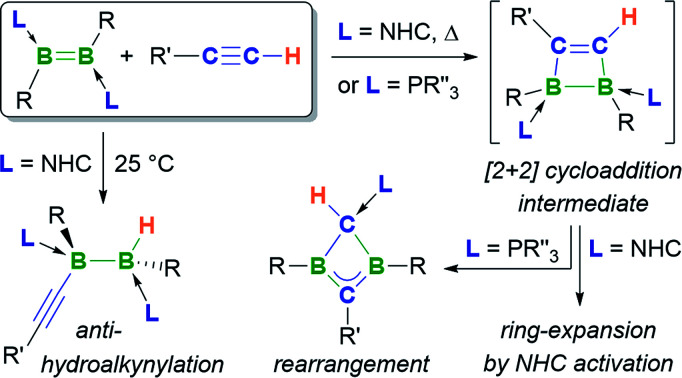

## Introduction

Transition-metal-catalysed reactions of terminal alkynes with olefins provide an atom-efficient way of forming new C–C bonds. Depending on the choice of catalyst and the nature of the terminal alkyne and alkene substrates, several types of reactivity can be targeted, including the hydroalkynylation of the C=C double bond,^1–8^ [2 + 2] cycloaddition reactions to yield cyclobutenes,^9–12^ or ene-yne metathesis to form conjugated dienes.^13–20^ While the reactions of terminal alkynes with alkenes require a catalyst to overcome high activation barriers, the analogous reactions with heavier group 14 alkene species proceed spontaneously owing to the much higher energy of their HOMO relative to that of alkenes. Therefore, disilenes, digermenes and distannenes undergo spontaneous [2 + 2] cycloadditions with terminal alkynes to form the corresponding 1,2-disila-,^21–26^ 1,2-digerma-^27–30^ and 1,2-distannacyclobut-3-enes.^31,32^ Only in the case of the acenaphthene-bridged distannene reported by Wesemann and co-workers was the cycloaddition with trimethylsilylacetylene found to be reversible.^31^ Experimental mechanistic studies on these cycloaddition reactions suggested a stepwise mechanism *via* a biradical or zwitterionic intermediate rather than a concerted process, which would be symmetry-forbidden.^23,25–27,33^

**Scheme 1:**
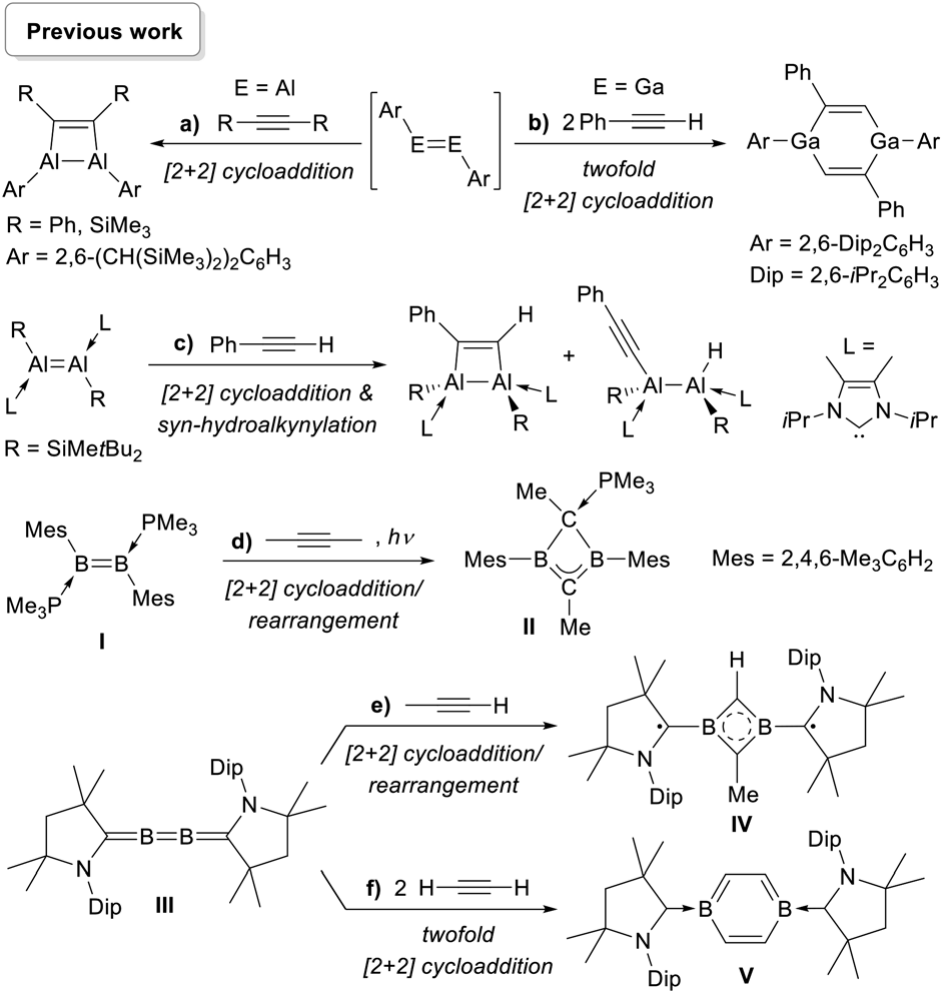
Spontaneous reactions of group 13 alkene analogues with alkynes.

Several spontaneous reactions with alkynes have been reported with group 13 alkene analogues. Similarly to the heavier group 14 congeners, the addition of alkynes to Tokitoh's dialumene-benzene adduct led to the formation of 1,2-dialuminacyclobut-3-enes (Scheme 1a),^34^ while Power's diaryldigallene underwent twofold cycloaddition with two equivalents of phenylacetylene to yield a 1,4-digallacyclohexa-2,5-diene (Scheme 1b).^35^ More recently, Inoue and co-workers reported that a doubly N-heterocyclic carbene (NHC)-stabilised 1,2-disilyldialumene undergoes both [2 + 2] cycloaddition and *syn*-selective hydroalkynylation with phenylacetylene (Scheme 1c).^36^ We have shown that under photolytic conditions the doubly phosphine-stabilised diborene **I** undergoes a cycloaddition/rearrangement reaction with 2-butyne to yield the phosphine-stabilised homoaromatic 1,3-dihydro-1,3-diborete **II**, presumably resulting from rearrangement of a 1,2-dihydro-1,2-diborete intermediate (Scheme 1d).^37^ In a similar manner the addition of propyne to our 2,3-diborabutatriene **III** also yielded an aromatic 1,3-diborete, **IV**, stabilised by two alkyl radical groups derived from cyclic (alkyl)(amino)carbene ligands (Scheme 1e), while the addition of acetylene led to the formation of the first neutral 1,4-diborabenzene, **V** (Scheme 1f).^37^

**Scheme 2:**
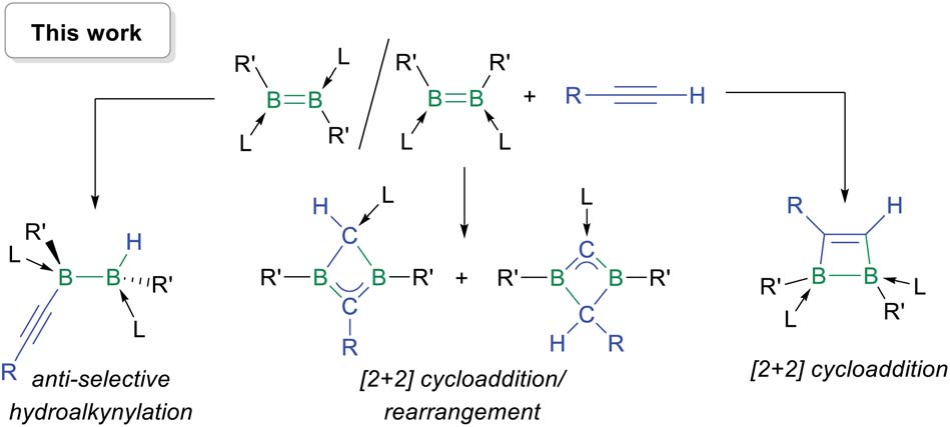
Reactivity of base-stabilised diborenes with terminal acetylenes. L = phosphine or NHC; R,R′ = anionic substituents.

In this work we show that, depending on the nature of the ligand environment at boron and the reaction conditions, the addition of terminal alkynes to diborenes may result in unprecedented hydroalkynylation of the B=B double bond or [2 + 2] cycloaddition (Scheme 2), with or without rearrangement of the resulting 1,2-dihydro-1,2-diborete. Computational mechanistic analyses provide a rationale for the experimentally observed selectivity.

## Results and discussion

### Reactivity of terminal alkynes with NHC-stabilised diborenes

**Scheme 3:**
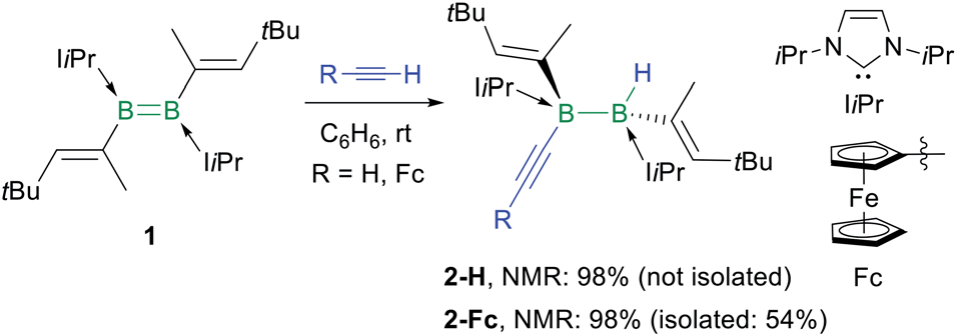
*Anti*-selective hydroalkynylation of NHC-stabilised diborene **1**. Relative stereochemistry shown only. NMR yields determined by ^11^B NMR spectroscopy.

**Fig. 1:**
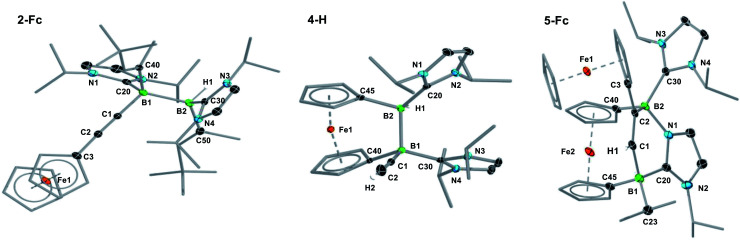
Crystallographically-derived molecular structures of (from left to right) **2-Fc**, **4-H** and **5-Fc**. Atomic displacement ellipsoids set at 50% probability. Ellipsoids of ligand periphery and hydrogen atoms omitted for clarity, except for alkene-, alkyne- and boron-bound hydrogen atoms. Selected bond lengths (Å) and angles (°) for **2-Fc**: B1–B2 1.826(3), B1–C1 1.594(3), B2–H1 1.144(19), B1–C20 1.663(3), B2–C30 1.621(3), C1–C2 1.206(2), torsion angles (C1–B1–B2–H1) 166(1), (C20–B1–B2–C30) 158.63(15), (C40–B1–B2–C50) 166.52(15); for **4-H**: B1–B2 1.835(2), B1–C1 1.592(2), B2–H1 1.147(17), B1–C20 1.612(2), B2–C30 1.629(2), C1–C2 1.201(2), torsion (C1–B1–B2–H1) 179.9(9), (C20–B1–B2–C30) −55.00(17), (C40–B1–B2–C50) 45.41(14), Cp tilt angle (bridging ferrocenediyl) 16.3; for **5-Fc**: B1–C1 1.620(4), C1–C2 1.350(3), C2–B2 1.618(3), B2–N1 1.583(3), N1–C20 1.356(3), C20–B1 1.642(4), C20–N2 1.361(3), B2–C30 1.655(3), Cp tilt angle (bridging ferrocenediyl) 2.5.

The reaction of the doubly NHC-stabilised divinyldiborene **1** (ref. 38) with acetylene in benzene at room temperature yielded a colourless solution with a single, very broad, ^11^B NMR resonance around −19 ppm (Scheme 3). While multiple attempts to isolate this compound failed because of its oily consistency, the reaction of **1** with ferrocenylacetylene (FcC≡CH) resulted in the formation of a compound, **2-Fc**, with a similar ^11^B NMR resonance at −18.6 ppm, in *ca.* 98% selectivity as determined by ^11^B NMR-spectroscopic analysis of the reaction mixture (Scheme 3).§§The relatively low isolated yields of many of the reaction products presented herein, despite the good to excellent selectivities observed by ^11^B NMR spectroscopy (see ^11^B NMR spectra of reaction mixtures prior to work-up, Fig. S39–S52 in the ESI†), is due to a combination of (a) the small reaction scales (typically 20–50 mg of diborene), (b) the very high solubility of the products in pentane at −30 °C (^11^B NMR spectra of the mother liquor after recrystallization always showed large amounts of product remaining in solution), and (c) the difficulty of separating the products from remaining excess alkyne.**2-Fc** was identified by X-ray crystallographic analysis (Fig. 1) as the hydroalkynylation product of the B=B double bond, resulting in a doubly NHC-stabilised 1-alkynyl-2-hydrodiborane.

The fact that only one resonance is observed in the ^11^B NMR spectrum despite the unsymmetrical substitution pattern of the diborane suggests that the resonances of the two boron nuclei overlap, *i.e.* that the hydride and the acetylide ligand have a very similar electronic influence on the boron nuclei. The ^1^H NMR spectrum shows the formation of a single diastereomer. The presence of the boron-bound hydride was confirmed by a B*H* singlet in the ^1^H{^11^B} NMR spectrum at 2.45 ppm. The hydroacetylenation of B–B multiple bonds offers a new atom-efficient route to alkynylboranes, which are traditionally synthesised by salt metathesis of halo- or alkoxyboranes with lithium or Grignard acetylides,^39–41^ or by the catalytic dehydrocoupling of terminal alkynes with hydroboranes.^42,43^ The solid-state structure of **2-Fc** shows that the addition of the C–H bond occurred with *anti*-selectivity, similarly to the hydrogenation,^44^ transfer hydrogenation^45^ and hydroboration of diborenes.^46,47^ This is rather unexpected since the 1,2-addition of the C–H bond to the B=B double bond should occur in a *syn* fashion, similar to the hydroalkynylation of Inoue's dialumene with phenylacetylene (Scheme 1c).^36^ The mechanistic analyses presented later in this paper (*vide infra*) shed some light on this unexpected *anti*-selectivity.

**Scheme 4:**
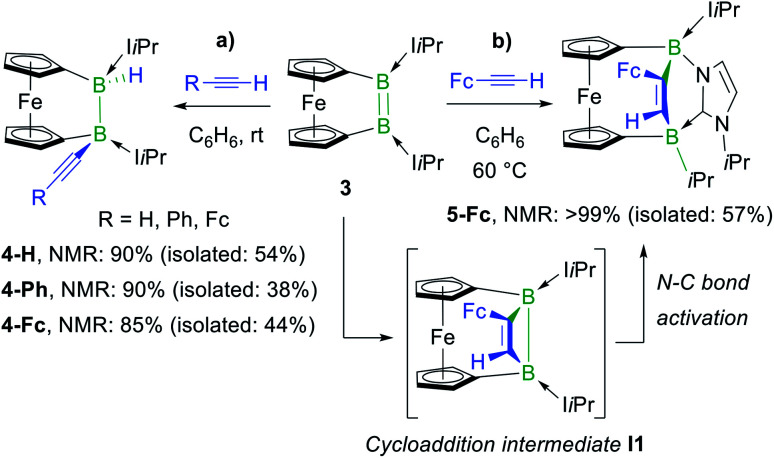
Divergent reactivity of dibora[2]ferrocenophane **3** with terminal acetylenes and postulated cycloaddition intermediate **I1** in square brackets. Relative stereochemistry shown only.

In a similar manner, the room-temperature reaction of acetylene, phenylacetylene and ferrocenylacetylene with the dibora[2]ferrocenophane **3** (ref. 48) resulted in hydroalkynylation of the B=B double bond, yielding the ferrocenediyl-bridged and doubly NHC-stabilised 1-alkynyl-2-hydrodiboranes **4-R** (R = H, Ph), with ≥85% selectivity according to ^11^B NMR spectra of the reaction mixtures (Scheme 4a).§ Like **2-Fc**, compounds **4-R** show only one ^11^B NMR resonance at *ca.* −18 ppm despite their unsymmetrical nature. The B*H* resonance was detected in the ^1^H{^11^B} NMR spectra as a 1H singlet at *ca.* 3.2 ppm, while the terminal acetylenic proton of **4-H** appeared as a 1H singlet at 2.70 ppm. Yellow single crystals of **4-H** suitable for X-ray diffraction analysis confirmed the 1,2-*anti*-addition of the C–H bond, as already observed in **2-Fc** (Fig. 1). The B–B bond length of **4-H** (1.835(2) Å) is comparable to that of **2-Fc** (1.826(3) Å) despite the additional strain imposed by the bridging ferrocenediyl ligand.

In contrast, the reaction of **3** with ferrocenylacetylene at 60 °C afforded the 1,2-diboraalkene **5-Fc** as the sole product (Scheme 4b),§ which formally results from the [2 + 2] cycloaddition of the acetylene to the diborene, yielding intermediate **I1**, followed by the insertion of the B–B bond into an exocyclic C–N bond at one NHC ligand. A similar exocyclic C–N bond activation at an NHC ligand has been observed in the reaction of a doubly NHC-stabilised diiododisilene with a thiolate.^49^ The ^11^B NMR spectrum of **5-Fc** shows two broad resonances at −7.7 and −14.7 ppm, the former corresponding to the *i*Pr-bearing B1 and the latter to the N-bound B2. The proton of the bridging alkene moiety appears in the ^1^H NMR spectrum as a deshielded 1H singlet at 8.02 ppm. The solid-state structure of **5-Fc** (Fig. 1) shows the formation of a six-membered 2,5-dihydro-1,2,5-azadiborinine ring, which adopts a boat conformation with the boron atoms at the apices, fused at C20–N1 with the imidazol-2-ylidene ring of the former NHC ligand. The C1–C2 bond length of 1.350(4) Å denotes a typical C=C double bond, while the N2–C20, C20–N1 and N1–B2 bond lengths of 1.361(3), 1.356(4) and 1.583(4) Å, respectively, are all within the range of partial double bonds, indicating some degree of π delocalisation over the N2–C20–N1–B2 moiety. The fact that the cycloaddition intermediate **I1** cannot be isolated and undergoes rapid ring-expansion by activation of the N–C_*i*Pr_ bond is likely due to the double ring strain in **I1**, imposed by both the four-membered 1,2-dihydro-1,2-diborete ring and the ferrocenediyl bridge. The temperature-dependent selectivity of the reaction of **3** with ferrocenylacetylene indicates that **4-Fc** is the kinetic product while **5-Fc** is the thermodynamic product. Attempts to obtain the analogous compounds **5-H** and **5-Ph** by heating the reaction mixtures of **3** with acetylene and phenylacetylene at 60 °C exclusively yielded **4-H** and **4-Ph**, respectively. These appear, therefore, to be both the thermodynamic and kinetic products. However, work-up of one room-temperature reaction of **3** with phenylacetylene yielded, besides **4-Ph**, a few crystals of **5-Ph**, the solid-state structure of which is analogous to that of **5-Fc** (see Fig. S53†), thus indicating that the activation energy of the cycloaddition reaction must actually be relatively close to that of the hydroalkynylation reaction in this case.

**Scheme 5:**
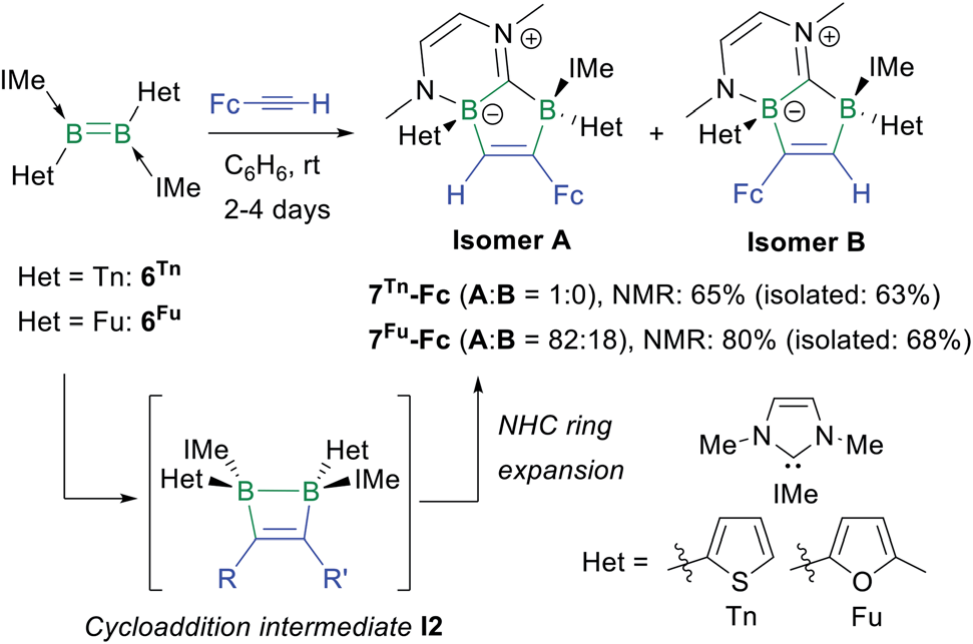
Reaction of ferrocenylacetylene with **6Tn** and **6Fu**, and postulated intermediate **I2** in square brackets. Relative stereochemistry shown only.

The reactions of the dithienyl- and difuryldiborenes **6Tn** and **6Fu**,^47^ respectively, with phenylacetylene and *tert*-butylacetylene proved extremely unselective regardless of the reaction conditions applied, yielding mixtures of up to six different products with ^11^B NMR shifts spanning the −25 to +3 ppm range. In contrast, the analogous reactions with ferrocenylacetylene at room temperature in benzene proved relatively selective, yielding the orange-coloured [1,3]diborolo[3,2-*b*][1,4,2]diazaborinine derivatives **7Tn-Fc** and **7Fu-Fc** as the major products with *ca.* 65% and 80% selectivity, respectively, as determined by ^11^B NMR spectroscopy (Scheme 5), alongside several minor products including the hydroalkynylation products (*δ*_11B_ ≈ −20 ppm).§**7Tn-Fc** and **7Fu-Fc** can be viewed as resulting from the cycloaddition of FcC≡CH to the diborene, yielding the intermediate 1,2-dihydro-1,2-diborete **I2** (Scheme 5), followed by ring expansion of one NHC ligand through the addition of the B–B bond of **I2** to the carbene carbon atom and insertion of one boron atom into the C_carbene_–N bond. This type of NHC ring expansion has previously been observed for a number of NHC-stabilised diboranes,^50–52^ and is probably favoured in this case by the release of ring strain upon expansion of the B_2_C_2_ to the B_2_C_3_ ring. The ^11^B NMR resonances of **7Tn-Fc** appear at −4.6 and −11.5 ppm, those of **7Fu-Fc** at −6.0 and −12.7 ppm. The ^1^H NMR BC*H* proton of the 1,2-diboraalkene moiety of **7Tn-Fc** appears as a broad resonance at 7.23 ppm. The ^1^H NMR spectrum of crude **7Fu-Fc** shows two distinct BC*H* resonances at 7.89 and 7.78 ppm in a 82 : 18 ratio. Since the cycloaddition step must take place in a *syn* fashion, only the (*S*,*R*)/(*R*,*S*) diastereomer of **7Fu-Fc** should be formed. The presence of two distinct BC*H* resonances therefore suggests the formation of the two regioisomers **A** and **B** of **7Fu-Fc** (Scheme 5).

An X-ray crystallographic analysis of the regioisomer **A** of **7Tn-Fc** (Fig. 3) confirms the ring expansion of the original 1,2-dihydro-1,2-diborete and of one NHC ligand, leading to the fused C_5_B_2_N_2_ heterocycle. As expected from the suggested reaction pathway *via***I2**, the structure is that of the (*S*,*R*)/(*R*,*S*) diastereomer. The C1–C2 bond length of 1.341(3) Å is that of a typical C=C double bond. Furthermore, the short C20–N2 bond length of 1.305(3) Å is indicative of a C=N double bond, making this compound a zwitterion.

### Reactivity of terminal alkynes with phosphine-stabilised diborenes

**Scheme 6:**
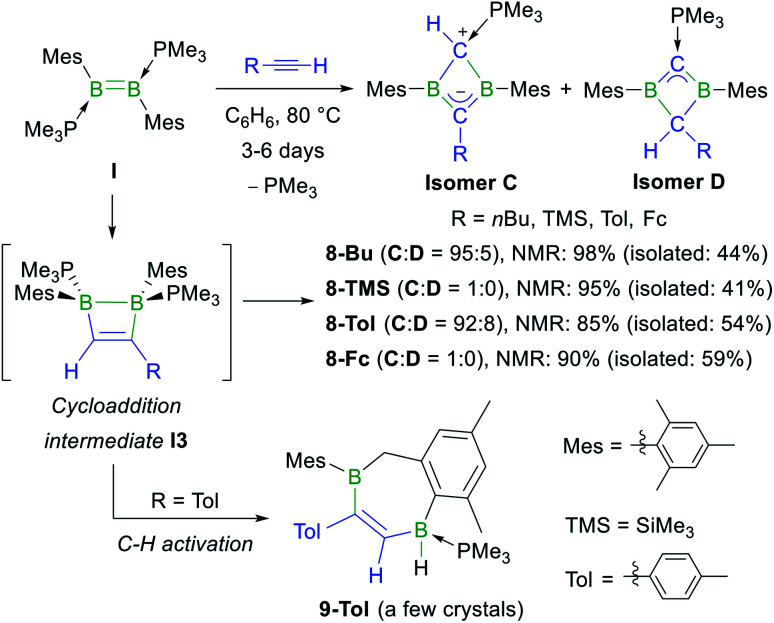
Reaction of phosphine-stabilised diborene **I** with terminal acetylenes, postulated intermediate **I3** in square brackets, and C–H activation product **9-Tol** deriving from **I3**. Relative stereochemistry shown only.

**Fig. 2:**
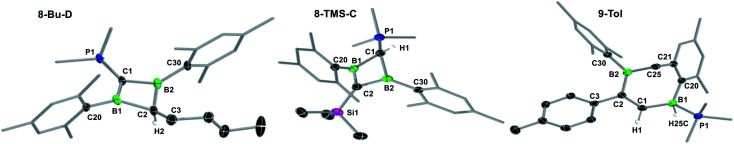
Crystallographically-derived molecular structures of (from left to right) **8-Bu-D**, **8-TMS-C** and **9-Tol**. Atomic displacement ellipsoids set at 50% probability. Ellipsoids of ligand periphery and hydrogen atoms omitted for clarity, except for C1/C2- and boron-bound hydrogen atoms. Selected bond lengths (Å) and angles (°) for **8-Bu-D**: B1–C1 1.501(3), C1–B2 1.509(3), B2–C2 1.615(3), C2–B1 1.613(3), B1⋯B2 1.992(3), C1–P1 1.698(2), B1–C1–B2 82.88(16), C1–B2–C2 96.40(17), B1–C2–B2 76.20(15), C2–B1–C1 96.78(16), Σ(∠B1) 359.78(18), Σ(∠B2) 359.78(18), Σ(∠C1) 355.10(16), torsion angles (C1–B1–C20–C21) 95.8(3), (C1–B2–C30–C31) −91.6(3); for **8-TMS-C**: B1–C1 1.6580(19), C1–B2 1.619(2), B2–C2 1.492(2), C2–B1 1.485(2), B1⋯B2 1.869(2), C1–P1 1.7455(14), B1–C1–B2 69.52(9), C1–B2–C2 99.21(11), B1–C2–B2 77.76(11), C2–B1–C1 100.67(11), Σ(∠B1) 359.36(15), Σ(∠B2) 358.22(12), Σ(∠C2) 351.35(11), torsion angles (C1–B1–C20–C21) −133.95(14), (C1–B2–C30–C31) −108.61(16); for **9-Tol**: B1–C1 1.603(2), C1–C2 1.356(2), C2–B2 1.558(2), B1–H1 1.159(17), B1–P1 1.9510(16).

Having shown that NHC-stabilised diborenes undergo both hydroalkynylation and [2 + 2] cycloaddition with terminal acetylenes, depending on the substitution pattern of the diborene and/or kinetic *versus* thermodynamic reaction control, our attention turned to phosphine-stabilised diborenes. Previous work by our group had shown that diborene **I** reacts with 2-butyne under photolytic conditions to generate the homoaromatic 1,3-dihydro-1,3-diborete **II** (Scheme 1d).^37^ In a similar manner, diborene **I** reacted with 1-hexyne, trimethylsilyl- (=TMS), *p*-tolyl- (=Tol) and ferrocenylacetylene at 80 °C over a period of several days to yield the 1,3-dihydro-1,3-diboretes **8-Bu**, **8-TMS**, **8-Tol** and **8-Fc**, respectively, with 85–98% selectivity as determined by ^11^B NMR spectroscopy (Scheme 6).§ This complete scission of B=B multiple and C≡C triple bonds is also observed in the reaction of a NHC-stabilised diboryne with alkynes, which leads to the formation of highly reactive antiaromatic 1,3-diboretes.^53^ As described previously, the formation of **8-R** is likely to proceed *via* a 1,2-dihydro-1,2-diborete **I3**, which spontaneously rearranges to the thermodynamically more stable 1,3 isomer,^54^ with loss of one PMe_3_. The existence of **I3** is supported by the fortuitous isolation of a few crystals of **9-Tol** (Scheme 6 and Fig. 2), which results from the intramolecular C–H activation of a mesityl-*ortho*-methyl group at the B–B bond of **I3**. In all cases the major or sole product obtained was the analogue of **II**, tautomer **C**, in which the remaining PMe_3_ ligand is bound to the positively charged, former terminal carbon of the acetylene. The ^11^B NMR shifts of **8-R** are dependent upon the nature of the substituent R, increasing from 25.6 to 38.8 ppm, in the order of R = Me < *n*Bu < *p*-tolyl < Fc < SiMe_3_, *i.e.* with the steric demands of R. Concomitantly, the ^31^P NMR shifts decrease from 23.2 to 15.6 ppm following the same order. For **8-Bu** and **8-Tol** a second minor product was observed in solution (<10%), in which the ^11^B NMR resonance is shifted significantly downfield to 54.7 and 51.4 ppm, respectively, compared to **C**, while the ^31^P NMR resonance is shifted more than 20 ppm upfield to −3.6 and −2.7 ppm, respectively. These compounds were identified by NMR spectroscopy and X-ray crystallographly as tautomer **D**, in which the proton at the phosphine-bound carbon atom has migrated to the R-bound carbon atom of the BCBC ring.

**Fig. 3:**
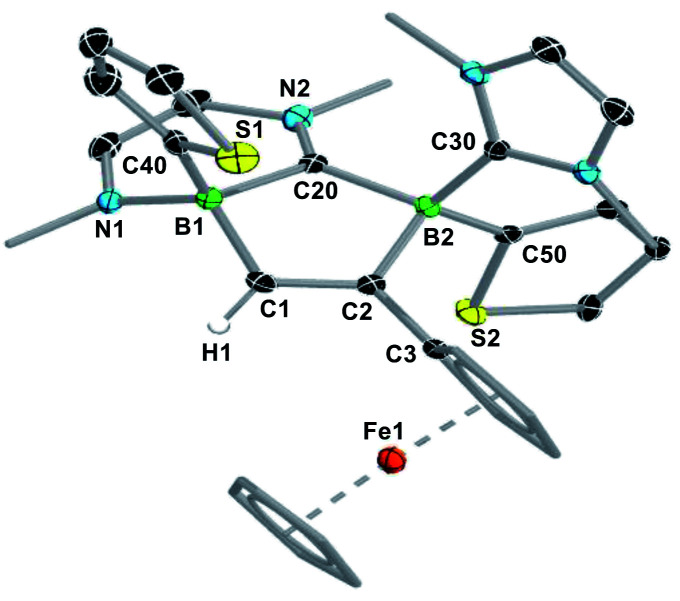
Crystallographically-derived molecular structure of **7Tn-Fc**. Atomic displacement ellipsoids set at 50% probability. Ellipsoids of ligand periphery and hydrogen atoms omitted for clarity, except for the diboraalkene-bound proton. Selected bond lengths (Å): B1–C1 1.603(3), C1–C2 1.341(3), C2–B2 1.664(3), B2–C20 1.644(3), C20–B1 1.631(3), B1–N1 1.559(3), C20–N2 1.305(3).

X-ray crystallographic analysis of **8-R-C** (R = TMS, Tol, Fc) and **8-Bu-D** (Fig. 2 and S54 and S55 in the ESI†) show a similar butterfly conformation to other structurally related 1,3-diboretes,^37,55–58^ with a puckering angle between the two CB_2_ planes of around 30°. The sterics of the substituent R influence the distortion of the BCBC heterocycle and the degree of rotation of the mesityl ligands, both aimed at minimising the steric repulsion between the mesityl and R groups. Thus the B⋯B distance decreases from 1.992(3) Å in the least sterically encumbered derivative **8-Bu-D** to 1.869(2) Å in the most sterically encumbered derivative **8-TMS-C**. Concomitantly, the absolute values of the torsion angles (C1–B1–C20–C21) and (C1–B2–C30–C31) increase from 95.8(3) and 91.6(3)°, respectively, in **8-Bu-D** to 133.95(14) and 108.61(16)°, respectively, in **8-TMS-C**. The B–C bonds to the sp^3^-hybridised carbon atom (1.613(3)–1.658(2) Å) are within the upper range of B–C single bonds, whereas the B–C bonds to the sp^3^-hybridised carbon atom (1.483(2)–1.509(3) Å) are within the range of partial double bonds, typical of a 2π-homoaromatic structure. For **8-Fc-C**, the π electron density seems to be delocalised over the entire CpCB_2_ moiety as shown by the near-coplanar arrangement of the Cp ring and CB_2_ unit, and the short C–C_Cp_ partial double bond (1.455(3) Å). It is noteworthy that the P1–C1 bond **8-Bu-D** (1.698(2) Å) is actually within the range of P=C double bonds (Ph_3_P=C(CH_3_)_2_: 1.696(6) Å).^59^

**Scheme 7:**
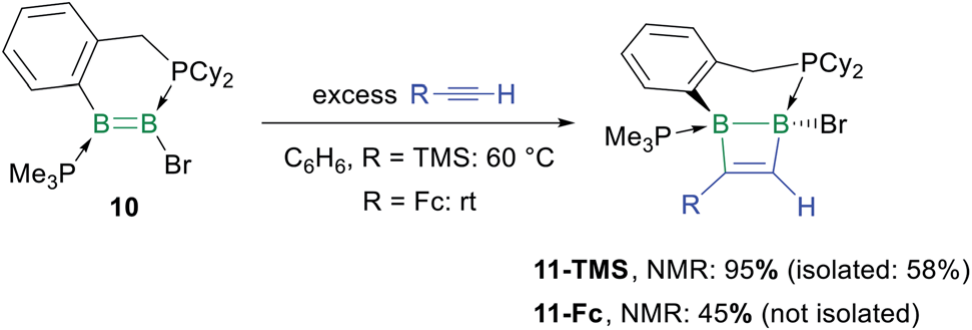
Cycloaddition of terminal acetylenes to the phosphine-stabilised cyclic diborene **10**. Relative stereochemistry shown only.

**Fig. 4:**
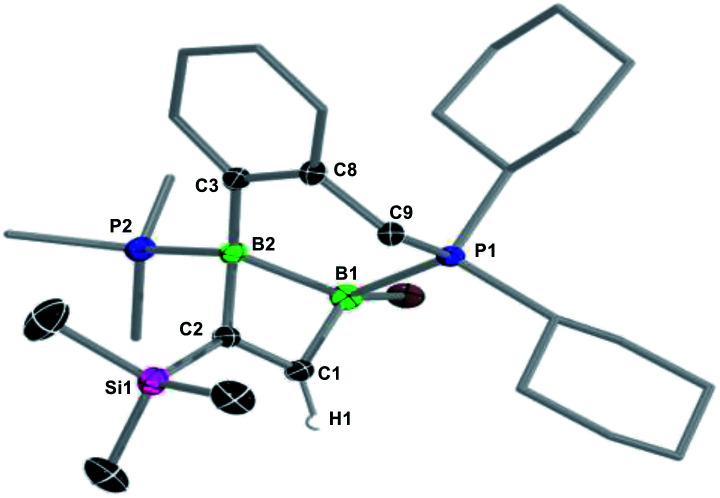
Crystallographically-derived molecular structure of **11-TMS**. Atomic displacement ellipsoids set at 50% probability. Ellipsoids of ligand periphery and hydrogen atoms omitted for clarity. Selected bond lengths (Å) and angles (°): B1–B2 1.832(5), B2–C2 1.629(5), C2–C1 1.355(5), C1–B1 1.566(5), B1–P1 1.930(4), B2–P2 1.922(4), C1–B1–B2 83.4(2), B1–B2–C2 78.5(2), B2–C2–C1 98.5(3), C2–C1–B1 97.2(3), torsion angles (C1,B1,B2,C2) 9.9(2), (B1,C1,C2,B2) 13.3(3).

In the hope of isolating the elusive 1,2-dihydro-1,2-diborete cycloaddition product we finally examined the reactivity of the cyclic diborene **10** (ref. 60) with terminal acetylenes. Indeed, the bridging benzylphosphine ligand of **10** should prevent rearrangement of the 1,2-diborete to the 1,3-isomer and the absence of NHC ligands or acidic protons should prevent further ligand activation reactions. As expected, diborene **10** underwent a [2 + 2] cycloaddition with trimethylsilylacetylene, resulting in the formation of the polycyclic 1,2-dihydro-1,2-diborete **11-TMS** with *ca.* 98% selectivity (Scheme 7).§ The ^11^B NMR spectrum of **11-TMS** shows two broad resonances very close to each other at −16.0 and −16.5 ppm, while the ^31^P NMR spectrum displays two broad doublets at 8.7 and −12.9 ppm for the PhCH_2_*P*Cy_2_ and *P*Me_3_ phosphorus nuclei, respectively (^3^*J*_P–P_ = 107 Hz). The proton of the bridging alkene moiety appears as a ^1^H NMR doublet at 8.16 ppm (^3^*J*_H–P_ = 6.0 Hz). The analogous reaction with ferrocenylacetylene at room temperature proved far less selective, ^11^B and ^31^P NMR spectra evidencing the formation of **11-Fc** (*δ*_11B_ = −17.5, −18.0 ppm) with *ca.* 45% selectivity, alongside several unidentified species. **11-TMS** is only the third structurally characterised 1,2-dihydro-1,2-diborete. Like Kaufman's 1,2-diamino-1,2-dihydro-1,2-benzodiborete (B–B 1.727(2) Å), obtained from the reductive cyclisation of the *o*-(BClN*i*Pr_2_)_2_C_6_H_4_ precursor,^61^ the B_2_C_2_ ring of **11-TMS** is slightly twisted (torsion angles (C1,B1,B2,C2) 9.9(2); (B1,C1,C2,B2) 13.3(3)°) due to the strain of the fused ring system. In contrast, Siebert's 1,2-diamino-1,2-dihydro-1,2-diborete (B–B 1.75 Å) is fully planar.^62^ Furthermore, the B–B bond in **11-TMS** is significantly elongated (1.832(5) Å) due to the additional coordination of the two phosphine ligands (Fig. 4).

### Computational mechanistic analyses

In order to gain more insight into the selectivity of these reactions we performed a computational mechanistic study, derived from theoretical calculations at the (SMD:benzene)ω-B97XD/def2-tzvpp//ω-B97XD/def2-svp level (see ESI† for further computational details).¶¶Four other basis sets of similar quality and size were tested, and the same trend was followed. There is a minor discrepancy with Pople's 6-311+G(d,p) basis set. The model diborenes chosen for this study were the doubly IMe-stabilised 1,2-bis(1-methylvinyl)diborene **R1**, representative of NHC-stabilised diborenes, and the doubly PMe_3_-stabilised 1,2-diphenyldiborene **R2**, representative of phosphine-stabilised diborenes, while propyne was chosen as the model alkyne.

**Fig. 5:**
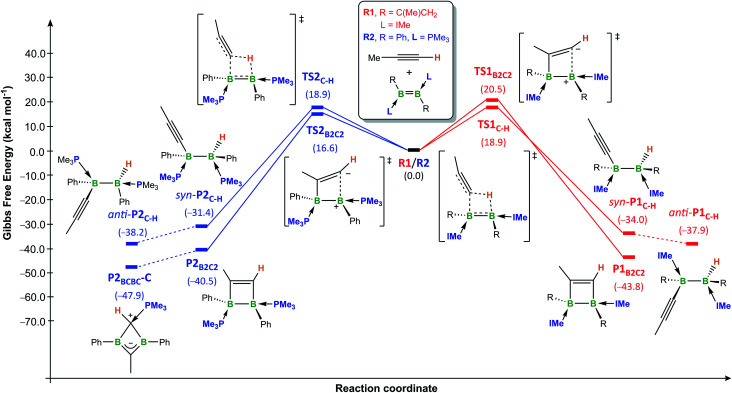
Energy profiles for the [2 + 2] cycloaddition and 1,2-addition of the C–H bond of propyne to the model doubly IMe-stabilised 1,2-bis(1-methylvinyl)diborene **R1** (red pathways) and the doubly PMe_3_-stabilised 1,2-diphenyldiborene **R2** (blue pathways) in benzene at room temperature, calculated at the (SMD:benzene)ω-B97XD/def2-tzvpp//ω-B97XD/def2-svp level of theory. Energies in parentheses in kcal mol^−1^.

The overall calculated reaction profiles for the NHC-stabilised diborene **R1** (Fig. 5, red pathways) show that the hydroalkynylation product *anti*-**P1C–H** is the kinetic product (Δ*G*^‡^ = 18.9 kcal mol^−1^), the reaction being highly exothermic (Δ*G*_1_ = −34.0 kcal mol^−1^). The [2 + 2] cycloaddition proceeds *via* an unsymmetrical zwitterionic late transition state, **TS1B2C2**, in which the B–C_Me_ bond is already pre-formed, similarly to what has been postulated for some cycloadditions of alkynes to disilenes.^25,33^ The [2 + 2] cycloaddition product **P1B2C2**, for which the energy barrier is only 1.6 kcal mol^−1^ higher than for the hydroalkynylation process, is the thermodynamic product, being 5.9 kcal mol^−1^ more stable than *anti*-**P1C–H**. This is in good agreement with the experimental observations that hydroalkynylation is favoured at room temperature for diborenes **1** and **3**, whereas cycloaddition is favoured at elevated temperatures for **3**. The fact that hydroalkynylation is not observed with diborenes **6Tn/Fu** suggests that the barriers of the two processes may be inverted in this case.

**Scheme 8:**
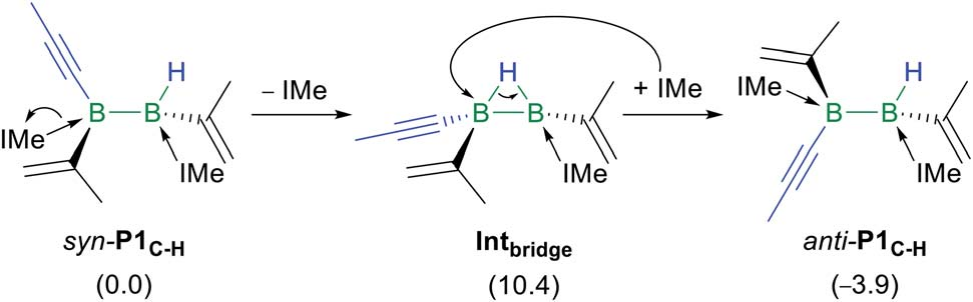
Mechanism of the *syn*-to-*anti* isomerisation of **P1C–H**. Energies in parentheses in kcal mol^−1^.

Given that the hydroalkynylation of **R1** shown in Fig. 5 produces the *syn*-addition product (*syn*-**P1C–H**) rather than the experimentally observed *anti*-addition product (*anti*-**P1C–H**), the *syn*-to-*anti* conversion mechanism was also investigated (see Fig. S59 in the ESI†). The only energetically viable route at 25 °C proceeds through a NHC decoordination–recoordination mechanism with a mild overall barrier (Δ*G*^‡^_R_ = 12.0 kcal mol^−1^), *via* a hydride-bridged diborane intermediate, **Intbridge** (Scheme 8). The resulting product, *anti*-**P1C–H**, is more stable than *syn*-**P1C–H** by 3.9 kcal mol^−1^.||||Although *anti*-**P1C–H** may be obtained directly from the hydroalkynylation of the *cis*-conformer of **R1**, this route was discarded due to the high rotation barrier of the B=B double bond. The absence of *syn*-to-*anti* isomerisation in Inoue's dialumene hydroalkynylation product (Scheme 1c)^36^ despite the much weaker Al–C_NHC_ bonds, which should in theory favour the dissociation of one NHC ligand required for the isomerisation (bond dissociation energies at 298 K: *D*^°^_B–C_ = 107 kcal mol^−1^, *D*^°^_Al–C_ = 64 kcal mol^−1^) ,^61,63^ may be due to the fact that the resulting singly NHC-stabilised 1-alkynyl-2-hydrodialane is less likely to stabilise itself through a three-centre-two-electron hydride bridge, as is common for hydrodiboranes.^64^

The overall calculated reaction profiles for the phosphine-stabilised diborene **R2** (Fig. 5, blue pathways) show that the cycloaddition product, **P2B2C2**, which is precursor to the final 1,3-dihydro-1,3-diborete **P2BCBC**, is both the kinetic (Δ*G*^‡^ = 16.6 kcal mol^−1^) and the thermodynamic product (Δ*G*_2_ = −40.5 kcal mol^−1^), being 9.1 kcal mol^−1^ more stable than the hydroalkynylation product *anti*-**P2C–H**. This is again in agreement with the experimental observations that [2 + 2] cycloaddition is favoured in all cases for diborenes **I** and **10**. The [2 + 2] cycloaddition again proceeds *via* an unsymmetrical zwitterionic late transition state, **TS2B2C2**, in which the B–C_Me_ bond is already pre-formed.**The origin of the ligand-controlled selectivity for cycloaddition versus hydroalkynylation seems to be steric rather than electronic control. See Fig. S61 in ESI† and associated text.

**Fig. 6:**
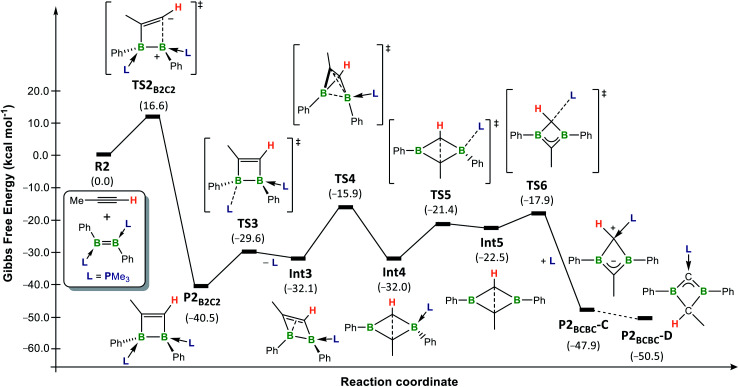
Energy profile for the conversion of model diborene **R2** and propyne into the tautomers **C** and **D** of **P2BCBC** in benzene at room temperature, calculated at the (SMD:benzene)ω-B97XD/def2-tzvpp//ω-B97XD/def2-svp level of theory. Energies in parentheses in kcal mol^−1^.

The rearrangement of **P2B2C2** to **P2BCBC** was also investigated computationally (Fig. 6). Initially, PMe_3_ dissociates from the boron atom at the β position to the methyl substituent of the 1,2-dihydro-1,2-diborete (Δ*G*^‡^ = 10.9 kcal mol^−1^) (**TS3**) and Δ*G*_3_ = 8.4 kcal mol^−1^ (**Int3**)).††††Dissociation of the other phosphine is unlikely since the analogous intermediate is 4 kcal mol^−1^ higher in energy than **Int3**. Moreover, a comparison of the B–P distances in **P2B2C2** suggests that the first phosphine (B–P 1.94 Å) is more labile than the second one (1.92 Å). To compensate for the loss of electron density, the B_2_C_2_ ring distorts from a planar ring to a nonplanar rhombic cluster, in which the sp^2^-hybridised boron atom B1 can interact with the C=C double bond (B1⋯C1 1.84 Å). A subsequent 1,2-shift of B2 in a tetrahedron-like transition state **TS4** (Δ*G*^‡^ = 16.2 kcal mol^−1^) reduces the bond order and generates a new nonplanar rhombic cluster, **Int4** (Δ*G*_4_ = 0.1 kcal mol^−1^, *i.e.***Int3** and **Int4** are energetically equivalent isomers, but not in equilibrium with each other), with a long C⋯C interaction (1.77 Å). **Int4** then converts into the tautomer **C** of the 1,3-dihydro-1,3-diborete **P2BCBC** by a PMe_3_ decoordination-recoordination mechanism from B2 to C1 *via* the phosphine-free intermediate **Int5** (Δ*G*_5_ = 9.5 kcal mol^−1^, Δ*G*^‡^_R_ = 10.6 kcal mol^−1^).‡‡‡‡PMe_3_ (NBO charge: +1.24) coordinates to the more negatively charged carbon (CH: −0.72 *versus* CMe: −0.53) of the diborete ring. The total energy barrier from **P2B2C2** to **TS4** (Δ*G*^‡^ = 24.6 kcal mol^−1^) constitutes the rate-limiting step of the overall reaction from **R2** and propyne to **P2B2C2**.^65^ This explains the elevated temperature (80 °C) required over a period of several days for reaction completion. In the final step, **C** tautomerises to the slightly more stable isomer **D** (Δ*G*_C–D_ = −2.6 kcal mol^−1^). Preliminary calculations suggest that this step is likely to be an intermolecular proton exchange between the positively-charged C1 carbon of one molecule and the negatively-charged C2 carbon of a second molecule (see Fig. S60 in the ESI for details†). The fact that **C** is the major or sole tautomer observed in the experimentally-realised systems **8-R** (R = Bu, Tol, SiMe_3_, Fc) is consistent with the increased steric bulk of the mesityl (experimental) boron substituent relative to the phenyl substituents present in the computational model, the former being far more likely to hinder an intermolecular approach. The intermolecular proton transfer would therefore only be possible in the least sterically hindered analogues, **8-Bu** and **8-Tol**, for which a small proportion of **D** is indeed observed.

## Conclusions

The reactions of terminal alkynes with doubly base-stabilised diborenes of the form R(L)B=B(L)R (L = Lewis base, R = anionic substituent) result in different outcomes, largely depending on the relative predominance of kinetic or thermodynamic control, the nature of the Lewis base L and the conformation (acyclic or cyclic) of the diborene. With NHC-stabilised diborenes the kinetic product tends to be the hydroalkynylation product, which is obtained with exclusive *anti* selectivity. Computational analyses show that this *anti* selectivity results from the rearrangement of the expected *syn* addition product through decoordination–recoordination of one NHC ligand and the formation of a singly NHC-stabilized hydride-bridged diborane intermediate. The thermodynamic product is the 1,2-dihydro-1,2-diborete, resulting from a [2 + 2] cycloaddition reaction between the B=B double and C≡C triple bonds. In all cases, however, the strained B_2_C_2_ ring undergoes spontaneous ring expansion by activating either an endo- or exocyclic C–N bond at one NHC ligand, thereby providing access to novel fused B, N-heterocycles.

With phosphine-stabilised diborenes experimental and computational data show that the 1,2-dihydro-1,2-diborete cycloaddition product is both the kinetic and thermodynamic product. For acyclic diborenes, this 1,2-dihydro-1,2-diborete invariably rearranges to a thermodynamically more stable, homoaromatic 1,3-dihydro-1,3-diborete, effectively resulting in the scission of both the original B=B double and C≡C triple bonds. Calculations show that this rearrangement proceeds *via* a four-step mechanism, with loss of one phosphine ligand and migration of the other phosphine from boron to the adjacent carbon atom. Depending on the substitution of the terminal alkyne precursor, two tautomers of this phosphine-stabilised 1,3-dihydro-1,3-diborete are obtained, differing by the attachment of the former acetylenic proton to either of the carbon atoms of the BCBC ring. Computational results suggest a bimolecular mechanism for this tautomerisation, which would indeed account for the observed selectivity. The elusive 1,2-dihydro-1,2-diborete cycloaddition product was finally stabilised by employing a cyclic diborene, the bridging benzylphosphine ligand of which prevents the 1,2-to-1,3-rearrangement.

## Author contributions

H. B. supervised the study. L. E., U. S., M. D., M. P., T. E. S., A. H., A. P. and F. R. carried out the synthetic work. M. A., M. H., J. M., D. P., A. R., K. R., F. S. and T. T. carried out the X-ray crystallographic analyses. J. O. C. J.-H. carried out the computational studies. M. A. prepared the manuscript. L. E., U. S., M. D., T. E. S., A. H., M. A. and J. O. C. J.-H. prepared the ESI. All authors read and commented on the manuscript.

## Conflicts of interest

The authors declare no conflict of interest.

## Supplementary Material

SC-012-D1SC02081A-s001

SC-012-D1SC02081A-s002
