# Supplementary material for: Reactions of diborenes with terminal alkynes: mechanisms of ligand-controlled anti-selective hydroalkynylation, cycloaddition and C[triple bond, length as m-dash]C triple bond scission
Source: Chem Sci. 2021 Jun 21;12(27):9506–15. doi: 10.1039/d1sc02081a (PMC8314202; doi:10.1039/d1sc02081a)
Supplement: SC-012-D1SC02081A-s001 [file SC-012-D1SC02081A-s001.pdf]

Electronic Supplementary Material (ESI) for Chemical Science.
This journal is © The Royal Society of Chemistry 2021

Supporting Information for

Reactions of diborenes with terminal alkynes: mechanisms of ligand-controlled *anti*-selective hydroalkynylation, cycloaddition and C≡C triple bond scission

Lukas Englert,[a,b] Uwe Schmidt,[a,b] Michael Dömling,[a,b] Max Passargus,[a,b] Tom E. Stennett,[a,b] Alexander Hermann,[a,b] Merle Arrowsmith,[a,b] Marcel Härterich,[a,b] Jonas Müssig,[a,b] Alexandra Phillipps,[a,b] Dominic Prieschl,[a,b] Anna Rempel,[a,b] Felix Rohm,[a,b] Krzysztof Radacki,[a,b] Fabian Schorr,[a,b] Torsten Thiess,[a,b] J. Oscar C. Jiménez-Halla,[c] Holger Braunschweig*[a,b]

[a] Institute for Inorganic Chemistry, Julius-Maximilians-Universität Würzburg, Am Hubland, 97074 Würzburg, Germany.

[b] Institute for Sustainable Chemistry & Catalysis with Boron, Julius-Maximilians-Universität Würzburg, Am Hubland, 97074 Würzburg, Germany.

[c] Departamento de Química, Universidad de Guanajuato, Noria Alta S/N, 36050 Guanajuato, México.

Table of Contents

Methods and materials 2
Synthetic procedures 3
NMR spectra of cleanly isolated compounds 12
^{1}H and ^{11}B NMR spectra of crude isolated products 42
^{11}B NMR spectra of reaction mixtures prior to work-up 50
X-ray crystallographic data 64
Computational details 70
References 109

Methods and materials

All manipulations were performed either under an atmosphere of dry argon or *in vacuo* using standard Schlenk line or glovebox techniques. Deuterated solvents were dried over molecular sieves and degassed by three freeze-pump-thaw cycles prior to use. All other solvents were distilled and degassed from appropriate drying agents. Solvents were stored under argon over activated 4 Å molecular sieves. NMR spectra were acquired on a Bruker Avance 400 or 500 NMR spectrometer. Chemical shifts (δ) are provided in ppm and internally referenced to the carbon nuclei ($^{13}C\{^1H\}$) or residual protons (^1H) of the solvent. Heteronuclei NMR spectra are referenced to external standards (^{11}B: $BF_3{\cdot}OEt_2$; ^{31}P: 85% H_3PO_4 in H_2O). Microanalyses (C, H, N, S) were performed on an Elementar vario MICRO cube elemental analyzer. High-resolution mass spectrometry data were obtained from a Thermo Scientific Exactive Plus spectrometer in LIFDI or ASAP mode.

Reagents were purchased from Sigma Aldrich or Alfa Aesar and used as received. Ferrocenylacetylene,[1] and the diborenes (I*i*Pr)$_2$B$_2$(C(Me)=CH*t*Bu)$_2$ (**1**, I*i*Pr = 1,3-di*iso*propylimidazol-2-ylidene),[2] (I*i*Pr)$_2$B$_2$(1,1′-μ^2-(C$_5$H$_4$)$_2$Fe)$_2$ (**3**),[3] (IMe)$_2$B$_2$Tn$_2$ (**6^{Tn}**, IMe = 1,3-dimethylimidazol-2-ylidene, Tn = 2-thienyl),[4] (IMe)$_2$B$_2$Fu$_2$ (**6^{Fu}**, Fu = 2-methylfuran-5-yl),[4] (Me$_3$P)$_2$B$_2$Mes$_2$ (**I**, Mes = 2,4,6-trimethylphenyl = mesityl)[5] and 1-PMe$_3$-2-Br-(μ^2-BnPCy$_2$)B$_2$ (**10**, BnPCy$_2$ = 1-dicyclohexylphospinomethyl-benz-2-yl)[6] were prepared according to known literature procedures.

Synthetic procedures

Synthesis of 2-Fc

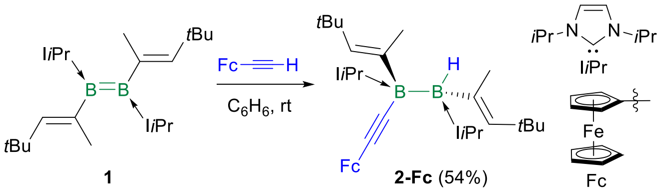

A solution of ferrocenylacetylene (8.07 mg, 38.4 μmol) in benzene (0.5 mL) was added to diborene **1** (20.0 mg, 38.4 μmol). After a couple of hours the reaction mixture turned orange, whereupon all volatiles were removed *in vacuo*. The solid residue was extracted with hexanes (2 mL) and the filtrate stored at –30 °C to yield **2-Fc** as a yellow crystalline solid (15.3 mg, 20.9 μmol, 54 %). ^{1}H NMR (500.1 MHz, 296 K, C_6D_6): δ = 6.40 (s, 2H, NC*H*), 6.36 (s, 2H, NC*H*), 6.35 (sept, ^{3}J = 6.6 Hz, 2H, *i*Pr-C*H*), 6.24 (s, 1H, *H*C*t*Bu), 5.99 (br, 2H, *i*Pr-C*H*), 5.39 (m, 1H, *H*C*t*Bu), 4.42 (m, 1H, Cp-$H_{2/5}$), 4.32 (m, 1H, Cp-$H_{2/5}$), 4.25 (s, 5H, Cp-$H_{1\text{-}5}$), 4.01 (m, 2H, Cp-$H_{3/4}$), 2.45 (br, 1H, B*H*), 1.94 (d, ^{4}J = 1.2 Hz, 3H, BCCH_3), 1.74 (d, ^{4}J = 1.2 Hz, 3H, BCCH_3), 1.44 (s, 9H, *t*Bu-CH_3), 1.42 (d, ^{3}J = 6.7 Hz, 12H, *i*Pr-CH_3), 1.30 (d, ^{3}J = 6.7 Hz, 6H, *i*Pr-CH_3), 1.28 (d, ^{3}J = 6.7 Hz, 6H, *i*Pr-CH_3), 1.17 (s, 9H, *t*Bu-CH_3). ^{13}C{^{1}H} NMR (125.8 MHz, 296 K, C_6D_6): δ = 177.4 ($C_{carbene}$, detected by HMBC), 172.7 ($C_{carbene}$, detected by HMBC), 139.2 (H*C*=CB), 133.3 (H*C*=CB), 115.5 (N*C*H), 114.7 (*NCH*), 94.2 (Cp*C*≡C), 74.7 (*i*-Cp-C_q), 70.7 (Cp-*C*$H_{2/5}$), 70.6 (Cp-*C*$H_{2/5}$), 69.7 (Cp-*C*$H_{1\text{-}5}$), 67.2 (Cp-*C*$H_{3/4}$), 67.1 (Cp-*C*$H_{3/4}$), 48.6 (*i*Pr-*C*H), 48.1 (*i*Pr-*C*H), 33.6 (*t*Bu-C_q), 33.4 (*t*Bu-C_q), 32.8 (*t*Bu-CH_3), 32.5 (*t*Bu-*C*H_3), 24.7 (*i*Pr-*C*H_3), 24.5 (*i*Pr-*C*H_3), 24.4 (*i*Pr-*C*H_3), 24.2 (BC*C*H_3), 20.2 (BC*C*H_3). *Note: the boron-bound carbon of the ferrocenylacetylene unit was not detected, presumably due to quadrupolar broadening.* ^{11}B{^{1}H}NMR (160.5 MHz, 296 K, C_6D_6): δ = –18.6 (s) ppm. Elemental analysis calculated for [$C_{44}H_{68}B_2FeN_4$] (M_W = 730.49): C 72.34, H 9.38, N 7.67%; found: C 72.37, H 9.60, N 7.75 %.

Synthesis of 4-H

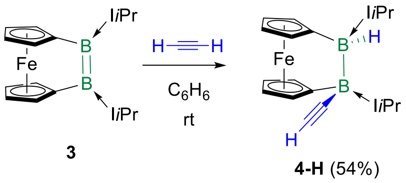

A solution of diborene **3** (115 mg, 225 μmol) in 5 mL benzene was frozen with liquid nitrogen and the argon atmosphere exchanged by an atmosphere of acetylene. The reaction was allowed

to slowly warm to room temperature, turning orange. After filtration and removal of the solvent *in vacuo*, the solid residue was washed with hexane (3 x 5 mL) and dried *in vacuo* to afford **4-H** as a crude orange solid (65 mg, 146 μmol, 54%). Multiple attempts to obtain an analytically pure sample of **4-H** failed. A handful of single crystals suitable for X-ray crystallographic analysis provided structural proof. $^{1}H\{^{11}B\}$ NMR (400 MHz, C_6D_6, 297 K): δ = 6.18 (s, 4H, CH_{IiPr}), 6.05 (m, 4H, CH_{-iPr}), 5.82 (s, 1H, CH_{Cp}), 5.63 (s, 1H, CH_{Cp}), 5.24 (s, 1H, CH_{Cp}), 4.71 (s, 2H, CH_{Cp}), 4.60 (s, 1H, CH_{Cp}), 4.45 (s, 1H, CH_{Cp}), 4.33 (s, 1H, CH_{Cp}), 3.15 (s, 1H, B*H*), 2.70 (s, 1H, C≡C*H*), 1.23 (d, ^{3}J = 6.47 Hz, 6H, $CH_{3\text{-}iPr}$), 1.16 (d, ^{3}J = 6.67 Hz, 6H, $CH_{3\text{-}iPr}$), 0.93 (d, ^{3}J = 6.76 Hz, 6H, $CH_{3\text{-}iPr}$), 0.61 (d, ^{3}J = 6.73 Hz, 6H, $CH_{3\text{-}iPr}$) ppm. ^{11}B NMR (128 MHz, C_6D_6, 297 K): δ = −18.6 ppm. HRMS-LIFDI *m/z* calculated for [$C_{30}H_{42}B_2N_4Fe$]: 536.2940; found: 536.2937.

Synthesis of 4-Ph

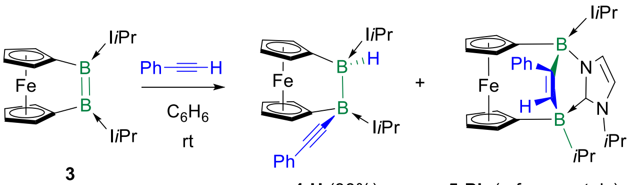

3 4-H (38%) 5-Ph (a few crystals)

A solution of diborene **3** (100 mg, 196 μmol) in 5 mL benzene was treated with phenylacetylene (50 mg, 490 μmol) and stirred for 2 h at room temperature. After filtration and removal of the solvent *in vacuo*, the solid residue was washed with hexane (3 x 5 mL) and dried *in vacuo* to afford **4-Ph** as a crude orange solid (45 mg, 74 μmol, 38%). Multiple attempts to obtain an analytically pure sample of **4-Ph** failed. One crystallisation attempt yielded a handful of crystals of the cycloaddition by-product **5-Ph**, which was only characterised structurally. $^{1}H\{^{11}B\}$ NMR (400 MHz, C_6D_6, 297 K): δ = 7.75 (m, 2H, $CH_{\text{-Ph}}$), 7.19 (m, 2H, $CH_{\text{-Ph}}$), 7.04 (m, 1H, $CH_{\text{-Ph}}$), 6.19 (s, 2H, CH_{IiPr}), 6.15 (s, 2H, CH_{IiPr}), 6.12 (m, 2H, CH_{iPr}), 6.02 (m, 2H, CH_{iPr}), 5.90 (s, 1H, CH_{Cp}), 5.61 (s, 1H, CH_{Cp}), 4.78 (s, 1H, CH_{Cp}), 4.77 (s, 1H, CH_{Cp}), 4.73 (s, 1H, CH_{Cp}), 4.63 (s, 1H, CH_{Cp}), 4.46 (s, 1H, CH_{Cp}), 4.35 (s, 1H, CH_{Cp}), 3.23 (s, 1H, B*H*), 1.29 (d, ^{3}J = 6.63 Hz, 6H, $CH_{3\text{-}iPr}$), 1.13 (d, ^{3}J = 6.68 Hz, 6H, $CH_{3\text{-}iPr}$), 0.90 (d, ^{3}J = 6.81 Hz, 6H, $CH_{3\text{-}iPr}$), 0.60 (d, ^{3}J = 6.77 Hz, 6H, $CH_{3\text{-}iPr}$) ppm. ^{11}B NMR (128 MHz, C_6D_6, 297 K): δ = −17.9 ppm. HRMS-LIFDI *m/z* calculated for [$C_{36}H_{46}B_2N_4Fe$]: 612.3253; found: 612.3233.

Synthesis of 4-Fc

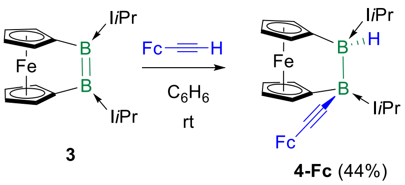

3 **4-Fc** (44%)

A mixture of diborene **3** (15 mg, 29.4 μmol) and ferrocenylacetylene (7 mg, 33.3 μmol) in 0.6 mL benzene was stirred for 2 h at room temperature After filtration and removal of the solvent *in vacuo*, the solid residue was washed with hexane (3 x 1 mL) and dried *in vacuo* to afford **4-Fc** as a crude orange solid (9 mg, 12.9 μmol, 44%). Multiple attempts to obtain an analytically pure sample of **4-Fc** failed. $^{1}H\{^{11}B\}$ NMR (400 MHz, C_6D_6, 297 K): δ = 6.19 (s, 2H, C$H_{\mathrm{I}i\mathrm{Pr}}$), 6.15 (s, 2H, C$H_{\mathrm{I}i\mathrm{Pr}}$), 6.12 (m, 2H, C$H_{i\mathrm{Pr}}$), 6.02 (m, 2H, C$H_{i\mathrm{Pr}}$), 5.90 (s, 1H, C$H_{\mathrm{Cp}}$), 5.61 (s, 1H, C$H_{\mathrm{Cp}}$), 4.78 (s, 1H, C$H_{\mathrm{Cp}}$), 4.77 (s, 1H, C$H_{\mathrm{Cp}}$), 4.73 (s, 1H, C$H_{\mathrm{Cp}}$), 4.63 (s, 1H, C$H_{\mathrm{Cp}}$), 4.46 (s, 1H, C$H_{\mathrm{Cp}}$), 4.35 (s, 1H, C$H_{\mathrm{Cp}}$), 3.23 (s, 1H, B*H*), 1.29 (d, ^{3}J = 6.63 Hz, 6H, C$H_{3\text{-}i\mathrm{Pr}}$), 1.13 (d, ^{3}J = 6.68 Hz, 6H, C$H_{3\text{-}i\mathrm{Pr}}$), 0.90 (d, ^{3}J = 6.81 Hz, 6H, C$H_{3\text{-}i\mathrm{Pr}}$), 0.60 (d, ^{3}J = 6.77 Hz, 6H, C$H_{3\text{-}i\mathrm{Pr}}$) ppm. ^{11}B NMR (128 MHz, C_6D_6, 297 K): −18.1 ppm. HRMS-LIFDI *m/z* calculated for [$C_{40}H_{50}B_2N_4Fe_2$]: 720.2915; found: 720.2894.

Synthesis of 5-Fc

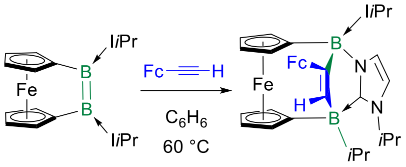

3 **5-Fc** (57%)

A mixture of diborene **3** (30 mg, 59 μmol) and ferrocenylacetylene (26 mg, 59 μmol), in 0.6 mL benzene was heated for 3 h at 60 °C. The reaction mixture turned orange. Insoluble residues were removed by filtration and the solution was stored for a few days at rt to afford compound **5-Fc** as crystalline orange solid. (24 mg, 34 μmol, 57%). ^{1}H NMR (500 MHz, C_6D_6, 297 K): δ = 8.02 (s, 1H, C=C*H*), 6.40 (d, ^{3}J = 1.7 Hz, 1H, NC*H*), 6.06 (d, ^{3}J = 2.0 Hz, 1H, NC*H*), 6.01 (d, ^{3}J = 2.0 Hz, 1H, NC*H*), 5.86 (d, ^{3}J = 1.7 Hz, 1H, NC*H*), 5.38 (sept, ^{3}J = 6.7 Hz, 2H, *i*Pr-C*H*), 5.12 (m, 1H, CH_{Cp}), 5.05 (sept, ^{3}J = 6.7 Hz, 1H, *i*Pr-C*H*), 4.68 (m, 1H, CH_{Cp}), 4.53 (m, 1H, CH_{Cp}) 4.49 (s, 5H, CH_{Cp}), 4.49 (m, 1H, CH_{Cp}), 4.40 (m, 1H, CH_{Cp}), 4.25 (m, 1H, CH_{Cp}), 4.20 (m, 1H, CH_{Cp}), 3.99 (m, 1H, CH_{Cp}), 3.93 (m, 1H, CH_{Cp}), 3.85 (m, 1H, CH_{Cp}), 3.50 (m, 1H, CH_{Cp}), 3.42 (m, 1H, CH_{Cp}), 1.85 (d, ^{3}J = 6.8 Hz, 3H, *i*Pr-CH_3), 1.74 (sept, ^{3}J = 6.8 Hz, 1H, *i*Pr-BC*H*), 1.49 (d, ^{3}J = 6.7 Hz, 3H, *i*Pr-CH_3), 1.26 (d, ^{3}J = 6.7 Hz, 3H, *i*Pr-CH_3), 1.19 (d,

^3J = 6.6 Hz, 3H, *i*Pr-C*H*$_3$), 0.85 (d, ^3J = 6.6 Hz, 6H, *i*Pr-C*H*$_3$), 0.64 (d, ^3J = 6.6 Hz, 3H, *i*Pr-C*H*$_3$), 0.49 (d, ^3J = 6.6 Hz, 3H, *i*Pr-C*H*$_3$) ppm. ^{13}C{^{1}H} NMR (126 MHz, C_6D_6, 297 K): δ = 173.1 (*C*$_{\text{Carbene}}$, detected by HMBC), 163.5 (*C*$_{\text{Carbene}}$, detected by HMBC), 153.6 (H*C*=C, detected by HSQC), 146.9 (HC=*C*, detected by HMBC), 122.4 (N*C*H), 116.8 (N*C*H), 116.1 (N*C*H), 113.1 (N*C*H), 98.3 (Fc*C*$_{\text{Cp}}$-C=CH), 77.5 (*C*H$_{\text{Cp}}$), 76.0 (*C*H$_{\text{Cp}}$), 72.4 (*C*H$_{\text{Cp}}$), 70.8 (*C*H$_{\text{Cp}}$), 70.4 (*C*H$_{\text{Cp}}$), 70.0 (5C, *C*H$_{\text{Cp}}$,), 68.5 (*C*H$_{\text{Cp}}$), 68.1 (*C*H$_{\text{Cp}}$), 67.5 (*C*H$_{\text{Cp}}$), 67.0 (*C*H$_{\text{Cp}}$), 66.3 (*C*H$_{\text{Cp}}$), 66.3 (*C*H$_{\text{Cp}}$), 65.4 (*C*H$_{\text{Cp}}$), 49.2 (*i*Pr-*C*H), 48.5 (*i*Pr-*C*H), 48.2 (*i*Pr-*C*H), 24.7 (*i*Pr-*C*H$_3$), 24.4 (*i*Pr-*C*H$_3$), 24.1 (*i*Pr-*C*H$_3$), 24.0 (*i*Pr-*C*H$_3$), 23.8 (*i*Pr-*C*H$_3$), 22.2 (*i*Pr-*C*H$_3$), 21.8 (*i*Pr-*C*H$_3$), 21.8 (*i*Pr-*C*H$_3$), 20.2 (B-*i*Pr-*C*H, detected by HSQC) ppm. *Note: The boron-bound carbon atoms in the bridging ferrocenyl unit could not be detected due to quadrupolar broadening.* ^{11}B NMR (160 MHz, C_6D_6, 297 K): δ = –7.7 (br), –14.7 (br) ppm. Elemental analysis calculated for [$C_{40}H_{50}B_2Fe_2N_4$] (M_w = 720.18): calcd. C 66.71, H 7.00, N 7.78%; found: C 66.83, H 6.71, N 7.30%.

Synthesis of 7^{Tn}-Fc

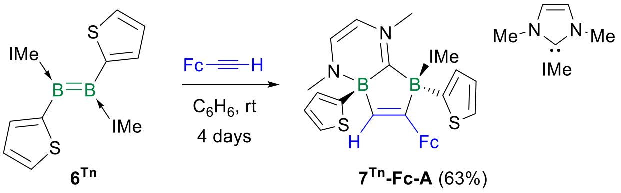

Diborene **6^{Tn}** (20.0 mg, 52.6 µmol) and ferrocenylacetylene (12.2 mg, 57.9 µmol, 1.10 equiv.) were dissolved in 0.5 mL C_6D_6. After 4 d at rt a red suspension was obtained. After removal of volatiles *in vacuo*, the brown residue was washed with diethyl ether (2 × 0.5 mL) and pentane (3 × 0.5 mL) to obtain analytically pure **7^{Tn}-Fc** as an orange solid (19.6 mg, 33.2 µmol 63%). Single crystals suitable for X-ray diffraction analysis were obtained by diffusing hexane into a saturated benzene solution at rt. ^{1}H{^{11}B} NMR (500.1 MHz, $CDCl_3$): δ = 7.23 (br s, 1H, BC*H*), 7.19 (dd, ^3J = 4.7 Hz, ^4J = 0.9 Hz, 1H, C*H*$_{\text{Tn}}$), 7.05 (dd, ^3J = 4.7 Hz, ^4J = 1.0 Hz, 1H, C*H*$_{\text{Tn}}$), 6.99 (br s, 1H, C*H*$_{\text{Tn}}$), 6.93 (dd, ^3J = 4.7 Hz, ^3J = 3.30 Hz, 1H, C*H*$_{\text{Tn}}$), 6.88 (d, ^3J = 1.9 Hz, 1H, C*H*$_{\text{IMe}}$), 6.80 (dd, ^3J = 4.7 Hz, ^3J = 3.2 Hz, 1H, C*H*$_{\text{Tn}}$), 6.79 (d, ^3J = 1.9 Hz, 1H, C*H*$_{\text{IMe}}$), 6.41 (d, ^3J = 4.7 Hz, 1H, BNC*H*), 6.26 (dd, ^3J = 3.2 Hz, ^4J = 1.0 Hz, 1H, C*H*$_{\text{Tn}}$), 5.31 (d, ^3J = 4.7 Hz, 1H, BNCHC*H*), 4.45 (m, 1H, C*H*$_{\text{Fc}}$), 4.08 (m, 1H, C*H*$_{\text{Fc}}$), 4.02 (s, 5H, $C_5H_{5\text{-Fc}}$), 3.97 (m, 1H, C*H*$_{\text{Fc}}$), 3.65 (s, 3H, C*H*$_{3\text{-IMe}}$), 3.33 (m, 1H, C*H*$_{\text{Fc}}$), 3.27 (s, 3H, C*H*$_{3\text{-IMe}}$), 3.10 (s, 3H, BNC*H*$_3$), 3.00 (s, 3H, B_2CNC*H*$_3$) ppm. ^{13}C{^{1}H} NMR (125.8 MHz, $CDCl_3$): δ = 152.6 (B*C*H, detected by HSQC), 138.9 (BN*C*H), 131.7 (*C*H$_{\text{Tn}}$), 127.5 (*C*H$_{\text{Tn}}$), 127.3 (*C*H$_{\text{Tn}}$), 127.0 (*C*H$_{\text{Tn}}$), 126.0 (*C*H$_{\text{Tn}}$), 124.2 (*C*H$_{\text{Tn}}$), 122.5 (*C*H$_{\text{IMe}}$), 121.9 (*C*H$_{\text{IMe}}$), 102.0 (BNCH*C*H), 93.6 (*C*$_{\text{q-Fc}}$) 70.0

(CH_{Fc}), 69.4 ($C_5H_{5\text{-}Fc}$), 66.9 (CH_{Fc}), 66.6 (CH_{Fc}), 66.1 (CH_{Fc}), 46.3 (BNCH_3), 39.6 (B_2CNCH_3), 37.4 ($CH_{3\text{-}IMe}$), 35.7 ($CH_{3\text{-}IMe}$) ppm. *Note: The resonances of the boron-bound carbon atoms could not be identified due to quadrupolar broadening by coupling to boron.* ^{11}B NMR (160.5 MHz, CDCl3): $\delta = -4.6$ (s), -11.5 (s) ppm. LIFDI-MS – pos-ESI-MS *m/z* calculated for $[C_{30}H_{31}B_2FeN_4S_2]^+ = [M + H]^+$: 589.1635; found: 589.1631.

Synthesis of 7^{Fu}-Fc

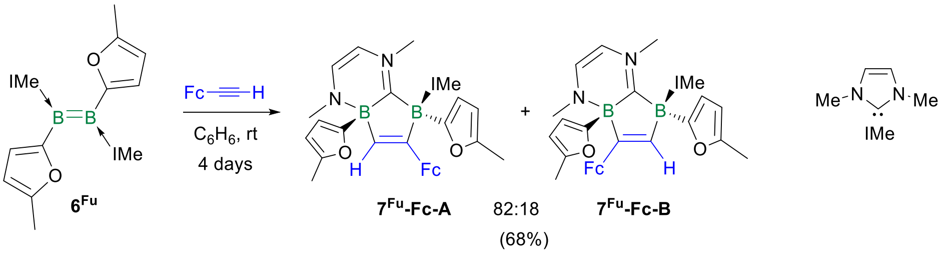

Diborene **6^{Fu}** (14.0 mg, 37.2 µmol) and ferrocenylacetylene (8.60 mg, 40.9 µmol, 1.10 equiv.) were dissolved in 0.5 mL C_6D_6. After 2 d at rt a red solution was obtained. A crude orange product (14.8 mg, 25.3 µmol, 68%) was isolated by drying and washing with pentane (2 × 0.5 mL). Multiple attempts to obtain analytically pure product failed but the ^{11}B NMR spectrum of the crude product mixture confirms the formation of **7^{Fu}-Fc-A** as the major isomer (82%) and **7^{Fu}-Fc-B** as the minor isomer (18%). ^{11}B NMR (160.5 MHz, C_6D_6): $\delta = -6.0$ (s), -12.7 (s) ppm. LIFDI-MS – pos-ESI-MS *m/z* calculated for $[C_{32}H_{35}B_2FeN_4O_2]^+ = [M + H]^+$: 585.2405; found: 585.2399.

Synthesis of 8-Bu

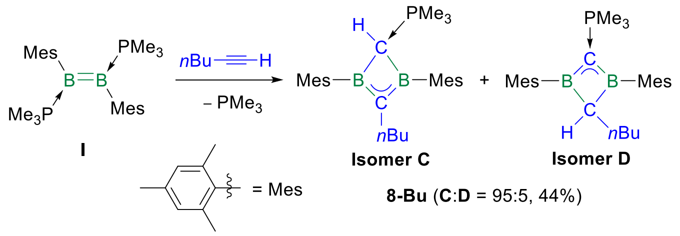

100 mg of diborene **I** (243 µmol, 1.00 eq.) and 30.0 µL of hex-1-yne (21.4 mg, 260 µmol, 1.07 eq.) were dissolved in 5 mL benzene and the yellow solution was heated for 3 days at 80 °C. After removal of all volatiles under reduced pressure, the colourless crude product was washed with 3 x 5 mL pentane, and the resulting colourless **8-Bu** dried in *vacuo* (44.7 mg, 107 µmol, 44% yield). Crystals suitable for X-ray diffraction analysis were obtained by slow evaporation of a saturated pentane solution at room temperature. NMR data showed that the compound is present in solution at rt as a 95:5 mixture of two tautomers (by integration of the ^{31}P NMR

resonances). **Isomer C**: ^{1}H NMR (500.1 MHz, C_6D_6): δ = 6.96 (s, 4H, *m*-Mes-C*H*), 2.83 (app. q, $^3J_{1H-1H}$ = $^4J_{31P-1H}$ = 7.6 Hz, 2H, CH_2CHB), 2.59 (s, 12H, *o*-Mes-CH_3), 2.30 (s, 6H, *p*-Mes-CH_3), 1.66–1.72 (m, 3H, CH_2CH$_2$CHB + B$_2$C*H*P), 1.45 (app. hex., $^3J_{1H-1H}$ = 7.5 Hz, 2H, CH$_3$CH_2), 0.89 (t, $^3J_{1H-1H}$ = 7.4 Hz, 3H, CH_3), 0.60 (d, $^2J_{31P-1H}$ = 12.3 Hz, 9H, PCH_3) ppm. ^{13}C{^{1}H} NMR (125.8 MHz, C_6D_6): δ = 172.6 (br, B*C*P), 141.7 (*i*-Mes-C_q), 139.7 (*o*-Mes-C_q), 135.0 (*p*-Mes-C_q), 127.9 (*m*-Mes-*C*H), 34.3 (d, $^4J_{31P-13C}$ = 5.6 Hz, *C*H$_2$), 32.9 (d, $^5J_{31P-13C}$ = 4.1 Hz, *C*H$_2$), 24.3 (br, B*C*H), 23.7 (*o*-Mes-*C*H$_3$), 23.5 (*C*H$_2$), 21.3 (*p*-Mes-*C*H$_3$), 14.4 (*C*H$_3$), 12.5 (d, $^1J_{31P-13C}$= 59.5 Hz, P*C*H$_3$) ppm. ^{11}B NMR (160.5 MHz, C_6D_6): δ = 29.8 (br s, fwmh ≈ 398 Hz) ppm. ^{31}P{^{1}H} NMR (202.5 MHz, C_6D_6): δ = 19.21 (s) ppm. **Isomer D**: ^{1}H NMR (400.1 MHz, C_6D_6): δ = 6.93 (s, 4H, *m*-Mes-C*H*), 2.60 (s, 1H, B$_2$C*H*), 2.56 (br s, 12H, *o*-Mes-CH_3), 2.30 (s, 6H, *p*-Mes-CH_3), 1.95-1.85 (m, 3H, CH_2), 1.60-1.53 (m, 2H, CH_2), 1.43-1.34 (m, 2H, CH_2), 0.85-0.80 (m, 3H, CH_3), 0.55 (d, $^2J_{31P-1H}$ = 13.0 Hz, 9H, PCH_3) ppm. ^{11}B NMR (128.4 MHz, C_6D_6): δ = 54.7 (br s, fwmh ≈ 642 Hz) ppm. ^{31}P{^{1}H} NMR (262.0 MHz, C_6D_6): δ = −3.61 (s) ppm. Elemental analysis calculated for [$C_{27}H_{41}B_2P$] (M_W = 418.22): C 77.54, H 9.88%; found: C 77.23, H 10.10%.

Synthesis of 8-TMS

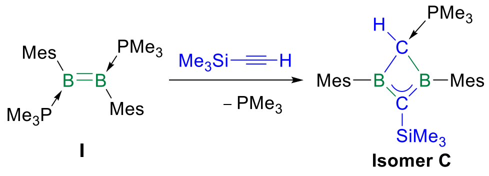

8-TMS (R = SiMe$_3$, 41%)

50 mg of diborene **I** (121 µmol, 1.00 eq.) and 17.3 µL of trimethylsilylacetylene (12.0 mg, 121 µmol, 1.00 eq.) were dissolved in 5 mL benzene and the yellow solution was heated for 6 days at 80 °C. After removal of all volatiles under reduced pressure, the colourless crude product was washed with 3 x 5 mL pentane, and the resulting colourless **8-TMS** dried in *vacuo* (21.8 mg, 50.2 µmol, 41% yield). Crystals suitable for X-ray diffraction analysis were obtained by slow evaporation of a saturated toluene solution at −30 °C. ^{1}H NMR (500.1 MHz, C_6D_6): δ = 6.93 (s, 4H, *m*-Mes-C*H*), 2.59 (s, 12H, *o*-Mes-CH_3), 2.28 (s, 6H, *p*-Mes-CH_3), 1.79 (d, $^2J_{31P-1H}$ = 6.9 Hz, 1H, B$_2$C*H*P), 0.57 (d, $^2J_{31P-1H}$ = 12.5 Hz, 9H, PCH_3), 0.30 (s, 9H, SiCH_3) ppm. ^{13}C{^{1}H} NMR (125.8 MHz, C_6D_6): δ = 159.4 (br, B*C*B), 143.0 (br, *i*-Mes-C_q), 138.4 (*o*-Mes-C_q), 135.0 (*p*-Mes-C_q), 127.9 (*m*-Mes-*C*H), 26.3 (d, $^1J_{31P-13C}$ = 69.0 Hz, B*C*H), 24.0 (*o*-Mes-*C*H$_3$), 21.3 (*p*-Mes-*C*H$_3$), 12.4 (d, $^1J_{31P-13C}$ = 59.0 Hz, P*C*H$_3$), 1.4 (d, $^4J_{31P-13C}$ = 2.2 Hz, Si*C*H$_3$) ppm. ^{11}B NMR (160.5 MHz, C_6D_6): δ = 38.8 (br s, fwmh ≈ 440 Hz) ppm. ^{31}P{^{1}H} NMR (202.5

MHz, C_6D_6): δ = 15.62 (s) ppm. Elemental analysis calculated for [$C_{26}H_{41}B_2PSi$] (M_W = 434.29): C 71.91, H 9.52%; found: C 71.65, H 9.81%.

Synthesis of 8-Tol

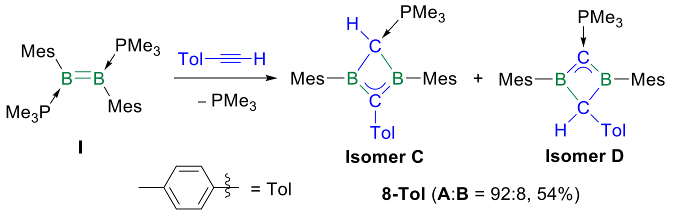

50 mg of diborene **I** (121 µmol, 1.00 eq.) and 15.4 µL of 4-methyl-1-ethynylbenzene (14.1 mg, 121 µmol, 1.00 eq.) were dissolved in 15 mL benzene and the yellow solution was heated for 6 days at 80 °C. After removal of all volatiles under reduced pressure, the colourless crude product was washed with 3 x 5 mL pentane, and the resulting colourless **8-Tol** dried in *vacuo* (29.4 mg, 65.0 µmol, 54% yield). Crystals suitable for X-ray diffraction analysis were obtained by slow evaporation of a saturated toluene solution at −30 °C. NMR data showed that the compound is present in solution at rt as a 92:8 mixture of two tautomers (by integration of the ^{31}P NMR resonances). **Isomer C**: ^{1}H NMR (500.1 MHz, C_6D_6): δ = 7.55 (d, $^{3}J_{1H-1H}$ = 7.9 Hz, 2H, *m*-Tol-C*H*), 7.01 (d, $^{3}J_{1H-1H}$ = 7.9 Hz, 2H, *o*-Tol-C*H*), 6.94 (s, 4H, *m*-Mes-C*H*), 2.55 (s, 12H, *o*-Mes-CH_3), 2.30 (s, 6H, *p*-Mes-CH_3), 2.13 (s, 3H, *p*-Tol-CH_3), 1.65 (d, $^{2}J_{31P-1H}$ = 3.2 Hz, 1H, B_2C*H*P), 0.50 (d, $^{2}J_{31P-1H}$ = 12.5 Hz, 9H, PCH_3) ppm. ^{13}C{^{1}H} NMR (125.8 MHz, C_6D_6): δ = 162.9 (br, B*C*B), 141.7 (*i*-Tol-C_q), 141.4 (br, *i*-Mes-C_q), 139.9 (*o*-Mes-C_q), 135.6 (*p*-Mes-C_q), 132.6 (d, $^{4}J_{31P-13C}$ = 1.8 Hz, *i*-Tol-C_q), 129.1 (*o*-Tol-*C*H), 128.4 (*m*-Tol-*C*H), 128.2 (*m*-Mes-*C*H), 23.6 (*o*-Mes-*C*H_3), 21.7 (br d, $^{1}J_{31P-13C}$ = 59.8 Hz, *C*H), 21.4 (*p*-Mes-*C*H_3), 21.3 (*p*-Tol-*C*H_3), 11.7 (d, $^{1}J_{P-C}$= 56.9 Hz, P*C*H_3) ppm. ^{11}B NMR (160.5 MHz, C_6D_6): δ = 33.8 (br s, fwmh ≈ 357 Hz) ppm. ^{31}P{^{1}H} NMR (202.5 MHz, C_6D_6): δ = 18.68 (s) ppm. **Isomer D**: ^{1}H NMR (500.1 MHz, C_6D_6): δ = 7.31 (d, $^{3}J_{1H-1H}$ = 8.0 Hz, 2H, *m*-Tol-C*H*), 6.89 (d, $^{3}J_{1H-1H}$ = 7.7 Hz, 2H, *o*-Tol-C*H*), 6.87 (s, 4H, *m*-Mes-C*H*), 3.43 (d, $^{4}J_{31P-1H}$ = 4.6 Hz, 1H, B_2C*H*), 2.53 (br s, 12H, *o*-Mes-CH_3), 2.26 (s, 6H, *p*-Mes-CH_3), 2.07 (s, 3H, *p*-Tol-CH_3), 0.56 (d, J_{31P-1H} = 13.0 Hz, 9H, PCH_3) ppm. ^{11}B NMR (160.5 MHz, C_6D_6): δ = 51.4 (br s) ppm. ^{31}P{^{1}H} NMR (202.5 MHz, C_6D_6): δ = −2.74 (s) ppm. Elemental analysis calculated for [$C_{30}H_{39}B_2P$] (M_W = 452.24): C 79.68, H 8.69%; found: C 78.66, H 8.74%.

Synthesis of 8-Fc

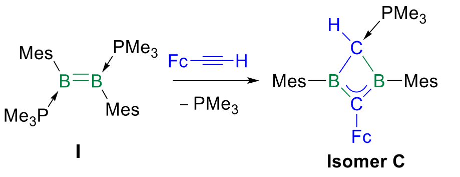

100 mg of diborene **I** (243 µmol, 1.00 eq.) and 51.0 mg of ferrocenylacetylene (243 µmol, 1.00 eq.) were dissolved in 8 mL benzene and the red solution was heated for 3 days at 80 °C. After removal of all volatiles under reduced pressure, the orange crude product was washed with 3 x 5 mL pentane, and the resulting orange-coloured **8-Fc** dried in *vacuo* (78.0 mg, 143 µmol, 59% yield). Crystals suitable for X-ray diffraction analysis were obtained by slow evaporation of a saturated benzene solution at room temperature. $^{1}H\{^{11}B\}$ NMR (500.1 MHz, C_6D_6): δ = 6.98 (s, 4H, *m*-Mes-C*H*), 4.42 (m, 2H, Cp-$H_{2/5}$), 4.10 (s, 5H, Cp-$H_{1\text{-}5}$), 4.04 (t, $^{3}J_{1H\text{-}1H}$ = 1.8 Hz, 2H, Cp-$H_{3/4}$), 2.62 (s, 12H, *o*-Mes-CH_3), 2.33 (s, 6H, *p*-Mes-CH_3), 1.58 (d, $^{2}J_{31P\text{-}1H}$ = 5.6 Hz, 1H, B_2C*H*P), 0.58 (d, $^{2}J_{31P\text{-}1H}$ = 12.5 Hz, 9H, PCH_3) ppm. $^{13}C\{^{1}H\}$ NMR (125.8 MHz, C_6D_6): δ = 156.8 (br, B*C*Fc), 142.1 (br, *i*-Mes-C_q), 139.2 (*o*-Mes-C_q), 135.4 (*p*-Mes-C_q), 127.9 (*m*-Mes-*C*H), 89.7 (d, $^{4}J_{31P\text{-}13C}$ = 4.0 Hz, *i*-Cp-C_q), 69.0 (Cp-*C*$H_{1\text{-}5}$), 68.4 (d, ^{5}J = 1.5 Hz, Cp-*C*$H_{2/5}$), 67.4 (Cp-*C*$H_{3/4}$), 23.9 (*o*-Mes-*C*H_3), 22.7 (d, $^{1}J_{31P\text{-}13C}$ = 65.5 Hz, *C*H), 21.4 (*p*-Mes-*C*H_3), 12.1 (d, $^{1}J_{31P\text{-}13C}$ = 58.1 Hz, P*C*H_3) ppm. ^{11}B NMR (160.5 MHz, C_6D_6): δ = 33.9 (br s, fwmh ≈ 450 Hz) ppm. $^{31}P\{^{1}H\}$ NMR (202.5 MHz, C_6D_6): δ = 17.18 (s) ppm. Elemental analysis calculated for [$C_{33}H_{41}B_2FeP$] (M_W = 546.13): C 72.58, H 7.57%; found: C 72.48, H 7.47%.

Synthesis of 11-TMS

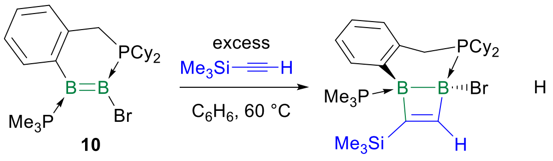

Diborene **10** (30 mg, 64 µg) was dissolved in benzene (0.6 mL) in an NMR tube with a J. Young valve. The solution was frozen and trimethylsilylacetylene (ca. 50 µl, excess) was added by vacuum transfer. After thawing, the solution was placed in a 60 °C heating block for 16 h. Volatiles were removed under reduced pressure, and the pale yellow residue was dissolved in Et_2O (ca. 0.4 mL) and stored at –30 °C, yielding several small batches of crystals of **11-TMS** (total yield 21 mg, 58%). These crystals were suitable for the X-ray diffraction analysis. $^{1}H\{^{11}B\}$ NMR (500.1 MHz, C_6D_6): δ = 8.16 (d, 1H, $^{3}J_{31P\text{-}1H}$ = 6.0 Hz, Me_3SiC=C*H*), 7.25 (app.

d, $^3J_{1H-1H}$ = 7.1 Hz, 1H, Ar-C*H*), 7.21 (app. t, $^3J_{1H-1H}$ = 7.1 Hz, 1H, Ar-C*H*), 7.12 (app. t, $^3J_{1H-1H}$ = 7.1 Hz, 1H, Ar-C*H*), 6.99 (app. d, $^3J_{1H-1H}$ = 7.1 Hz, 1H, Ar-C*H*), 3.10 (t, $^2J_{31P-1H}$ = $^2J_{1H-1H}$ = 13.4 Hz, 1H, PCH_2), 2.29 (t, $^2J_{31P-1H}$ = $^2J_{1H-1H}$ = 13.4 Hz, 1H, PCH_2), 2.23-2.13 (m, 1H, Cy-C*H*), 2.13-1.99 (m, 3H, Cy-CH_2), 1.83-1.73 (m, 1H, Cy-C*H*), 1.73-1.37 (m, 10H, Cy-CH_2), 1.24 (d, 9H, $^2J_{31P-1H}$ = 10.3 Hz, P(CH_3)$_3$), 1.18-0.78 (m, 7H, Cy-CH_2), 0.09 (s, 9H, Si(CH_3)$_3$). ^{13}C{^{1}H} NMR (125.8 MHz, C_6D_6): δ = 167.7 (br s, Me_3SiC*C*H), 166.2 (br s, Me_3Si*C*CH), 150.9 (v br, Ar-*C*B), 139.4 (dd, $^2J_{31P-13C}$ = 18.8, $^3J_{31P-13C}$ = 5.4 Hz, Ar-*C*CH_2P), 130.6 (br m, Ar-*C*H), 129.6 (dd, $^3J_{31P-13C}$ = 6.8, $^4J_{31P-13C}$ = 3.2 Hz, Ar-*C*H), 126.1 (d, $^4J_{31P-13C}$ = 1.7 Hz, Ar-*C*H), 124.3 (Ar-*C*H), 34.8 (br d, $^1J_{31P-13C}$ = 25.4 Hz, P*C*H), 31.8 (d, $^1J_{31P-13C}$ = 33.4 Hz, P*C*H), 28.8 (Cy-*C*H_2), 28.0 (Cy-*C*H_2), 27.9 (d, $^2J_{31P-13C}$ = 11.0 Hz, Cy-*C*H_2), 27.7 (d, $^2J_{31P-13C}$ = 8.8 Hz, Cy-*C*H_2), 27.6 (d, $^2J_{31P-13C}$ = 3.0 Hz, Cy-*C*H_2), 27.5 (d, $^3J_{31P-13C}$ = 2.8 Hz, Cy-*C*H_2), 27.3 (d, $^3J_{31P-13C}$ = 2.2 Hz, Cy-*C*H_2), 27.2 (d, $^2J_{31P-13C}$ = 5.7 Hz, Cy-*C*H_2), 26.5 (d, $^3J_{31P-13C}$ = 1.1 Hz, Cy-*C*H_2), 26.3 (d, $^3J_{31P-13C}$ = 1.2 Hz, Cy-*C*H_2), 25.2 (d, $^1J_{31P-13C}$ = 40.3 Hz, P*C*H_2), 12.3 (br. d, $^1J_{31P-13C}$ = 37.0 Hz, P(*C*H_3)$_3$), –0.1 (d, $^3J_{31P-13C}$ = 1.7 Hz, Si(*C*H_3)$_3$). ^{11}B NMR (160.5 MHz, C_6D_6): δ = –16.0 (br s), –16.5 (br s). ^{31}P{^{1}H} NMR (202.5 MHz, C_6D_6): δ = 8.7 (br, PCy_2), –12.9 (br d, $^3J_{31P-31P}$ = 107 Hz, PMe_3). ^{29}Si NMR (99.4 MHz, C_6D_6): δ = –14.5 (s, *Si*Me_3). LIFDI-MS – pos-ESI-MS *m/z* calculated for $[C_{24}H_{38}B_2BrPSi]^+$ = [M + H – PMe_3]$^+$: 487.1928; found: 487.1923.

NMR spectra of cleanly isolated compounds

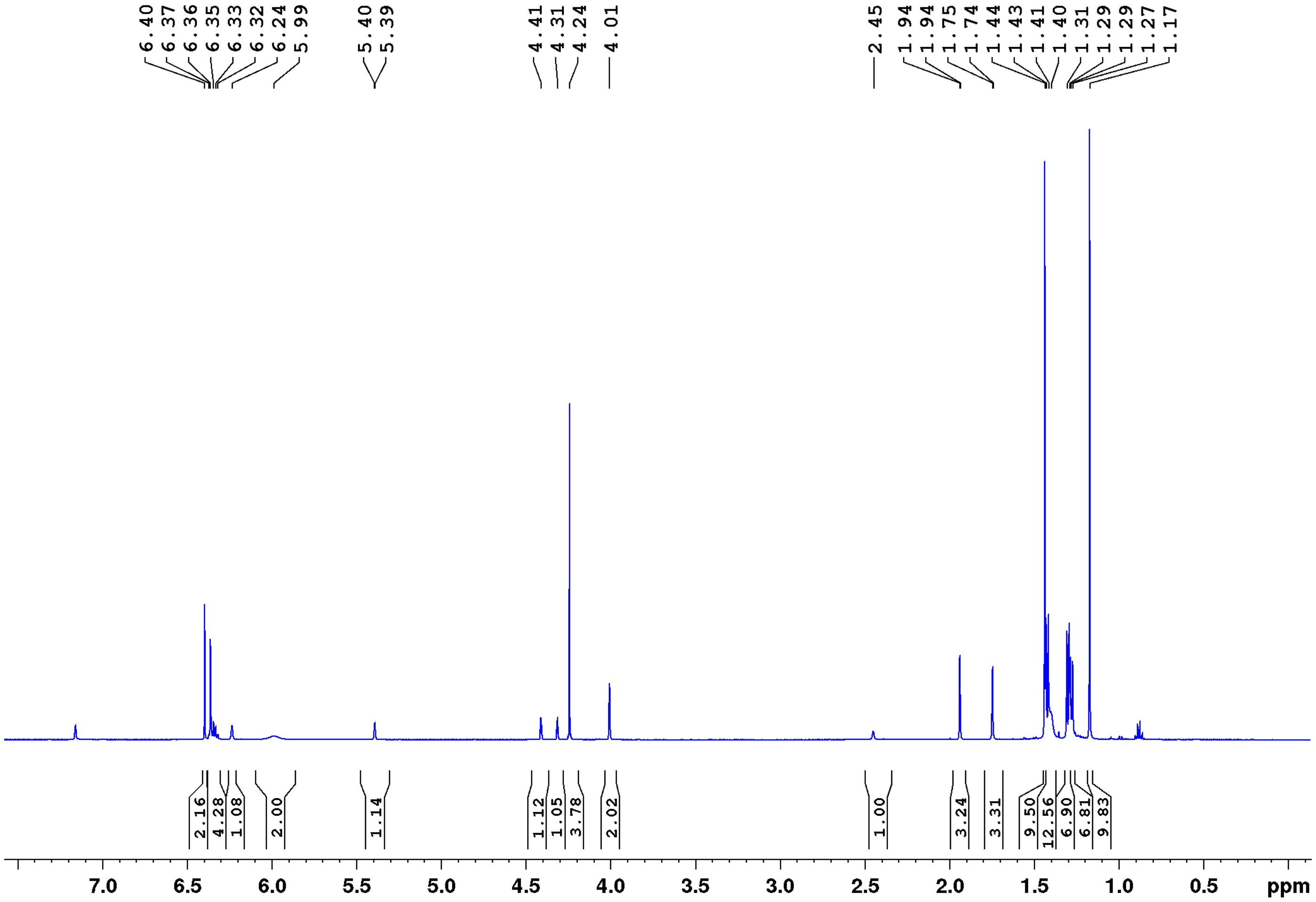

Figure S1. $^{1}H\{^{11}B\}$ NMR spectrum of **2-Fc** in C_6D_6. The additional triplets at 0.87 and 0.89 ppm belong to residual pentane and hexane used for washing, respectively.

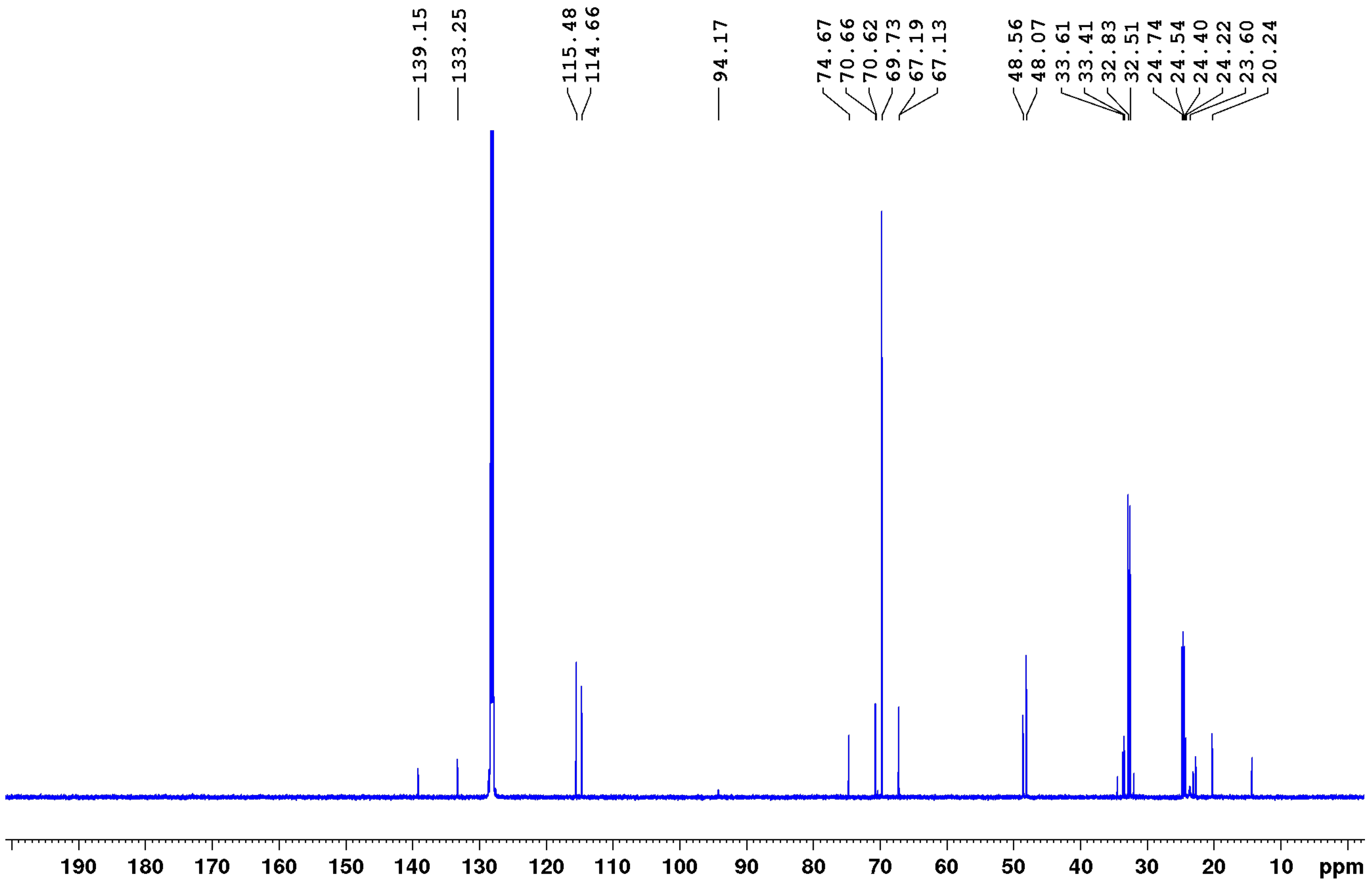

Figure S2. $^{13}C\{^1H\}$ NMR spectrum of **2-Fc** in C_6D_6. The additional resonances at 14.3, 22.7 and 34.5 ppm belong to residual pentane, those at 14.3, 23.0 and 31.0 ppm to hexane used for washing.

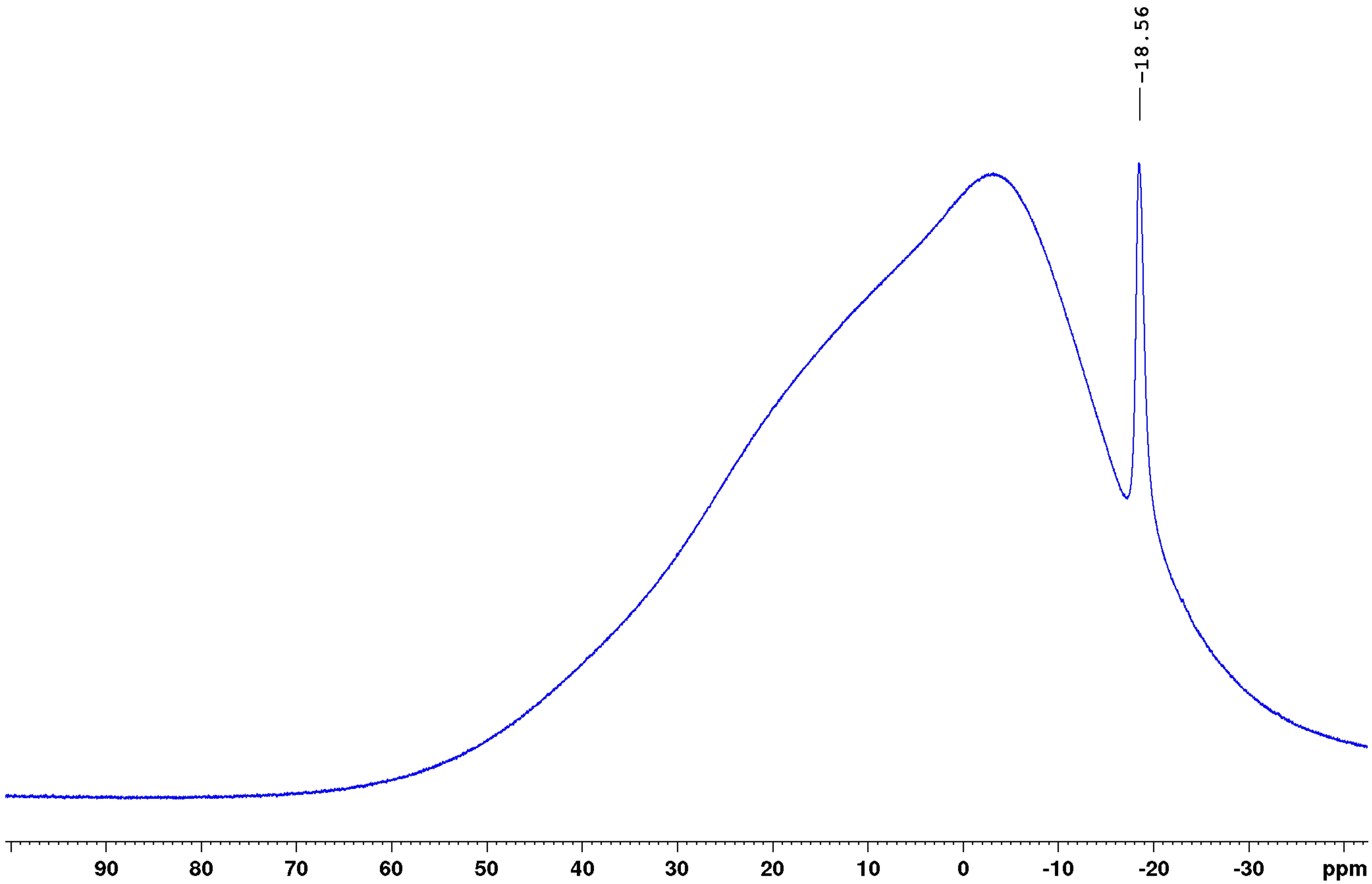

Figure S3. $^{11}B\{^1H\}$ NMR spectrum of **2-Fc** in C_6D_6.

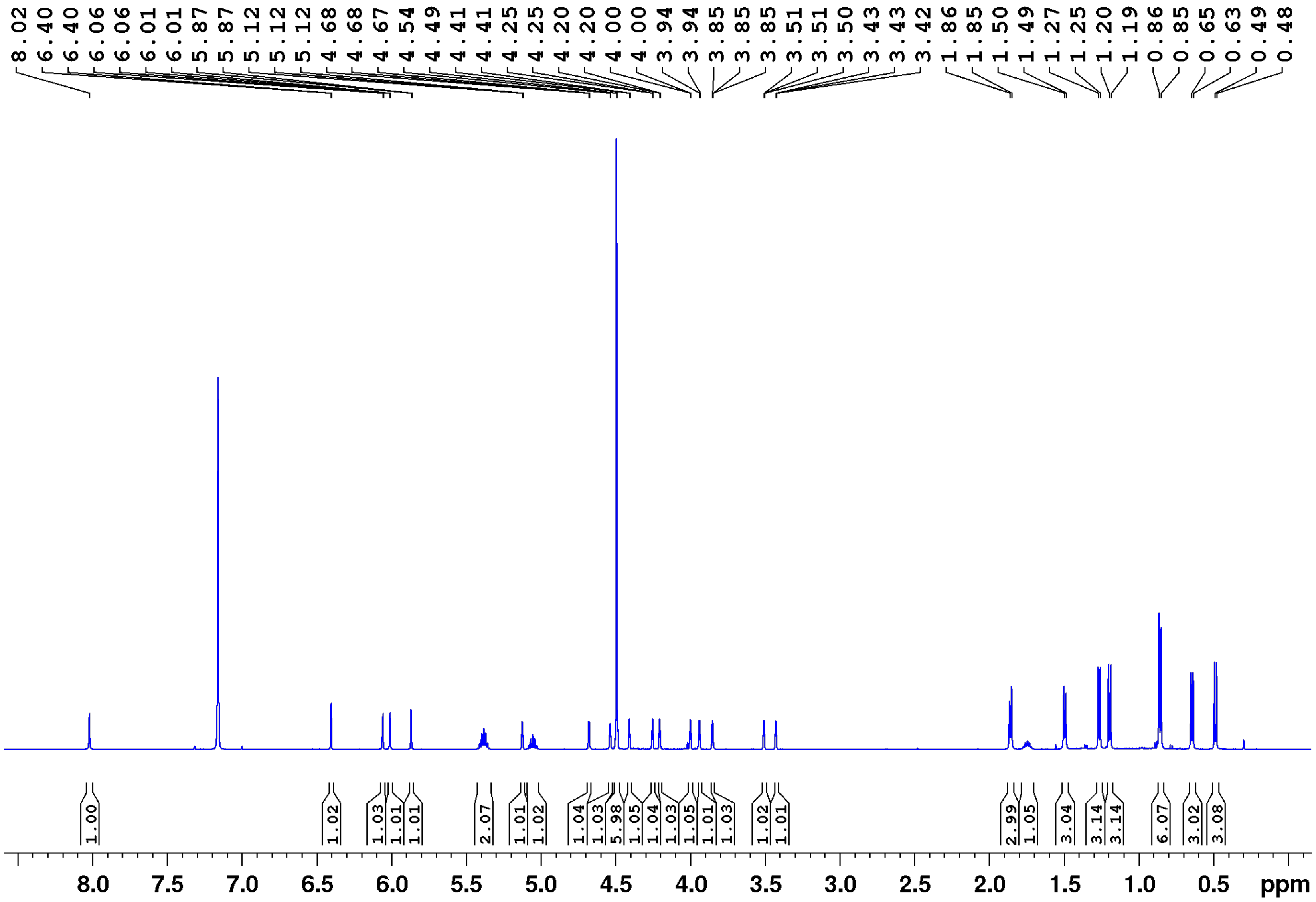

Figure S4. ^{1}H NMR spectrum of **5-Fc** in C_6D_6.

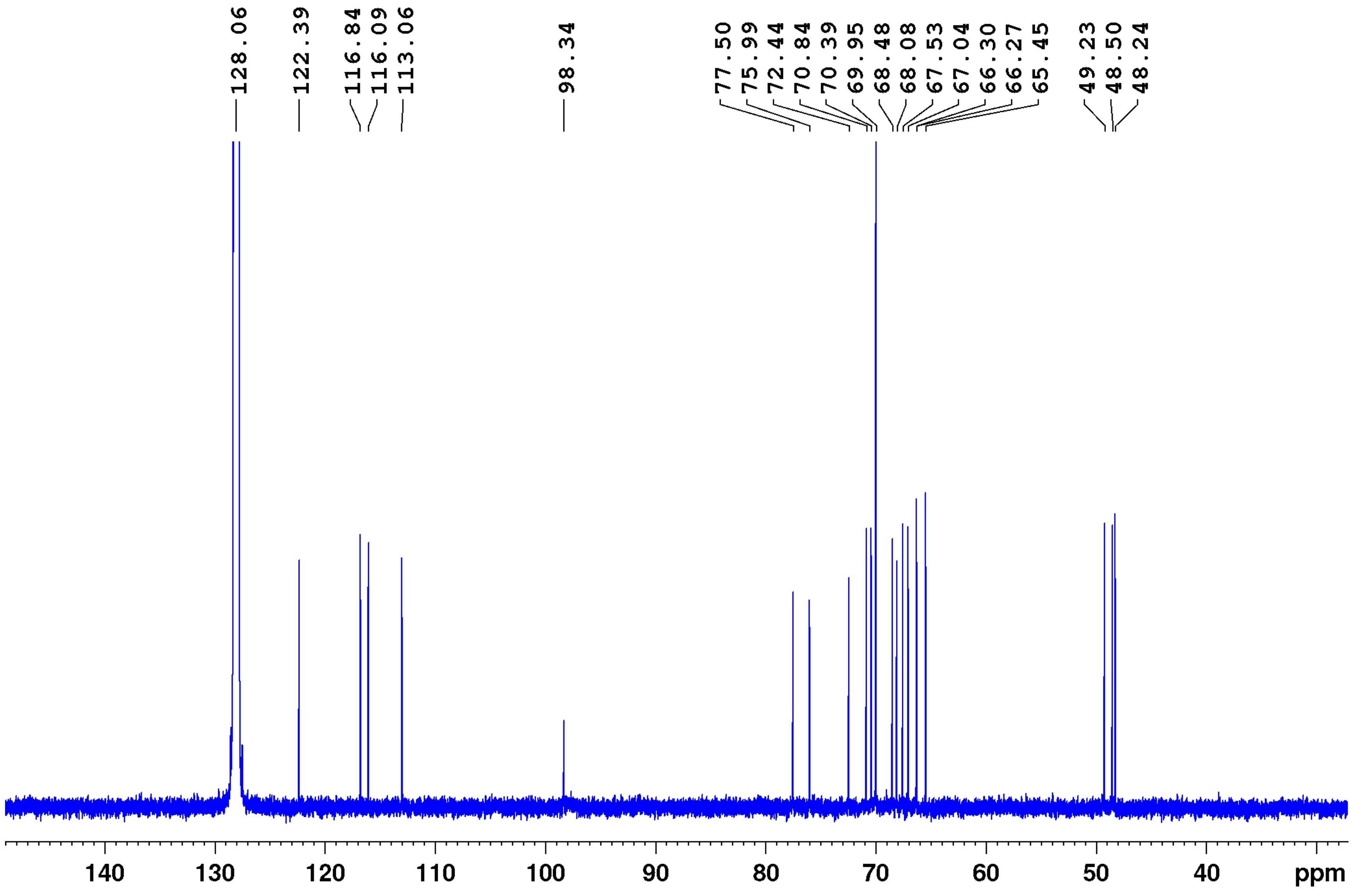

Figure S5. $^{13}C\{^{1}H\}$ NMR spectrum of **5-Fc** in C_6D_6.

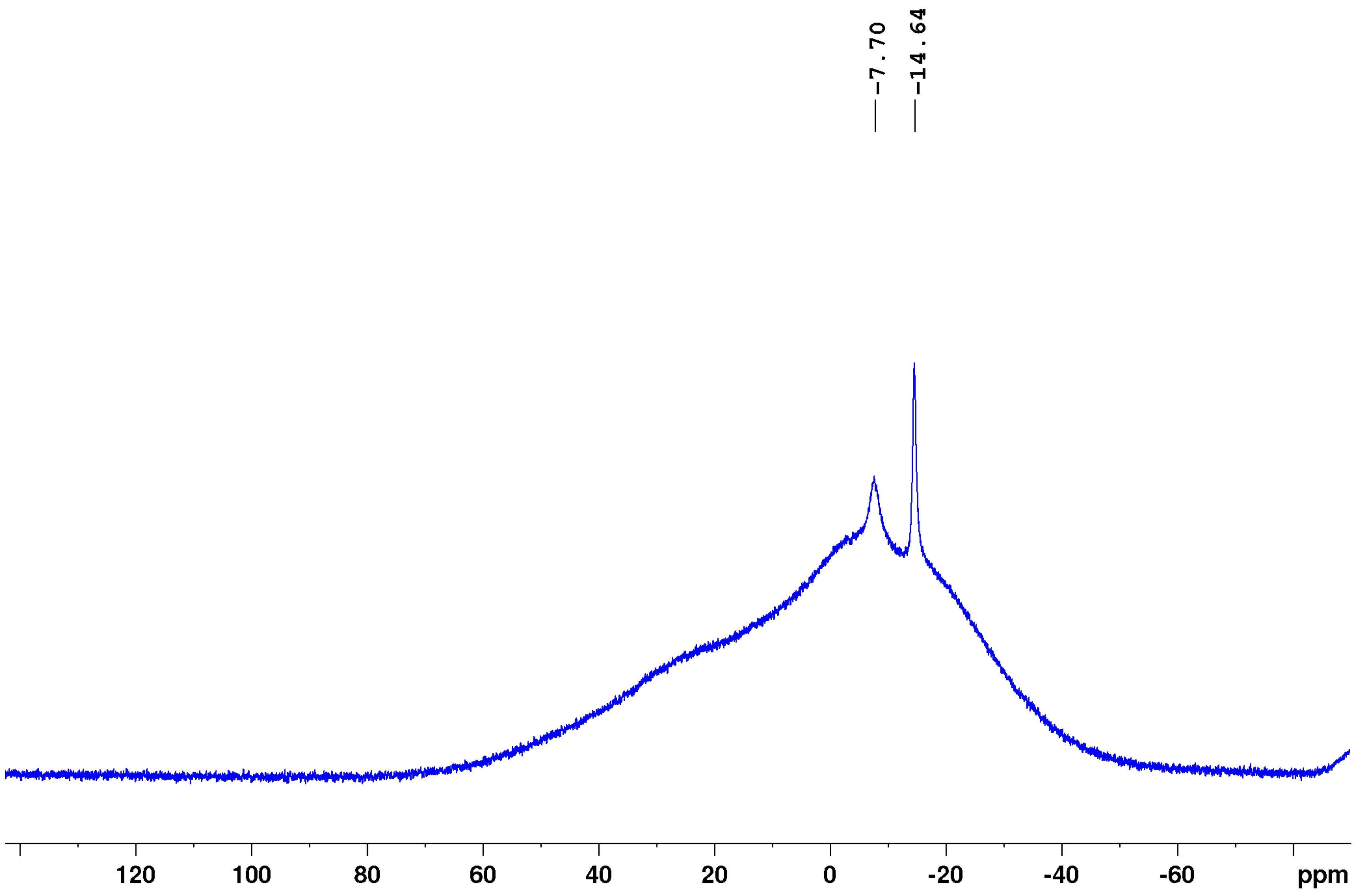

Figure S6. ^{11}B NMR spectrum of **5-Fc** in C_6D_6.

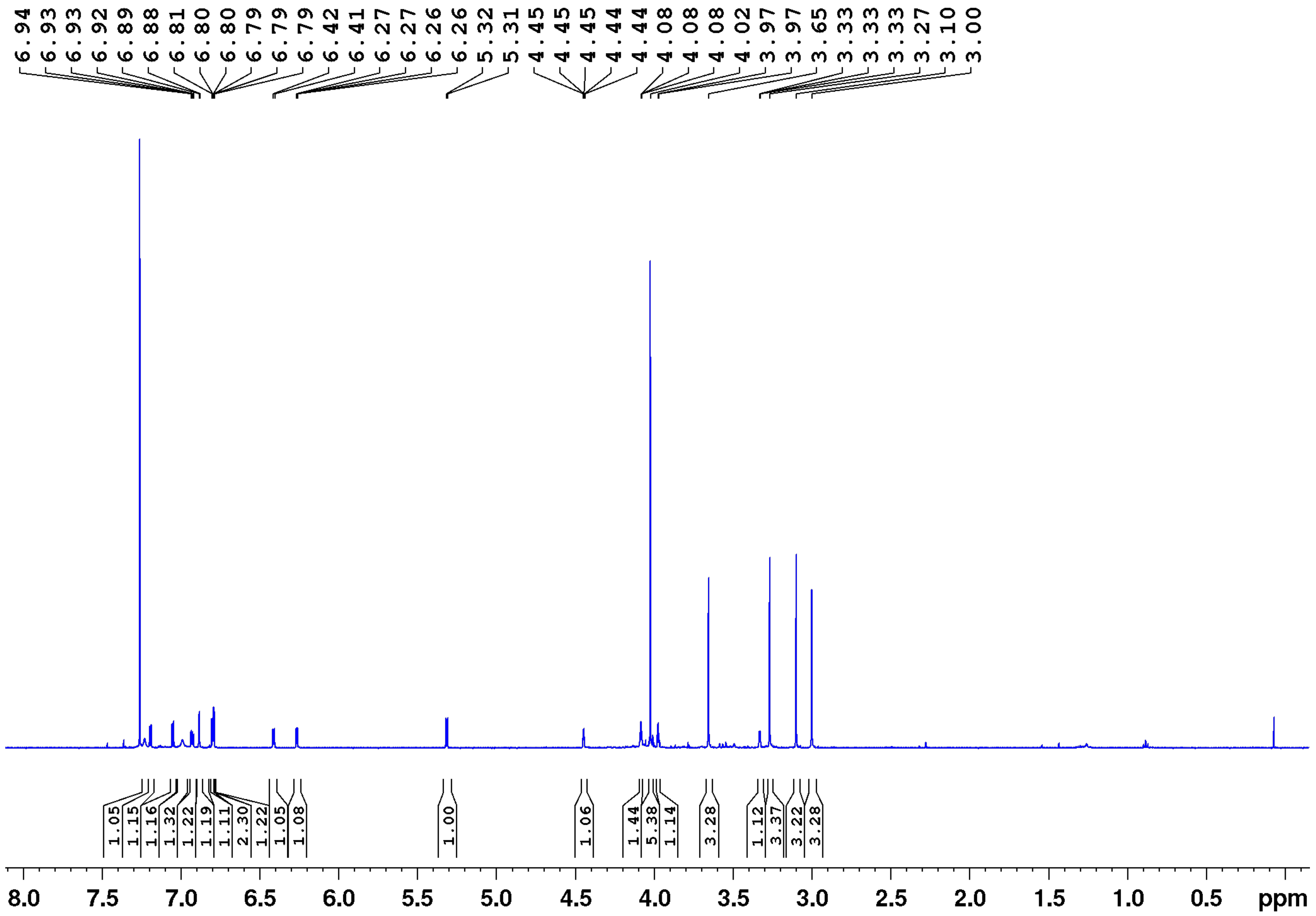

Figure S7. ^{1}H NMR spectrum of $\mathbf{7^{Tn}}$**-Fc** in $CDCl_3$.

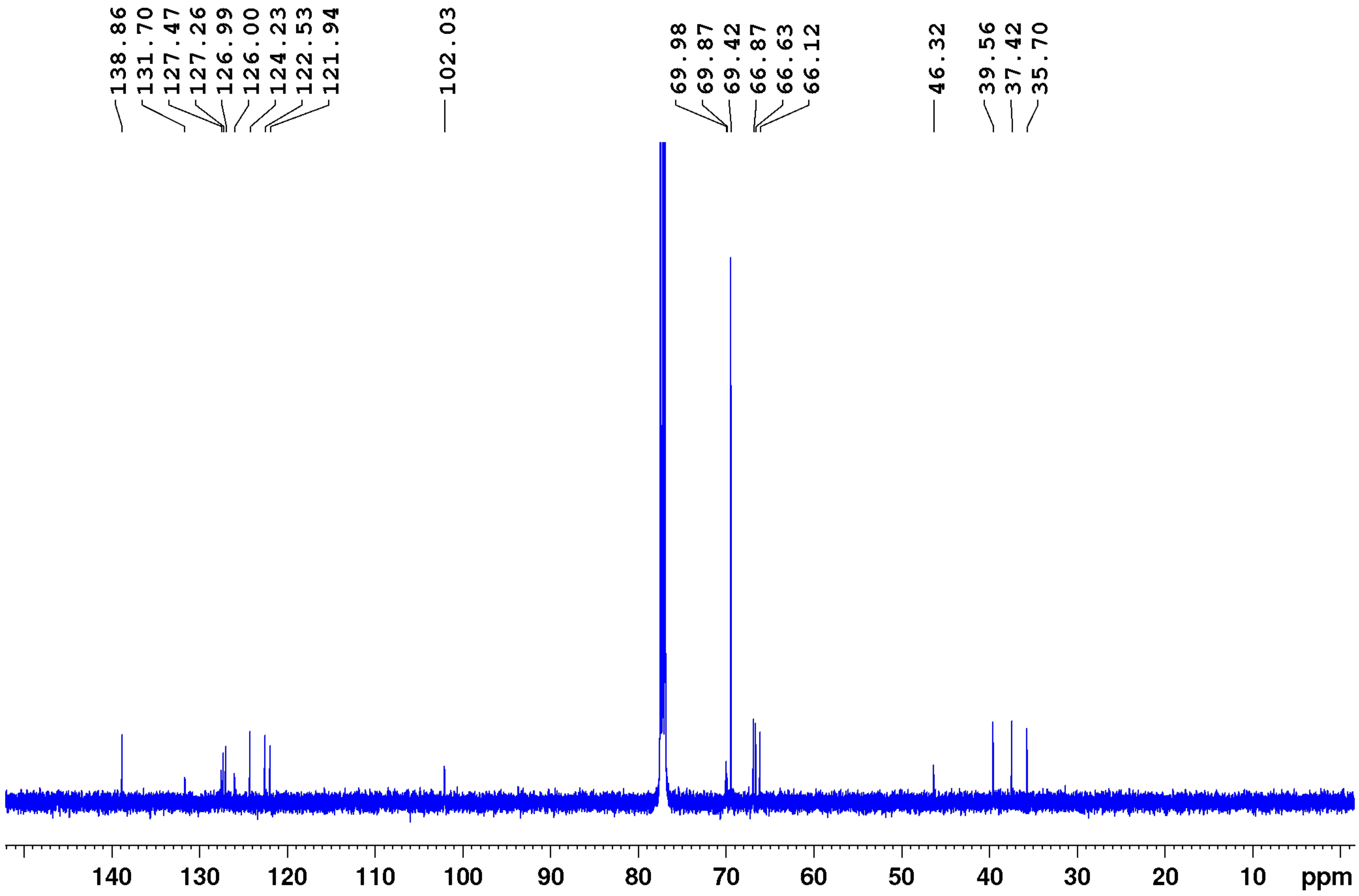

Figure S8. $^{13}C\{^1H\}$ NMR spectrum of $\mathbf{7^{Tn}}$**-Fc** in $CDCl_3$.

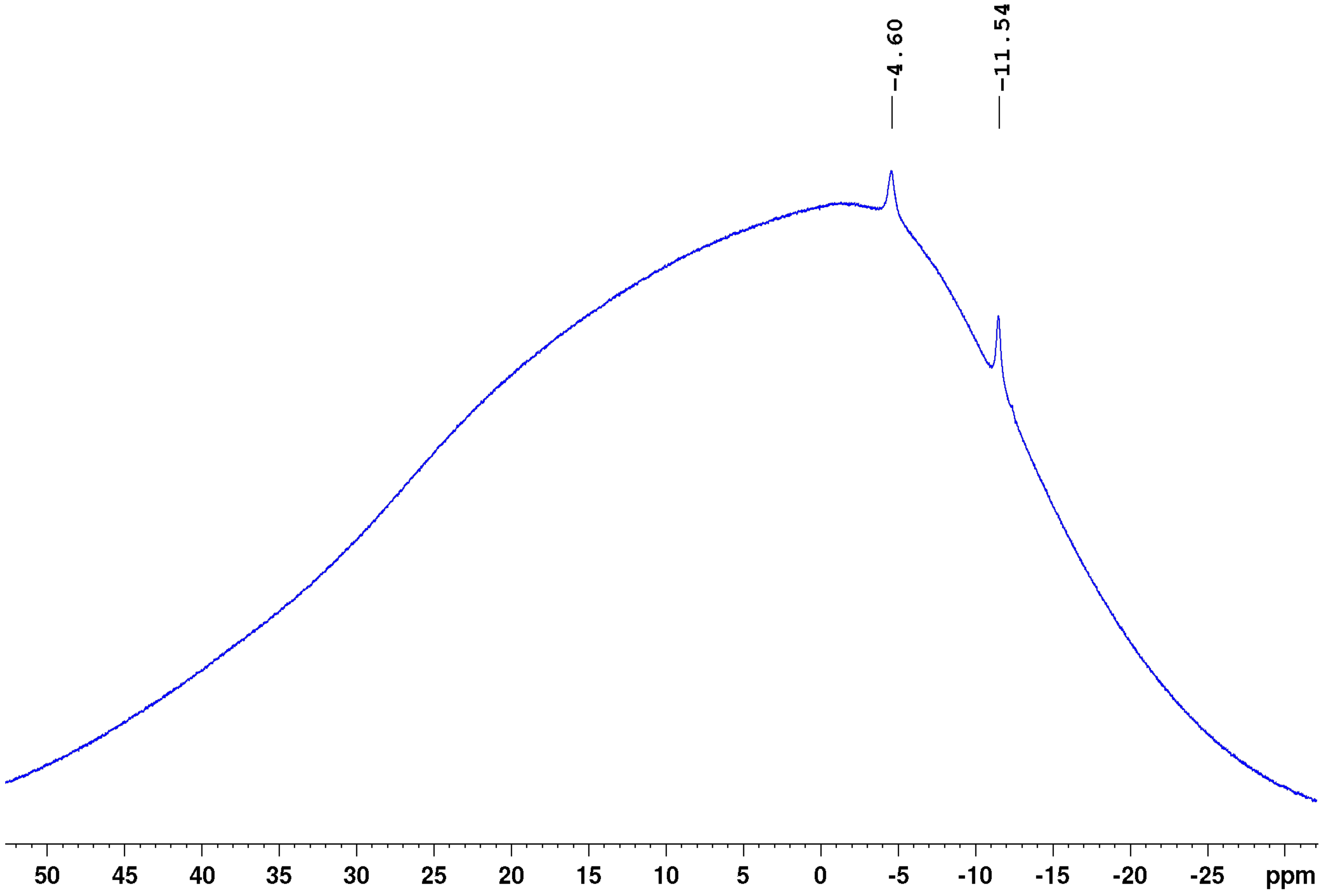

Figure S9. ^{11}B NMR spectrum of $\mathbf{7^{Tn}}$**-Fc** in $CDCl_3$.

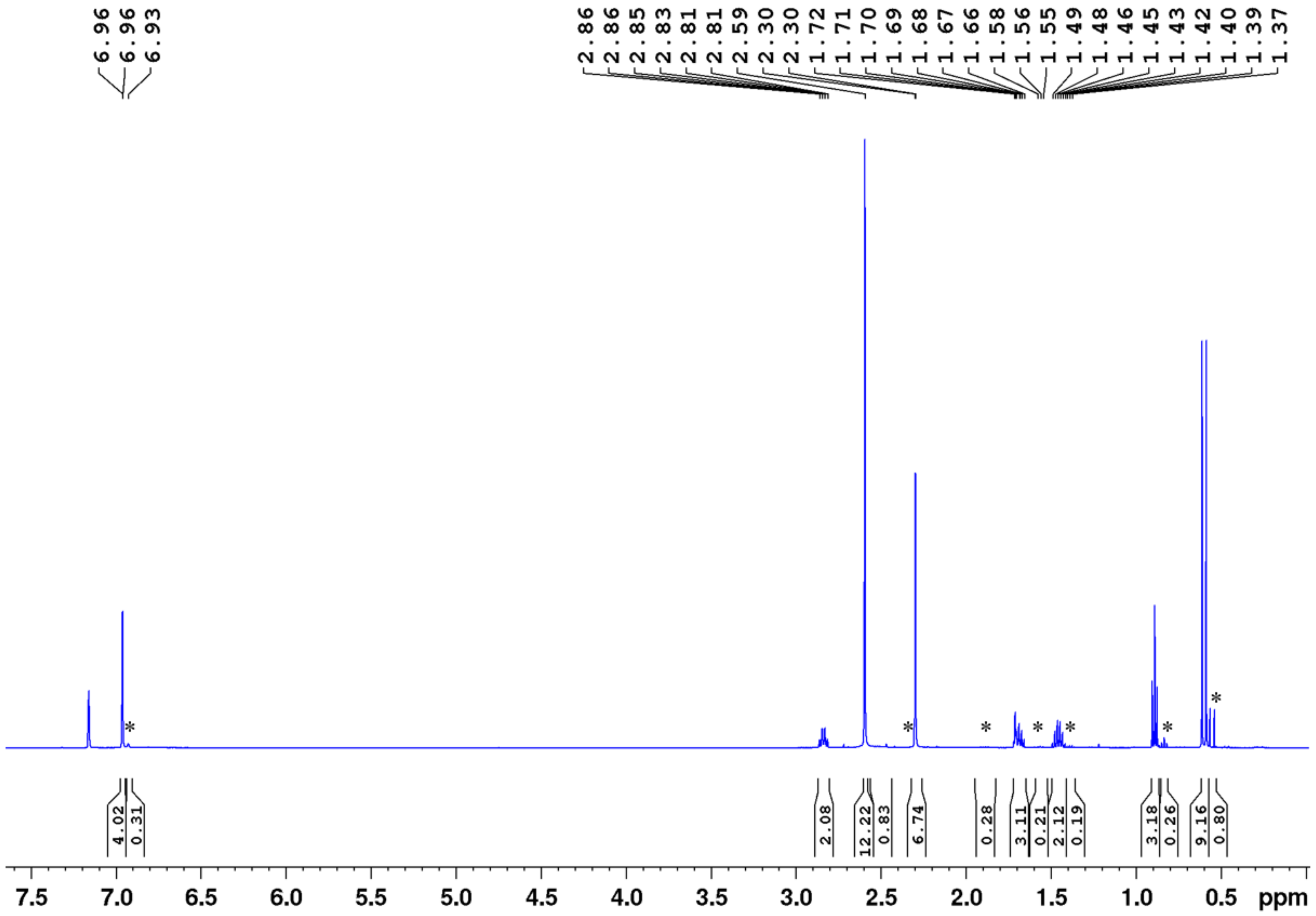

Figure S10. ^{1}H NMR spectrum of **8-Bu** in C_6D_6. The minor isomer **D** is marked with *.

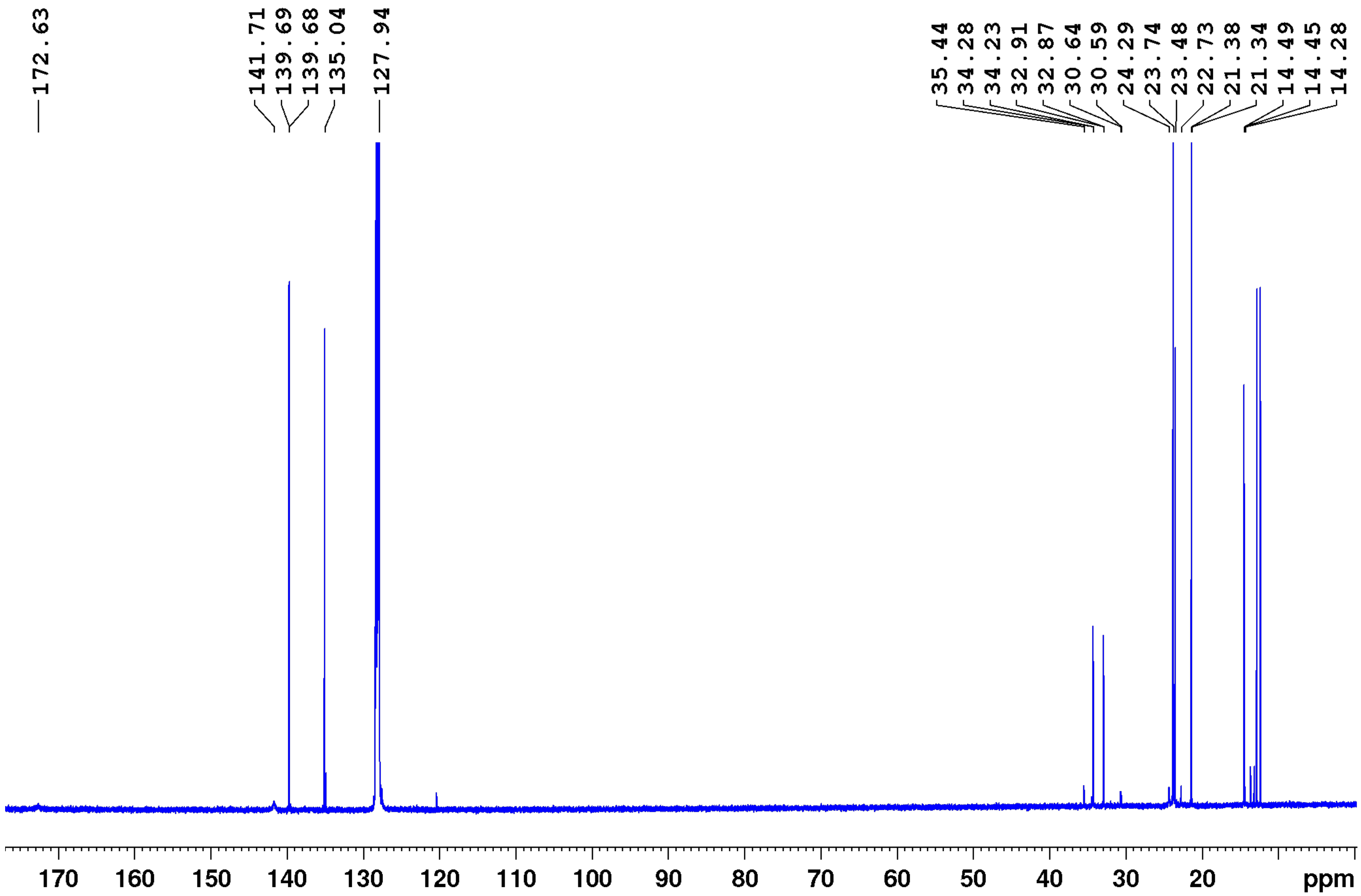

Figure S11. $^{13}C\{^{1}H\}$ NMR spectrum of **8-Bu** in C_6D_6. Minor resonances belong to the minor isomer **D**.

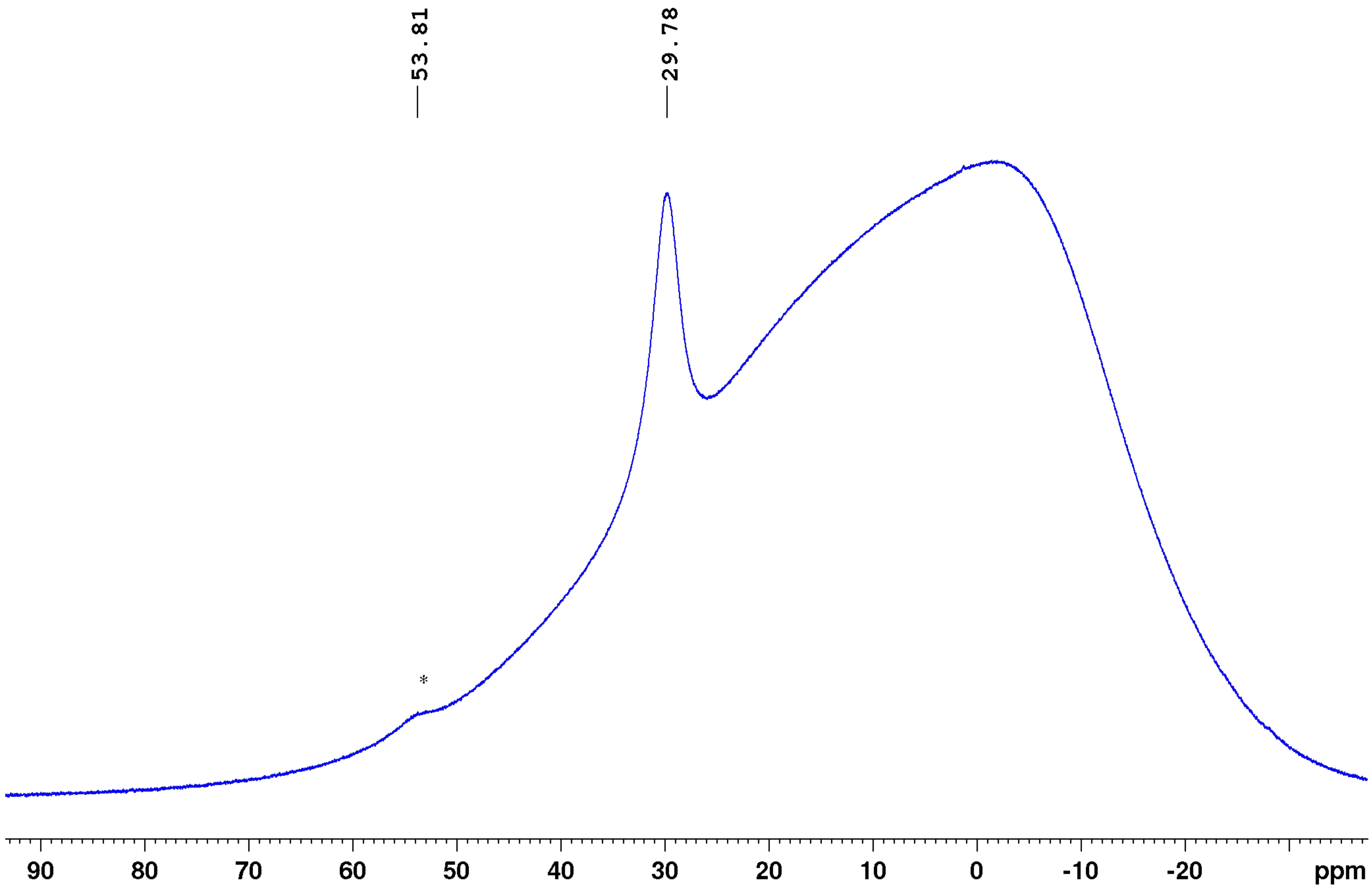

Figure S12. ^{11}B NMR spectrum of **8-Bu** in C_6D_6. The minor isomer **D** is marked with *.

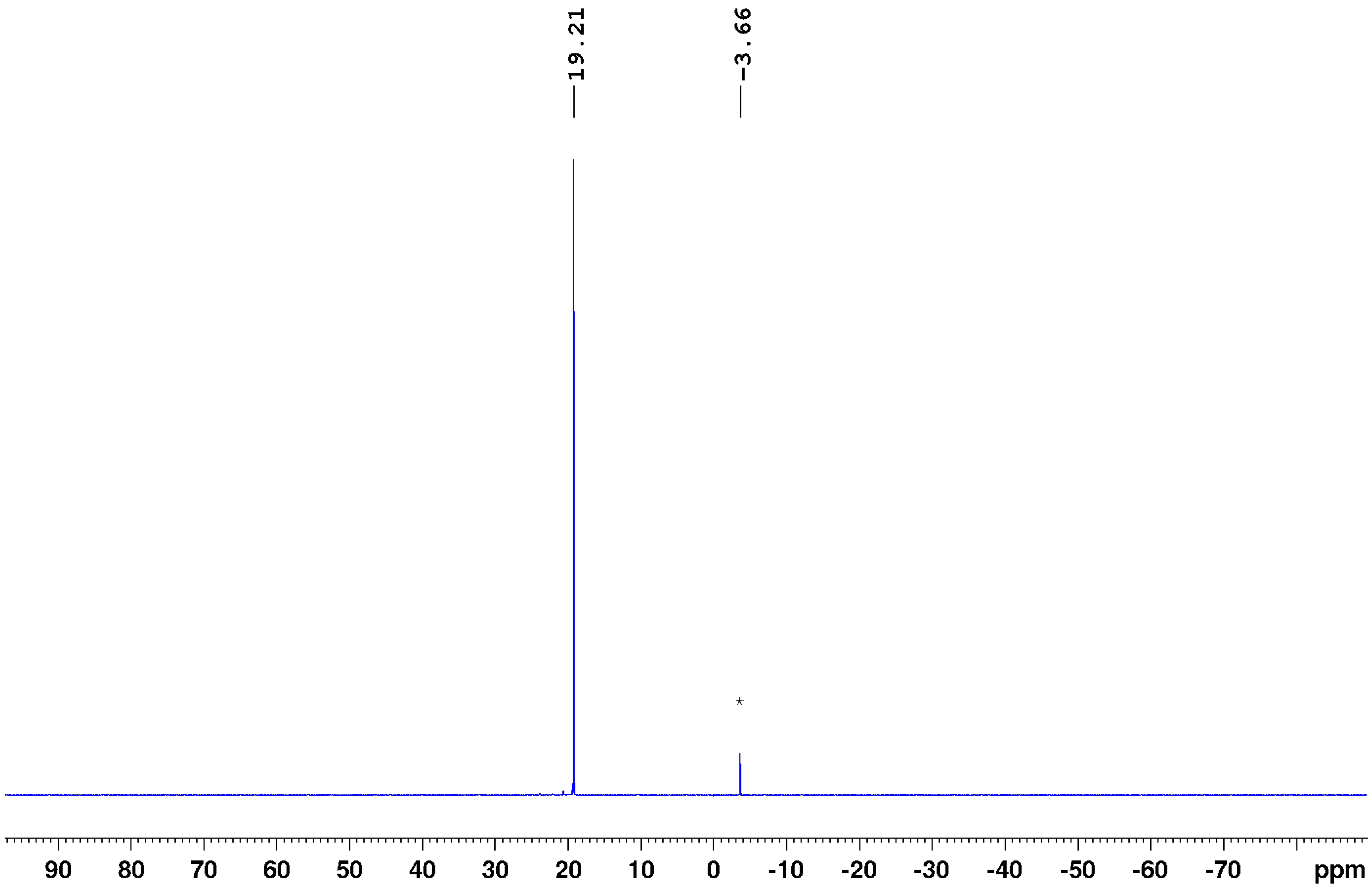

Figure S13. $^{31}P\{^1H\}$ NMR spectrum of **8-Bu** in C_6D_6. The minor isomer **D** is marked with *.

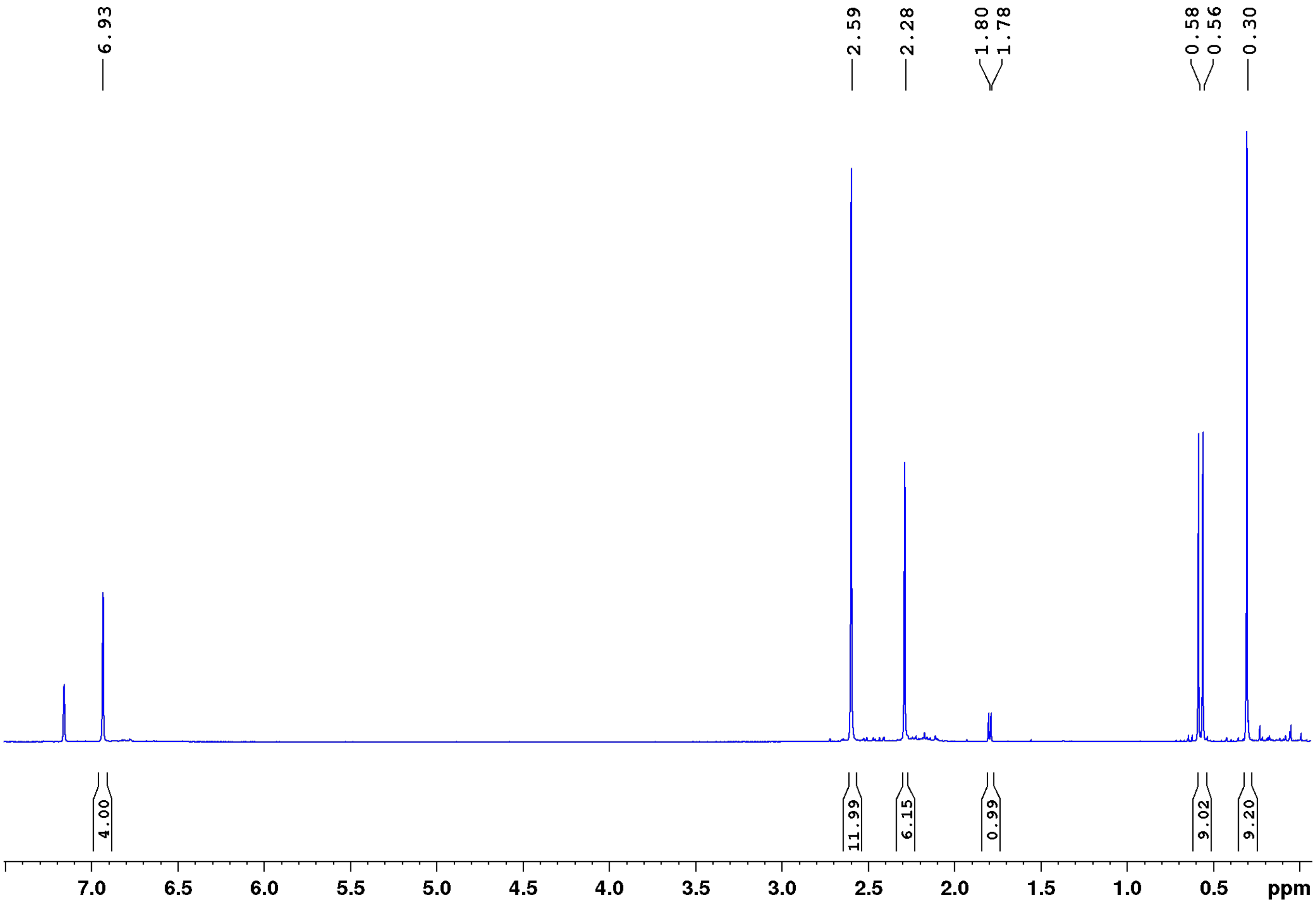

Figure S14. ^{1}H NMR spectrum of **8-TMS** in C_6D_6.

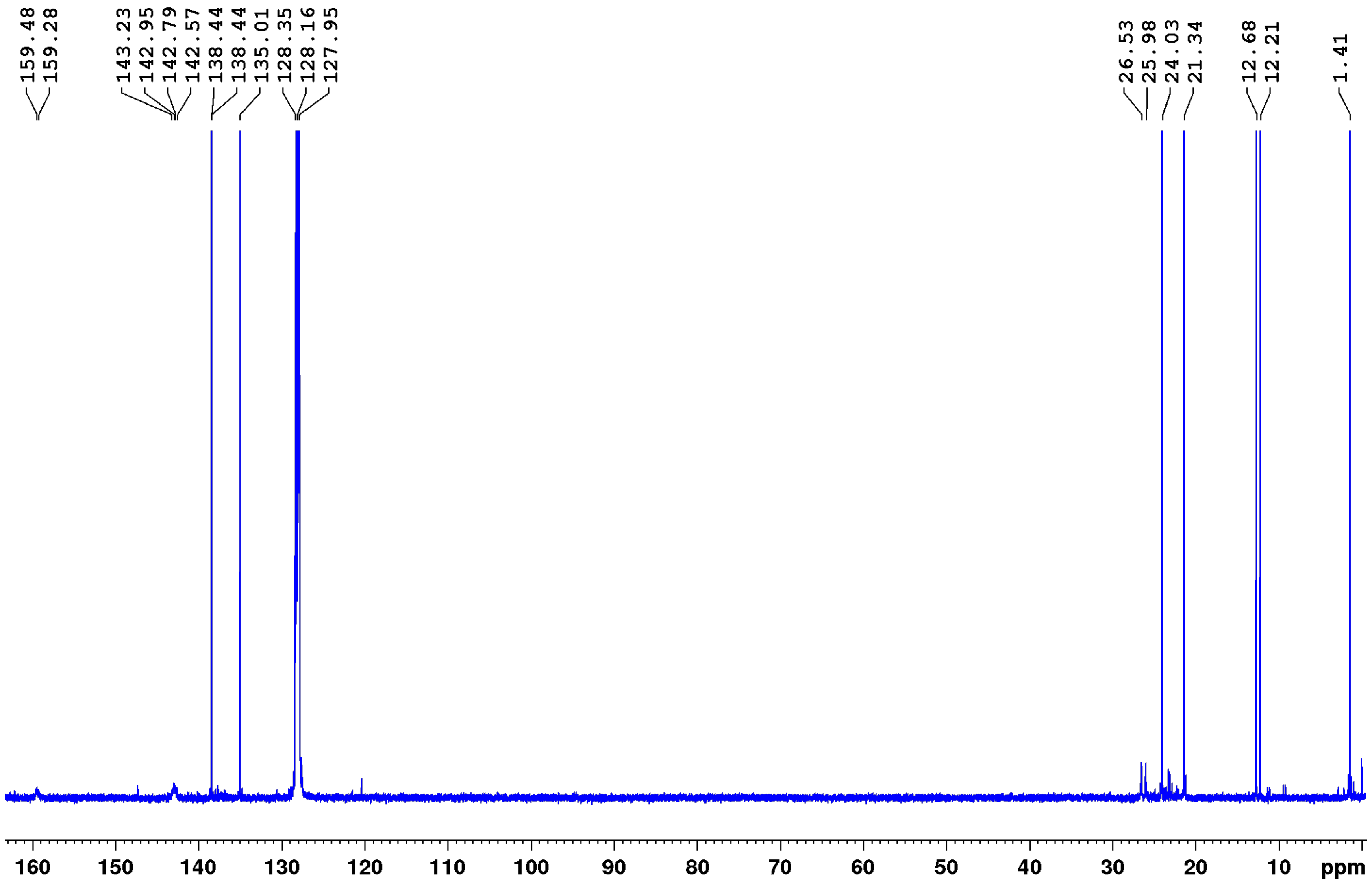

Figure S15. $^{13}C\{^{1}H\}$ NMR spectrum of **8-TMS** in C_6D_6.

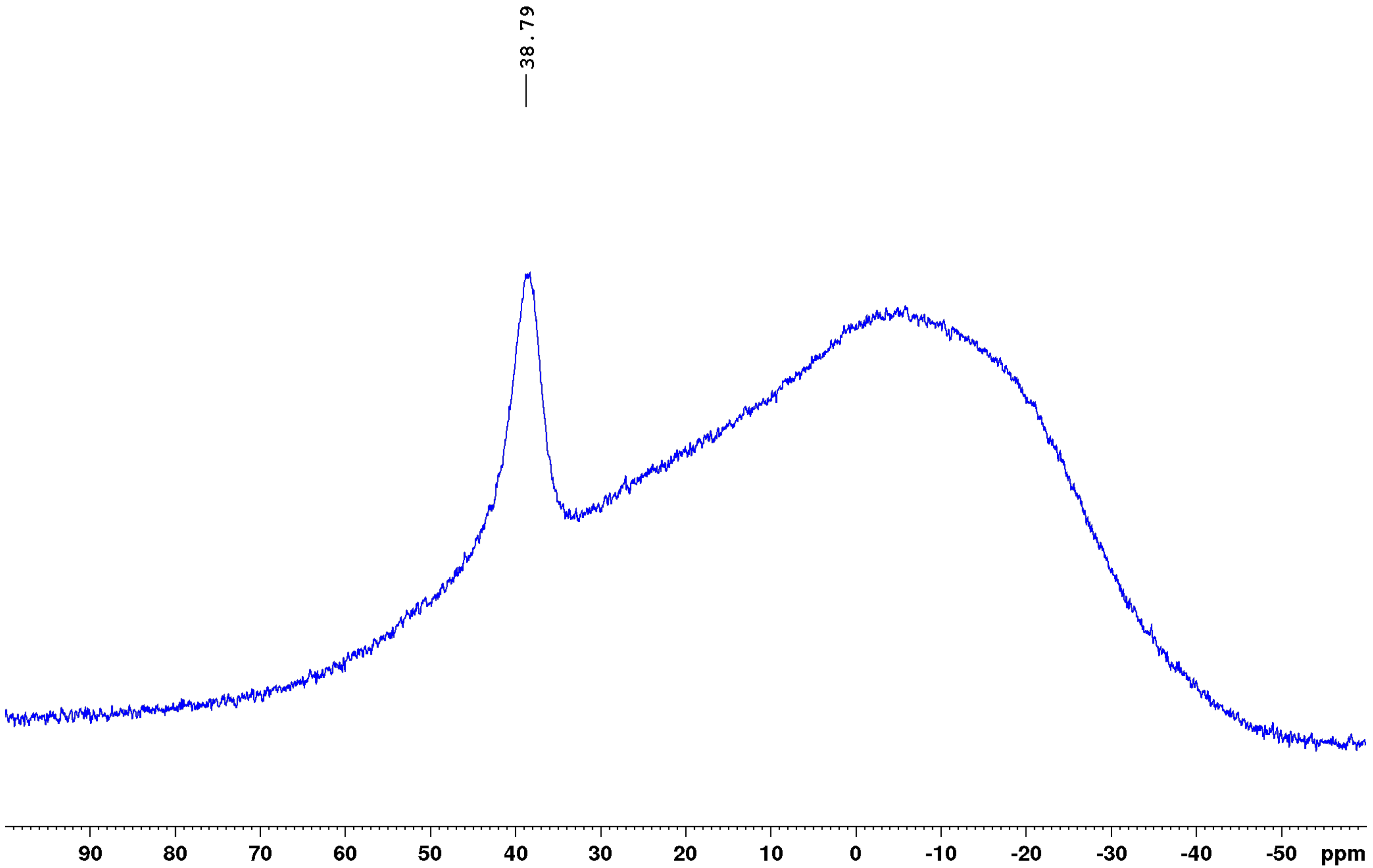

Figure S16. ^{11}B NMR spectrum of **8-TMS** in C_6D_6.

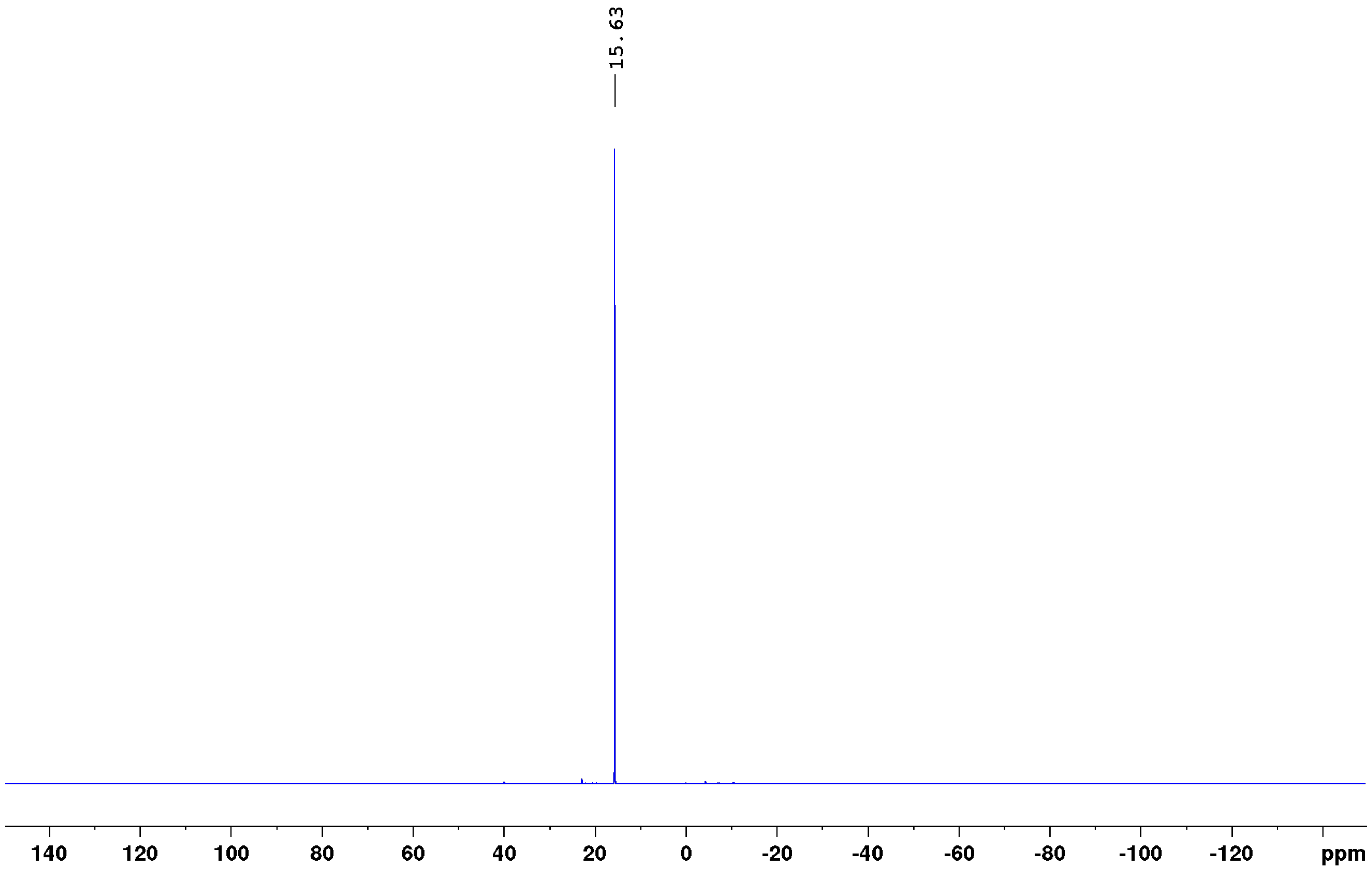

Figure S17. $^{31}P\{^{1}H\}$ NMR spectrum of **8-TMS** in C_6D_6.

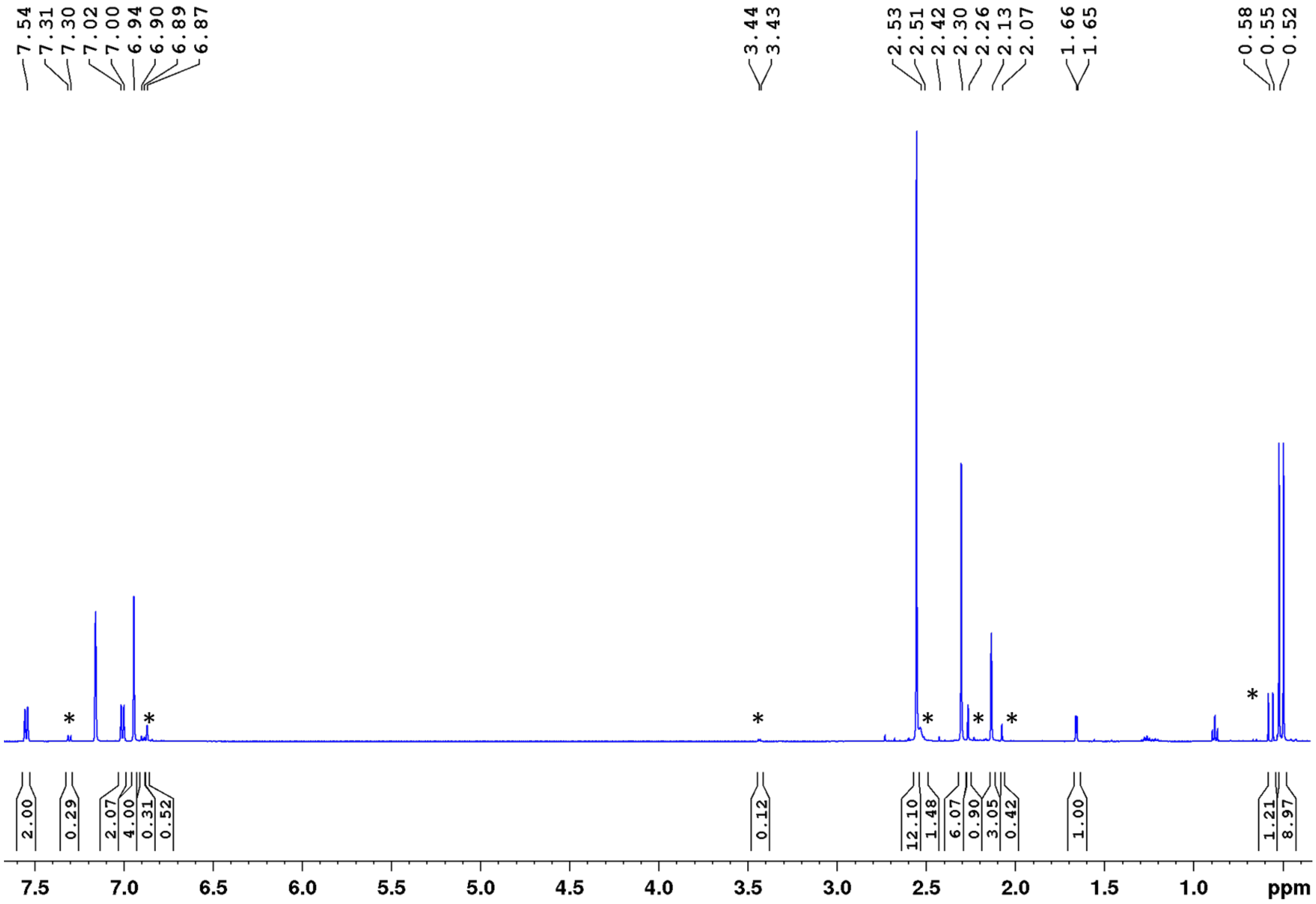

Figure S18. ^{1}H NMR spectrum of **8-Tol** in C_6D_6. The minor isomer **D** is marked with *.

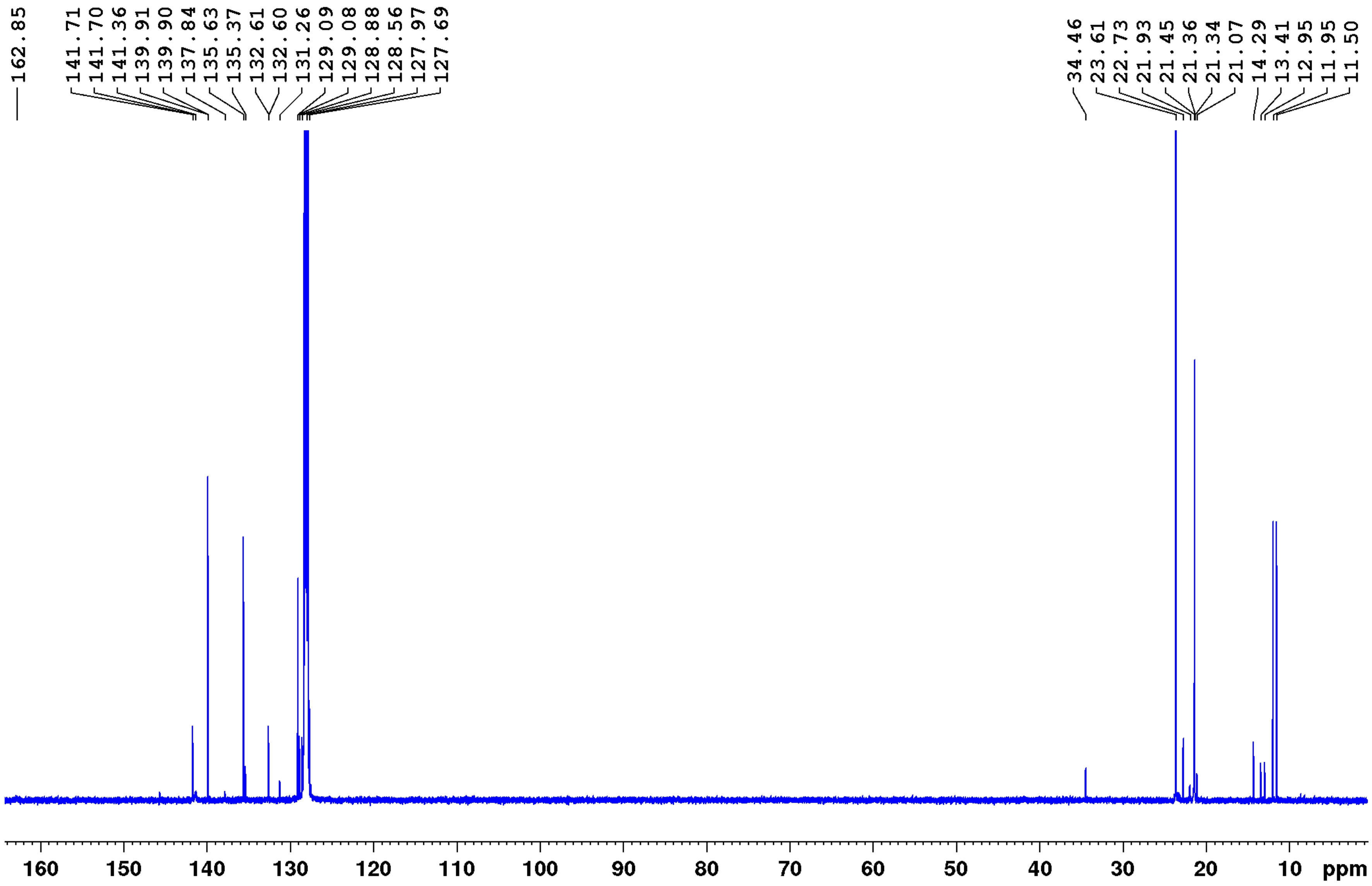

Figure S19. $^{13}C\{^{1}H\}$ NMR spectrum of **8-Tol** in C_6D_6.

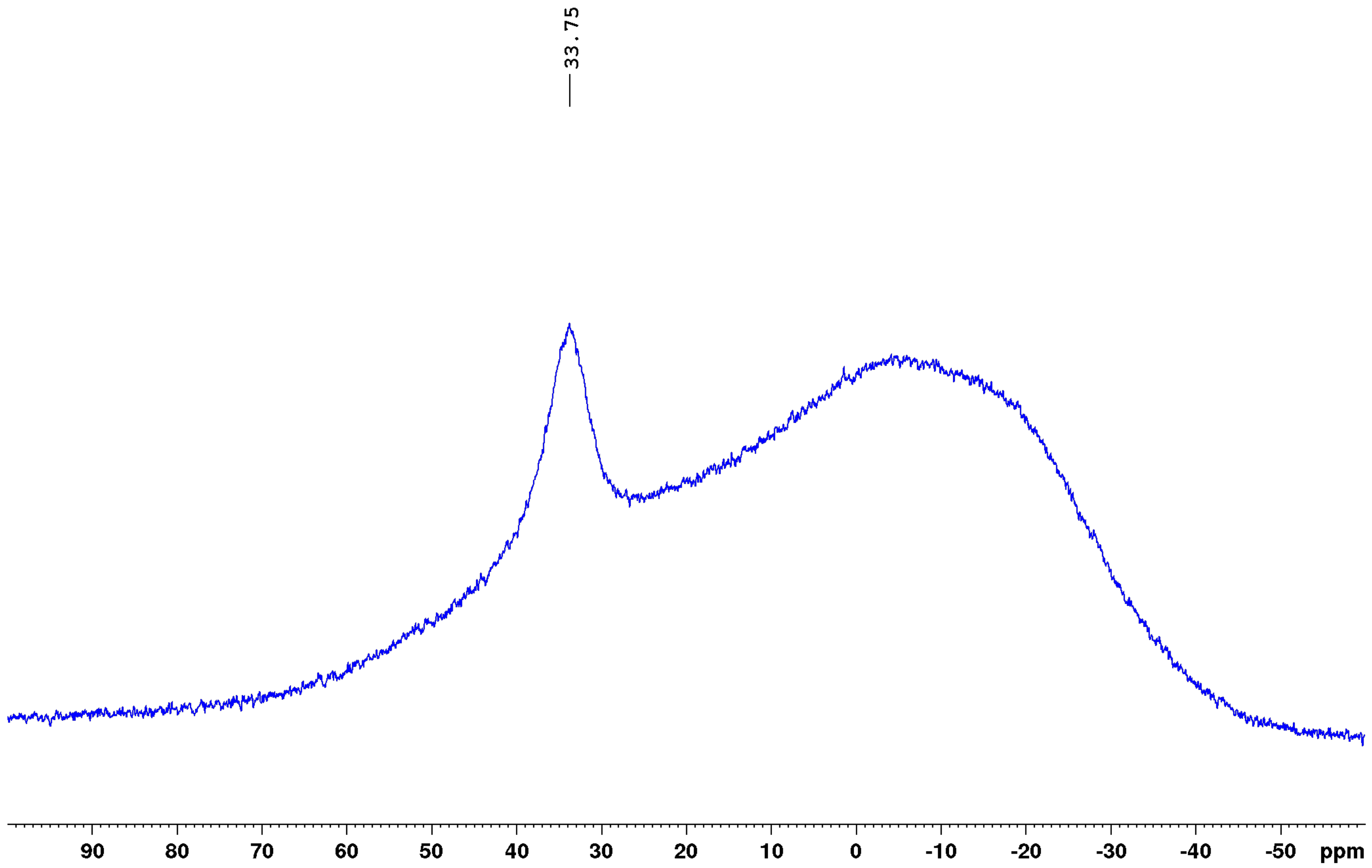

Figure S20. ^{11}B NMR spectrum of **8-Tol** in C_6D_6.

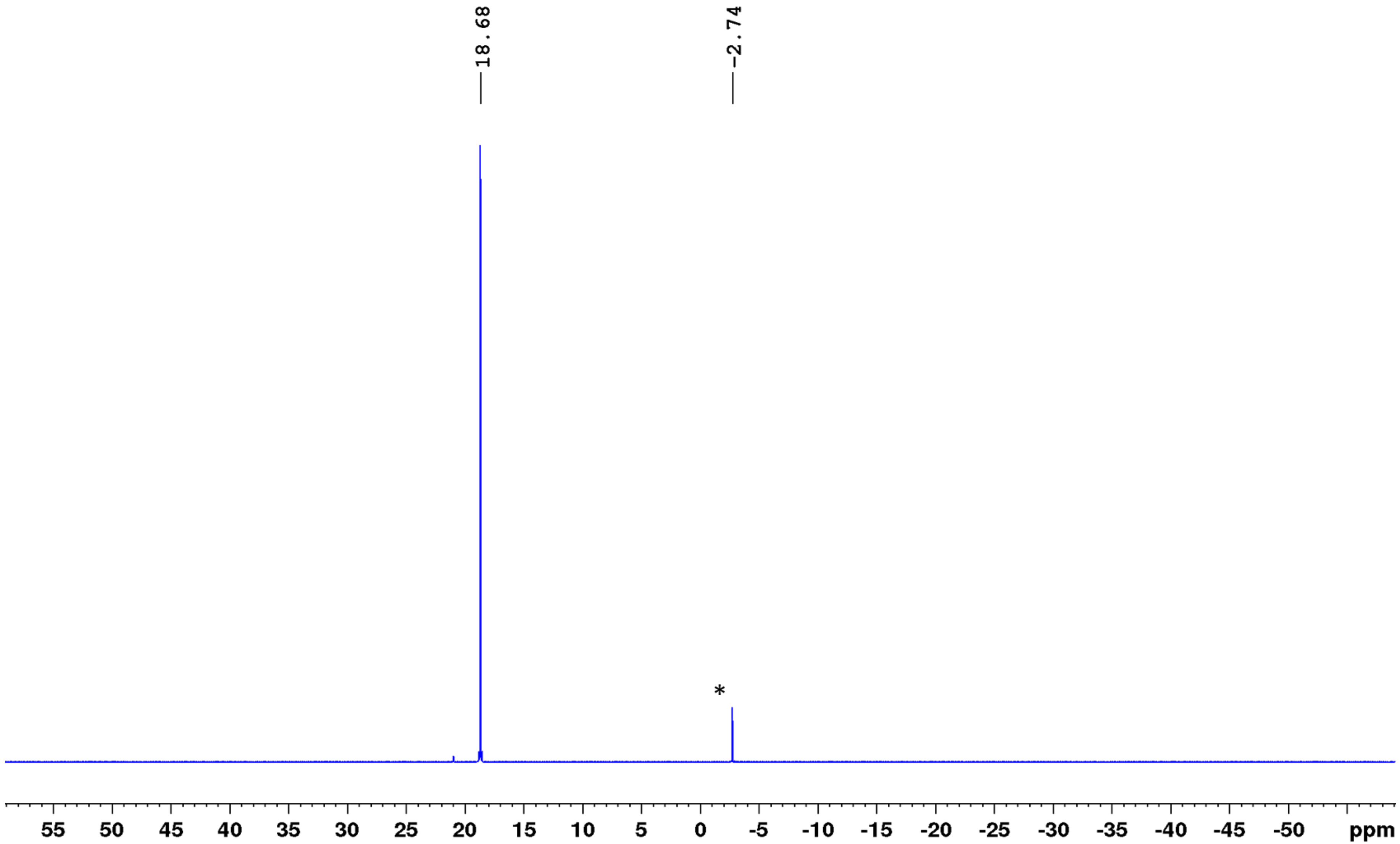

Figure S21. $^{31}P\{^{1}H\}$ NMR spectrum of **8-Tol** in C_6D_6. The minor isomer **D** is marked with *.

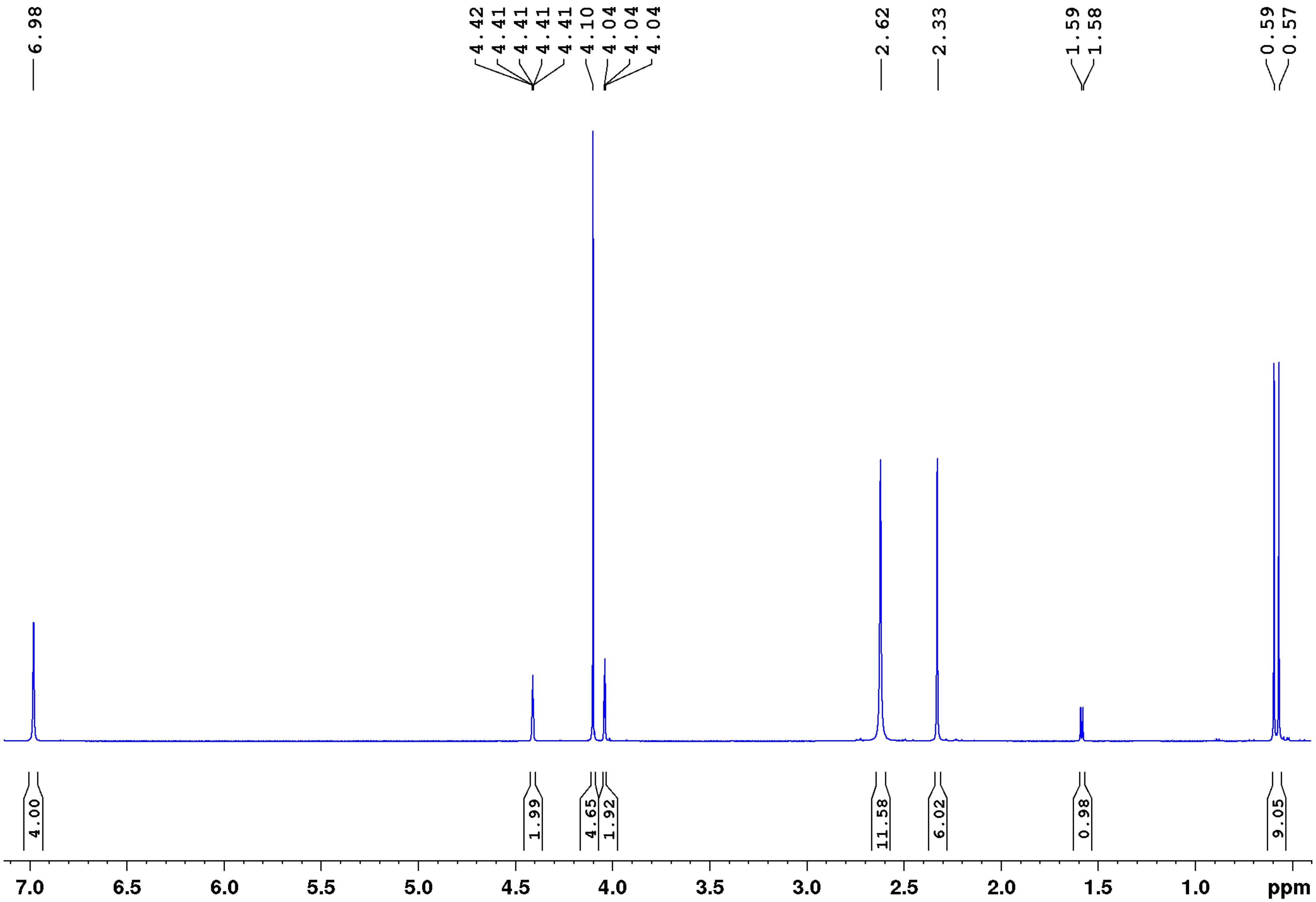

Figure S22. ^{1}H NMR spectrum of **8-Fc** in C_6D_6.

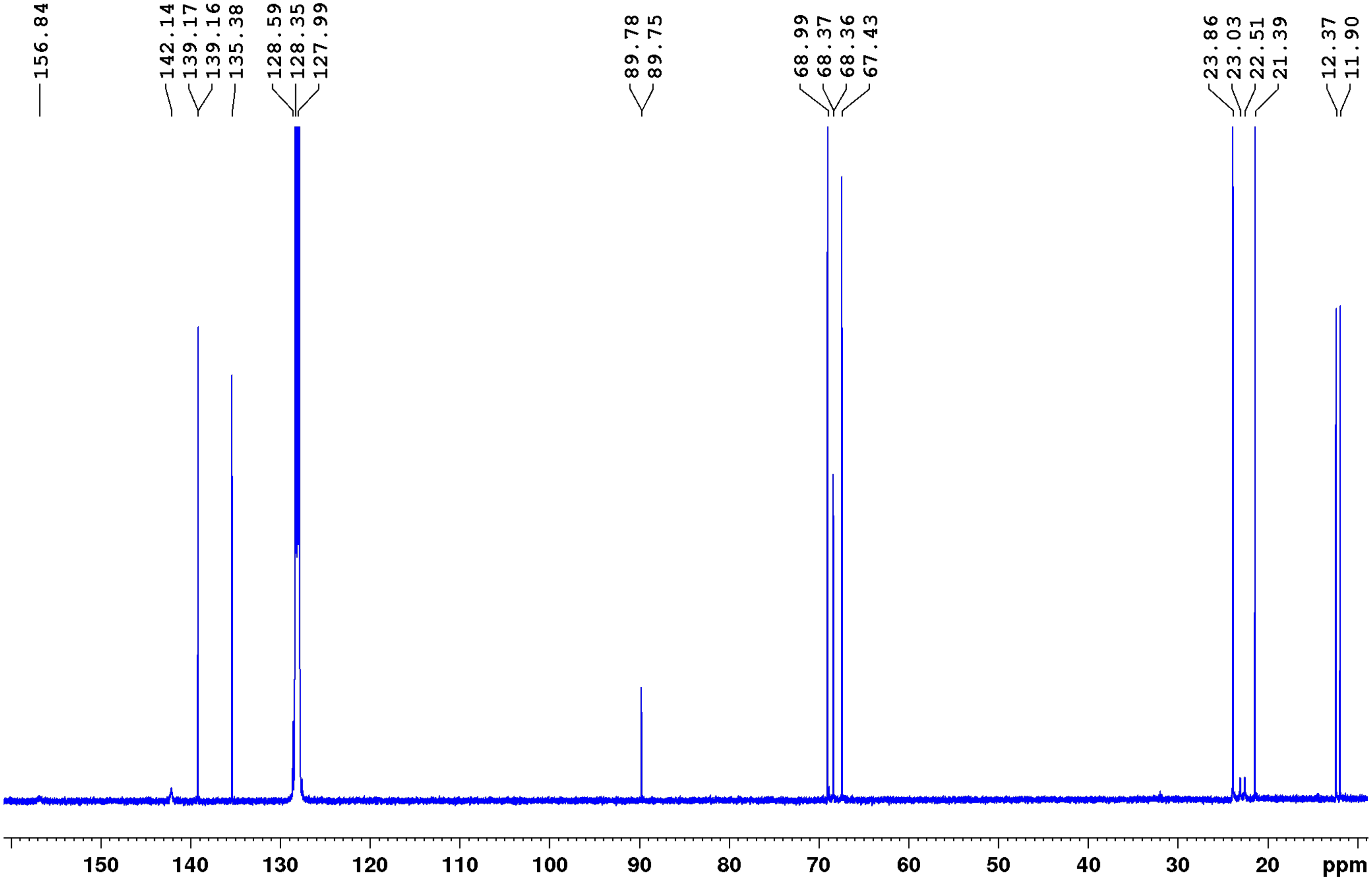

Figure S23. ${}^{13}C\{{}^{1}H\}$ NMR spectrum of **8-Fc** in C_6D_6.

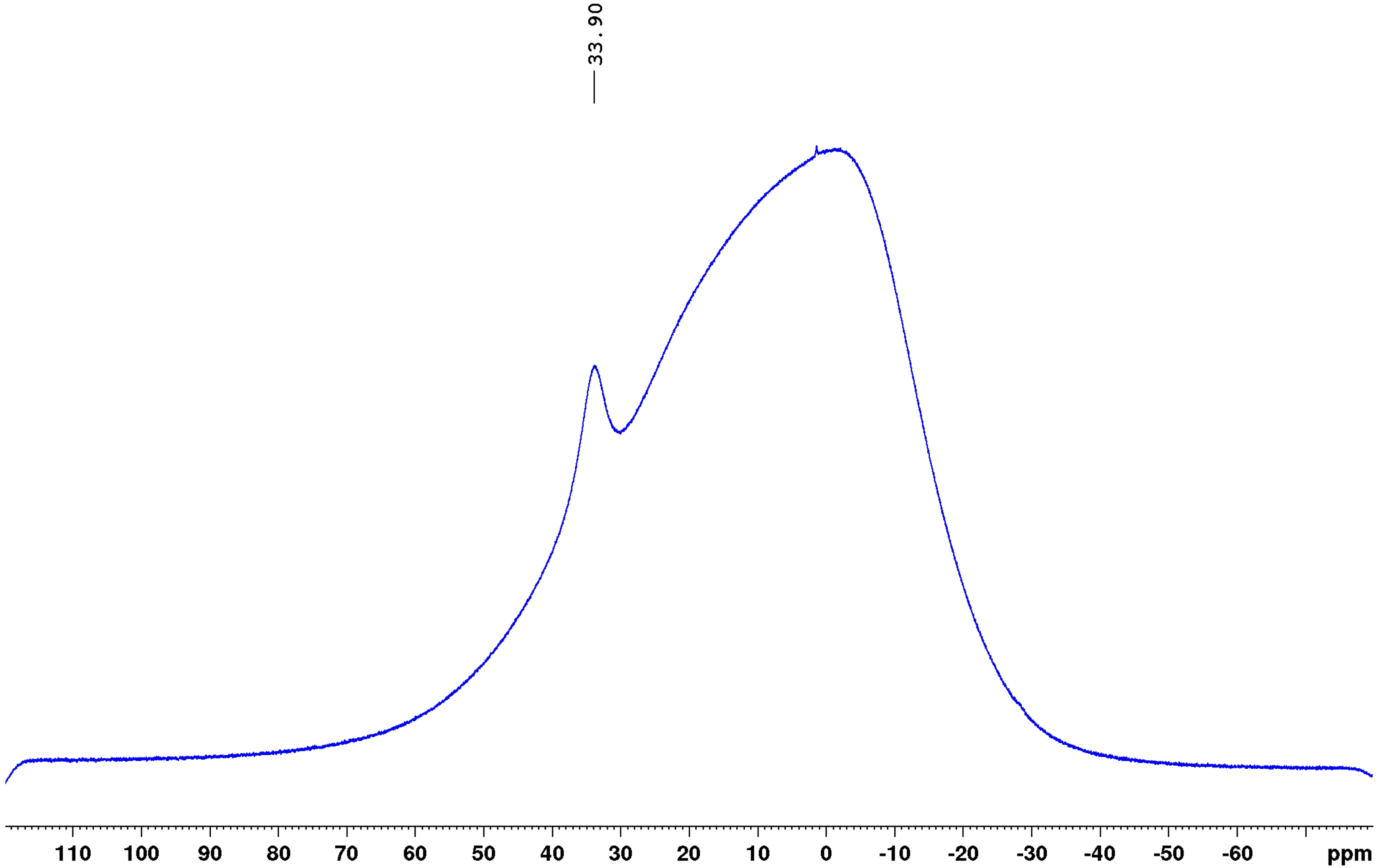

Figure S24. ^{11}B NMR spectrum of **8-Fc** in C_6D_6.

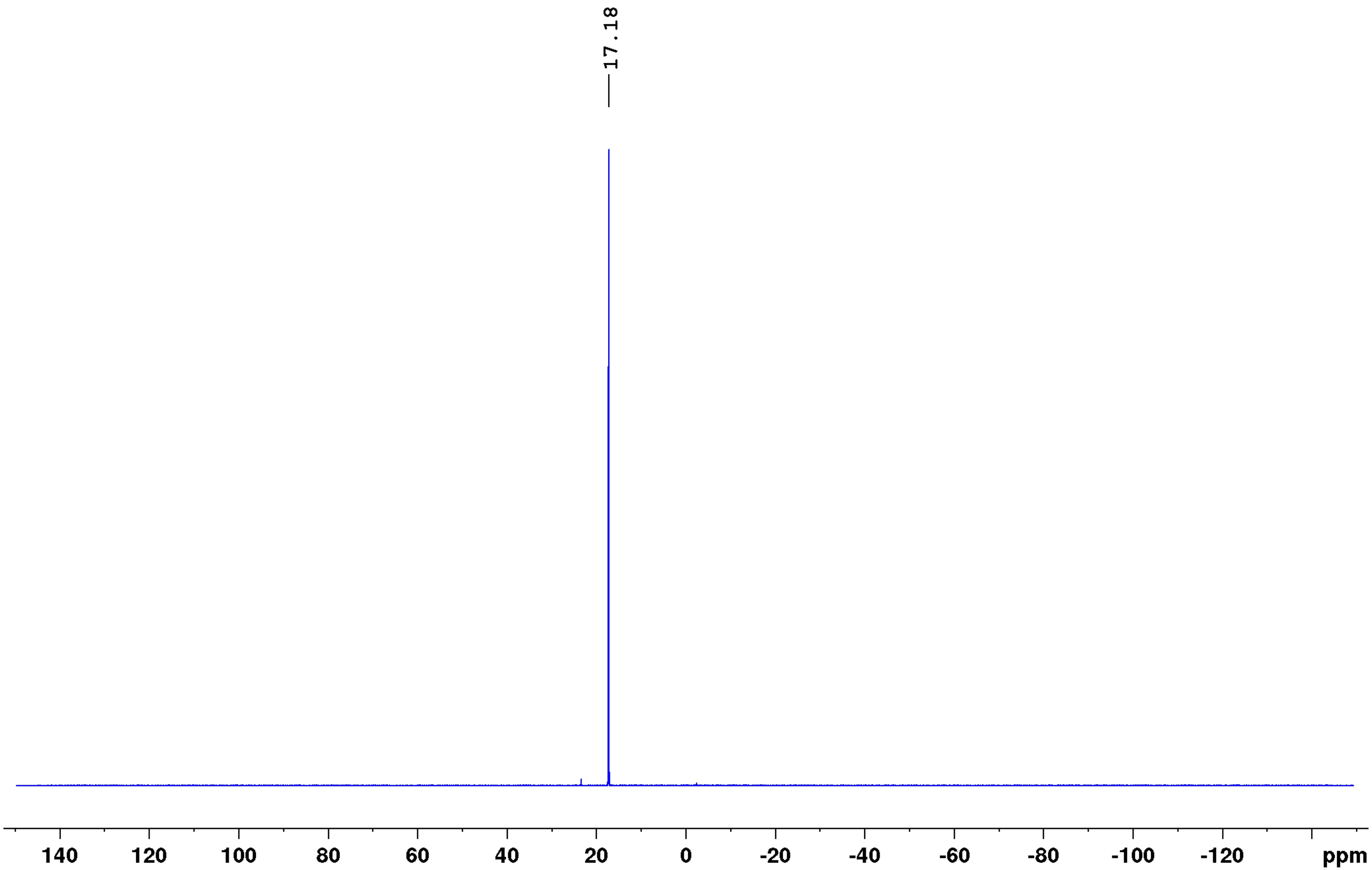

Figure S25. $^{31}P\{^{1}H\}$ NMR spectrum of **8-Fc** in C_6D_6.

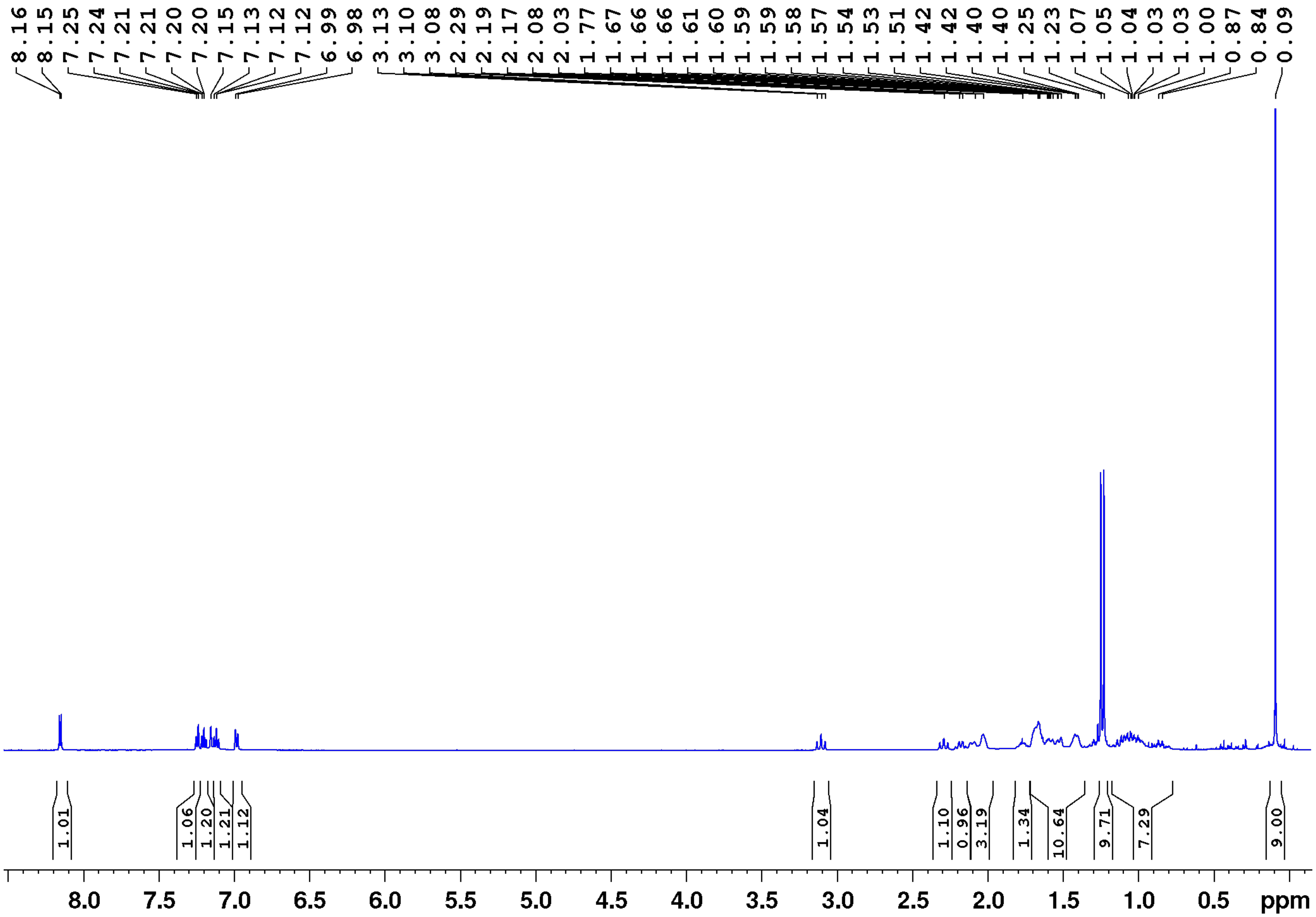

Figure S26. ^{1}H NMR spectrum of **11-TMS** in C_6D_6.

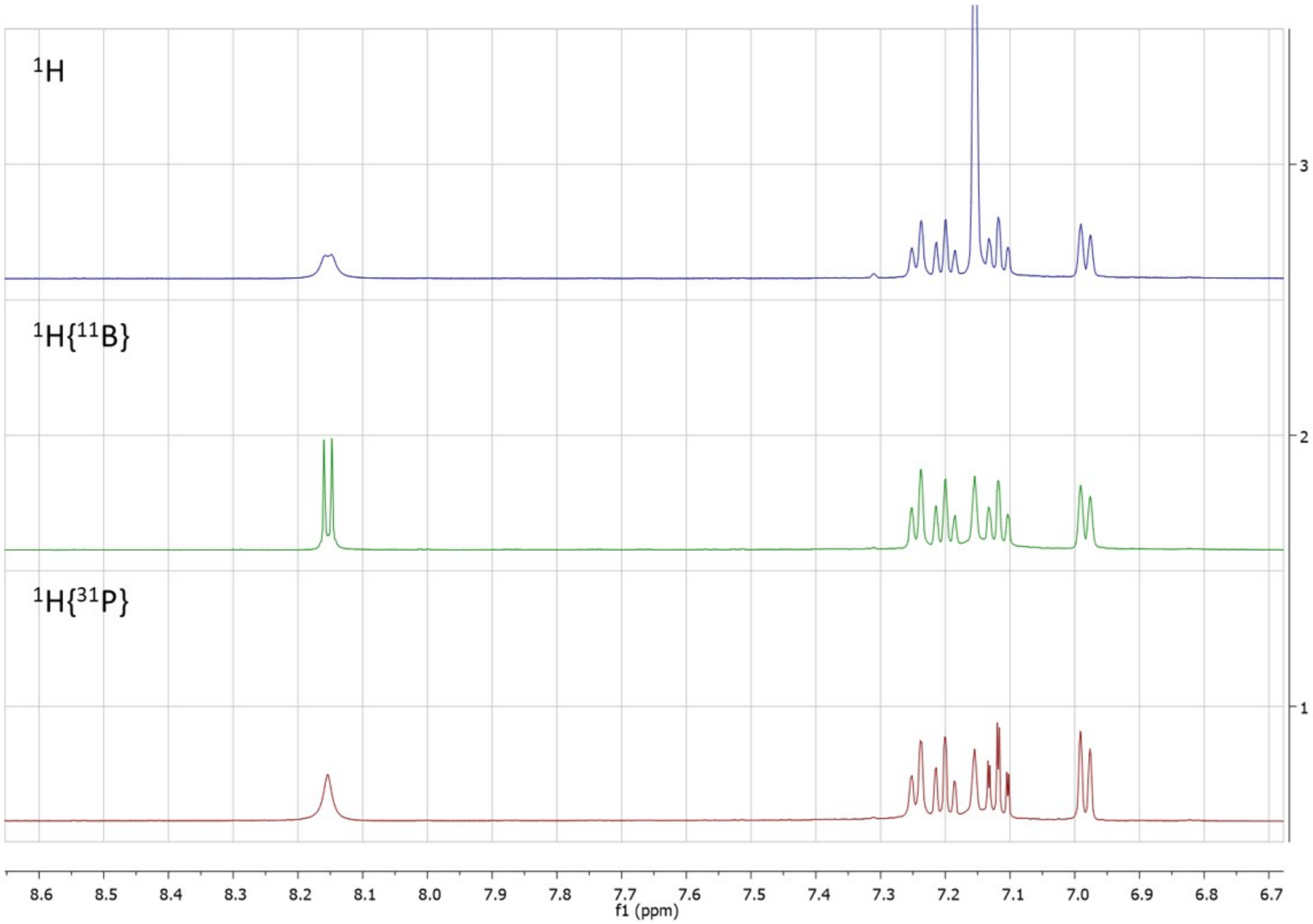

Figure S27. ^{1}H, ^{1}H{^{11}B} and ^{1}H{^{31}P} NMR spectra of **11-TMS** in C_6D_6 in the aromatic region.

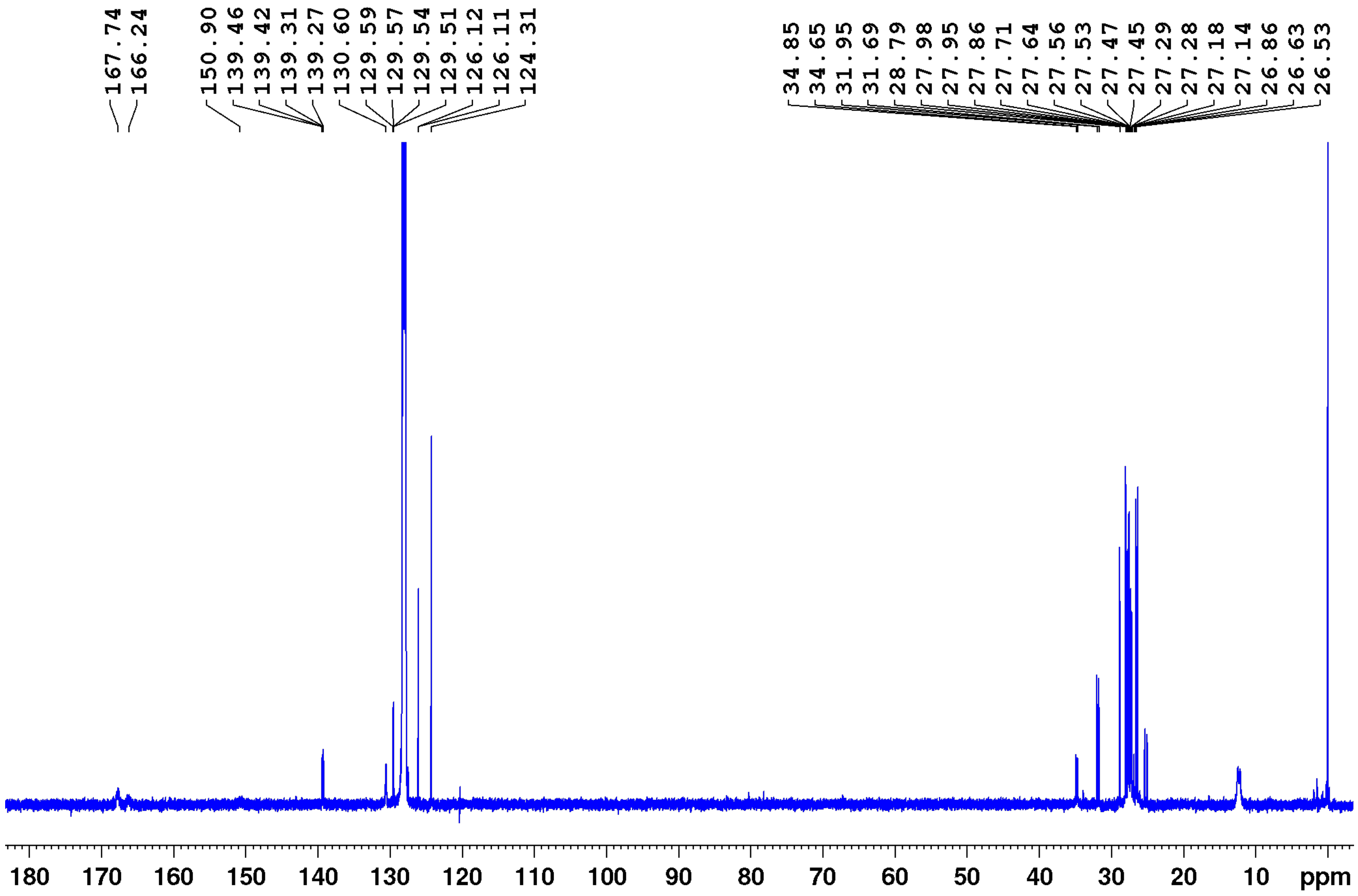

Figure S28. $^{13}C\{^{1}H\}$ NMR spectrum of **11-TMS** in C_6D_6.

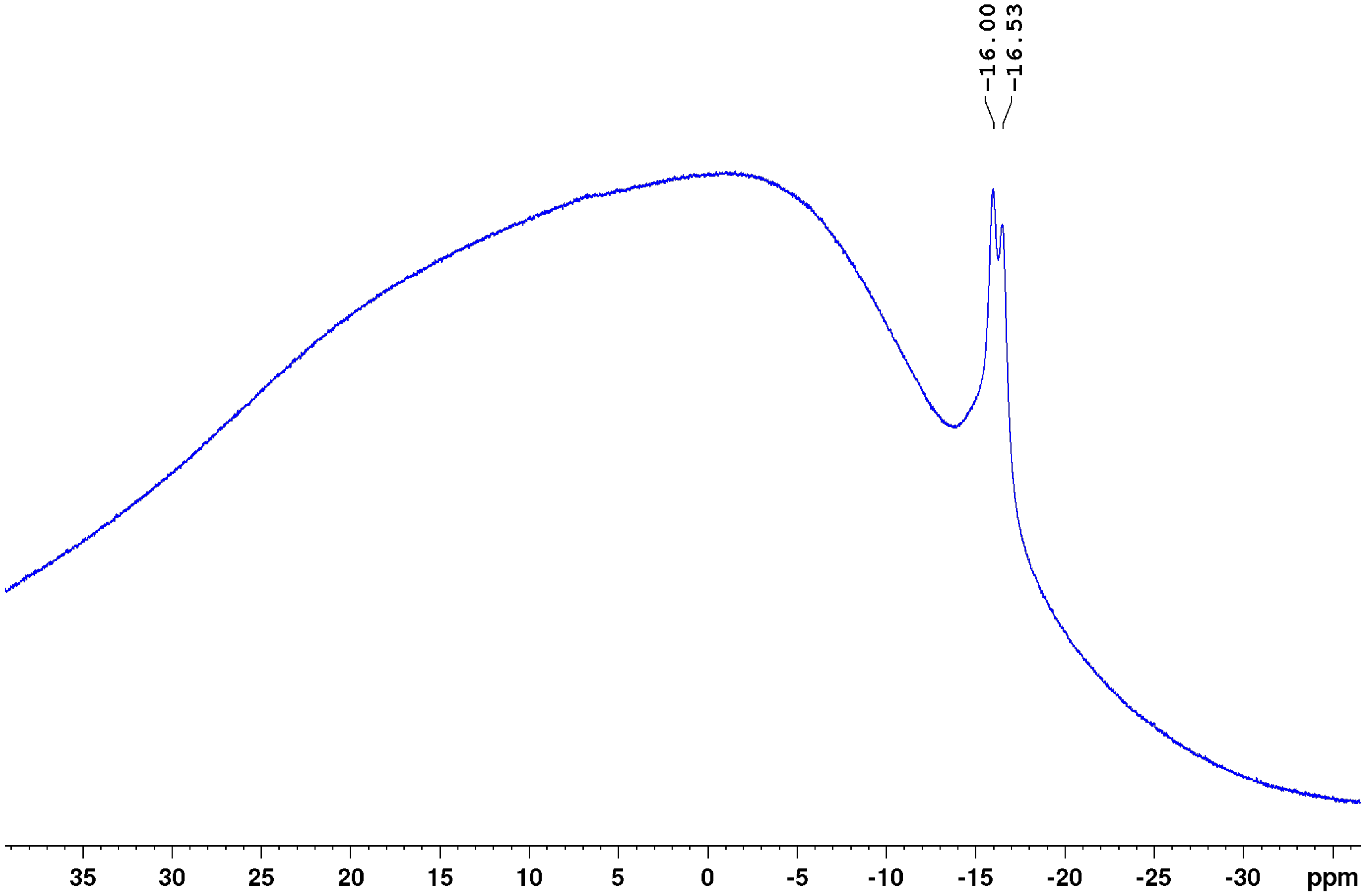

Figure S29. ^{11}B NMR spectrum of **11-TMS** in C_6D_6.

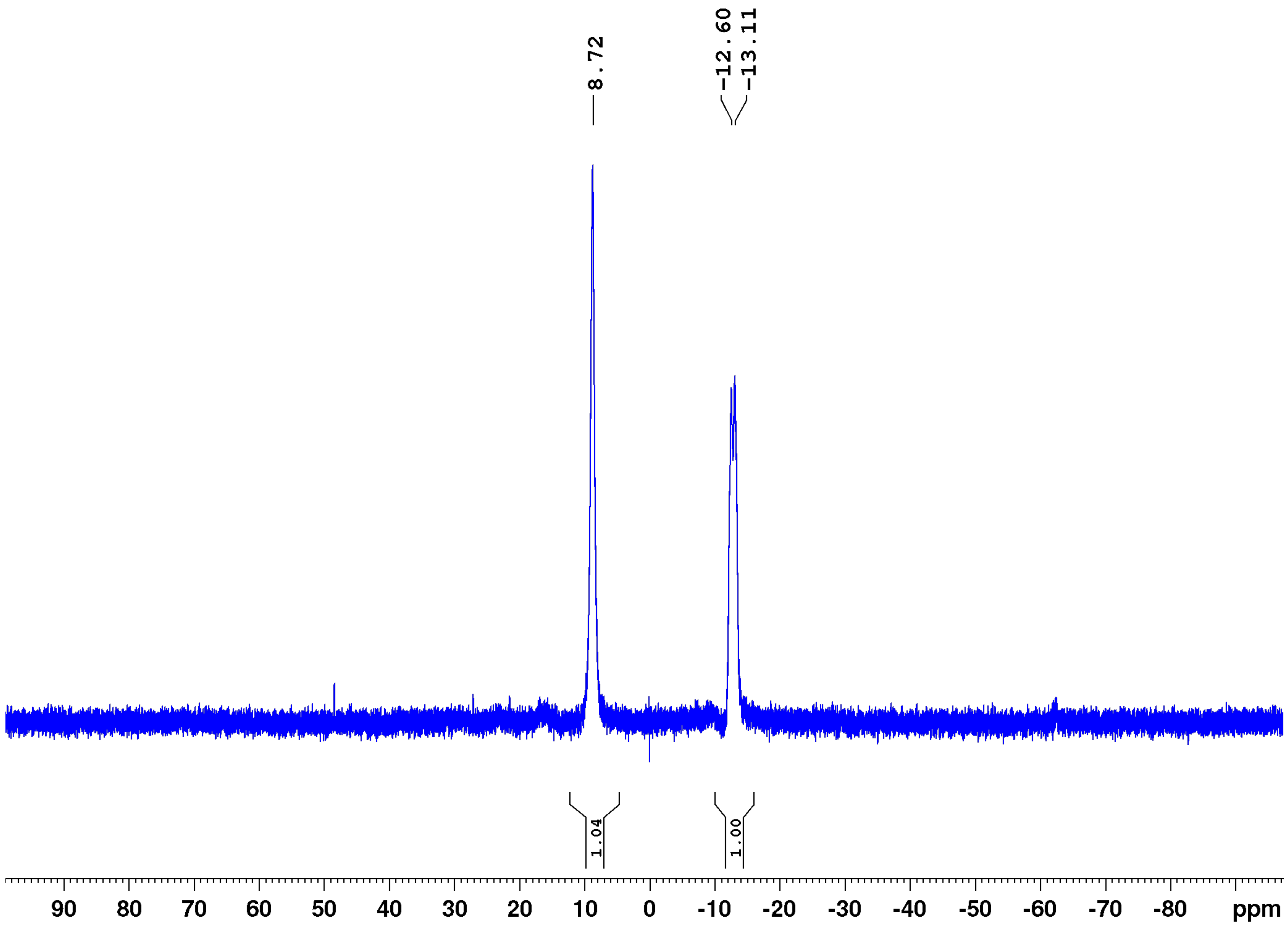

Figure S30. ^{31}P NMR spectrum of **11-TMS** in C_6D_6.

^{1}H and ^{11}B NMR spectra of crude isolated products

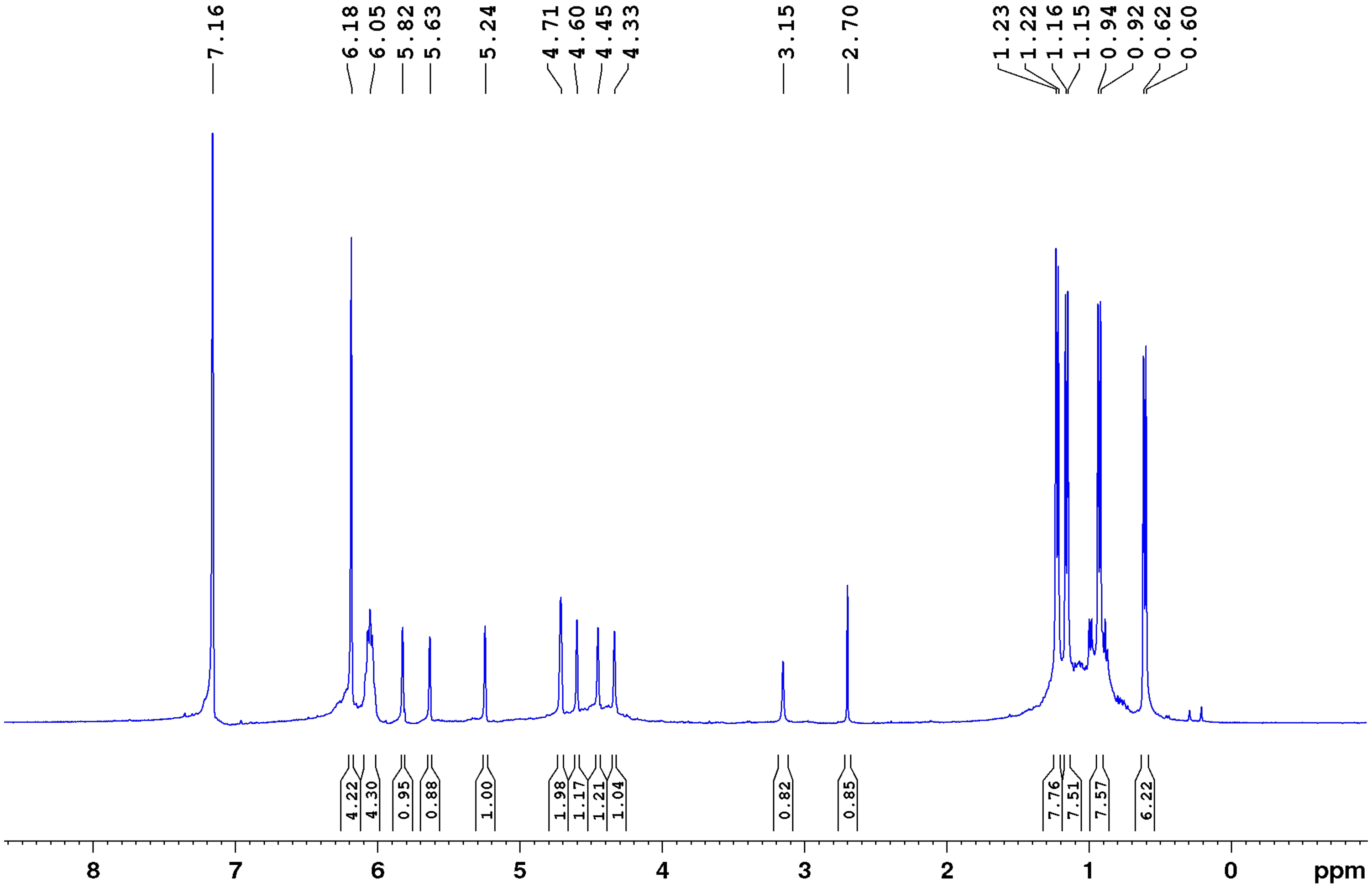

Figure S31. ^{1}H{^{11}B} NMR spectrum of crude **4-H** in C_6D_6.

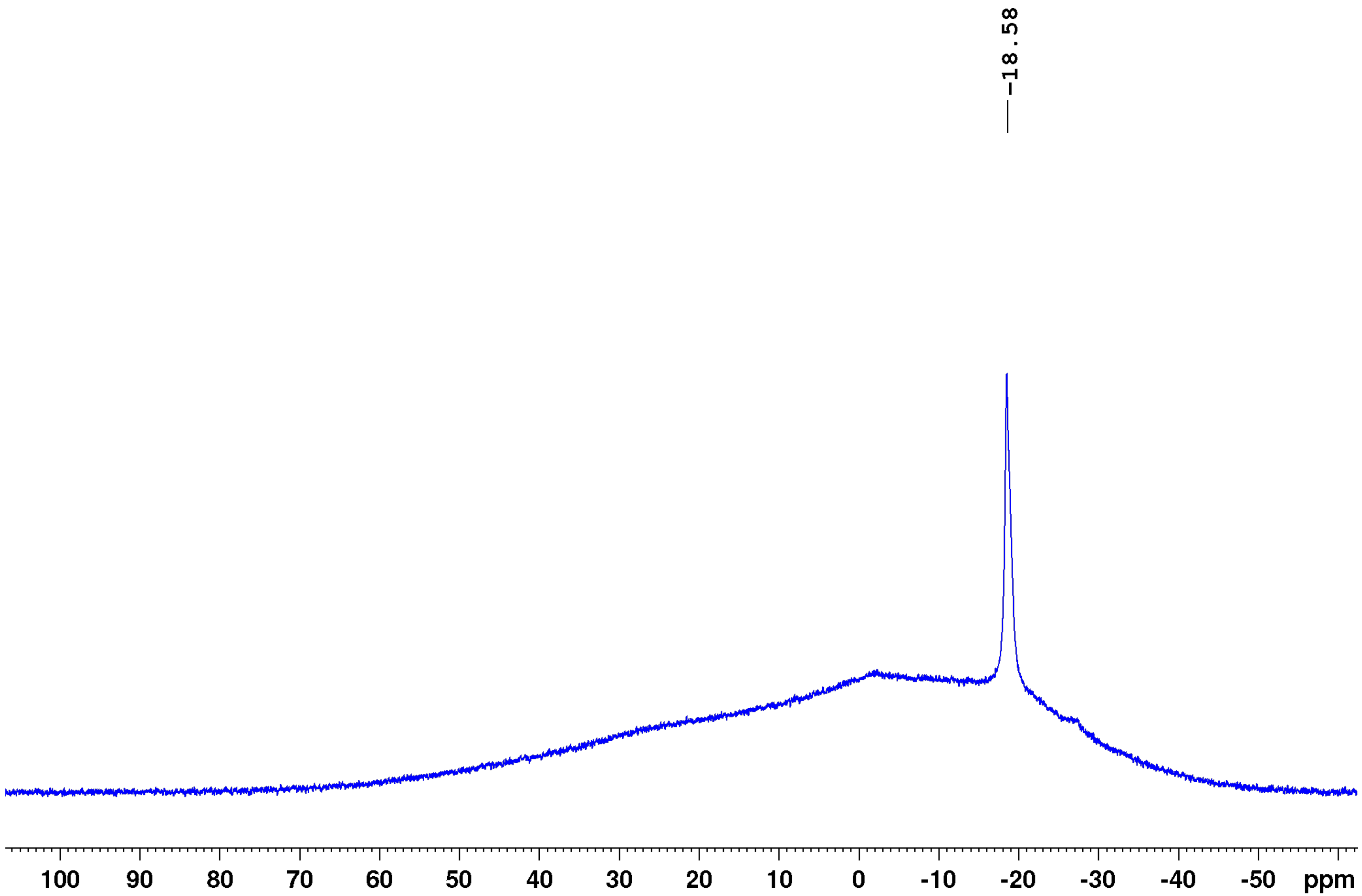

Figure S32. ^{11}B NMR spectrum of crude **4-H** in C_6D_6.

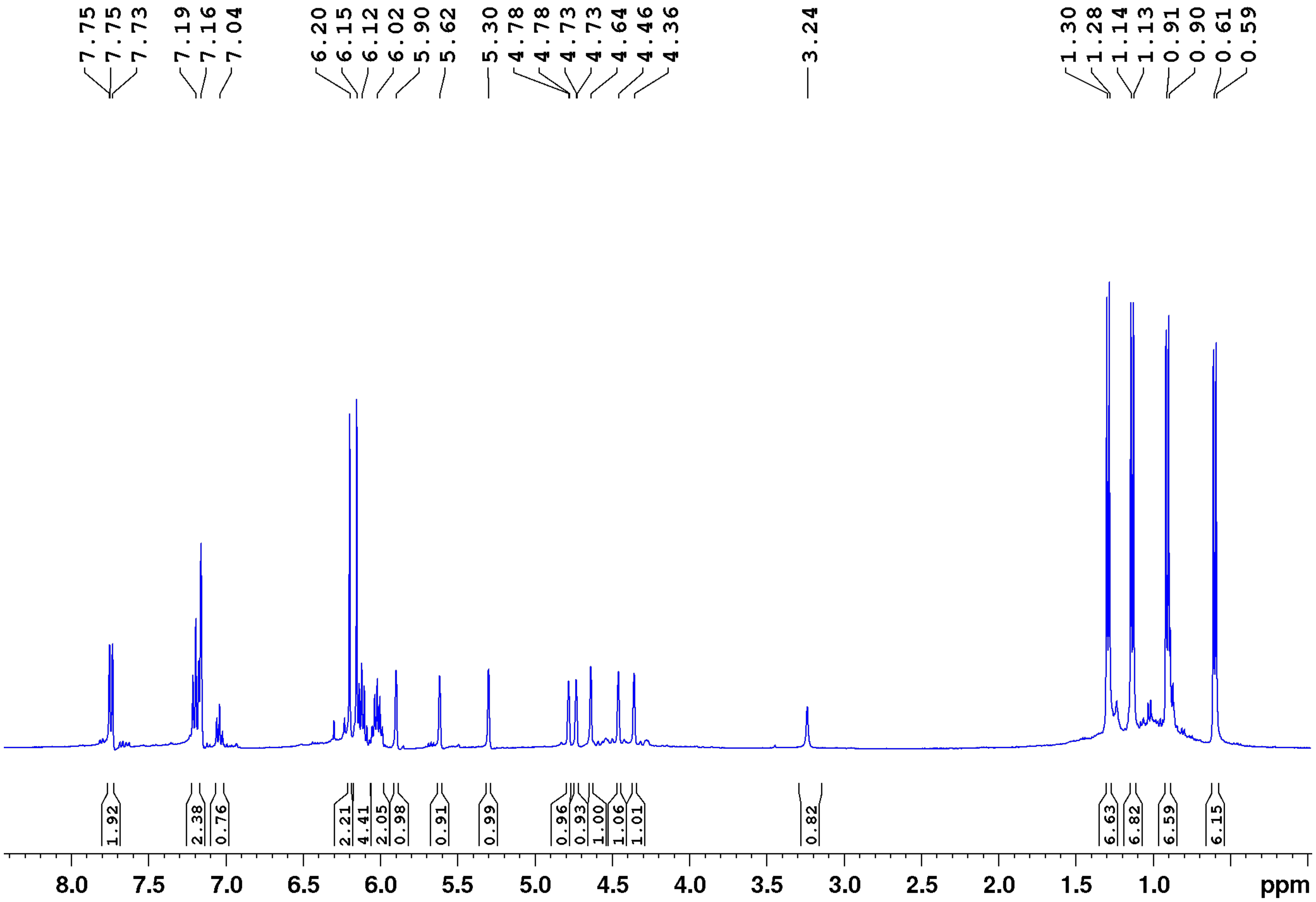

Figure S33. $^{1}H\{^{11}B\}$ NMR spectrum of crude **4-Ph** in C_6D_6.

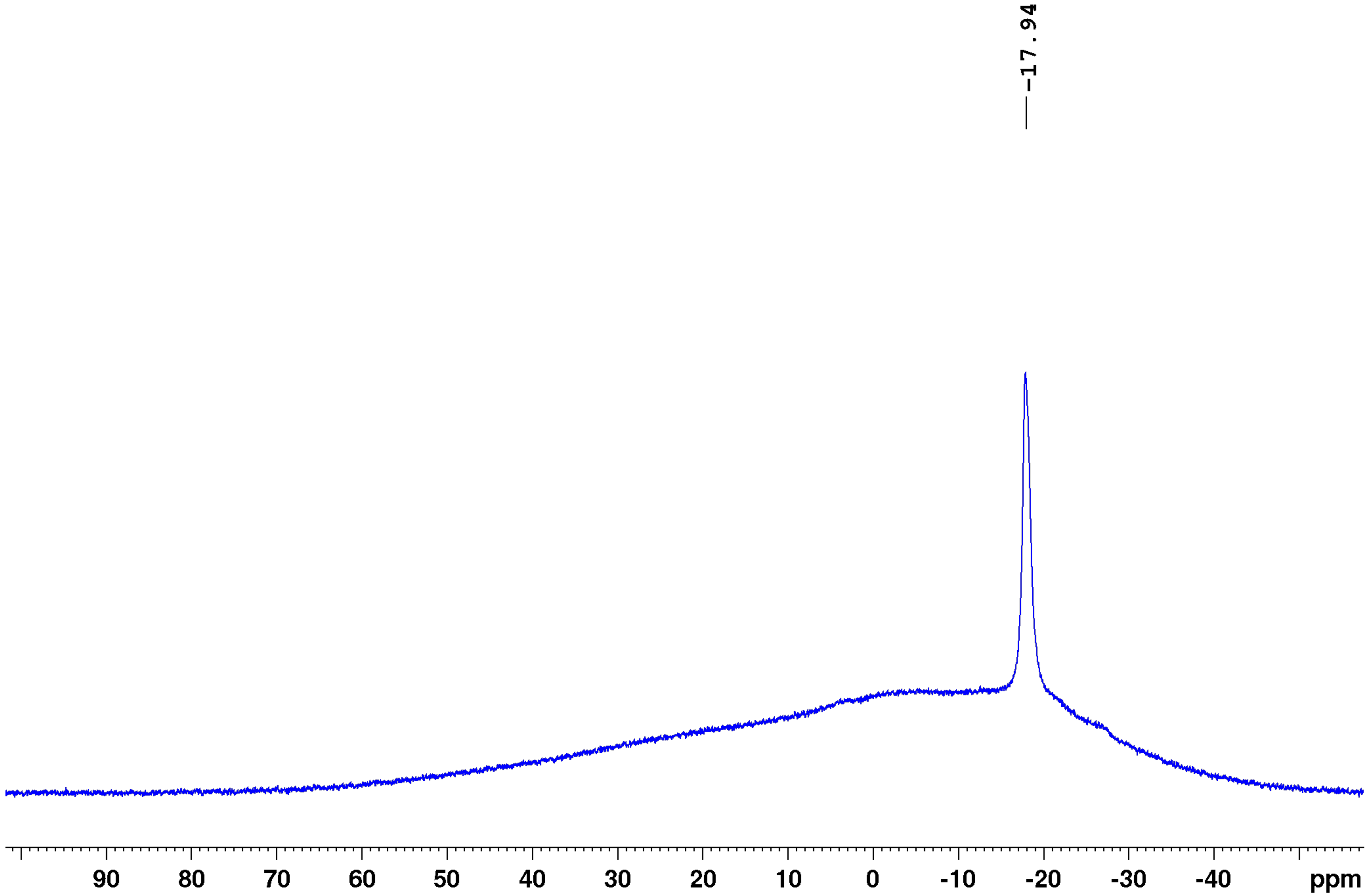

Figure S34. ^{11}B NMR spectrum of crude **4-Ph** in C_6D_6.

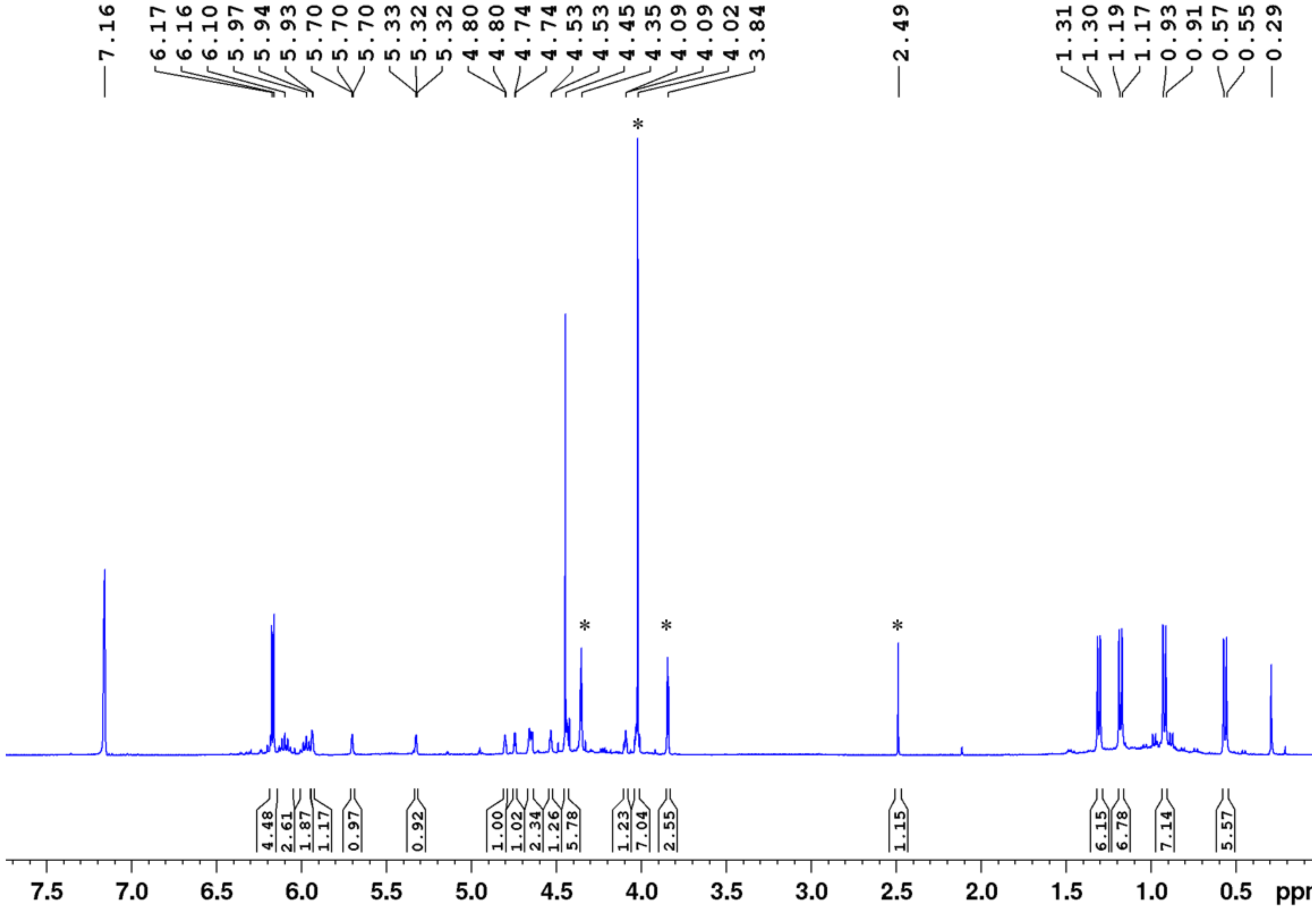

Figure S35. ^{1}H NMR spectrum of crude **4-Fc** in C_6D_6. Resonances marked with * belong to excess ferrocenylacetylene.

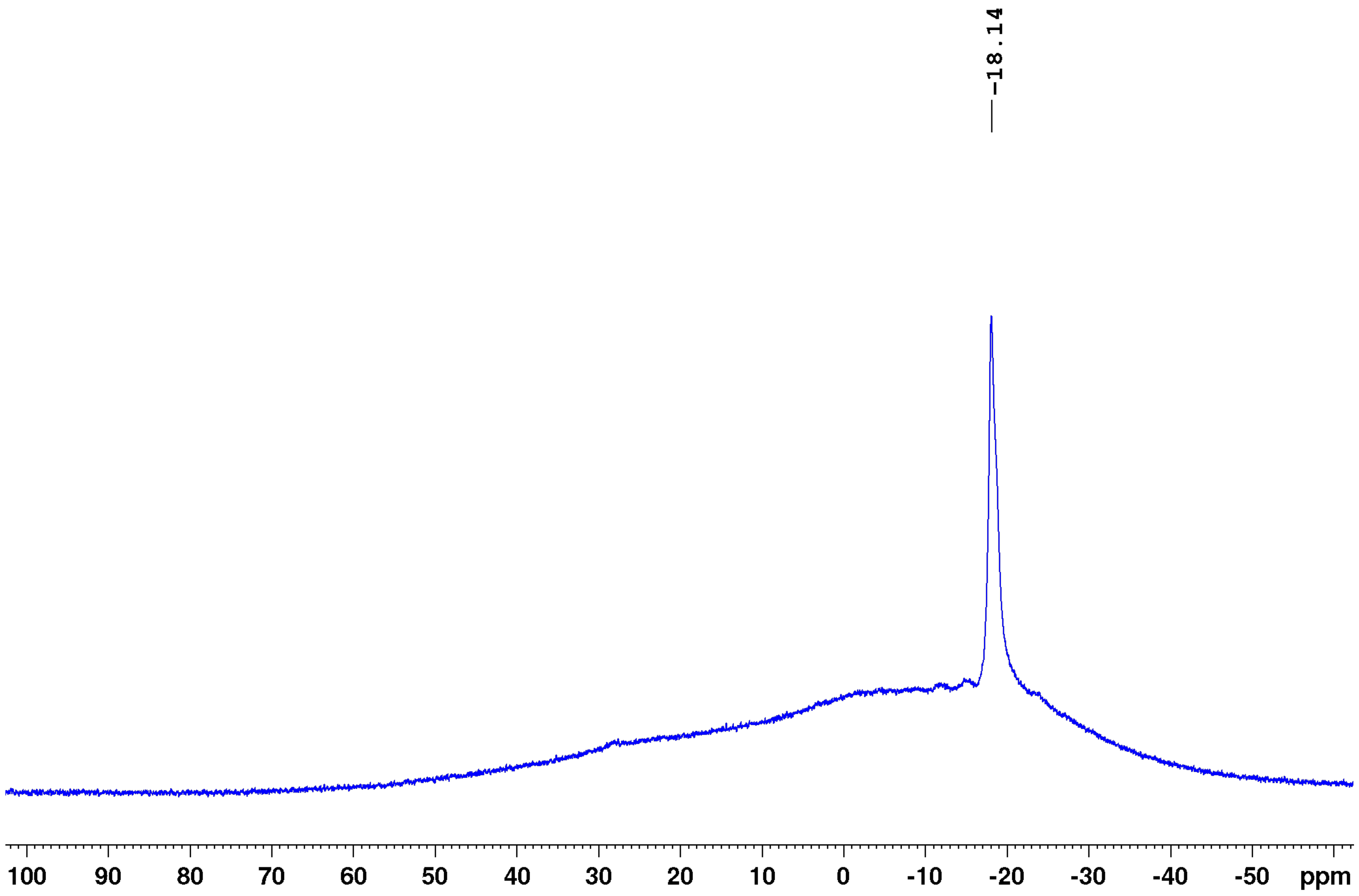

Figure S36. ^{11}B NMR spectrum of crude **4-Fc** in C_6D_6.

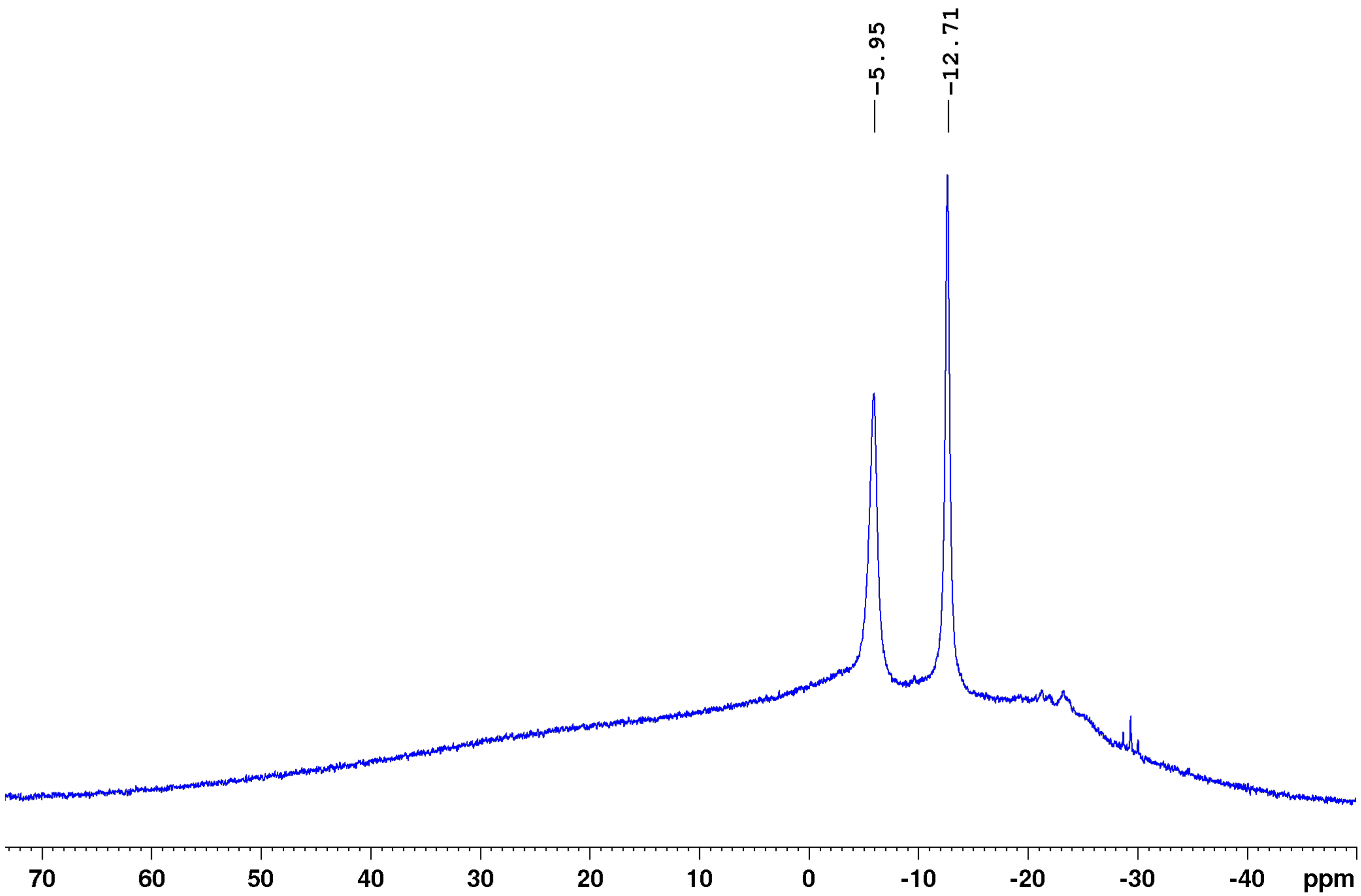

Figure S37. ^{11}B NMR spectrum of crude **7^{Fu}-Fc** in C_6D_6.

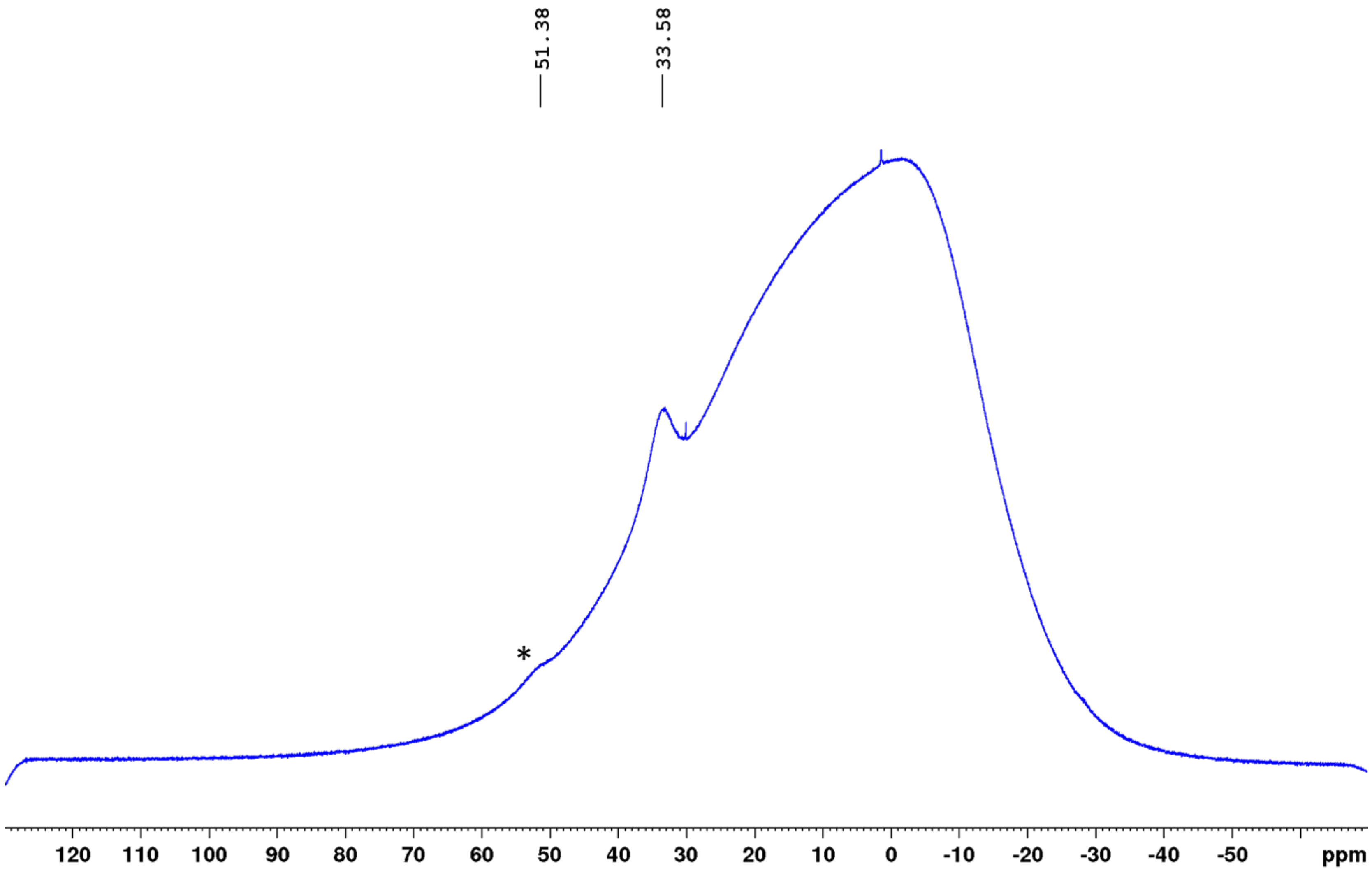

Figure S38. ^{11}B NMR spectrum of crude **8-Tol** in C_6D_6, showing both isomers. δ_{11B} = 51.4 (br, **8-Tol-D**), 33.6 (s, **8-Tol-C**).

^{11}B NMR spectra of reaction mixtures prior to work-up

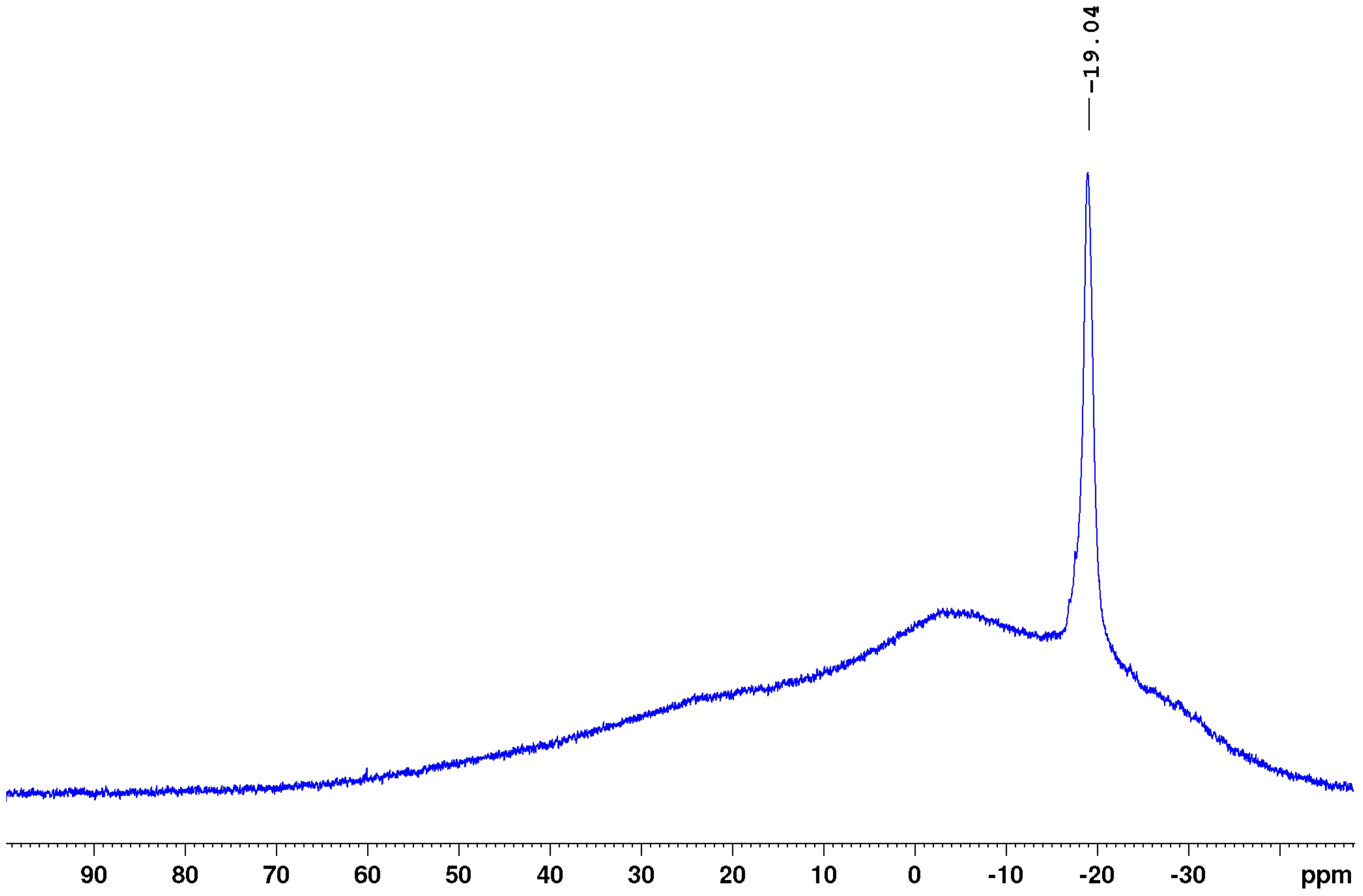

Figure S39. Unlocked ^{11}B NMR spectrum of the reaction of diborene **1** with acetylene after 2 h at rt in C_6H_6. Selectivity for **2-H** (δ_{11B} = –19.0 ppm) ca. 98%.

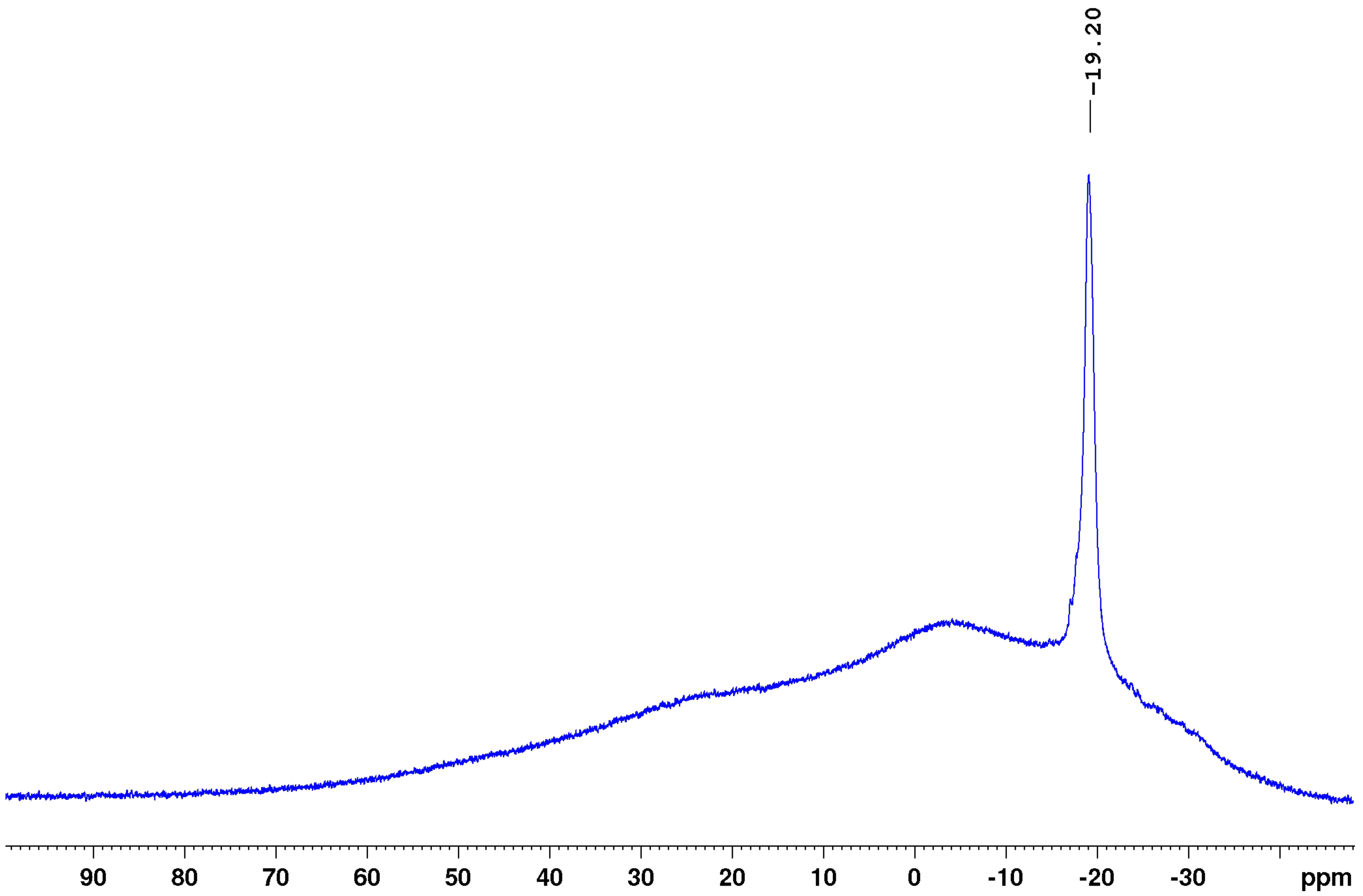

Figure S40. Unlocked ^{11}B NMR spectrum of the reaction of diborene **1** with FcC≡CH after 2 h at rt in C_6H_6. Selectivity for **2-Fc** (δ_{11B} = –19.2 ppm) ca. 98%.

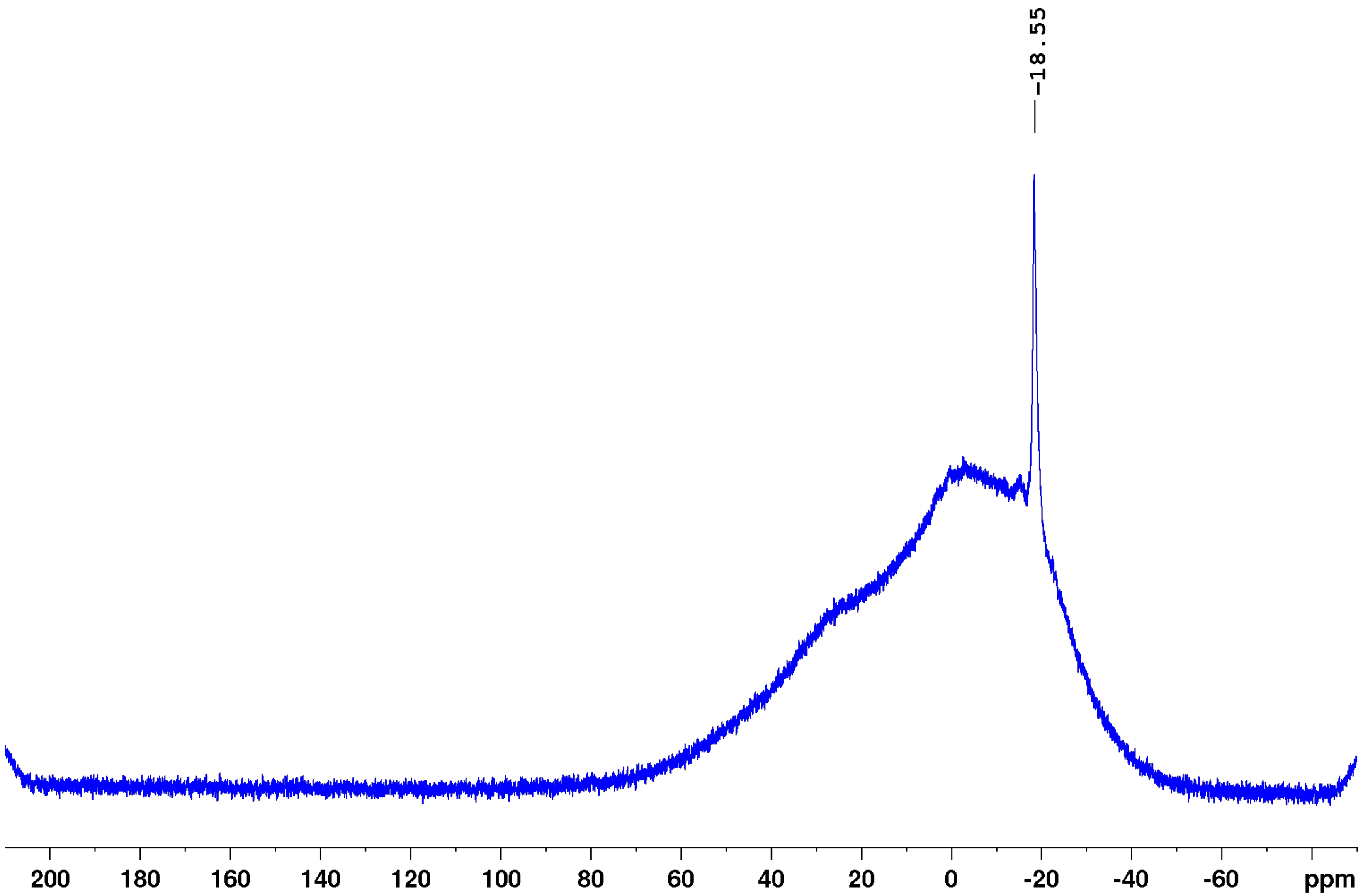

Figure S41. Unlocked ^{11}B NMR spectrum of the reaction of diborene **3** with acetylene after 2 h at rt in C_6H_6. Selectivity for **4-H** (δ_{11B} = –18.6 ppm) ca. 90%.

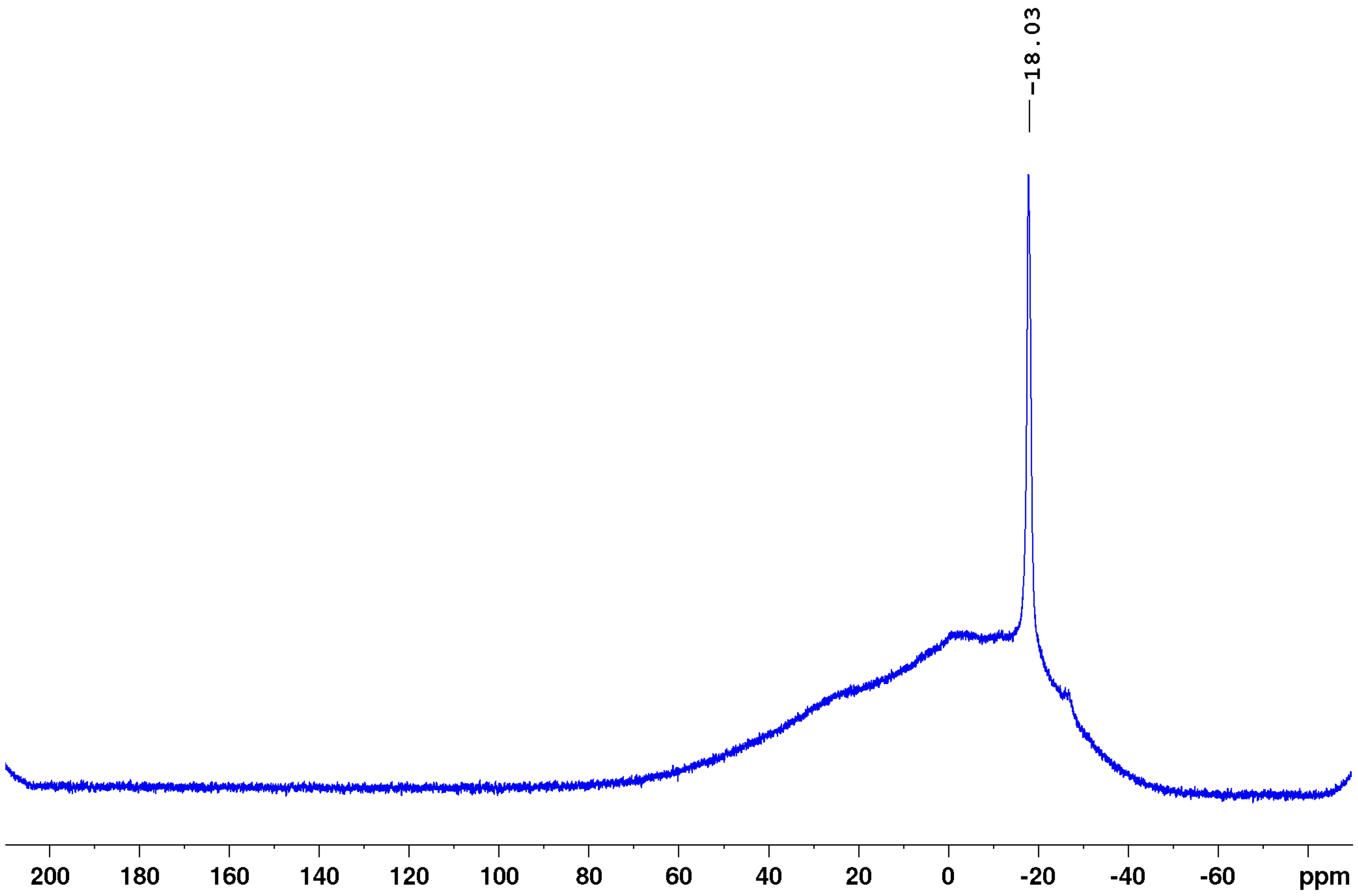

Figure S42. Unlocked ^{11}B NMR spectrum of the reaction of diborene **3** with PhC≡CH after 2 h at rt in C_6H_6. Selectivity for **4-Ph** (δ_{11B} = –18.0 ppm) ca. 90%.

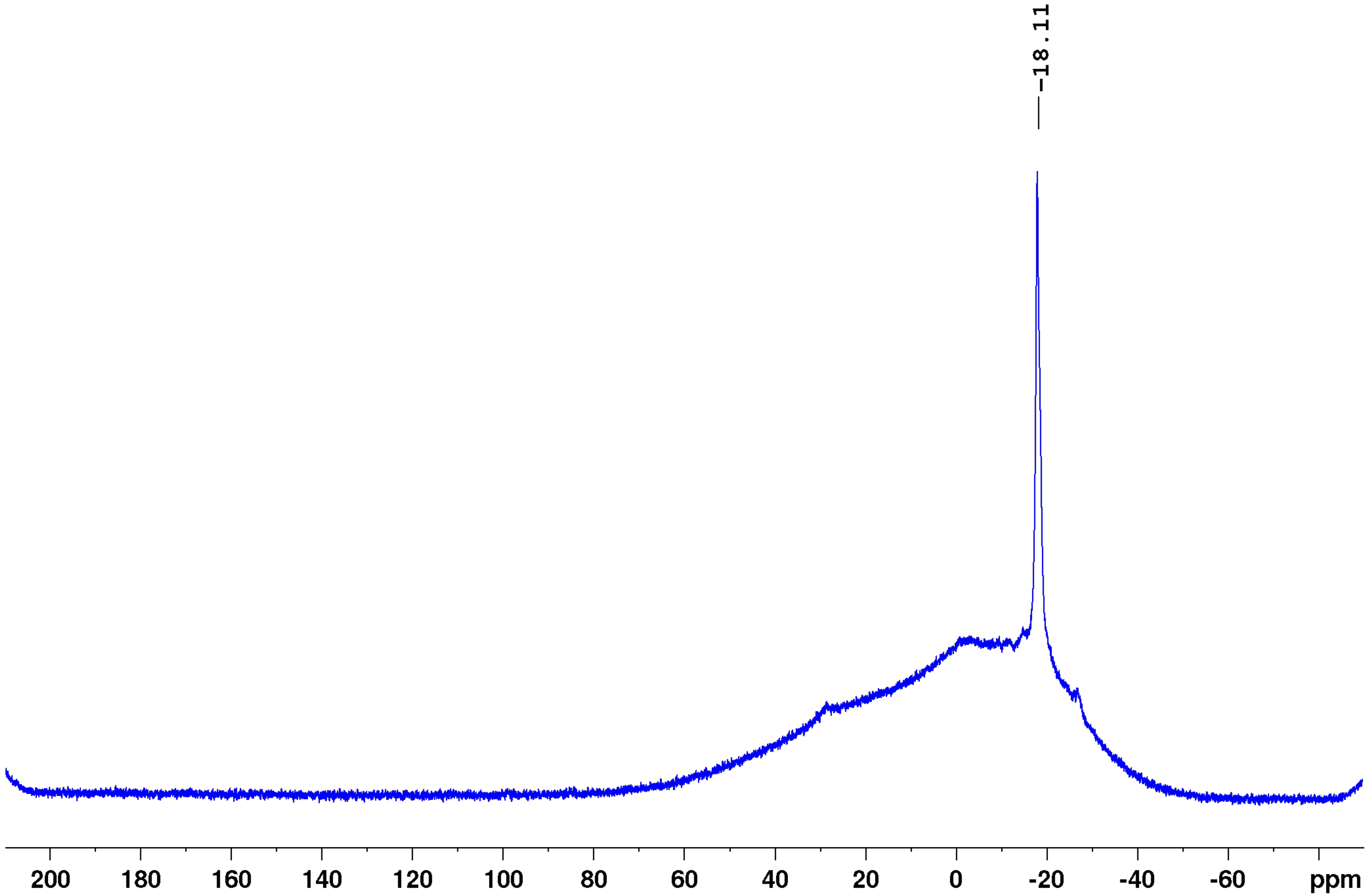

Figure S43. Unlocked ^{11}B NMR spectrum of the reaction of diborene **3** with FcC≡CH after 2 h at rt in C_6H_6. Selectivity for **4-Fc** (δ_{11B} = –18.1 ppm) ca. 85%.

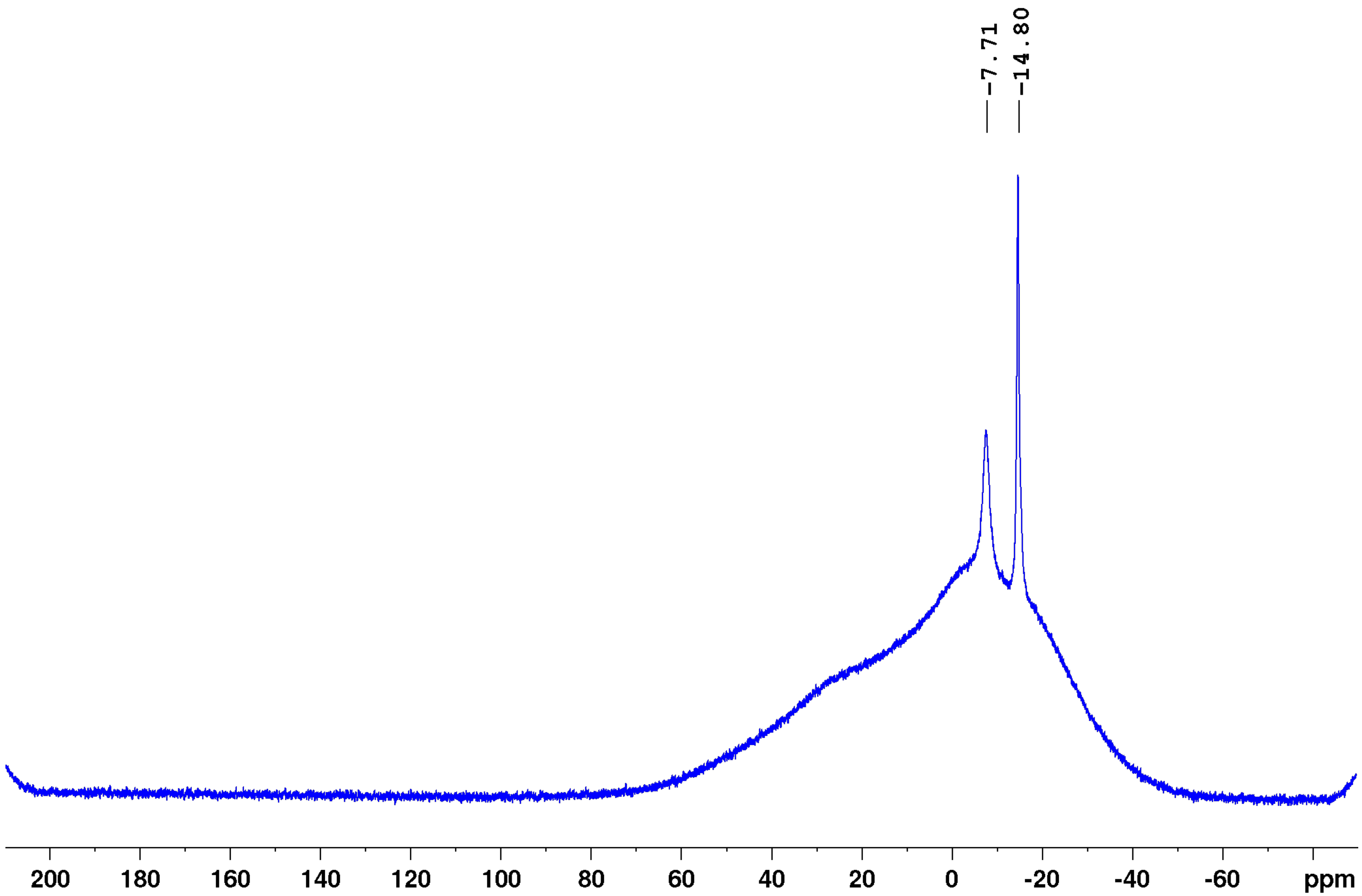

Figure S44. Unlocked ^{11}B NMR spectrum of the reaction of diborene **3** with FcC≡CH after 3 h at 60 °C in C_6H_6. Selectivity for **5-Fc** (δ_{11B} = –7.7, –14.8 ppm) > 99%.

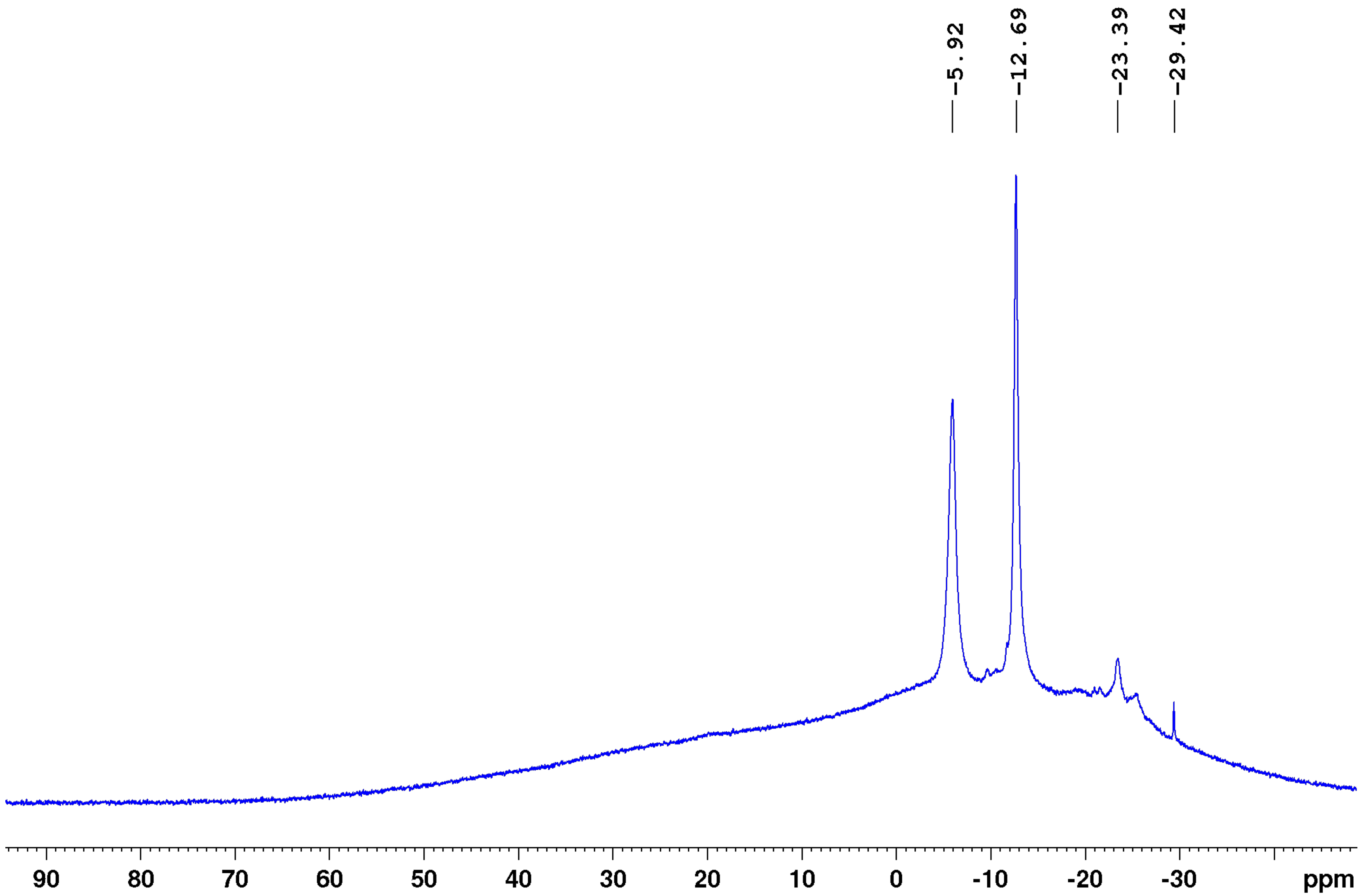

Figure S45. ^{11}B NMR spectrum of the reaction of diborene $\mathbf{6^{Fu}}$ with FcC≡CH after 2 days at rt in C_6D_6. Selectivity for $\mathbf{7^{Fu}}$**-Fc** (δ_{11B} = –5.9, –12.7 ppm) ca. 80%.

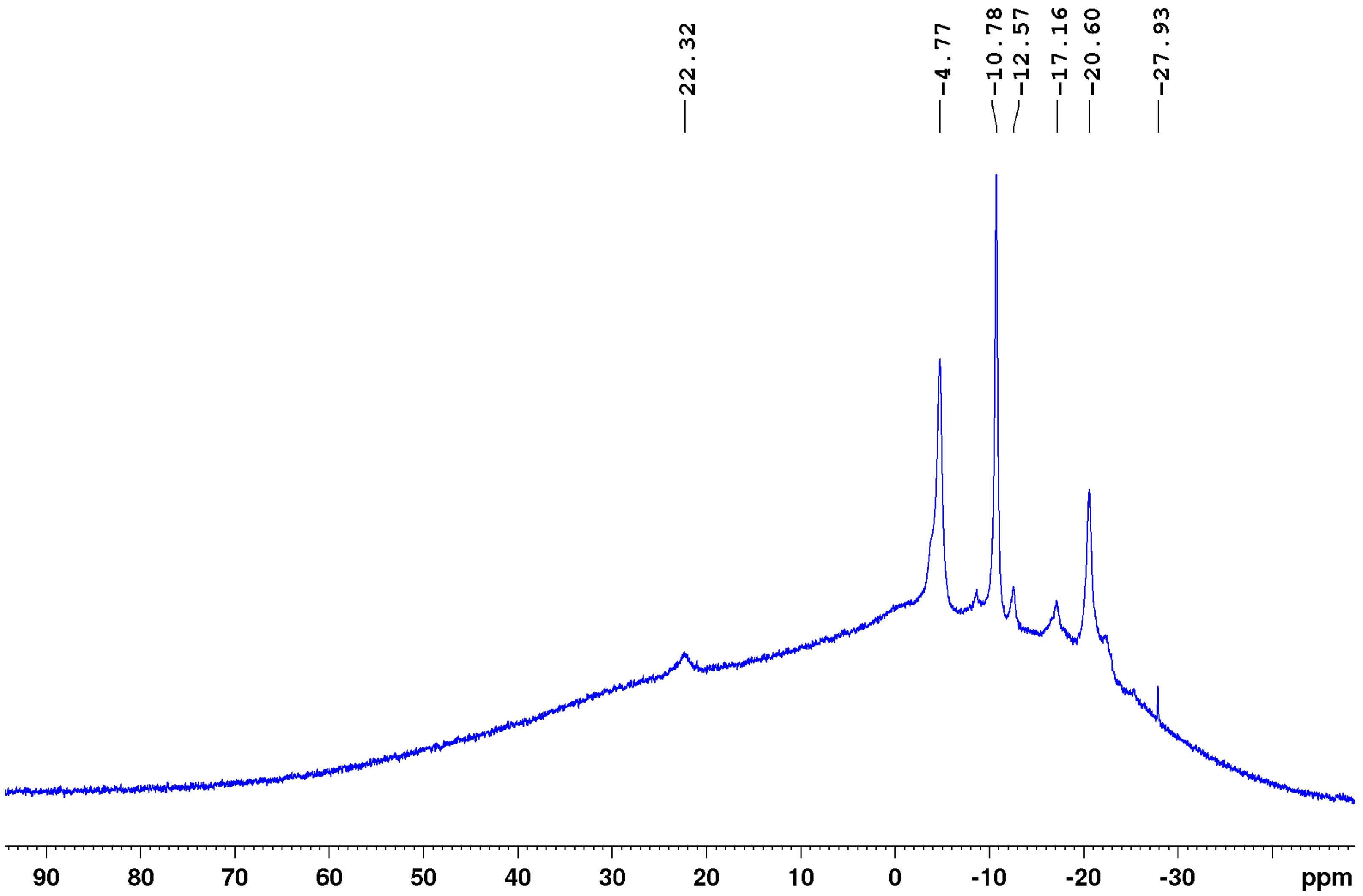

Figure S46. ^{11}B NMR spectrum of the reaction of diborene **6^{Tn}** with FcC≡CH after 4 days at rt in C_6D_6. Selectivity for **7^{Tn}-Fc** (δ_{11B} = –4.8, –10.8 ppm) ca. 65%.

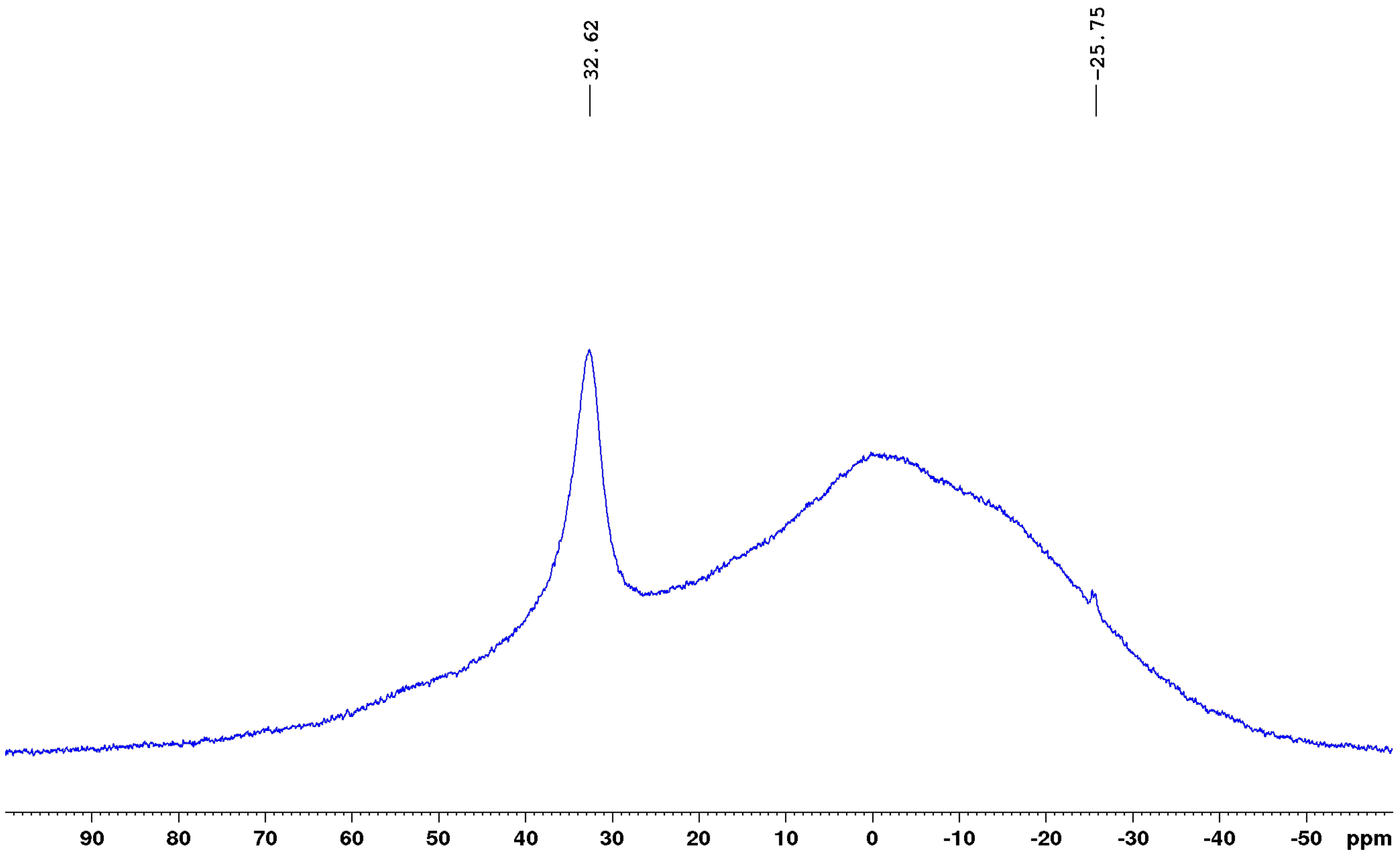

Figure S47. Unlocked ^{11}B NMR spectrum of the reaction of diborene **I** with *n*BuC≡CH after 3 days at 80 °C in C_6H_6. Selectivity for **8-Bu-C** (δ_{11B} = 32.6 ppm) and **8-Bu-D** (δ_{11B} = 54 ppm, very broad) ca. 98%.

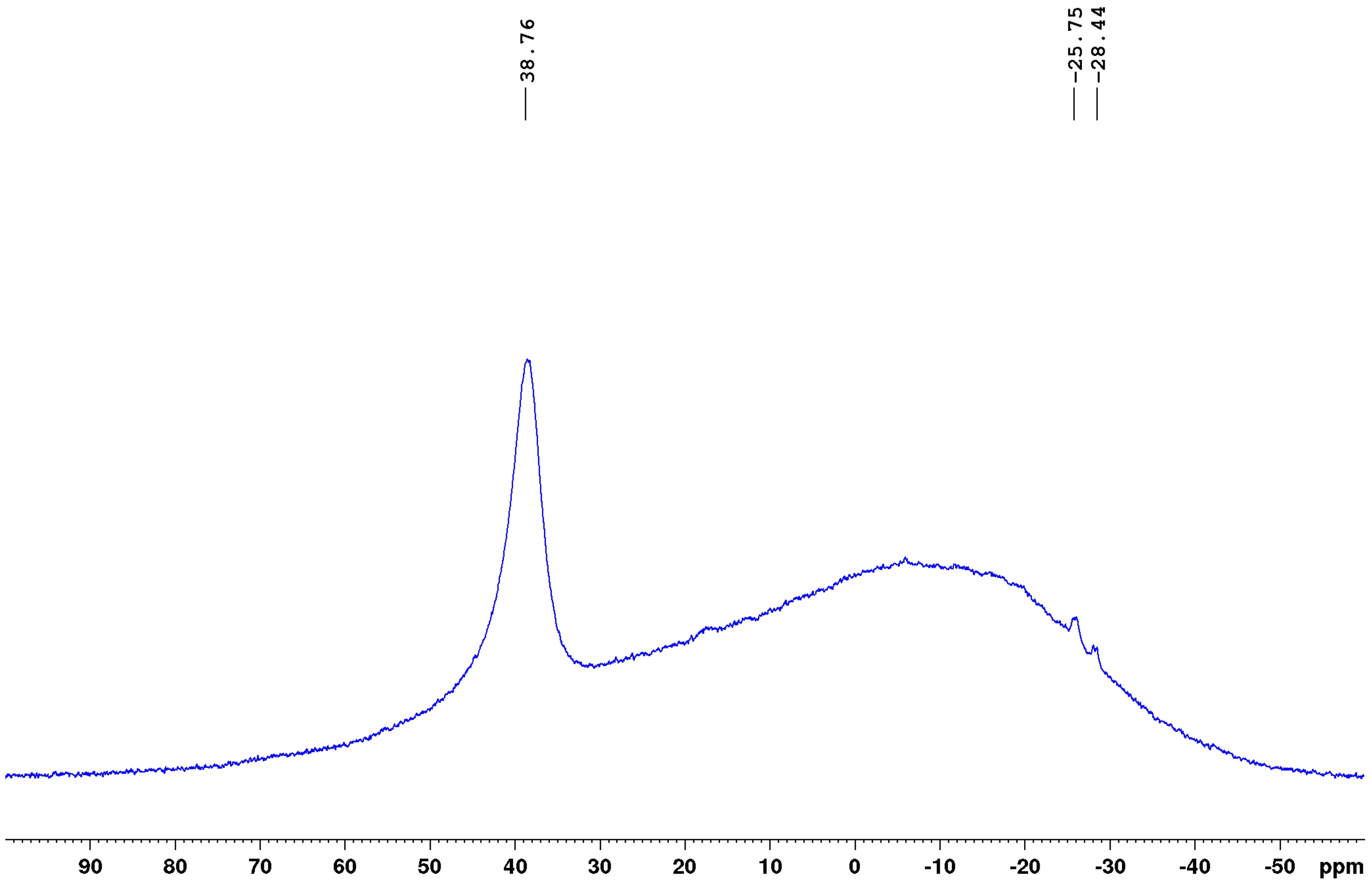

Figure S48. Unlocked ^{11}B NMR spectrum of the reaction of diborene **I** with $Me_3SiC{\equiv}CH$ after 6 days at 80 °C in C_6H_6. Selectivity for **8-TMS** (δ_{11B} = 38.8 ppm) ca. 95%.

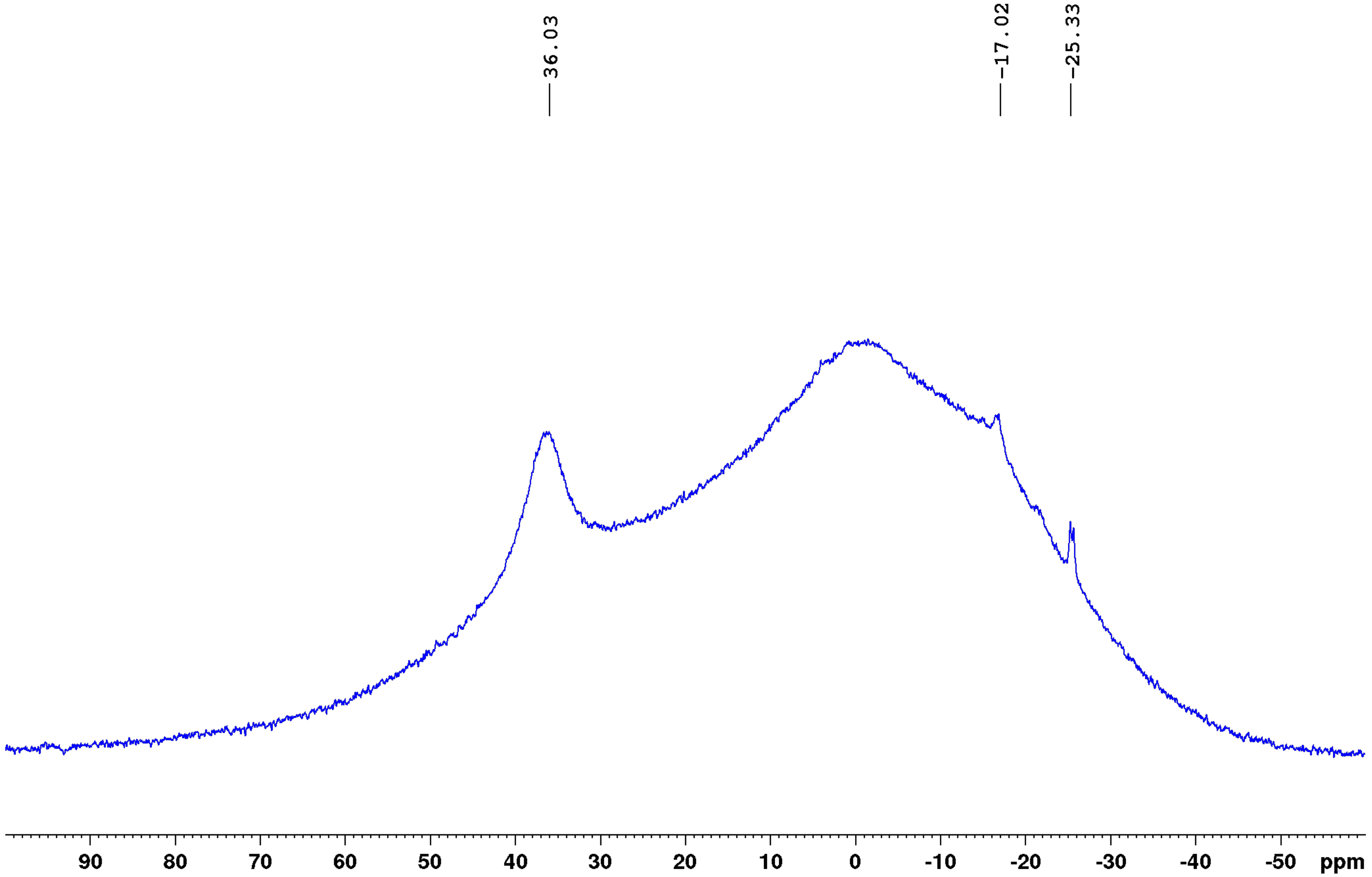

Figure S49. Unlocked ^{11}B NMR spectrum of the reaction of diborene **I** with (*p*-tol)C≡CH after 6 days at 80 °C in C_6H_6. Selectivity for **8-Tol-C** (δ_{11B} = 36.0 ppm) ca. 85%. **8-Tol-D** not visible due to line-broadening.

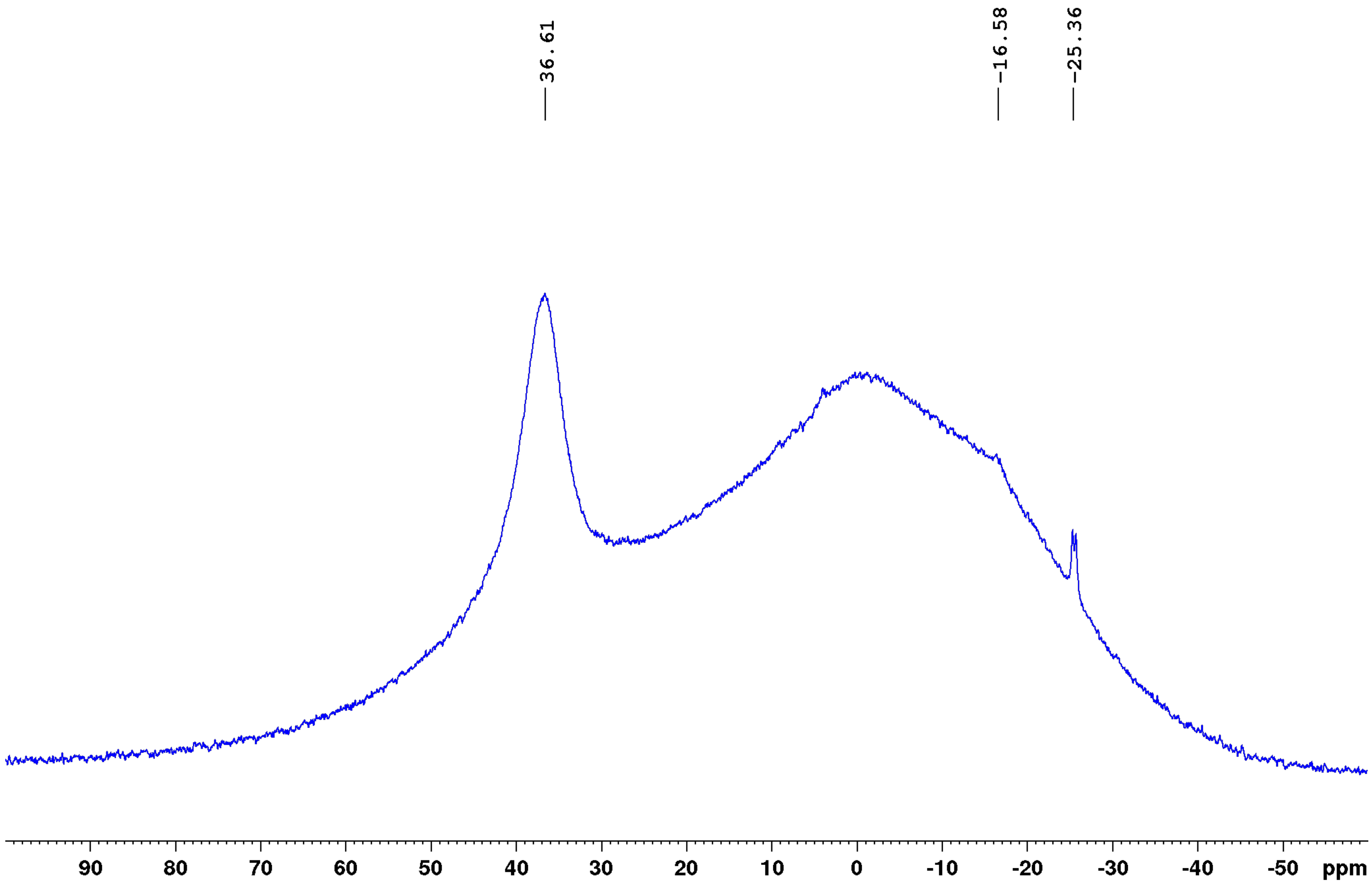

Figure S50. Unlocked ^{11}B NMR spectrum of the reaction of diborene **I** with FcC≡CH after 3 days at 80 °C in C_6H_6. Selectivity for **8-Fc** (δ_{11B} = 36.0 ppm) ca. 90%.

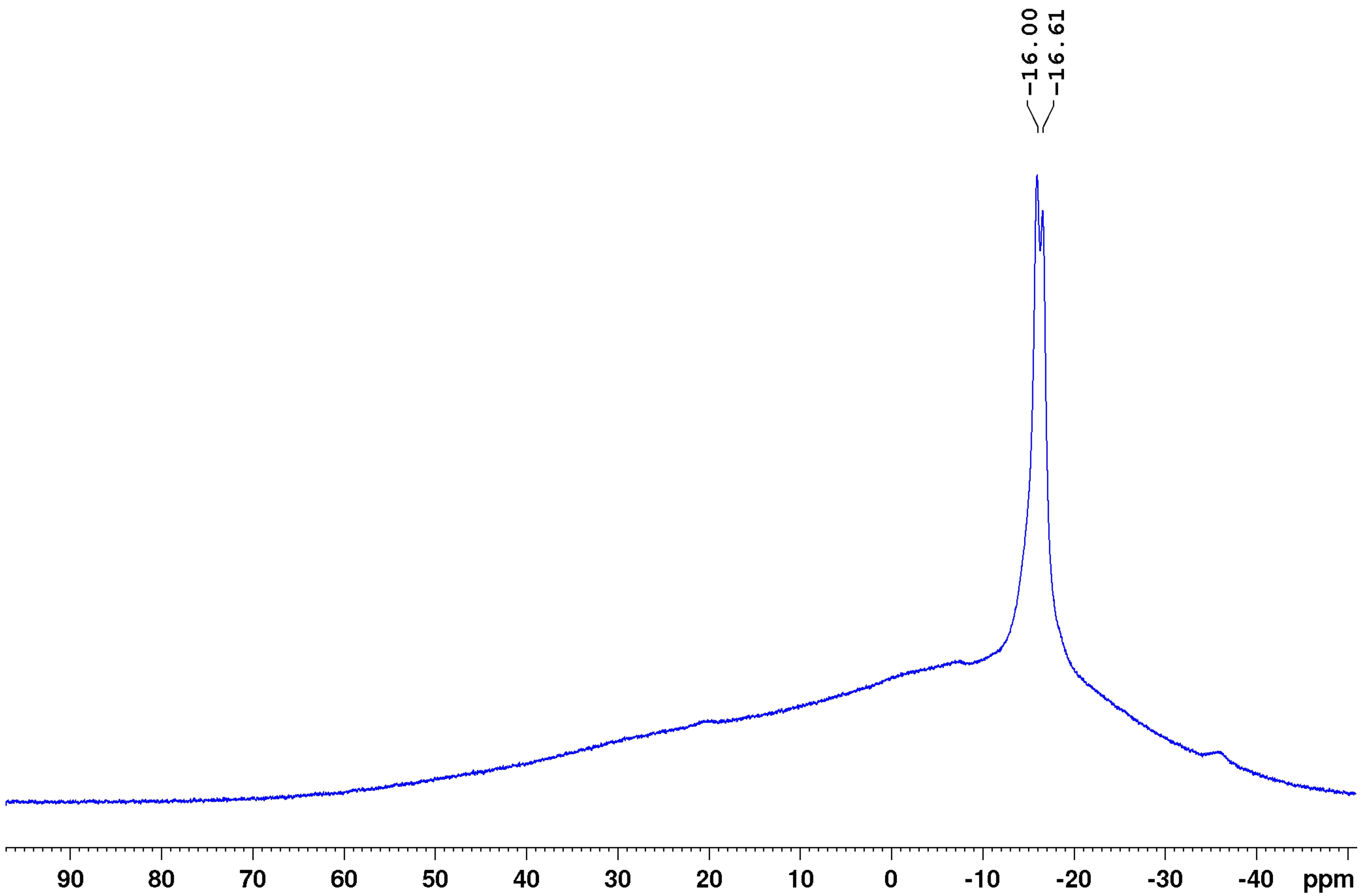

Figure S51. ^{11}B NMR spectrum of the reaction of diborene **10** with $Me_3SiC{\equiv}CH$ after 18 h at 60 °C in C_6D_6. Selectivity for **11-TMS** (δ_{11B} = –16.3 ppm) ca. 90%.

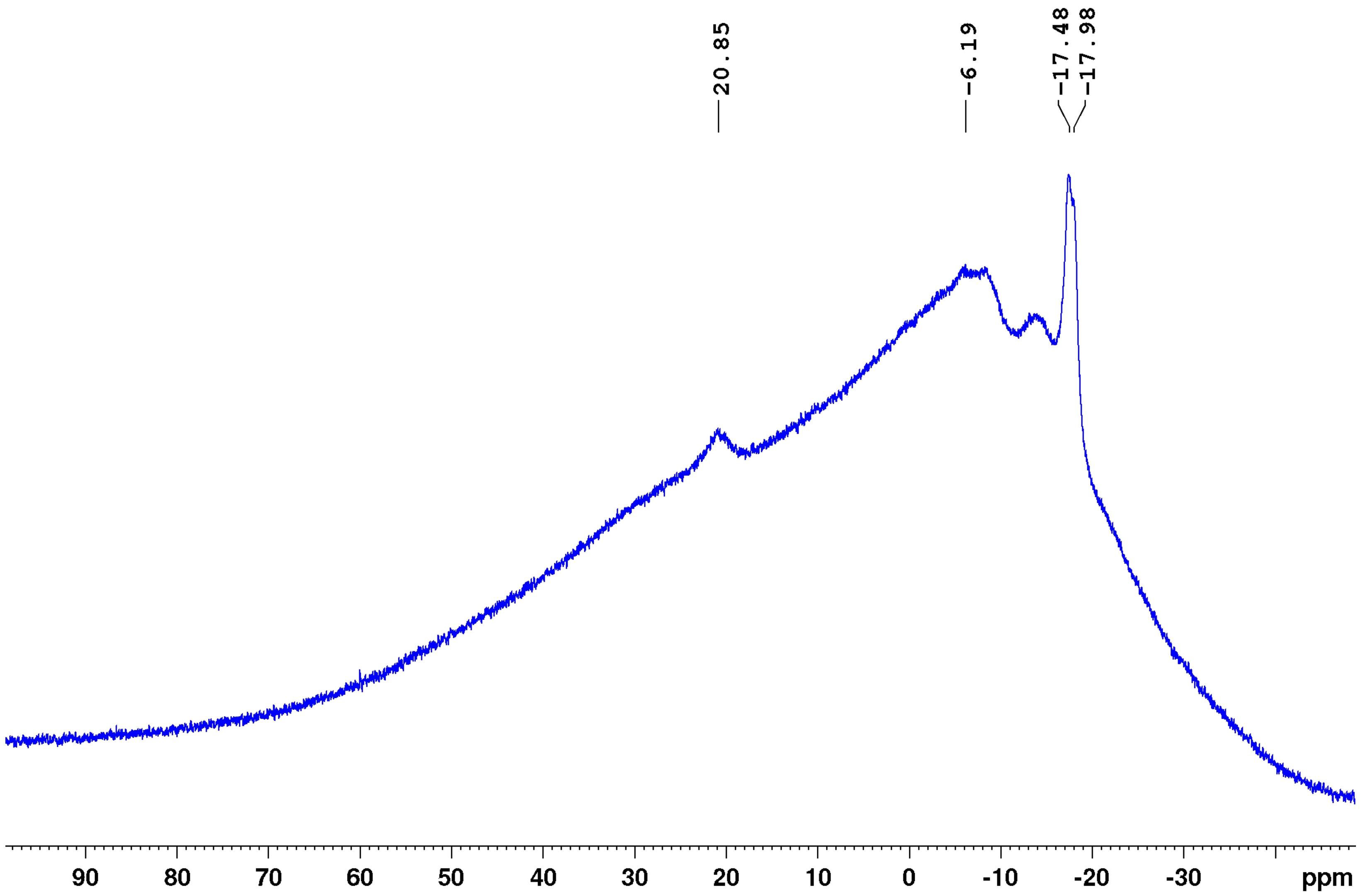

Figure S52. ^{11}B NMR spectrum of the reaction of diborene **10** with FcC≡CH after 4 d at rt in C_6D_6. Selectivity for **11-Fc** (δ_{11B} = –17.5, –18.0 ppm) ca. 45%.

X-ray crystallographic data

The crystal data of **7^{Tn}-Fc**, **8-Bu-D** and **9-Tol** were collected on a Bruker X8-APEX II diffractometer with a CCD area detector and multi-layer mirror monochromated $Mo_{K\alpha}$ radiation. The crystal data of **2-Fc**, **4-H**, **5-Fc** and **11-TMS** were collected on a Bruker D8 Quest diffractometer with a CMOS area detector and multi-layer mirror monochromated $Mo_{K\alpha}$ radiation. The crystal data of **8-TMS/Tol/Fc-C** were collected on a XtaLAB Synergy, Dualflex diffractometer with a HyPix area detector and multi-layer mirror monochromated $Cu_{K\alpha}$ radiation. The structures were solved using intrinsic phasing method,[7] refined with the ShelXL program[8] and expanded using Fourier techniques. All non-hydrogen atoms were refined anisotropically. Hydrogen atoms were included in structure factor calculations. All Hydrogen atoms except those attached to boron were assigned to idealised positions. The coordinates of boron-bound hydrogen atoms were refined freely.

Crystallographic data have been deposited with the Cambridge Crystallographic Data Centre as supplementary publication no. CCDC **2076364–2076374**. These data can be obtained free of charge from The Cambridge Crystallographic Data Centre *via* www.ccdc.cam.ac.uk/data_request/cif.

Crystal data for 2-Fc: $C_{44}H_{68}B_2FeN_4$, M_r = 730.49, yellow block, 0.324×0.19×0.157 mm^3, triclinic space group $P\bar{1}$, a = 11.183(2) Å, b = 14.077(3) Å, c = 15.999(4) Å, α = 73.040(11)°, β = 74.517(6)°, γ = 66.833(13)°, V = 2181.4(9) Å^3, Z = 2, ρ_{calcd} = 1.112 g·cm^{-3}, μ = 0.379 mm^{-1}, F(000) = 792, T = 100(2) K, R_1 = 0.0559, $wR2$ = 0.0922, 8586 independent reflections [$2\theta \leq 52.042°$] and 479 parameters.

Refinement details for 4-H: The asymmetric unit contains 1.5 toluene molecules. The half-molecule is positioned on an inversion centre and was modelled as twofold disordered in a 67:33 ratio, with ADPs restrained using SIMU 0.01. Five reflections affected by the beamstop were omitted.

Crystal data for 4-H: $C_{37}H_{50}B_2FeN_4\cdot(C_7H_8)_{0.5}$, $M_r = 674.34$, yellow plate, 0.39×0.22×0.03 mm^3, monoclinic space group $P2_1/n$, $a = 11.1288(18)$ Å, $b = 24.205(4)$ Å, $c = 13.651(2)$ Å, $\beta = 95.012(12)$°, $V = 3663.1(11)$ Å^3, $Z = 4$, $\rho_{calcd} = 1.223$ g·cm^{-3}, $\mu = 0.446$ mm^{-1}, $F(000) = 1444$, $T = 100(2)$ K, $R_1 = 0.0497$, $wR2 = 0.0776$, 7773 independent reflections [$2\theta \leq 53.466$°] and 527 parameters.

Crystal data for 5-Ph: $C_{36}H_{46}B_2FeN_4$, $M_r = 612.24$, orange block, 0.307×0.203×0.097 mm^3, monoclinic space group $P\ 2_1/n$, $a = 11.39750(6)$ Å, $b = 19.08180(12)$ Å, $c = 29.38682(19)$ Å, $\alpha = 90.0$°, $\beta = 90.9480(5)$°, $\gamma = 90.0$°, $V = 6390.31(7)$ Å^3, $Z = 8$, $\rho_{calcd} = 1.273$ g·cm^{-3}, $\mu = 4.011$ mm^{-1}, $F(000) = 2608.000$, $T = 100.00(10)$ K, $R_1 = 0.0472$, $wR2 = 0.1062$, 13410 independent reflections [$2\theta \leq 154.9616$°] and 791 parameters.

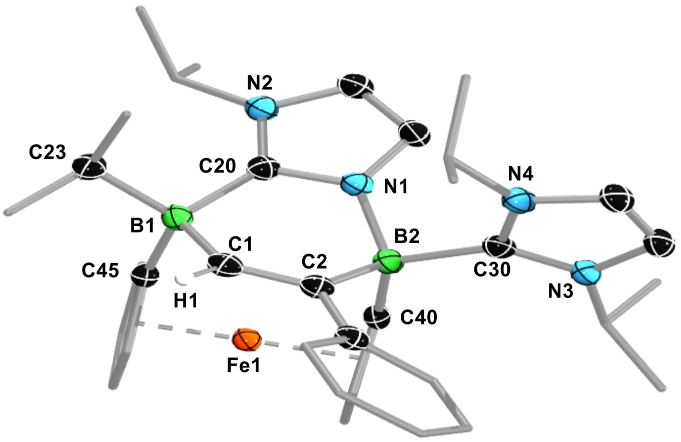

Figure S53. Crystallographically-derived molecular structures of **5-Ph** (one of the two distinct molecular units found in the asymmetric unit) Atomic displacement ellipsoids set at 50% probability. Ellipsoids of ligand periphery and hydrogen atoms omitted for clarity, except for alkene and boron-bound hydrogens. Selected bond lengths (Å) and angles (°): B1–C1 1.630(3), C1–C2 1.341(3), C2–B2 1.624(3), B2–N1 1.584(2), N1–C20 1.345(2), C20–B1 1.634(3), C20–N2 1.361(2), B2–C30 1.652(3), Cp tilt angle 3.7.

Refinement details for 5-Fc: The asymmetric unit contains two heavily disordered benzene molecules, modelled as fourfold disordered in a 20:12:55:13 ratio (RESI 11 - 14) and threefold disordered in a 59:21:20 (RESI 21- 23), respectively, using SUMP 1.0. The benzene rings within these residues were idealised with AFIX 66 and their ADPs restrained with SIMU 0.005.

Crystal data for 5-Fc: $C_{40}H_{60}B_2Fe_2N_4\cdot(C_6H_6)_2$, $M_r = 876.37$, orange block, $0.112\times0.068\times0.053$ mm^3, triclinic space group $P\bar{1}$, $a = 10.7631(17)$ Å, $b = 14.107(3)$ Å, $c = 17.311(4)$ Å, $\alpha = 66.988(9)°$, $\beta = 73.379(11)°$, $\gamma = 72.819(18)°$, $V = 2267.4(9)$ Å^3, $Z = 2$, $\rho_{calcd} = 1.284$ g·cm^{-3}, $\mu = 0.680$ mm^{-1}, $F(000) = 928$, $T = -173$ K, $R_1 = 0.0604$, $wR2 = 0.1078$, 8938 independent reflections [$2\theta \leq 52.034°$] and 742 parameters.

Refinement details for 7^{Tn}-Fc: The asymmetric unit contains one benzene molecule (RESI 41 and 42 BENZ), which was modelled as twofold disordered by rotation in a 54:46 ratio using AFIX 66 and SIMU restraints. Both thienyl groups were modelled as twofold flip disordered with the exception of the boron-bound carbon atoms C40 and C50. The disorder of RESI 11 and 12 THI1 was refined to a 89:11 ratio. The disorder of RESI 21 and 22 THI1 was refined to a 84:16 ratio. ADPs within these disorders were restrained with SIMU 0.005. The Cp group (RESI 31 and 32 CP) was modelled as twofold disordered by rotation in a 58:42 ratio. ADPs within this disorder were restrained with SIMU 0.01.

Crystal data for 7^{Tn}-Fc: $C_{30}H_{32}B_2FeN_4S_2\cdot C_6H_6$, $M_r = 668.29$, orange block, $0.23\times0.23\times0.098$ mm^3, triclinic space group $P\bar{1}$, $a = 8.3590(5)$ Å, $b = 9.7472(6)$ Å, $c = 20.9676(13)$ Å, $\alpha = 87.181(2)°$, $\beta = 80.992(2)°$, $\gamma = 76.775(2)°$, $V = 1642.40(17)$ Å^3, $Z = 2$, $\rho_{calcd} = 1.351$ g·cm^{-3}, $\mu = 0.619$ mm^{-1}, $F(000) = 700$, $T = 100(2)$ K, $R_1 = 0.0572$, $wR2 = 0.0960$, 6479 independent reflections [$2\theta \leq 52.044°$] and 546 parameters.

Crystal data for 8-Bu-D: $C_{27}H_{41}B_2P$, $M_r = 418.19$, colourless plate, $0.227\times0.197\times0.108$ mm^3, triclinic space group $P\bar{1}$, $a = 8.490(4)$ Å, $b = 12.424(9)$ Å, $c = 12.896(6)$ Å, $\alpha = 85.23(2)°$, $\beta = 89.53(3)°$, $\gamma = 73.32(2)°$, $V = 1298.4(13)$ Å^3, $Z = 2$, $\rho_{calcd} = 1.070$ g·cm^{-3}, $\mu = 0.117$ mm^{-1}, $F(000) = 456$, $T = 103(2)$ K, $R_1 = 0.0738$, $wR2 = 0.1442$, 5120 independent reflections [$2\theta \leq 52.036°$] and 281 parameters.

Crystal data for 8-TMS-C: $C_{26}H_{41}B_2PSi$, $M_r = 434.27$, colourless block, $0.244\times0.181\times0.074$ mm^3, monoclinic space group $P2_1/c$, $a = 14.7281(2)$ Å, $b = 9.22097(9)$ Å,

$c = 20.6828(3)$ Å, β = 110.0801(16)°, $V = 2638.14(6)$ Å^3, $Z = 4$, $\rho_{calcd} = 1.093$ g·cm^{-3}, $\mu = 1.406$ mm^{-1}, $F(000) = 944$, $T = 99.99(10)$ K, $R_1 = 0.0395$, $wR2 = 0.0431$, 5445 independent reflections [$2\theta \leq 68.13°$] and 283 parameters.

Crystal data for 8-Tol-C: $C_{30}H_{39}B_2P$, $M_r = 452.20$, yellow block, 0.479×0.193×0.135 mm^3, triclinic space group $P\ \bar{1}$, $a = 9.07205(19)$ Å, $b = 11.8014(2)$ Å, $c = 13.9051(3)$ Å, α = 77.8582(17)°, β = 79.2238(17)°, γ = 71.2414(19)°, $V = 1366.61(5)$ Å^3, $Z = 2$, $\rho_{calcd} = 1.099$ g·cm^{-3}, $\mu = 0.978$ mm^{-1}, $F(000) = 488$, $T = 180.00(10)$ K, $R_1 = 0.0517$, $wR2 = 0.1525$, 5325 independent reflections [$2\theta \leq 68.13°$] and 308 parameters

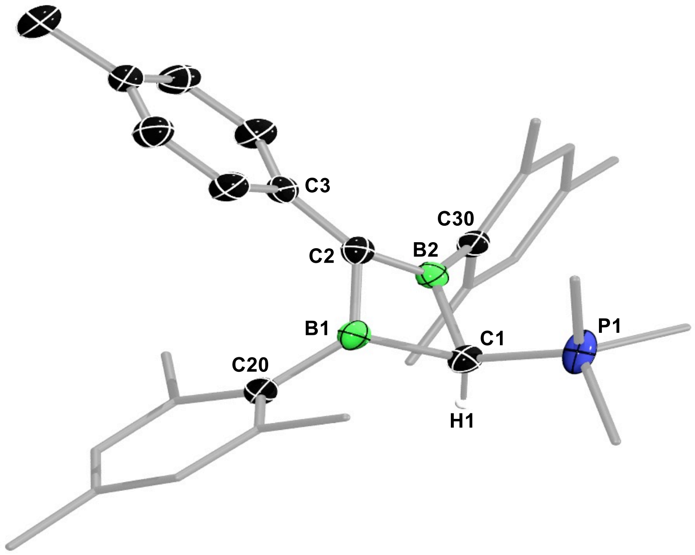

Figure S54. Crystallographically-derived molecular structures of **8-Tol-C**. Atomic displacement ellipsoids set at 50% probability. Ellipsoids of ligand periphery and hydrogen atoms omitted for clarity, except for alkene and boron-bound hydrogens. Selected bond lengths (Å) and angles (°): B1–C1 1.623(2), C1–B2 1.657(2), B2–C2 1.483(2), C2–B1 1.489(2), B1···B2 1.941(3), C1–P1 1.7352(17), B1-C1-B2 72.58(11), C1-B2-C2 97.80(13), B1-C2-B2 81.57(13), C2-B1-C1 99.06(14), Σ(∠B1) 358.26(15), Σ(∠B2) 359.96(14), Σ(∠C2) 352.71(14), torsion angles (C1-B1-C20-C21) – 114.7(2), (C1-B2-C30-C31) – 122.11(17).

Crystal data for 8-Fc-C: $C_{33}H_{41}B_2FeP$, $M_r = 546.10$, red needle, 0.285× 0.044× 0.040 mm^3, monoclinic space group $P\ 2_1/n$, $a = 15.7588(3)$ Å, $b = 8.14753(17)$ Å, $c = 23.2953(4)$ Å, $\beta = 102.6160(16)°$, $V = 2918.78(9)$ Å^3, $Z = 4$, $\rho_{calcd} = 1.243$ g·cm^{-3}, $\mu = 4.791$ mm^{-1},

$F(000) = 1160$, $T = 100$ K, $R_1 = 0.0499$, $wR2 = 0.1239$, 5733 independent reflections [$2\theta \leq 72.127°$] and 343 parameters.

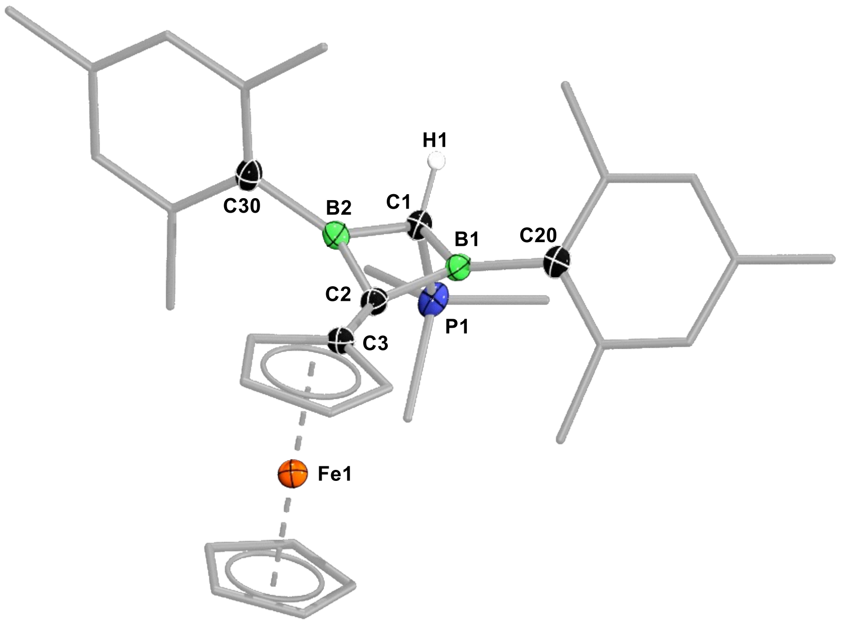

Figure S55. Crystallographically-derived molecular structures of **8-Fc-C**. Atomic displacement ellipsoids set at 50% probability. Ellipsoids of ligand periphery and hydrogen atoms omitted for clarity, except for alkene and boron-bound hydrogens. Selected bond lengths (Å) and angles (°): B1–C1 1.660(3), C1–B2 1.627(3), B2–C2 1.487(3), C2–B1 1.484(3), B1···B2 1.969(3), C1–P1 1.740(2), B1-C1-B2 73.59(13), C1-B2-C2 99.02(15), B1-C2-B2 82.98(15), C2-B1-C1 97.70(15), Σ(∠B1) 359.93(17), Σ(∠B2) 359.21(17), Σ(∠C2) 354.01(17), torsion angles (C1-B1-C20-C21) – 124.2(2), (C1-B2-C30-C31) 99.6(2).

Crystal data for 9-Tol: $C_{30}H_{39}B_2P$, $M_r = 452.20$, colourless block, 0.237×0.124×0.092 mm^3, triclinic space group $P\bar{1}$, $a = 10.2300(4)$ Å, $b = 11.4470(5)$ Å, $c = 11.7418(5)$ Å, $\alpha = 88.516(2)°$, $\beta = 87.444(2)°$, $\gamma = 85.144(2)°$, $V = 1368.36(10)$ Å^3, $Z = 2$, $\rho_{calcd} = 1.098$ g·cm^{-3}, $\mu = 0.116$ mm^{-1}, $F(000) = 488$, $T = 100(2)$ K, $R_1 = 0.0462$, $wR2 = 0.0997$, 5280 independent reflections [$2\theta \leq 52.044°$] and 311 parameters.

Refinement details for 11-TMS: The Platon program Squeeze[9] was employed to deal with the electron density of highly disordered co-crystallised solvent. A total of 276 electrons per

unit cell, i.e. ca. 67 electrons per asymmetric unit, were squeezed, which corresponds to ca. 1.5 molecules of Et_2O.

Crystal data for 11-TMS: $C_{27}H_{47}B_2BrP_2Si$·[+ squeezed $(Et_2O)_{1.5}$], M_r = 563.20, colourless needle, 0.12×0.10×0.10 mm^3, orthorhombic space group *Pbca*, a = 17.197(5) Å, b = 13.698(4) Å, c = 29.410(12) Å, V = 6928(4) Å^3, Z = 8, ρ_{calcd} = 1.080 g·cm^{-3}, μ = 1.326 mm^{-1}, $F(000)$ = 2384, T = 100(2) K, R_1 = 0.0568, $wR2$ = 0.1029, 6816 independent reflections [$2\theta \leq 52.044°$] and 304 parameters.

Computational details

Gas-phase theoretical calculations were performed using the Gaussian16 program.[10] Reaction mechanisms described herein were developed by exploring the potential energy surface with the hybrid, range-separated density-functional ω-B97XD,[11] which considers dispersion interactions through a range separation (22% for short range and 100% Hartree-Fock for long range). The electronic configurations of each atom were described with the split-valence basis set def2-svp.[12] Geometry optimisations were carried out without symmetry constraints, and the stationary points characterised by analytical frequency calculations, *i.e.* energy minima (reactants, intermediates and products) must exhibit only positive harmonic frequencies, whereas each energy maximum (transition state) exhibits one and only one negative frequency. From these last calculations, zero-point energy (ZPE) and thermal and entropy corrections were obtained, which were added to the electronic energy to express the calculated values as Gibbs free energies. The electronic energy was improved later by executing single-point calculations upon the optimised gas-phase geometries using the triple-zeta valence basis set def2-tzvpp.[12] Single-point calculations were also performed to include solvent effects through the PCM model using the SMD parameters according to Truhlar's model[13] with benzene as solvent (ε = 2.2706). As a result, the composite level of theory can be expressed as follows: (SMD:benzene)ω-B97XD/def2-tzvpp//ω-B97XD/def2-svp. Visualisation of optimised geometries and drawing of structures were done using the CYLview program.[14] Model systems of the reactants were used to explore the potential energy surface without drastically losing important steric effects, as follows:

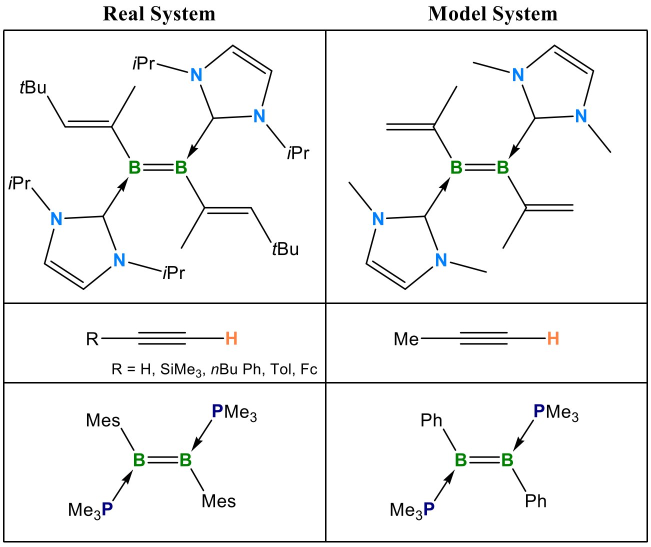

Figure S56. Real and model systems employed for the mechanistic calculations.

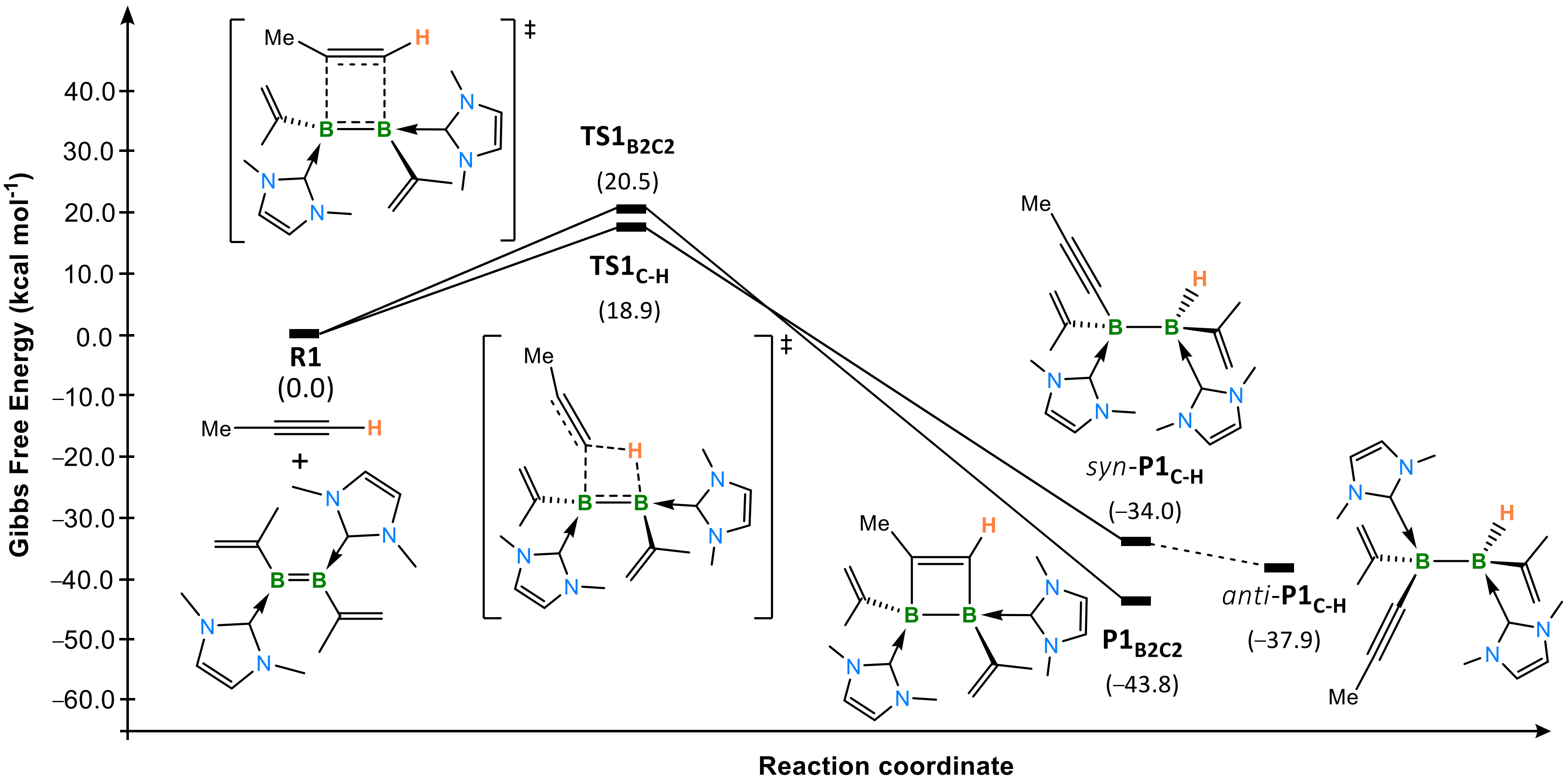

Figure S57. Energy profiles for the [2+2]-cycloaddition and 1,2-addition of the C–H bond of propyne to the model doubly IMe-stabilised 1,2-bis(1-methylvinyl)diborene **R1** in benzene at room temperature, calculated at the (SMD:benzene)ω-B97XD/def2-tzvpp//ω-B97XD/def2-svp level of theory. Energies in parentheses in kcal mol^{-1}.

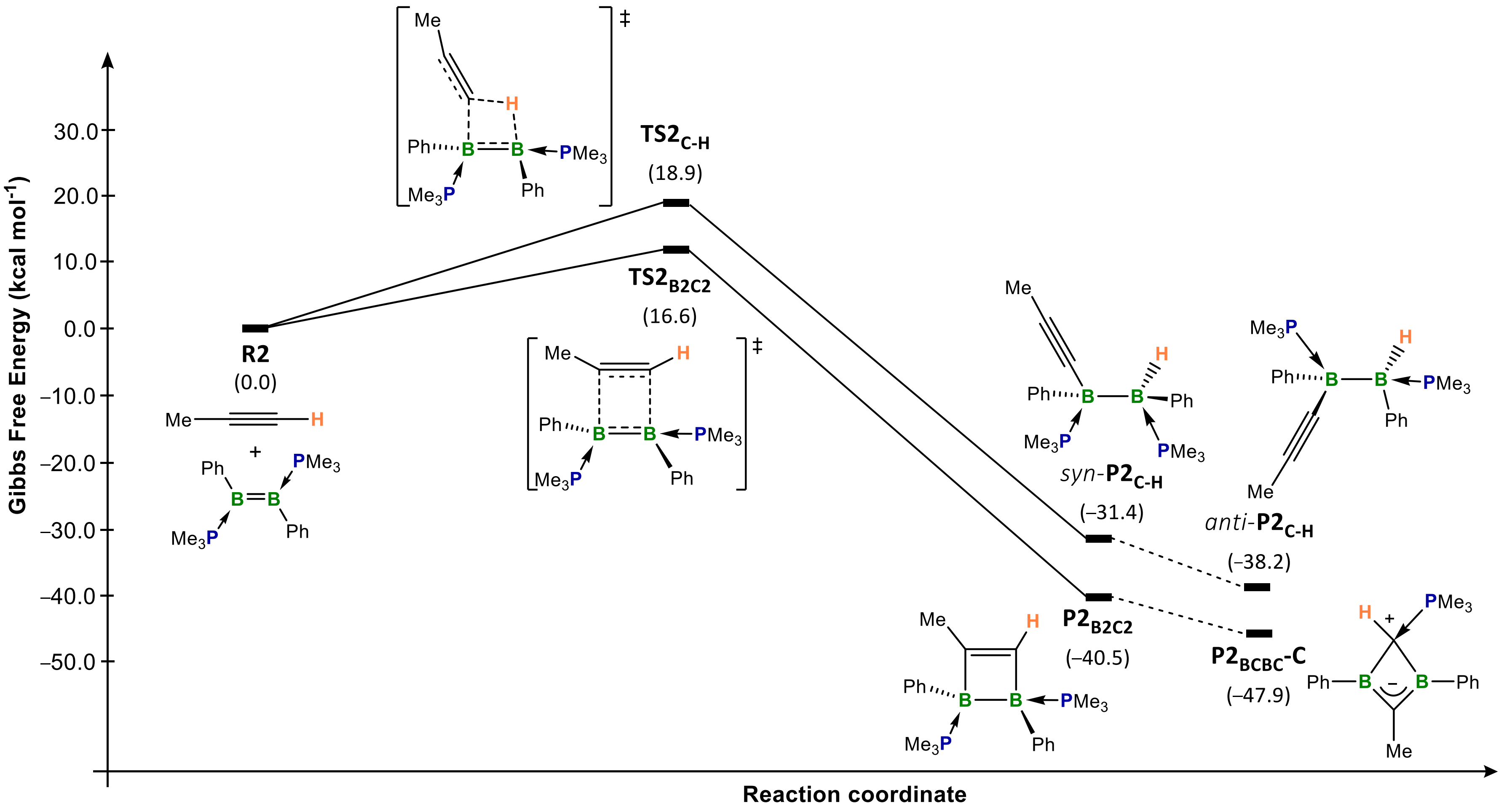

Figure S58. Energy profiles for the [2+2]-cycloaddition and 1,2-addition of the C–H bond of propyne to the model doubly PMe_3-stabilised 1,2-diphenyldiborene **R2** in benzene at room temperature, calculated at the (SMD:benzene)ω-B97XD/def2-tzvpp//ω-B97XD/def2-svp level of theory. Energies in parentheses in kcal mol^{-1}.

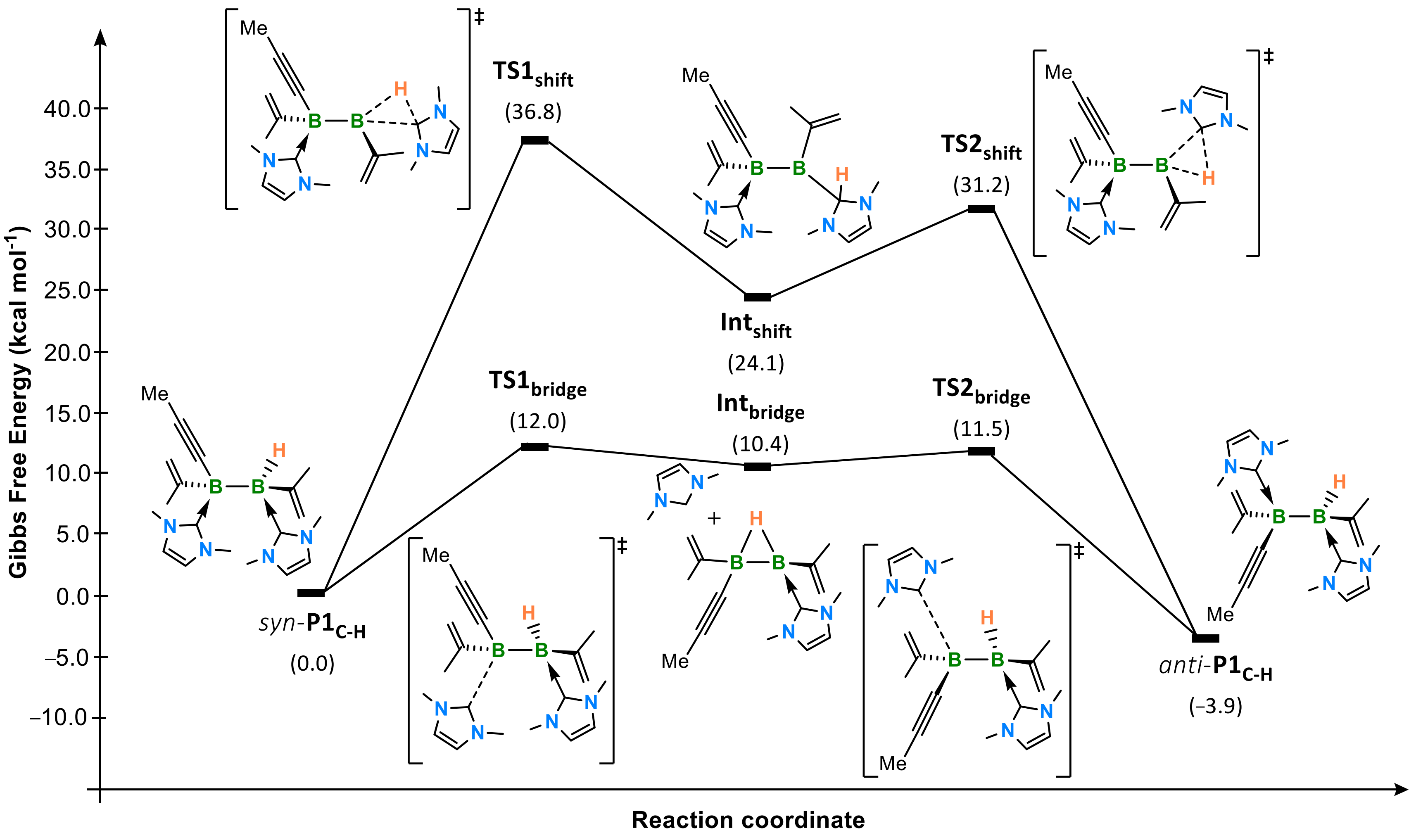

Figure S59. Explored reaction mechanisms for the *syn*-to-*anti* isomerisation of the model hydroalkynylation product *syn*-**P1**$_{\mathbf{C\text{-}H}}$ calculated at the (SMD:benzene)ω-B97XD/def2-tzvpp//ω-B97XD/def2-svp level. At 25 °C only the decoordination-coordination mechanism of the NHC is operational. Other tested routes gave higher energy barriers.

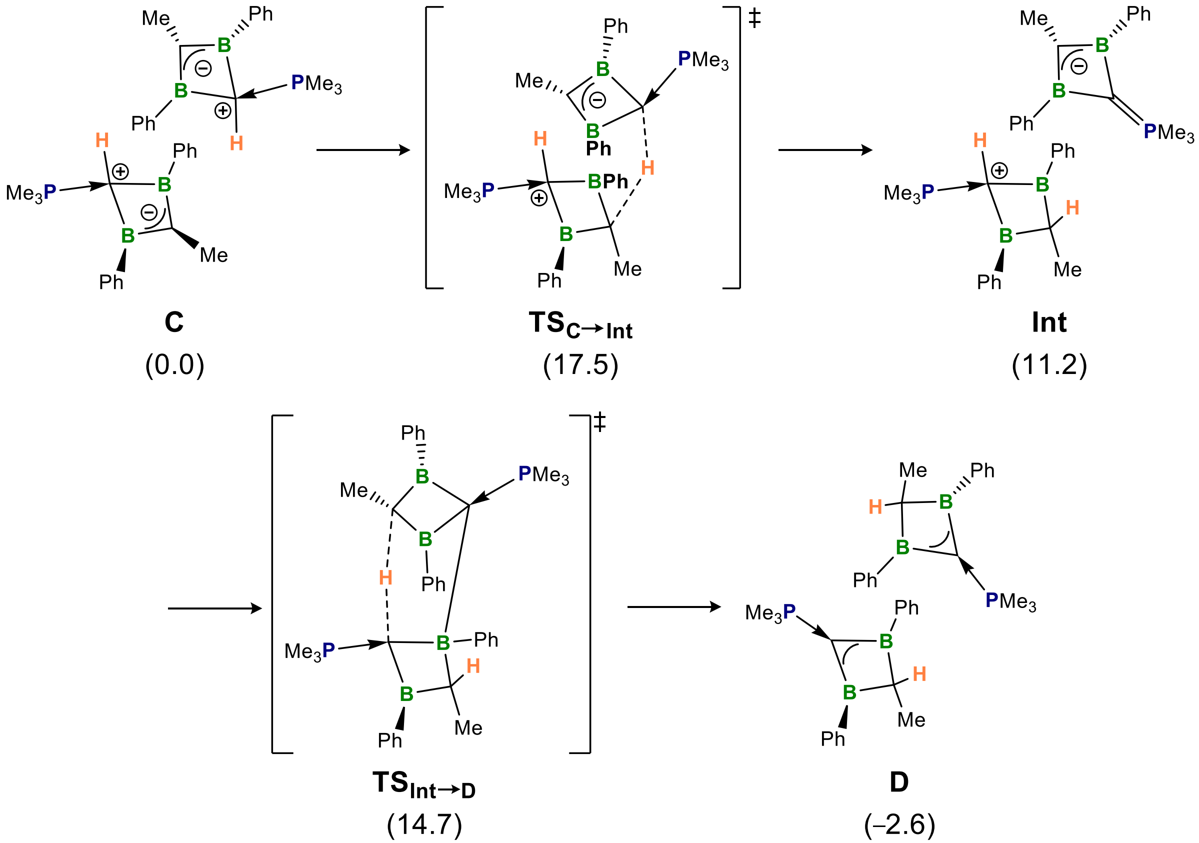

Figure S60. Proposed reaction mechanism for the **C**→**D** isomerisation calculated at the ω-B97XD/def2-svp level. This intermolecular mechanism was sketched as an initial exploration of the potential energy surface and was not adjusted to the full level of theory, as it was not the main mechanistic focus.

Table S1. Comparison of energy differences between $TS_{C\text{-}H}$ and TS_{B2C2} transition states calculated by using ω-B97XD with different basis sets.

Basis set	R1	R2
def2-tzvpp	1.6	2.3
6-311+G(d,p)	-1.1	0.2
cc-pVTZ	3.0	3.4
TZVP	1.8	2.2
Sapporo-TZP-2012[15]	3.3	2.5

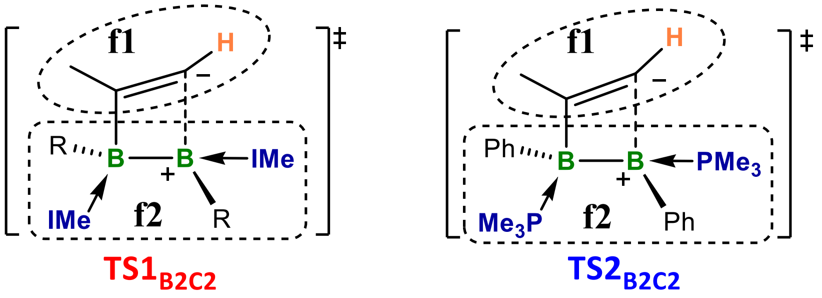

	$TS1_{B2C2}$
E_{def}(f1)	61.2
E_{def}(f2)	28.5
E_{int}	-69.7
E_{TS}	20.0

	$TS2_{B2C2}$
E_{def}(f1)	61.5
E_{def}(f2)	23.5
E_{int}	-69.4
E_{TS}	15.6

Figure S61. Energy decomposition analysis of key transition states used to explain the experimentally-observed selectivity. Displayed electronic energies are in kcal·mol^{-1} and were calculated at the ω-B97XD/def2-svp level.

Because our DFT calculations show that NHC-diborenes prefer 1,2-addition whereas phosphine-diborene prefers cycloaddition, in agreement with our experiments, we decided to seek more insight to explain the origin of this selectivity. Since the donor ligand affects the B-B double bond, we first investigated the B-B bond distance in the doubly IMe-stabilised 1,2-bis(1-methylvinyl)diborene **R1** and the doubly PMe_3-stabilised 1,2-diphenyldiborene **R2**: 1.6072 and 1.5743 Å, respectively. Both bond distances increase by almost the same amount upon [2+2] cycloaddition. Moreover, as shown in Figure 5, $\mathbf{TS1_{C\text{-}H}}$ and $\mathbf{TS2_{C\text{-}H}}$ for 1,2-addition are energetically the same. Thus, the source of selectivity is found in the energy difference between $\mathbf{TS1_{B2C2}}$ and $\mathbf{TS2_{B2C2}}$ (ca. 4 kcal·mol^{-1}) for the [2+2] cycloaddition.

Therefore, we analysed both transition states using the deformation-interaction model in which,

$$E_{TS} = \sum_{i=f1}^{f2} E_{def}\,(i) + E_{int} \qquad (1)$$

where the energy of the transition state (E_{TS}) is decomposed into the sum of the deformation energies (E_{def}) of each fragment (f1 stands for methylacetylene and f2 is the diborene **R1**/**R2**), defined as the energy required to bring the isolated reactants to their deformed shape as in the transition state geometry, and the interaction energy (E_{int}) defined as the energy difference between the transition state (total energy) and the sum of energies of each isolated fragment in the transition state geometry.

We can see that the interaction energies and deformation energies of methylacetylene do not vary significantly, but the diborene **R1**/**R2** deformation energies do. This means that the doubly IMe-stabilised 1,2-bis(1-methylvinyl)diborene **R1** significantly distorts compared to diborene **R2** and the associated energy barrier of the former increases sizeably. This is because the IMe ligands are more coplanar with the B-B double bond initially and must bend perpendicularly when the [2+2] cycloaddition takes place.

Table S2. Cartesian coordinates of the optimised geometries for all the species involved in the calculated reaction mechanism (xyz format) at the ω-B97XD/def2-svp level. Hydrogen atoms in the pictures were omitted for clarity.

- Reaction of **R1** with propyne.

R1 + propyne

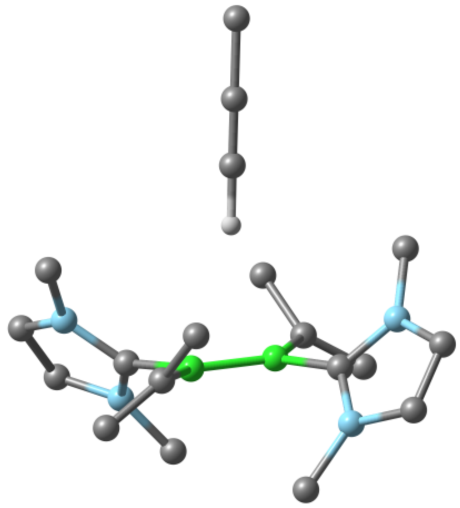

E(scf) = −1009.62380957 a.u.

B	0.630020	-0.685670	-0.522606
B	-0.501628	-0.603005	0.615623
C	-2.006934	-0.788146	0.197521
C	-4.220382	-0.429298	-0.062926
C	-3.908695	-1.562399	-0.739337
H	-4.531671	-2.233196	-1.323100
C	2.139375	-0.564493	-0.095626
C	4.286218	0.123141	0.023070
C	4.106523	-0.819644	0.980922
H	4.798139	-1.228662	1.711141
H	-5.168754	0.086352	0.055765
H	5.164940	0.702688	-0.244068
N	-3.053111	0.030900	0.505570
N	-2.556473	-1.776547	-0.564743
N	2.794600	-1.238957	0.892134
N	3.080563	0.267089	-0.627434
C	-1.843923	-2.913058	-1.108809
H	-1.563611	-2.740815	-2.157525

TS1$_{B2C2}$

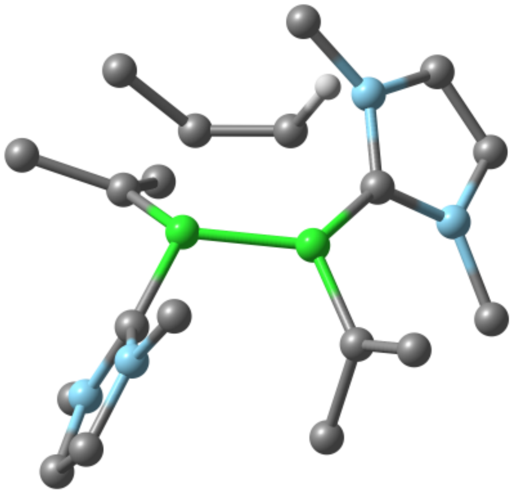

E(scf) = −1009.59192379 a.u.

ν_{min} = −242.3 cm^{-1}

B	-0.558684	0.693014	-0.166693
B	0.680371	-0.468591	0.476298
C	-0.299447	1.674599	-1.422629
C	0.206091	-1.965883	0.815597
C	-0.453011	1.720255	1.635500
C	0.419353	0.973369	2.321950
H	0.738868	1.168538	3.365140
C	-1.143636	2.976710	2.083770
H	-0.777318	3.857260	1.525206
H	-2.234429	2.941065	1.908984
H	-0.972405	3.175866	3.159409
H	2.416214	-3.082902	0.473541
C	2.841823	-2.662577	-0.443713
H	3.788348	-3.165410	-0.676105
H	2.140933	-2.831229	-1.271091
N	3.110587	-1.246477	-0.275597
C	2.224950	-0.276243	0.105405

H	-0.914538	-3.032120	-0.536287
H	-2.475638	-3.809018	-1.029407
C	-2.921978	1.259600	1.257436
H	-2.830631	1.051622	2.332213
H	-2.008943	1.777614	0.930505
H	-3.796811	1.893484	1.064289
C	2.810344	1.225845	-1.676231
H	2.763882	0.730266	-2.655512
H	1.834485	1.694174	-1.483299
H	3.596053	1.992067	-1.673011
C	2.219828	-2.269915	1.730090
H	1.901758	-1.861786	2.699780
H	1.322793	-2.651899	1.225001
H	2.959635	-3.068625	1.881258
C	-0.312588	-0.277821	2.173904
C	0.428592	-0.811996	-2.107841
C	1.207778	-1.597823	-2.879507
H	2.015192	-2.202613	-2.449535
H	1.072282	-1.677711	-3.966470
C	-1.000818	-0.921069	3.139652
H	-0.874836	-0.691822	4.206336
H	-1.719323	-1.713929	2.899492
C	-0.655528	-0.001659	-2.785764
H	-0.746652	-0.223408	-3.862678
H	-1.638767	-0.163498	-2.317240
H	-0.452689	1.076756	-2.672479
C	0.650638	0.811884	2.593756
H	0.750768	0.896981	3.689198
H	1.654066	0.655288	2.168251
H	0.316908	1.787611	2.201941
C	-0.418556	4.133406	-0.279498
C	-0.270102	2.933487	-0.209037
H	-0.144740	1.852370	-0.141642
C	-0.597545	5.579752	-0.366231
H	-1.622548	5.831297	-0.677599
H	-0.411429	6.059219	0.606505
N	2.950515	0.876146	0.063295
C	4.246242	0.625961	-0.323982
C	4.349872	-0.704452	-0.541944
H	5.191588	-1.310362	-0.862562
H	4.984238	1.417562	-0.406991
H	2.490727	2.262422	1.564547
C	2.523419	2.202436	0.470103
H	1.520107	2.406468	0.090246
H	3.237120	2.926443	0.056236
H	-1.616739	-0.583554	2.451054
C	-2.633648	-0.290437	2.177586
H	-3.366124	-0.932582	2.682943
H	-2.788879	0.753359	2.473289
N	-2.825686	-0.447402	0.743209
C	-2.003508	-0.020957	-0.251592
N	-2.613332	-0.441452	-1.397860
C	-3.788074	-1.106807	-1.118087
C	-3.918842	-1.108948	0.227978
H	-4.694383	-1.523011	0.864588
H	-4.421341	-1.521603	-1.896140
H	-1.033058	-0.675573	-2.757326
C	-2.078042	-0.348110	-2.743882
H	-2.117020	0.681213	-3.119663
H	-2.676030	-0.998447	-3.394396
C	-0.412297	-2.784722	-0.308444
H	-0.189564	-3.860143	-0.214490
H	-1.510652	-2.686638	-0.304066
H	-0.078163	-2.451578	-1.304806
C	-1.238681	2.851491	-1.566681
H	-2.294017	2.525779	-1.542217
H	-1.125872	3.535719	-0.711680
H	-1.075712	3.425671	-2.493957
C	0.697039	1.530437	-2.313564
H	1.405010	0.696387	-2.253725
H	0.851850	2.236211	-3.140612
C	0.248605	-2.497606	2.047631

H	0.096180	6.018540	-1.098860

Int1$_{B2C2}$

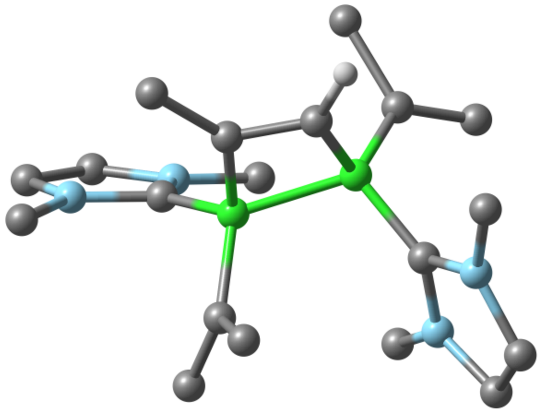

E(scf) = –1009.69965037 a.u.

B	0.871341	0.385895	0.823212
B	-0.637059	-0.537766	0.140675
C	-2.093317	0.119310	-0.214038
C	-4.320322	0.204165	-0.579648
C	-3.834985	1.458009	-0.732070
H	-4.331305	2.392172	-0.976702
C	2.178673	0.162576	-0.087864
C	4.167498	-0.563853	-0.857617
C	3.556144	0.100513	-1.868115
H	3.874922	0.292800	-2.887911
H	-5.330471	-0.185052	-0.665933
H	5.127516	-1.069327	-0.817204
N	-3.245654	-0.600257	-0.270553
N	-2.474029	1.385575	-0.512155
N	2.344621	0.540100	-1.378059
N	3.315113	-0.504419	0.226765
C	-1.606162	2.534691	-0.675685
H	-1.936997	3.350936	-0.019610
H	-0.588178	2.253555	-0.395033
H	-1.629816	2.871476	-1.722142
C	-3.362849	-2.040059	-0.138256
H	-2.361072	-2.473292	-0.104896
H	-3.912047	-2.299448	0.776486

H	-0.125490	-3.508546	2.261054
H	0.639928	-1.917322	2.888206

TS1$_{C\text{-}H}$

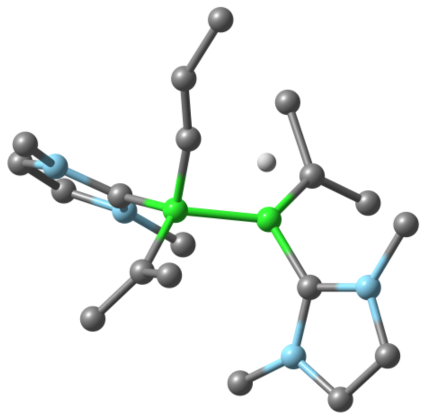

E(scf) = –1009.59796636 a.u.

ν_{min} = –870.2 cm^{-1}

B	-0.668444	-0.149903	-0.470045
B	0.813234	-0.326048	0.534069
C	2.192663	0.297088	0.042176
C	4.341514	0.477469	-0.593176
C	3.760141	1.689179	-0.774389
H	4.164448	2.623420	-1.151933
C	-2.053679	0.474107	0.129158
C	-4.247987	0.928930	0.292866
C	-3.618279	1.607886	1.282616
H	-4.004716	2.259895	2.059943
H	5.354892	0.140386	-0.789007
H	-5.299735	0.870658	0.029507
N	3.372895	-0.359927	-0.084876
N	2.447944	1.562745	-0.372043
N	-2.274275	1.317840	1.165545
N	-3.277804	0.244303	-0.400737
C	1.530139	2.677994	-0.261845
H	1.525543	3.252903	-1.196155
H	0.525396	2.279705	-0.102564
H	1.823219	3.324428	0.578813
C	3.587292	-1.744688	0.283990

H	-3.888054	-2.440879	-1.015713
C	3.609535	-1.058370	1.533854
H	3.429152	-0.299954	2.304713
H	2.966256	-1.923804	1.740665
H	4.663530	-1.361822	1.556714
C	1.385342	1.323617	-2.125737
H	1.626768	1.260061	-3.193594
H	0.389612	0.901317	-1.953652
H	1.408318	2.371534	-1.794680
C	-0.262710	-1.552834	-1.078164
C	0.780368	1.920311	1.333409
C	1.628353	2.912333	0.994903
H	2.483684	2.746438	0.328389
H	1.529049	3.930650	1.393335
C	-0.710042	-1.431232	-2.344633
H	-0.406471	-2.116461	-3.146389
H	-1.405969	-0.635267	-2.641591
C	-0.313053	2.225506	2.332750
H	-0.445652	3.306493	2.505827
H	-1.277624	1.788318	2.027433
H	-0.077209	1.750100	3.298328
C	0.654063	-2.711442	-0.762080
H	0.948023	-3.279089	-1.659779
H	1.565440	-2.375480	-0.246111
H	0.164742	-3.405894	-0.058050
C	-0.560117	-1.196434	1.639567
C	0.618612	-0.675769	2.020019
H	1.109276	-0.893213	2.988295
C	-1.424843	-2.120630	2.454526
H	-2.434081	-1.699925	2.610741
H	-1.566545	-3.100927	1.965571
H	-0.987689	-2.306105	3.450337

H	3.627972	-1.840347	1.377152
H	2.729114	-2.332221	-0.075721
H	4.524547	-2.093235	-0.166476
C	-3.537134	-0.567547	-1.581662
H	-3.086610	-0.084412	-2.458406
H	-3.080416	-1.559043	-1.448128
H	-4.622780	-0.652427	-1.710989
C	-1.273715	1.896894	2.041608
H	-1.603384	1.804197	3.084822
H	-0.332811	1.350395	1.925175
H	-1.126489	2.959639	1.800017
C	0.805401	-0.711086	2.081878
C	-0.386381	0.532966	-1.928094
C	-0.910947	1.704119	-2.333847
H	-1.596086	2.286235	-1.703420
H	-0.683107	2.141281	-3.314720
C	1.718383	-0.240146	2.957609
H	1.691441	-0.496030	4.024223
H	2.534589	0.424097	2.647043
C	0.517279	-0.220338	-2.873330
H	0.723424	0.339611	-3.799893
H	1.476745	-0.475236	-2.394849
H	0.057813	-1.186487	-3.135996
C	-0.300709	-1.600032	2.596903
H	-0.241683	-1.756094	3.686115
H	-1.291093	-1.178808	2.356976
H	-0.274237	-2.576822	2.091246
C	-1.195565	-2.913923	-0.694725
C	-0.790712	-1.734904	-0.590975
H	0.734217	-1.864969	-0.254038
C	-0.882791	-4.342013	-0.558569
H	-1.479934	-4.808730	0.242070
H	-1.093141	-4.907785	-1.480543
H	0.188121	-4.490918	-0.304758

- Decoordination-coordination mechanism for the isomerisation of *syn*-**P1**$_{\mathbf{C\text{-}H}}$ to *anti*-**P1**$_{\mathbf{C\text{-}H}}$.

syn-**P1**$_{\mathbf{C\text{-}H}}$

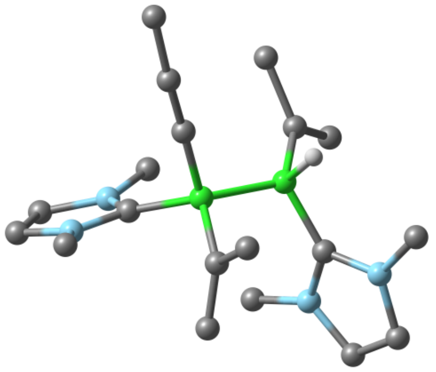

E(scf) = –1009.67511666 a.u.

B	-0.611456	0.444702	0.263648
B	0.898007	0.669945	-0.774035
C	2.191062	0.001040	-0.079731
C	4.114789	-0.208739	1.072678
C	3.632085	-1.455637	0.857976
H	4.017644	-2.428069	1.148072
C	-1.670818	-0.826246	0.210227
C	-3.457550	-2.070801	0.797428
C	-2.980149	-2.571134	-0.365813
H	-3.317372	-3.399847	-0.980793
H	5.009449	0.126998	1.587767
H	-4.299044	-2.377864	1.411166
N	3.227768	0.666885	0.481805
N	2.461859	-1.305889	0.144436
N	-1.891262	-1.796344	-0.710815
N	-2.653817	-1.004868	1.132578
C	1.615357	-2.399558	-0.273573
H	2.024666	-3.337707	0.120118
H	0.604041	-2.252450	0.123375
H	1.573216	-2.446986	-1.369192
C	3.405697	2.107527	0.470345
H	3.238165	2.494134	-0.540723
H	2.696588	2.589182	1.153929
H	4.432127	2.331653	0.787076
C	-2.927312	-0.135859	2.266406

TS1$_{\mathbf{bridge}}$

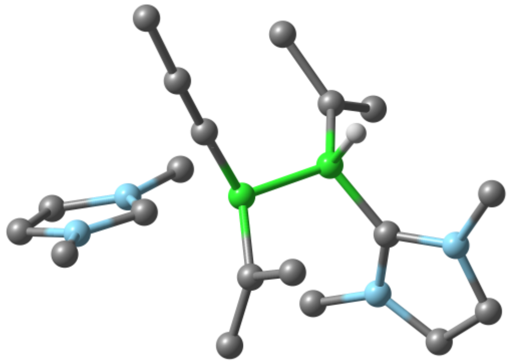

E(scf) = –1009.64983125 a.u.

ν_{min} = –165.0 cm^{-1}

B	0.378470	0.726126	-0.223868
B	-1.054766	0.522121	0.827160
C	-2.320054	-0.087370	0.038728
C	-4.352946	-0.278643	-0.907792
C	-3.670947	-1.420499	-1.169523
H	-3.955756	-2.308315	-1.725583
C	1.931047	-0.768642	-0.255622
C	3.976326	-1.564025	-0.783183
C	3.456299	-2.381763	0.163243
H	3.863078	-3.265191	0.646639
H	-5.354747	0.030764	-1.189574
H	4.932222	-1.591217	-1.298403
N	-3.512917	0.521591	-0.160351
N	-2.433136	-1.283178	-0.579467
N	2.207508	-1.875176	0.471650
N	3.033067	-0.585563	-1.021731
C	-1.403390	-2.297792	-0.577542
H	-1.487196	-2.903214	-1.488476
H	-0.429884	-1.797270	-0.565271
H	-1.498533	-2.936371	0.312272
C	-3.881546	1.819881	0.374633
H	-3.656682	1.855910	1.446716
H	-3.323284	2.620391	-0.126985
H	-4.957012	1.968108	0.216099

H	-2.220415	-0.330207	3.081184
H	-2.826865	0.908268	1.943777
H	-3.957484	-0.318711	2.596397
C	-1.205077	-1.965341	-1.976177
H	-1.874744	-1.689642	-2.802515
H	-0.327258	-1.315019	-2.008475
H	-0.895967	-3.013436	-2.093788
C	0.898117	0.465106	-2.380471
C	-0.021379	0.567513	1.784320
C	0.370114	-0.473464	2.542640
H	0.229947	-1.512769	2.218022
H	0.842587	-0.342634	3.524484
C	1.813900	-0.227686	-3.085736
H	1.788211	-0.284805	-4.181998
H	2.656381	-0.740115	-2.604262
C	0.152168	1.964793	2.323294
H	0.795646	2.006258	3.217905
H	0.553094	2.635455	1.547704
H	-0.831920	2.389497	2.584817
C	-0.193572	1.190065	-3.130982
H	-0.134276	1.044163	-4.222482
H	-1.191994	0.881652	-2.780392
H	-0.142328	2.268881	-2.913713
C	-2.352791	2.549915	-0.415131
C	-1.587518	1.650723	-0.107705
H	1.055380	1.879973	-0.613076
C	-3.223290	3.650405	-0.826359
H	-4.287209	3.365122	-0.793413
H	-3.097870	4.527133	-0.171032
H	-2.999984	3.974429	-1.855838
C	3.234714	0.504590	-1.963097
H	2.680747	0.319773	-2.891593
H	2.872779	1.437134	-1.513671
H	4.308720	0.589599	-2.172192
C	1.355621	-2.463555	1.485543
H	1.876173	-2.472891	2.453381
H	0.443141	-1.868789	1.592692
H	1.092423	-3.494962	1.208384
C	-0.907825	-0.128871	2.298504
C	-0.043622	0.855926	-1.774386
C	0.031264	-0.090639	-2.725315
H	0.504898	-1.061586	-2.540123
H	-0.362699	0.069127	-3.736985
C	-1.711054	-1.081657	2.810119
H	-1.585471	-1.467307	3.830575
H	-2.546992	-1.507998	2.241301
C	-0.675343	2.185477	-2.116992
H	-1.065143	2.219024	-3.146909
H	-1.496393	2.419309	-1.420582
H	0.059324	2.996961	-1.988663
C	0.205129	0.425544	3.153265
H	0.229963	-0.013433	4.164683
H	1.184225	0.268042	2.670460
H	0.106270	1.519064	3.242090
C	2.004184	2.780723	0.744641
C	1.311981	1.876137	0.309297
H	-1.332730	1.712300	0.996856
C	2.813015	3.863381	1.301169
H	3.879806	3.744558	1.052214
H	2.491108	4.841580	0.910223
H	2.730342	3.902720	2.399108

Int$_{bridge}$

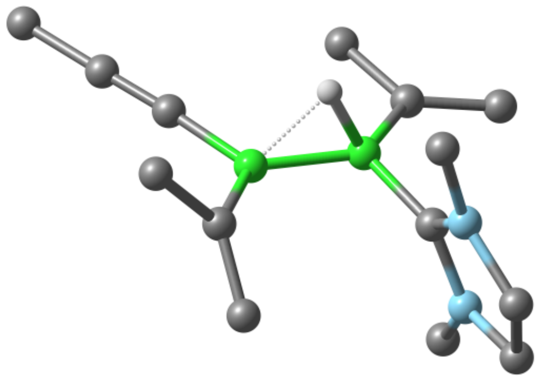

E(scf) = −705.135121293 a.u.

B	-1.121214	0.496409	0.140306
B	-0.049968	-0.675038	-0.336288
C	1.460933	-0.233431	-0.152132
C	3.445928	0.800370	-0.285945
C	3.459705	0.032153	0.831138
H	4.231786	-0.120205	1.578968
H	4.201615	1.457193	-0.705349
N	2.216947	0.610317	-0.885629
N	2.233684	-0.596692	0.896416
C	1.783341	-1.460659	1.970634
H	2.430049	-1.311296	2.843526
H	0.747320	-1.195006	2.223598
H	1.801682	-2.512299	1.655742
C	1.761179	1.307922	-2.070679
H	1.147577	0.631449	-2.677833
H	1.157109	2.180304	-1.784784
H	2.632830	1.633262	-2.651990
C	-0.310630	-2.232800	-0.583415
C	-0.672770	2.017611	0.382270
C	0.286133	2.337571	1.265173
H	0.828837	1.571031	1.827640
H	0.546965	3.379322	1.491641
C	0.693364	-3.098243	-0.813276
H	0.514675	-4.168197	-0.975503
H	1.740825	-2.776946	-0.864898
C	-1.423509	3.100732	-0.354013

TS2$_{bridge}$

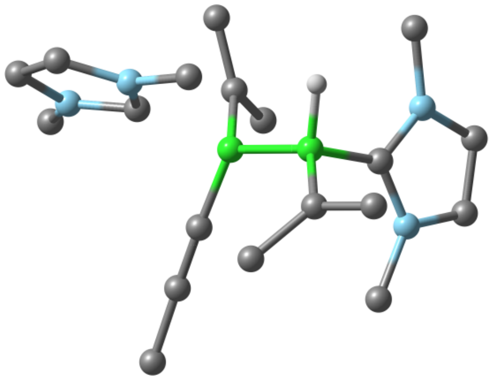

E(scf) = −1009.65686178 a.u.

ν_{min} = −147.3 cm^{-1}

B	-0.300879	0.302962	0.338486
B	0.755156	-0.912797	-0.422421
C	2.242908	-0.546645	0.049533
C	4.247853	0.454767	0.256195
C	4.135968	-0.445361	1.263174
H	4.820031	-0.701136	2.066421
C	-2.339611	-0.356932	0.141988
C	-4.575992	-0.277200	0.404803
C	-4.280163	-1.440933	-0.224614
H	-4.921027	-2.242540	-0.580095
H	5.050389	1.139963	0.001039
H	-5.528997	0.140933	0.716098
N	3.080764	0.379720	-0.475169
N	2.905513	-1.053397	1.117949
N	-2.905646	-1.469939	-0.370986
N	-3.375148	0.370402	0.615419
C	2.397835	-2.094703	1.994070
H	3.152478	-2.296728	2.764394
H	1.462102	-1.767388	2.462639
H	2.204121	-3.008337	1.417733
C	2.796608	1.171197	-1.658148
H	2.857465	0.542906	-2.556769
H	1.786387	1.591233	-1.578070
H	3.526599	1.988013	-1.716892
C	-3.239539	1.626138	1.332579

H	-1.057851	4.111702	-0.113263
H	-1.353000	2.957309	-1.446304
H	-2.497401	3.046570	-0.113862
C	-1.730167	-2.738963	-0.566828
H	-1.792208	-3.815445	-0.792400
H	-2.199825	-2.549858	0.411204
H	-2.347507	-2.191431	-1.296636
C	-3.859651	0.122837	0.257191
C	-2.652530	0.267557	0.178202
H	-0.589887	0.048830	-1.268905
C	-5.301297	-0.083905	0.347951
H	-5.674077	0.194415	1.345841
H	-5.839250	0.531720	-0.389663
H	-5.568948	-1.137151	0.170001

Free IMe

E(scf) = –304.479402766 a.u.

H	2.428646	-1.664564	0.000197
C	2.434783	-0.568744	0.000009
H	2.965429	-0.211042	-0.895405
H	2.965587	-0.210736	0.895209
N	1.059891	-0.122159	0.000050
C	0.000047	-0.977690	-0.000047
N	-1.059854	-0.122197	-0.000074
C	-0.677957	1.209127	-0.000041
H	-1.387492	2.032704	0.000149
C	0.677790	1.209138	0.000035
H	1.387285	2.032726	-0.000126
H	-2.428557	-1.664655	-0.000854
C	-2.434717	-0.568832	0.000040
H	-2.965116	-0.211708	0.895824
H	-2.965716	-0.210236	-0.894798

H	-2.943956	1.436016	2.373136
H	-2.463601	2.233313	0.852058
H	-4.198829	2.158704	1.303123
C	-2.197299	-2.564949	-1.009289
H	-2.378752	-2.559402	-2.093418
H	-1.124687	-2.449950	-0.823508
H	-2.541706	-3.518766	-0.586565
C	0.698592	-1.137558	-2.049782
C	-0.139540	0.407436	1.944147
C	-0.746567	-0.391871	2.837464
H	-1.452624	-1.168329	2.522578
H	-0.560071	-0.308926	3.916204
C	1.472594	-2.062514	-2.642072
H	1.441376	-2.256126	-3.723171
H	2.170360	-2.679324	-2.062379
C	0.821699	1.465720	2.435971
H	0.997145	1.407933	3.522441
H	1.797463	1.395310	1.925718
H	0.436054	2.469860	2.194731
C	-0.245034	-0.330359	-2.906669
H	-0.263740	-0.667963	-3.956532
H	-1.271696	-0.361894	-2.507422
H	0.022490	0.738055	-2.886988
C	-0.430907	2.817373	-0.881222
C	-0.404411	1.716412	-0.354293
H	0.502579	-1.982615	0.139760
C	-0.450485	4.117702	-1.548802
H	-1.400354	4.273355	-2.084274
H	-0.334149	4.946213	-0.832392
H	0.360331	4.202971	-2.290623

anti-**P1**$_{\mathbf{C\text{-}H}}$

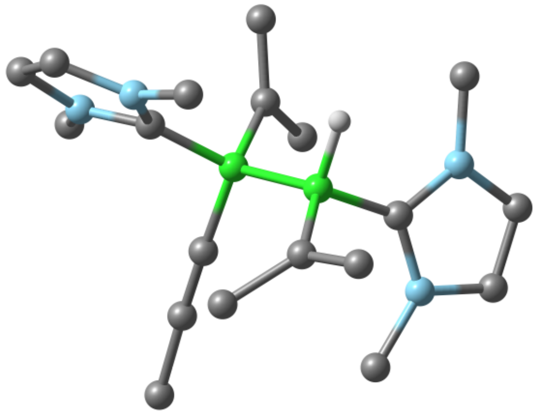

E(scf) = –1009.68577468 a.u.

B	-0.569426	-0.182903	-0.314571
B	0.654965	0.968650	0.380929
C	2.119677	0.513451	-0.098028
C	4.119570	-0.507056	-0.275927
C	4.001932	0.342678	-1.324253
H	4.677332	0.552848	-2.147775
C	-2.148871	0.260557	-0.121357
C	-4.392375	0.095428	-0.268132
C	-4.106820	1.298634	0.282574
H	-4.755079	2.099482	0.624541
H	4.919893	-1.185070	0.004061
H	-5.343594	-0.368374	-0.511025
N	2.960612	-0.389520	0.462769
N	2.776705	0.963421	-1.197056
N	-2.732170	1.386466	0.358229
N	-3.188386	-0.523768	-0.509579
C	2.289111	1.987003	-2.105039
H	3.003566	2.082271	-2.932282
H	1.303977	1.706160	-2.493815
H	2.203425	2.947073	-1.579030
C	2.719575	-1.090537	1.710021
H	2.865877	-0.411149	2.560619
H	1.691321	-1.467827	1.714774
H	3.415913	-1.935861	1.776764
C	-3.072692	-1.803890	-1.187771

H	-2.619812	-1.652069	-2.176104
H	-2.431834	-2.475343	-0.605550
H	-4.076686	-2.232084	-1.294066
C	-2.080843	2.569083	0.899233
H	-2.120533	2.559551	1.996978
H	-1.035616	2.585027	0.579922
H	-2.599715	3.459136	0.519760
C	0.647546	1.238216	2.007113
C	-0.338734	-0.306239	-1.933509
C	-0.920306	0.510887	-2.831265
H	-1.613817	1.303744	-2.524862
H	-0.719400	0.438906	-3.908279
C	1.412417	2.204426	2.542348
H	1.419210	2.427073	3.618459
H	2.064498	2.826545	1.917251
C	0.591241	-1.386996	-2.428889
H	0.822560	-1.292978	-3.502795
H	1.537974	-1.398874	-1.865175
H	0.142176	-2.378767	-2.253478
C	-0.236088	0.433102	2.930003
H	-0.193418	0.783924	3.974802
H	-1.288023	0.456633	2.600208
H	0.029735	-0.634871	2.907863
C	-0.277787	-2.709745	0.900612
C	-0.423227	-1.616198	0.375999
H	0.507256	2.054700	-0.190436
C	-0.061457	-3.996287	1.561928
H	-0.911531	-4.263826	2.209450
H	0.069052	-4.812907	0.833964
H	0.839061	-3.977104	2.198964

- Reaction of **R2** with propyne

R2 + propyne

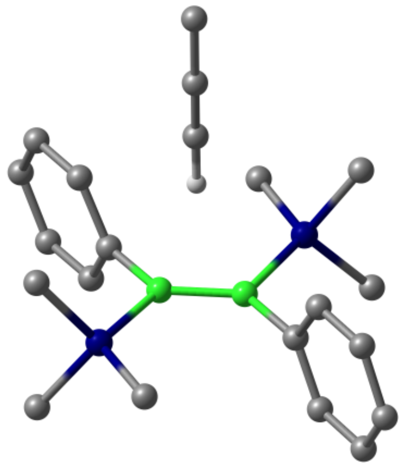

E(scf) = –1550.78016386 a.u.

B	0.654899	-0.142309	-0.708059
B	-0.654886	-0.738600	-0.070604
C	2.210724	-0.407184	-0.442990
C	2.959385	0.359401	0.470963
C	2.905640	-1.426826	-1.120836
C	4.310496	0.108346	0.714866
H	2.464539	1.164770	1.023233
C	4.256641	-1.686337	-0.885802
H	2.358929	-2.044690	-1.840151
C	4.966274	-0.920106	0.038596
H	4.854514	0.718146	1.441187
H	4.758706	-2.494219	-1.425075
H	6.023176	-1.121671	0.229041
C	-2.210781	-0.490602	-0.352217
C	-2.963394	0.463075	0.359970
C	-2.902140	-1.226389	-1.333602
C	-4.314622	0.689169	0.093181
H	-2.471606	1.062275	1.132891
C	-4.253104	-1.009641	-1.608524
H	-2.352494	-1.978388	-1.908370
C	-4.966653	-0.045427	-0.896744
H	-4.861370	1.447519	0.660012
H	-4.752242	-1.594817	-2.385693

TS2$_{B2C2}$

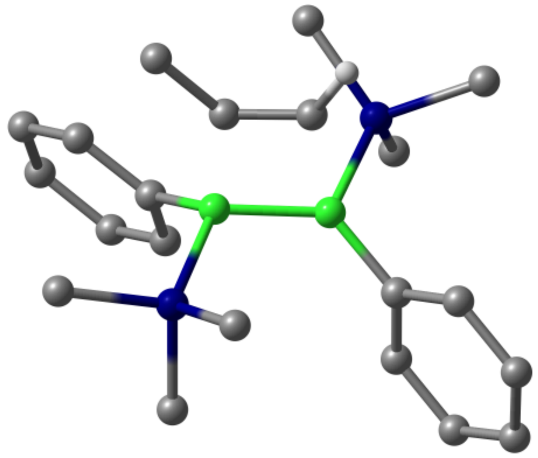

E(scf) = –1550.76527711 a.u.

ν_{min} = –202.8 cm^{-1}

B	0.772206	0.388196	0.325680
B	-0.806540	-0.391902	0.353270
C	2.003618	-0.105106	-0.573172
C	3.316453	-0.190055	-0.076552
C	1.814827	-0.482152	-1.916320
C	4.377401	-0.630982	-0.868764
H	3.503847	0.087943	0.964100
C	2.867956	-0.919635	-2.719883
H	0.807541	-0.433051	-2.344264
C	4.158416	-0.998724	-2.196207
H	5.383710	-0.690681	-0.445817
H	2.681028	-1.203524	-3.758959
H	4.986804	-1.346455	-2.817835
C	-2.194834	0.039300	-0.286951
C	-3.315722	0.363942	0.497686
C	-2.352494	0.109986	-1.683897
C	-4.527165	0.743827	-0.080561
H	-3.212075	0.325097	1.585583
C	-3.561716	0.485181	-2.270267
H	-1.503560	-0.130744	-2.332783
C	-4.656458	0.806281	-1.468176
H	-5.378130	0.995595	0.557690

H	-6.023620	0.130600	-1.110688
P	0.463175	1.208887	-2.036670
P	-0.462567	-1.977690	1.362105
C	-1.225405	1.707872	-2.491700
H	-1.192083	2.490789	-3.263245
H	-1.782479	0.836930	-2.861106
H	-1.747777	2.080792	-1.600647
C	1.264366	0.781953	-3.625398
H	0.750128	-0.087233	-4.059345
H	1.238986	1.620899	-4.336736
H	2.308480	0.500847	-3.424067
C	1.287205	2.774738	-1.570657
H	0.820466	3.165573	-0.654509
H	2.344891	2.564790	-1.355170
H	1.217353	3.524586	-2.372709
C	1.225317	-2.432461	1.864261
H	1.189642	-3.155316	2.692299
H	1.761889	-2.864562	1.009457
H	1.770099	-1.531590	2.176169
C	-1.251396	-1.395356	2.907539
H	-0.759835	-0.466261	3.231449
H	-2.307873	-1.171471	2.700455
H	-1.185426	-2.148532	3.706830
C	-1.297998	-3.576433	1.054130
H	-2.342437	-3.375809	0.773775
H	-0.804041	-4.077360	0.209493
H	-1.272485	-4.230043	1.938751
C	-0.003268	3.170579	2.797478
C	-0.009102	2.246285	2.014883
H	-0.012569	1.419172	1.304248
C	0.006564	4.282686	3.743320
H	0.855082	4.956018	3.550119
H	-0.918954	4.872904	3.667846
H	0.092716	3.917868	4.777749
H	-3.648974	0.529819	-3.358927
H	-5.604076	1.105378	-1.922109
P	0.562655	2.286937	0.152074
P	-0.670022	-2.290090	0.548164
C	-0.746772	3.017001	1.175482
H	-0.762702	4.110512	1.061890
H	-1.712330	2.593674	0.865220
H	-0.572452	2.733077	2.221327
C	0.161550	2.806486	-1.552690
H	-0.810758	2.375404	-1.831765
H	0.116255	3.901629	-1.643313
H	0.930485	2.413497	-2.233862
C	2.082024	3.215162	0.543553
H	2.328151	3.069701	1.603802
H	2.906380	2.811650	-0.062222
H	1.962368	4.288453	0.336602
C	0.850998	-2.920244	1.312966
H	0.859441	-4.019392	1.281350
H	1.713056	-2.522183	0.759062
H	0.907309	-2.558891	2.346298
C	-2.061802	-3.023409	1.463239
H	-2.023934	-2.656238	2.497331
H	-3.000868	-2.679708	1.005535
H	-2.021869	-4.122217	1.445954
C	-0.719947	-3.138914	-1.074928
H	-1.625919	-2.827440	-1.615337
H	0.161446	-2.833161	-1.657501
H	-0.720037	-4.233196	-0.963490
C	0.838881	0.280297	2.398891
C	-0.403868	-0.148130	2.662435
H	-0.808516	-0.344258	3.673535
C	1.938245	0.581312	3.375624
H	2.805760	-0.082932	3.217471
H	2.317312	1.613621	3.278373
H	1.599947	0.446301	4.419719

P2$_{B2C2}$

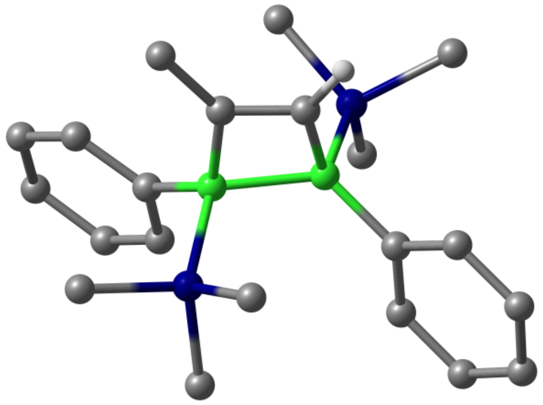

E(scf) = –1550.86817235 a.u.

B	-0.790957	-0.425566	0.522763
B	0.831074	0.390452	0.636454
C	-2.068516	0.031120	-0.352651
C	-3.355164	0.157321	0.202580
C	-1.954803	0.330330	-1.724385
C	-4.454101	0.568834	-0.554134
H	-3.500543	-0.067961	1.262611
C	-3.044463	0.735820	-2.494666
H	-0.971114	0.249642	-2.199656
C	-4.304717	0.861561	-1.909166
H	-5.435960	0.660755	-0.081994
H	-2.910068	0.960553	-3.556281
H	-5.162057	1.184729	-2.504266
C	2.209949	0.026810	-0.117321
C	3.372181	-0.294761	0.607163
C	2.327473	0.003185	-1.520550
C	4.572829	-0.626330	-0.023259
H	3.327097	-0.294617	1.700649
C	3.521413	-0.321807	-2.164582
H	1.446842	0.236418	-2.129347
C	4.654281	-0.641405	-1.415345
H	5.452610	-0.875104	0.576455
H	3.568149	-0.331591	-3.256954
H	5.591494	-0.901571	-1.912949
P	-0.461994	-2.285803	0.080139
P	0.552023	2.302324	0.425725

TS3

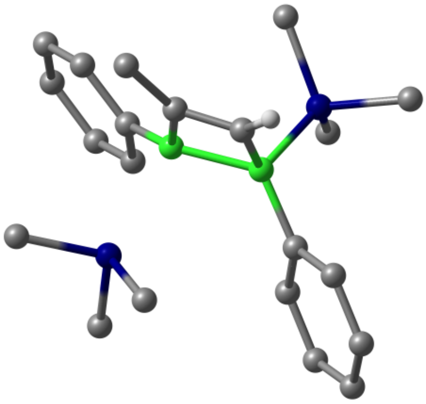

E(scf) = –1550.84683310 a.u.

ν_{min} = –133.7 cm^{-1}

B	-0.806212	-0.048459	0.624374
B	0.826168	0.665469	0.628798
C	-2.105614	0.108958	-0.287502
C	-3.379785	0.321479	0.266844
C	-2.029393	0.077994	-1.693674
C	-4.512767	0.496910	-0.531264
H	-3.488469	0.351688	1.354473
C	-3.152857	0.241233	-2.501648
H	-1.053644	-0.083811	-2.163913
C	-4.404336	0.455051	-1.920049
H	-5.487585	0.662791	-0.065147
H	-3.054288	0.206367	-3.589904
H	-5.288832	0.588846	-2.547361
C	2.132516	0.089603	-0.118047
C	3.331422	-0.147219	0.577963
C	2.135246	-0.232455	-1.488258
C	4.457938	-0.686084	-0.046025
H	3.375287	0.080141	1.647641
C	3.254855	-0.765644	-2.127258
H	1.218607	-0.077093	-2.066969
C	4.425154	-1.000424	-1.404707
H	5.368988	-0.864701	0.531514
H	3.212460	-1.006432	-3.193121
H	5.303647	-1.425331	-1.895964
P	-0.345860	-2.429024	0.265315

C	0.909710	-3.050596	0.996610	P	0.637381	2.535473	0.173120
H	0.988603	-4.120897	0.757911	C	1.126927	-3.024383	1.163285
H	1.844264	-2.537524	0.730143	H	1.187235	-4.123055	1.185477
H	0.739215	-2.913798	2.072768	H	2.024795	-2.610585	0.682283
C	-0.099574	-2.631909	-1.672177	H	1.087677	-2.629706	2.189253
H	0.830222	-2.116268	-1.949216	C	-0.129282	-3.058772	-1.438897
H	0.013534	-3.712126	-1.844201	H	0.768210	-2.597861	-1.874718
H	-0.922019	-2.247793	-2.291870	H	-0.022616	-4.154326	-1.459817
C	-1.906464	-3.349736	0.433887	H	-1.003127	-2.767014	-2.040075
H	-2.122461	-3.314316	1.510941	C	-1.718703	-3.454702	0.912299
H	-2.777205	-2.947700	-0.104479	H	-1.834815	-3.262405	1.988444
H	-1.734092	-4.392930	0.130716	H	-2.650825	-3.154098	0.411130
C	-0.966257	2.934289	1.204393	H	-1.549185	-4.531107	0.753782
H	-1.009798	4.030895	1.138045	C	-0.822273	3.308090	0.938294
H	-1.837391	2.494098	0.699324	H	-0.875946	4.380607	0.702899
H	-0.981859	2.618909	2.255949	H	-1.725240	2.800803	0.568597
C	1.904218	3.248674	1.209385	H	-0.768949	3.163080	2.025834
H	1.937171	2.991942	2.277820	C	2.048711	3.575520	0.687843
H	2.856320	2.940915	0.752900	H	2.165913	3.493463	1.778038
H	1.772708	4.334682	1.095403	H	2.960744	3.182106	0.215486
C	0.497354	2.961645	-1.274640	H	1.914526	4.631648	0.411466
H	1.418155	2.676980	-1.802737	C	0.459482	2.870034	-1.609212
H	-0.365960	2.527624	-1.797600	H	1.338540	2.474647	-2.137607
H	0.405891	4.057585	-1.267706	H	-0.438055	2.350615	-1.974451
C	-0.694371	-0.329343	2.144076	H	0.366816	3.947867	-1.806336
C	0.579671	0.122973	2.210950	C	-0.682189	0.033161	2.216519
H	1.156143	0.276306	3.141519	C	0.590880	0.502619	2.226915
C	-1.621199	-0.636352	3.286750	H	1.190286	0.692915	3.136533
H	-2.457616	0.083293	3.323957	C	-1.568729	-0.270672	3.390659
H	-2.083787	-1.633782	3.190450	H	-2.397090	0.455680	3.460229
H	-1.104200	-0.591540	4.260148	H	-2.041574	-1.262799	3.299251
				H	-1.019310	-0.236503	4.346199

Int3

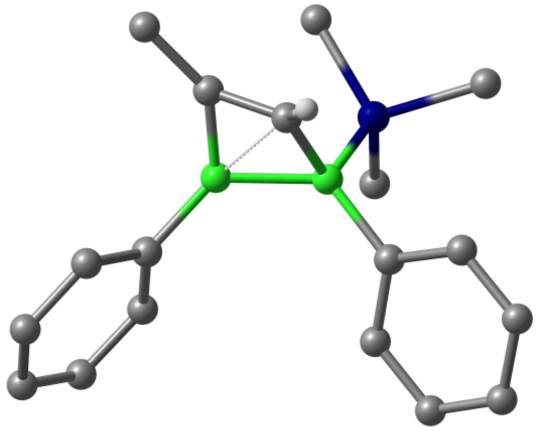

E(scf) = −1089.95612950 a.u.

B	0.973041	0.882723	-0.278396
B	-0.695563	0.423240	-0.397263
C	2.271563	0.055064	0.059209
C	3.320552	-0.098103	-0.863307
C	2.430485	-0.548424	1.317485
C	4.467508	-0.825850	-0.550198
H	3.231148	0.354301	-1.855447
C	3.582486	-1.260700	1.649141
H	1.629235	-0.455593	2.057440
C	4.604064	-1.405719	0.711246
H	5.262347	-0.938859	-1.291751
H	3.680498	-1.713869	2.638821
H	5.504093	-1.972448	0.961527
C	-1.301955	-1.059534	-0.487325
C	-2.333348	-1.369417	-1.392157
C	-0.858785	-2.099678	0.347079
C	-2.894747	-2.644356	-1.463766
H	-2.703939	-0.589216	-2.065686
C	-1.415520	-3.378067	0.288226
H	-0.041695	-1.906441	1.047347
C	-2.438331	-3.655144	-0.617818
H	-3.690969	-2.851915	-2.183478
H	-1.042105	-4.165500	0.947945
H	-2.874173	-4.655811	-0.669251
P	-1.759056	1.470639	0.799767

TS4

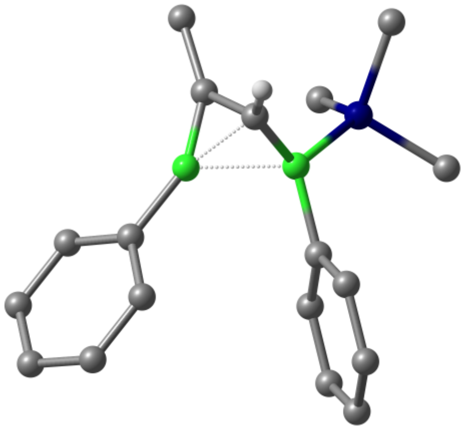

E(scf) = −1089.92878242 a.u.

$\nu_{min} = -150.2\ cm^{-1}$

B	0.453799	-1.395774	0.649329
B	-0.982375	-0.066216	0.577464
C	1.950200	-1.256606	0.247370
C	2.786414	-0.295678	0.838650
C	2.504087	-2.080924	-0.746826
C	4.119841	-0.163351	0.456716
H	2.376001	0.369657	1.602033
C	3.836063	-1.953001	-1.136689
H	1.874272	-2.837361	-1.223431
C	4.647830	-0.991844	-0.533610
H	4.749965	0.594028	0.929253
H	4.244853	-2.604989	-1.912779
H	5.692548	-0.887676	-0.836992
C	-0.180653	1.285397	0.268243
C	0.200059	2.125693	1.327214
C	0.193228	1.679308	-1.026512
C	0.913895	3.303016	1.106027
H	-0.060865	1.839007	2.350437
C	0.908230	2.853357	-1.259897
H	-0.053874	1.032535	-1.875704
C	1.270458	3.672423	-0.191098
H	1.196865	3.936406	1.950850
H	1.197672	3.123859	-2.278487
H	1.835104	4.590933	-0.367489

C	-1.286196	3.219641	0.949091
H	-1.951333	3.742477	1.651032
H	-0.246358	3.284364	1.295907
H	-1.345903	3.691843	-0.041226
C	-3.542768	1.498416	0.407066
H	-3.686346	1.987665	-0.567052
H	-3.896798	0.460385	0.325665
H	-4.126623	2.028218	1.173705
C	-1.692482	0.795353	2.489477
H	-1.973837	-0.267077	2.448992
H	-0.658949	0.866088	2.856371
H	-2.367695	1.330885	3.172049
C	0.843904	2.052201	-1.215950
C	-0.185299	1.252027	-1.662546
H	-0.405462	1.076521	-2.730299
C	1.628844	3.116376	-1.897122
H	1.346243	4.106462	-1.502549
H	2.706986	2.998709	-1.710445
H	1.450251	3.134895	-2.984823

P	-2.734766	-0.033802	-0.307000
C	-4.040921	-0.667739	0.793824
H	-5.020643	-0.669466	0.294709
H	-3.791022	-1.682694	1.126599
H	-4.078614	-0.023543	1.683203
C	-3.256260	1.676798	-0.664353
H	-3.172999	2.271104	0.256546
H	-2.576898	2.117281	-1.406772
H	-4.289957	1.707130	-1.037751
C	-2.965052	-0.903168	-1.897607
H	-2.242676	-0.501053	-2.622216
H	-2.752829	-1.972170	-1.761872
H	-3.985104	-0.774940	-2.289312
C	-0.783471	-2.118296	0.656321
C	-0.761786	-1.062256	1.707837
H	-0.847958	-1.168182	2.793942
C	-1.565426	-3.356563	0.396245
H	-1.356907	-4.076415	1.206617
H	-2.658069	-3.211471	0.389759
H	-1.274896	-3.834396	-0.551230

Int4

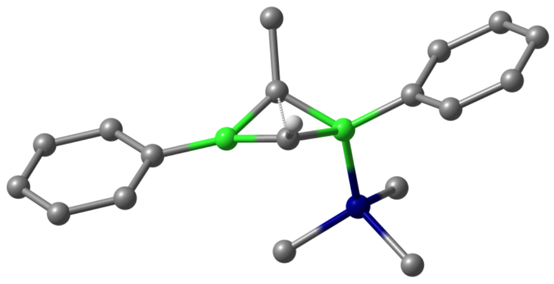

E(scf) = −1089.95646016 a.u.

B	-1.509761	-0.521823	-0.220992
B	0.871879	-0.129483	-0.116957
C	-3.061346	-0.487942	-0.203217
C	-3.773232	-0.722621	0.985986
C	-3.800998	-0.203569	-1.364203
C	-5.166013	-0.682672	1.016478
H	-3.217700	-0.941675	1.902216

TS5

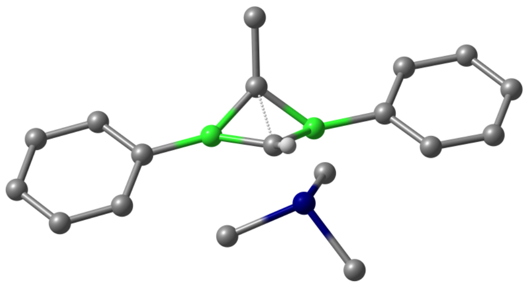

E(scf) = −1089.93843200 a.u.

$\nu_{min} = -162.8\ cm^{-1}$

B	1.452782	-0.878087	0.331659
B	-0.836853	-0.608997	0.262721
C	2.977587	-0.605297	0.254820
C	3.678695	-0.760250	-0.953780
C	3.697323	-0.168532	1.380486
C	5.044484	-0.496333	-1.036425

C	-5.193734	-0.159935	-1.342419
H	-3.267551	-0.016088	-2.300214
C	-5.877652	-0.399853	-0.149900
H	-5.700075	-0.870734	1.950945
H	-5.749965	0.060633	-2.256716
H	-6.969731	-0.364904	-0.129271
C	2.399785	-0.608949	-0.207258
C	3.135352	-0.937135	0.945122
C	3.080937	-0.673261	-1.434416
C	4.478485	-1.309448	0.880995
H	2.637255	-0.909435	1.919750
C	4.424135	-1.043777	-1.512075
H	2.540231	-0.433421	-2.355356
C	5.129031	-1.361773	-0.351501
H	5.019419	-1.564479	1.796012
H	4.923443	-1.089084	-2.483339
H	6.180722	-1.652761	-0.408064
P	0.855591	1.787340	0.246844
C	1.681170	2.734280	-1.070786
H	1.711135	3.811152	-0.851535
H	2.705324	2.350070	-1.184550
H	1.143132	2.563255	-2.014036
C	-0.807429	2.502293	0.405856
H	-1.362953	2.326267	-0.525974
H	-1.339893	1.991472	1.220693
H	-0.757625	3.580946	0.610421
C	1.735478	2.243274	1.775388
H	1.236035	1.762504	2.628675
H	2.763515	1.858053	1.714357
H	1.757189	3.331976	1.927190
C	-0.292970	-0.984206	0.533466
C	-0.287949	-0.625069	-1.080824
H	-0.120547	-1.294851	-1.934174
C	-0.087412	-2.300106	1.244043
H	0.070989	-2.127142	2.321648
H	-0.948869	-2.979435	1.142465
H	3.136495	-1.095221	-1.842454
C	5.062812	0.098578	1.306283
H	3.169552	-0.038191	2.329274
C	5.737345	-0.065593	0.095634
H	5.572774	-0.625232	-1.984051
H	5.605781	0.435488	2.192516
H	6.808074	0.143838	0.034055
C	-2.412190	-0.721777	0.272319
C	-3.129091	-0.992305	-0.906091
C	-3.156666	-0.515726	1.445948
C	-4.522553	-1.050702	-0.916876
H	-2.578073	-1.161606	-1.836338
C	-4.550438	-0.571126	1.445900
H	-2.627437	-0.308327	2.380830
C	-5.238084	-0.837255	0.261610
H	-5.054333	-1.266798	-1.846979
H	-5.104493	-0.409275	2.374030
H	-6.329798	-0.882319	0.257826
P	-0.703302	1.772104	-0.303372
C	-1.516584	2.845074	0.934927
H	-1.495050	3.908727	0.651967
H	-2.560813	2.517151	1.047875
H	-1.013638	2.716479	1.904458
C	0.986542	2.455029	-0.461785
H	1.529604	2.290935	0.480648
H	1.523895	1.907395	-1.250025
H	0.984295	3.529695	-0.699824
C	-1.529531	2.222506	-1.872387
H	-1.031898	1.700650	-2.702777
H	-2.572776	1.875473	-1.829086
H	-1.511938	3.306722	-2.062307
C	0.269460	-1.465160	-0.394618
C	0.270812	-1.050780	1.244903
H	0.160796	-1.656516	2.151908
C	0.109081	-2.771494	-1.126848
H	0.084672	-2.581467	-2.212789

H	0.808236	-2.823085	0.871528

Int5

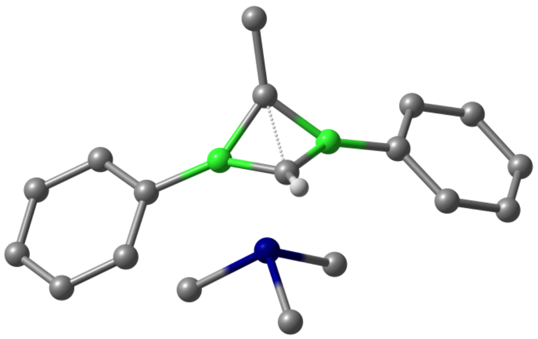

E(scf) = –1089.93926584 a.u.

B	1.066096	-1.489082	0.430867
B	-1.064141	-1.483286	0.430532
C	2.470892	-0.857378	0.237388
C	3.063364	-0.788139	-1.034532
C	3.154963	-0.269385	1.314220
C	4.288661	-0.153985	-1.226510
H	2.542800	-1.227848	-1.889168
C	4.381749	0.366337	1.131249
H	2.710440	-0.308234	2.312601
C	4.948464	0.426575	-0.142147
H	4.731533	-0.105007	-2.223893
H	4.896312	0.820152	1.981354
H	5.907756	0.928063	-0.290524
C	-2.469267	-0.852515	0.237036
C	-3.062320	-0.784697	-1.034664
C	-3.154242	-0.266116	1.314164
C	-4.289213	-0.153439	-1.226110
H	-2.540992	-1.223347	-1.889406
C	-4.382496	0.366858	1.131665
H	-2.709375	-0.304247	2.312421
C	-4.949878	0.425693	-0.141511
H	-4.732680	-0.105648	-2.223283
H	-4.897729	0.819507	1.981979
H	-5.910374	0.924985	-0.289490

H	0.936691	-3.472849	-0.936533
H	-0.836520	-3.274040	-0.867666

TS6

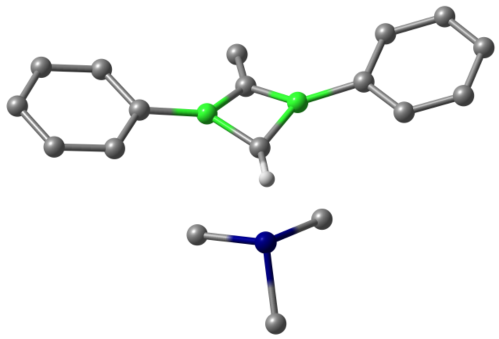

E(scf) = –1089.93034737 a.u.

ν_{min} = –157.0 cm^{-1}

B	1.035926	-0.629678	-0.141066
B	-1.036745	-0.629508	-0.140680
C	2.560932	-0.954297	-0.311332
C	3.244683	-1.773485	0.603135
C	3.303068	-0.399933	-1.367606
C	4.607967	-2.031337	0.470589
H	2.694602	-2.222077	1.435332
C	4.667677	-0.647107	-1.506636
H	2.796152	0.241205	-2.094914
C	5.322563	-1.465665	-0.585863
H	5.117656	-2.674421	1.192177
H	5.224472	-0.203839	-2.335882
H	6.391792	-1.664239	-0.692478
C	-2.561852	-0.953611	-0.311065
C	-3.303338	-0.400307	-1.368346
C	-3.246326	-1.771276	0.604230
C	-4.668001	-0.647064	-1.507573
H	-2.795869	0.239654	-2.096301
C	-4.609684	-2.028655	0.471540
H	-2.696755	-2.219029	1.437217
C	-5.323617	-1.464075	-0.585944
H	-5.224277	-0.204658	-2.337627
H	-5.119946	-2.670524	1.193805

P	0.000935	1.600362	-0.375982
C	0.002217	1.921213	1.443691
H	0.001827	2.997222	1.681810
H	-0.886983	1.454482	1.892443
H	0.892801	1.455474	1.890750
C	1.433189	2.654203	-0.879473
H	2.358051	2.193616	-0.497523
H	1.505016	2.685556	-1.977158
H	1.359020	3.685260	-0.496865
C	-1.432583	2.653305	-0.877771
H	-1.505562	2.684608	-1.975377
H	-2.356742	2.192117	-0.494861
H	-1.358667	3.684397	-0.495218
C	-0.001114	-2.337064	-0.215314
C	-0.000277	-1.770701	1.457233
H	-0.002080	-2.194676	2.464992
C	-0.005535	-3.632860	-0.967436
H	0.078263	-3.408847	-2.043422
H	0.848443	-4.274538	-0.703925
H	-0.934312	-4.204059	-0.822565

H	-6.392898	-1.662293	-0.692697
P	0.000994	2.555201	0.024726
C	1.428248	2.469195	1.179315
H	1.339277	3.194842	2.002586
H	1.492687	1.455414	1.601525
H	2.359794	2.658768	0.626129
C	0.001350	4.344363	-0.432012
H	0.890864	4.567266	-1.039582
H	-0.888207	4.567626	-1.039385
H	0.001582	5.000170	0.453304
C	-1.425766	2.470213	1.180004
H	-2.357291	2.661756	0.627465
H	-1.491533	1.456139	1.601310
H	-1.335292	3.194957	2.003908
C	-0.000254	-0.881668	0.946432
C	-0.000471	0.084553	-1.031354
H	-0.000510	0.584697	-1.999392
C	-0.000035	-1.287880	2.374810
H	-0.000280	-2.393183	2.416668
H	0.898101	-0.956247	2.920179
H	-0.897797	-0.955855	2.920557

P2$_{BCBC}$-C (dimer)

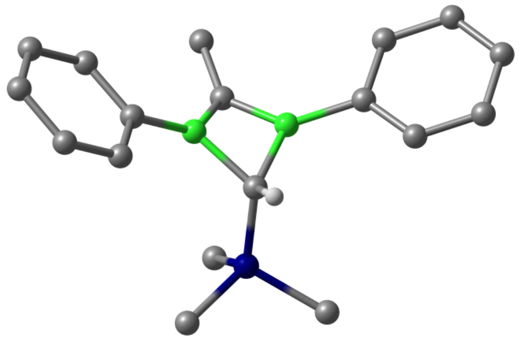

E(scf) = –2179.97893529 a.u.

B	-2.979714	-0.066997	0.857664
B	-1.629100	-1.368189	0.614328
C	-4.463453	0.430155	0.625194
C	-5.557350	-0.441906	0.755593
C	-4.741578	1.759206	0.263185

P2$_{BCBC}$-D (dimer)

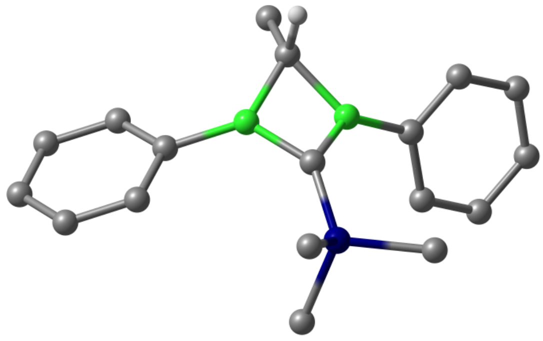

E(scf) = –2179.98339781 a.u.

B	-2.715201	0.601371	0.123240
B	-1.318845	-0.440248	1.013566
C	-4.205931	1.067488	-0.112827
C	-5.284843	0.169925	-0.171191
C	-4.496323	2.432858	-0.269386

C	-6.863893	-0.014810	0.517444	C	-6.591633	0.607006	-0.386346
H	-5.375365	-1.481589	1.043583	H	-5.097040	-0.898959	-0.027685
C	-6.045256	2.200308	0.035476	C	-5.796020	2.880059	-0.503794
H	-3.914528	2.471290	0.171007	H	-3.680303	3.159278	-0.210708
C	-7.111997	1.309328	0.155119	C	-6.848879	1.966726	-0.561805
H	-7.695099	-0.717237	0.618590	H	-7.413390	-0.113065	-0.416354
H	-6.231772	3.242575	-0.235999	H	-5.992089	3.947278	-0.634043
H	-8.134519	1.647389	-0.028819	H	-7.869800	2.314709	-0.736446
C	-1.184880	-2.794577	0.078462	C	-0.791303	-1.534424	2.029931
C	0.067651	-3.050357	-0.509048	C	-0.268160	-2.753848	1.566940
C	-2.060896	-3.887608	0.207780	C	-0.801342	-1.337302	3.421390
C	0.429080	-4.325254	-0.945857	C	0.229075	-3.724548	2.434559
H	0.789750	-2.237299	-0.624471	H	-0.225468	-2.936301	0.490883
C	-1.714377	-5.163378	-0.237335	C	-0.316281	-2.304408	4.303508
H	-3.040873	-3.727874	0.667389	H	-1.205707	-0.406183	3.825407
C	-0.465012	-5.387456	-0.815622	C	0.204596	-3.501407	3.812417
H	1.413636	-4.486527	-1.392560	H	0.640483	-4.655636	2.036496
H	-2.421305	-5.989700	-0.127954	H	-0.343989	-2.122932	5.380800
H	-0.188609	-6.386199	-1.161597	H	0.589684	-4.258129	4.499937
P	-2.003219	0.029579	-1.953930	P	-2.234014	-1.784926	-1.551885
C	-2.421742	1.679817	-2.571396	C	-3.262854	-1.121109	-2.889129
H	-2.510461	1.675842	-3.666921	H	-3.459905	-1.888093	-3.651292
H	-3.370755	2.001683	-2.121335	H	-4.208887	-0.749230	-2.472479
H	-1.623698	2.377181	-2.275371	H	-2.729111	-0.274568	-3.343655
C	-0.531979	-0.487482	-2.867732	C	-0.740904	-2.402095	-2.375784
H	-0.333328	-1.546159	-2.652487	H	-0.067772	-2.883970	-1.654074
H	-0.684685	-0.351502	-3.947867	H	-1.010150	-3.122504	-3.161351
H	0.338624	0.106055	-2.536729	H	-0.197719	-1.553263	-2.812217
C	-3.349026	-1.090946	-2.399801	C	-3.089936	-3.273185	-0.942733
H	-3.096482	-2.098353	-2.037001	H	-2.508225	-3.700346	-0.113652
H	-4.274696	-0.757026	-1.909395	H	-4.078470	-2.985567	-0.559371
H	-3.485451	-1.113626	-3.489741	H	-3.205815	-4.022213	-1.739490
C	-2.343996	-0.968319	1.850914	C	-1.686035	1.113651	1.230959
C	-1.756136	0.033275	-0.212540	C	-1.944272	-0.606539	-0.353293
H	-0.997385	0.791748	0.018894	H	-0.089352	0.576339	-1.661292
C	-2.700551	-1.597315	3.156243	C	-2.162534	1.762858	2.522674

H	-2.477924	-2.678059	3.179902
H	-2.120893	-1.141179	3.978370
H	-3.763494	-1.458864	3.416505
B	2.921789	0.022603	-0.793047
B	1.892284	1.619707	-0.719263
C	4.195571	-0.842432	-0.427878
C	5.493610	-0.310864	-0.510397
C	4.074213	-2.177425	-0.004690
C	6.615328	-1.067127	-0.170006
H	5.622756	0.723154	-0.843699
C	5.190265	-2.946823	0.324837
H	3.079622	-2.631470	0.057789
C	6.466749	-2.389356	0.249590
H	7.612737	-0.625008	-0.236042
H	5.064490	-3.985016	0.642780
H	7.343863	-2.985024	0.513449
C	1.647465	3.120350	-0.274231
C	0.380875	3.577632	0.130301
C	2.690789	4.061784	-0.291025
C	0.161365	4.904646	0.500650
H	-0.460500	2.876895	0.157586
C	2.485930	5.387885	0.090840
H	3.687095	3.741665	-0.610428
C	1.218473	5.814579	0.487390
H	-0.836731	5.229471	0.805606
H	3.318867	6.095375	0.074688
H	1.054337	6.853343	0.783419
P	1.730572	0.283628	1.916235
C	1.675812	-1.386930	2.609650
H	1.681321	-1.348897	3.708136
H	2.542237	-1.958151	2.250695
H	0.749268	-1.872878	2.267050
C	0.380294	1.184751	2.713279
H	-0.576160	0.724808	2.410999
H	0.406054	2.235156	2.393697
H	0.481767	1.125292	3.806459

H	-1.354034	1.915185	3.259415
H	-2.611705	2.753673	2.340582
H	-2.939881	1.157164	3.017566
B	1.813333	-0.335932	-1.046017
B	1.604331	1.573111	-0.724662
C	2.541556	-1.663840	-1.499607
C	3.048223	-1.833925	-2.799470
C	2.638143	-2.761995	-0.626945
C	3.637361	-3.032013	-3.202903
H	2.980472	-1.009544	-3.513220
C	3.206049	-3.971340	-1.026881
H	2.234582	-2.672920	0.384244
C	3.714109	-4.108276	-2.318739
H	4.032135	-3.130821	-4.217288
H	3.256304	-4.809881	-0.327170
H	4.166506	-5.050840	-2.636316
C	2.125786	3.063250	-0.676687
C	1.471826	4.026000	0.110566
C	3.270325	3.472315	-1.380348
C	1.942859	5.336318	0.199960
H	0.565700	3.746950	0.658505
C	3.750979	4.778626	-1.290847
H	3.803378	2.750503	-2.006851
C	3.089125	5.716232	-0.497605
H	1.411388	6.065548	0.816708
H	4.646960	5.068687	-1.845622
H	3.462780	6.740467	-0.427592
P	2.613484	0.514103	1.768805
C	2.955687	-1.082974	2.550155
H	3.417152	-0.930598	3.536207
H	3.637018	-1.660069	1.909971
H	2.014459	-1.637504	2.667812
C	1.615286	1.450592	2.953231
H	0.634839	0.964025	3.035750
H	1.473846	2.466235	2.557966
H	2.101641	1.495739	3.937933

C	3.277152	1.063130	2.435518	C	4.212426	1.378429	1.698973
H	3.303050	2.086037	2.031315	H	4.050611	2.377777	1.269451
H	4.124049	0.495750	2.023728	H	4.888089	0.818789	1.036950
H	3.343211	1.096926	3.531655	H	4.660235	1.468170	2.699426
C	2.625187	1.013895	-1.856563	C	0.973668	0.728887	-1.916656
C	1.622090	0.272673	0.159700	C	1.846262	0.438671	0.245632
H	0.725394	-0.288360	-0.118173	H	-0.891173	1.694067	0.732689
C	3.245106	1.475559	-3.133710	C	1.178814	1.057830	-3.386004
H	4.262265	1.074004	-3.278985	H	0.864552	0.236254	-4.055663
H	3.295727	2.575519	-3.206242	H	2.236617	1.266450	-3.619569
H	2.652685	1.133452	-4.000489	H	0.610972	1.953718	-3.687227

TS2$_{C-H}$

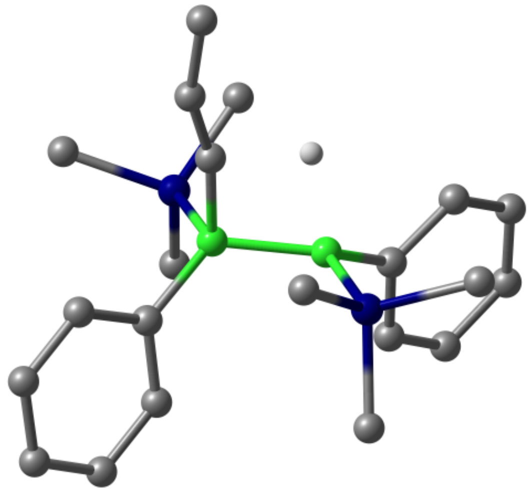

E(scf) = –1550.76020835 a.u.

ν_{min} = –858.1 cm^{-1}

B	0.752028	-0.382844	0.321112
B	-0.806822	0.440129	0.403718
C	1.603150	-0.313459	3.002551
C	1.083574	-0.280110	1.877173
H	-0.388744	0.221250	2.068931
C	1.726220	-0.125809	4.448592
H	1.980067	-1.065994	4.963337
H	2.524634	0.593579	4.691244
H	0.785373	0.255038	4.889993
C	-2.239532	0.026145	-0.159890

syn-**P2$_{C-H}$**

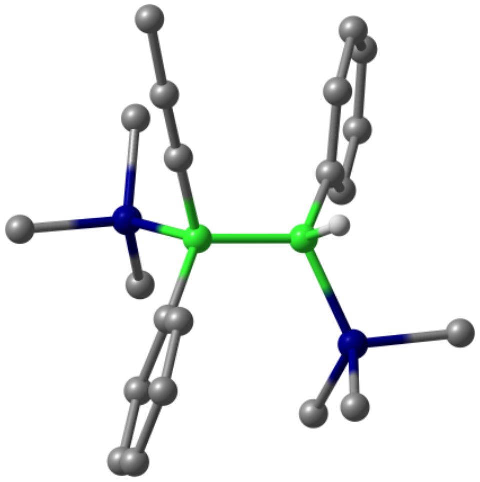

E(scf) = –1550.84204495 a.u.

B	0.434053	0.762477	-0.174545
B	-0.406829	-0.677709	-0.826623
C	-0.328750	2.967865	-1.726038
C	0.014904	2.011588	-1.052210
H	-0.158510	-0.677989	-2.036055
C	-0.740957	4.093142	-2.563182
H	-1.075749	4.948656	-1.955459
H	0.090486	4.443516	-3.194486
H	-1.571464	3.814485	-3.231019
C	-1.997925	-0.726344	-0.571021
C	-2.827783	0.082392	-1.373374

C	-2.476143	-0.075078	-1.544741
C	-3.331335	-0.248355	0.684436
C	-3.721644	-0.435990	-2.060093
H	-1.657278	0.131745	-2.241848
C	-4.579686	-0.614981	0.180211
H	-3.182050	-0.177681	1.765725
C	-4.781411	-0.711888	-1.196555
H	-3.865291	-0.503931	-3.141672
H	-5.402695	-0.826914	0.867850
H	-5.757604	-0.999461	-1.593917
C	1.909937	0.018622	-0.733653
C	3.246663	0.200842	-0.337790
C	1.624426	0.218997	-2.097031
C	4.239824	0.569019	-1.247627
H	3.502652	0.057508	0.716495
C	2.609522	0.577314	-3.017077
H	0.592425	0.102154	-2.445042
C	3.926914	0.757000	-2.593398
H	5.267945	0.708992	-0.902953
H	2.348197	0.724412	-4.068489
H	4.702540	1.044028	-3.307420
P	-0.571089	2.321759	0.451826
P	0.433960	-2.292973	0.133754
C	-1.944445	3.198996	1.269874
H	-2.887720	2.899287	0.790207
H	-1.978211	2.886764	2.322700
H	-1.827220	4.290571	1.204615
C	0.946182	2.891649	1.272934
H	0.955474	2.528438	2.308250
H	1.814328	2.462705	0.752772
H	0.999616	3.989680	1.249278
C	-0.492761	3.089018	-1.207858
H	-0.408645	4.184364	-1.149883
H	0.378730	2.679402	-1.739239
H	-1.402643	2.822344	-1.765327
C	-0.813430	-2.935761	1.287788
C	-2.642656	-1.436156	0.458314
C	-4.196557	0.197768	-1.144450
H	-2.368903	0.653373	-2.184709
C	-4.015634	-1.333863	0.697721
H	-2.061173	-2.094489	1.113599
C	-4.801640	-0.506448	-0.100709
H	-4.801229	0.844636	-1.785775
H	-4.471004	-1.903458	1.512400
H	-5.875218	-0.417135	0.080963
C	2.047026	0.596298	-0.085832
C	2.814337	0.858916	-1.236939
C	2.755109	0.103847	1.024498
C	4.184697	0.614913	-1.287159
H	2.308329	1.260281	-2.119223
C	4.130189	-0.141264	0.991393
H	2.226866	-0.108358	1.958996
C	4.854262	0.105042	-0.172740
H	4.739397	0.825444	-2.205464
H	4.636061	-0.525829	1.881115
H	5.929116	-0.086708	-0.209131
P	0.434012	-2.397454	-0.475975
P	-0.298894	1.345054	1.573319
C	-0.560295	-3.752470	-1.190359
H	-1.546074	-3.782081	-0.706424
H	-0.714570	-3.537347	-2.257197
H	-0.059395	-4.724991	-1.077886
C	2.049824	-2.570315	-1.298316
H	1.949964	-2.242226	-2.341747
H	2.794466	-1.931310	-0.805499
H	2.383532	-3.617862	-1.267952
C	0.773958	-2.955599	1.233814
H	-0.153008	-2.978930	1.823030
H	1.224331	-3.959141	1.236699
H	1.474179	-2.249391	1.699758
C	-1.991025	2.007037	1.467157
H	-2.695806	1.194933	1.243591

H	-1.789689	-2.496368	1.040187	H	-2.266792	2.489107	2.416377
H	-0.870925	-4.032331	1.234511	H	-2.026662	2.735548	0.646550
H	-0.534428	-2.620045	2.302589	C	0.678796	2.721534	2.262648
C	1.951548	-3.237832	0.474778	H	0.240548	3.109246	3.193560
H	1.782419	-4.323082	0.426291	H	1.709199	2.385927	2.443913
H	2.718736	-2.945602	-0.256666	H	0.707506	3.516195	1.503253
H	2.304285	-2.951751	1.476530	C	-0.424672	0.123940	2.927925
C	-0.107658	-2.830587	-1.518599	H	-1.090208	-0.685410	2.599105
H	0.631923	-2.498851	-2.261213	H	0.557404	-0.298984	3.175864
H	-0.209437	-3.924399	-1.565421	H	-0.849753	0.591510	3.828072
H	-1.075355	-2.360586	-1.744403				

anti-**P2**$_{\mathbf{C-H}}$

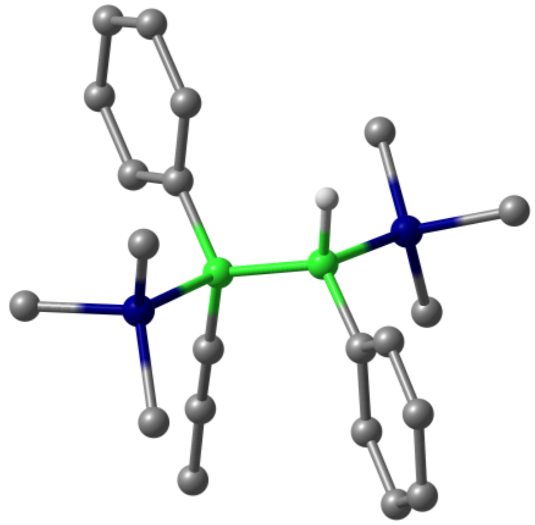

E(scf) = – 1550.85684695 a.u.

B	0.618456	-0.383067	0.179856
B	-0.564614	0.647010	-0.666221
C	0.172550	-0.725939	2.927281
C	0.374319	-0.566733	1.734059
H	-0.242868	0.817579	-1.846917
C	-0.087026	-0.888083	4.357259
H	-0.808427	-1.699370	4.542262
H	0.833530	-1.129990	4.911009
H	-0.506607	0.031390	4.795883
C	-2.130470	0.248282	-0.577633
C	-2.795245	0.010395	0.642086

C	-2.894519	0.069164	-1.745464
C	-4.131070	-0.386285	0.693320
H	-2.233980	0.099786	1.576289
C	-4.229475	-0.338785	-1.708269
H	-2.421054	0.249796	-2.715954
C	-4.856831	-0.568519	-0.484566
H	-4.608004	-0.566635	1.660624
H	-4.784215	-0.474973	-2.640649
H	-5.901688	-0.886245	-0.448484
C	2.178582	-0.062918	-0.178774
C	3.181713	-0.066953	0.806792
C	2.595605	0.238356	-1.489436
C	4.515144	0.220412	0.509813
H	2.901227	-0.293824	1.839500
C	3.926478	0.515930	-1.800854
H	1.847722	0.283928	-2.285860
C	4.896880	0.512011	-0.798876
H	5.262844	0.214638	1.307681
H	4.207193	0.747258	-2.832064
H	5.939669	0.736461	-1.035927
P	-0.385363	2.457529	-0.010593
P	0.314332	-2.203488	-0.496776
C	-1.451395	3.617970	-0.930656
H	-1.157453	3.604328	-1.989752
H	-2.491175	3.267385	-0.858725
H	-1.377057	4.643396	-0.539992
C	-0.838453	2.720182	1.734441
H	-1.900325	2.471087	1.869004
H	-0.240743	2.037725	2.355430
H	-0.664998	3.763281	2.036619
C	1.291444	3.151258	-0.155276
H	1.643806	3.035556	-1.189405
H	1.299527	4.214219	0.126878
H	1.974842	2.585406	0.493088
C	-1.241309	-2.979254	0.041871
H	-2.091216	-2.468100	-0.429996

H	-1.251891	-4.046439	-0.223405
H	-1.328153	-2.861788	1.131019
C	1.618016	-3.351008	0.055637
H	1.444657	-4.375111	-0.305111
H	2.588974	-2.984282	-0.306437
H	1.635787	-3.340567	1.155057
C	0.296420	-2.331852	-2.311435
H	-0.501033	-1.680664	-2.696351
H	1.259976	-1.983313	-2.707441
H	0.119238	-3.368037	-2.633443

$TS_{C \rightarrow Int}$

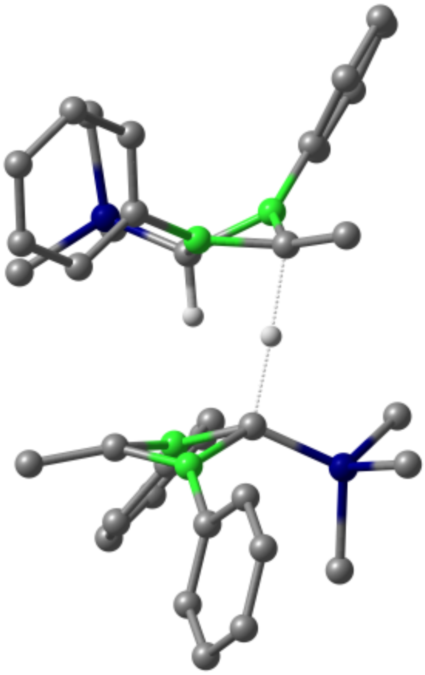

E(scf) = –2179.95736486 a.u.

ν_{min} = –1491.6 cm^{-1}

B	-2.157464	0.203682	-1.092351
B	-1.147872	1.502024	-0.142930
C	-3.558538	-0.251695	-1.648769
C	-4.353543	0.607683	-2.426229
C	-4.061954	-1.538182	-1.390086
C	-5.601244	0.209829	-2.905012
H	-3.984616	1.611967	-2.653861
C	-5.301649	-1.951455	-1.877379

Int

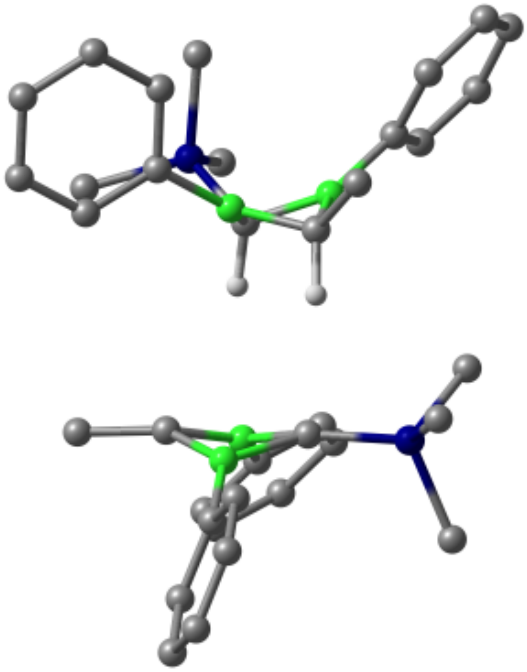

E(scf) = –2179.96887346 a.u.

B	-2.246923	0.850977	-0.491652
B	-1.232547	2.158887	0.467336
C	-3.643213	0.400384	-1.048070
C	-4.438218	1.259762	-1.825530
C	-4.146629	-0.886103	-0.789387
C	-5.685919	0.861908	-2.304313
H	-4.069291	2.264046	-2.053162
C	-5.386324	-1.299376	-1.276680
H	-3.539987	-1.588109	-0.208075

H	-3.455312	-2.240188	-0.808774	C	-6.163710	-0.420187	-2.030656
C	-6.079035	-1.072266	-2.631355	H	-6.288750	1.552853	-2.898780
H	-6.204075	0.900774	-3.499479	H	-5.748888	-2.309378	-1.069318
H	-5.664213	-2.961457	-1.670017	H	-7.139018	-0.735436	-2.408898
H	-7.054343	-1.387515	-3.009597	C	-1.193806	3.529272	1.223482
C	-1.109131	2.877193	0.622783	C	-0.131547	3.847659	2.086792
C	-0.046872	3.195580	1.486093	C	-2.219487	4.476772	1.070925
C	-2.134812	3.824693	0.470226	C	-0.105098	5.056643	2.781524
C	-0.020423	4.404564	2.180825	H	0.688809	3.131955	2.196290
H	0.773484	2.479876	1.595591	C	-2.203028	5.683381	1.772543
C	-2.118353	5.031302	1.171844	H	-3.049535	4.265341	0.388723
H	-2.964860	3.613262	-0.211976	C	-1.145057	5.975232	2.632906
C	-1.060382	5.323153	2.032207	H	0.735805	5.283873	3.441459
H	0.820480	4.631794	2.840760	H	-3.015165	6.402856	1.640676
H	-2.930490	5.750777	1.039977	H	-1.126485	6.921011	3.179654
H	-1.041810	6.268932	2.578955	P	-2.730789	0.717774	2.395925
P	-2.447464	-0.132035	1.915516	C	-3.455048	-0.900066	2.762651
C	-3.015993	-1.823960	2.216529	H	-4.030035	-0.859392	3.698742
H	-3.460681	-1.906406	3.218520	H	-4.116271	-1.196217	1.937107
H	-3.763447	-2.094922	1.458679	H	-2.643745	-1.636828	2.861364
H	-2.155575	-2.505468	2.139062	C	-1.794815	1.202668	3.861603
C	-1.353289	0.291075	3.287791	H	-1.413523	2.223112	3.713651
H	-1.058132	1.345536	3.189947	H	-2.415570	1.153643	4.767038
H	-1.841935	0.118825	4.256867	H	-0.930898	0.526116	3.949621
H	-0.445936	-0.325854	3.198727	C	-4.099130	1.874310	2.162259
C	-3.902490	0.937676	1.971652	H	-3.689722	2.886116	2.029346
H	-3.576971	1.984300	1.884203	H	-4.648929	1.587760	1.253463
H	-4.551575	0.691570	1.118177	H	-4.774656	1.857045	3.028626
H	-4.452508	0.796375	2.912258	C	-1.162039	1.780436	-0.999121
C	-1.096498	1.137924	-1.599820	C	-1.674519	0.695225	1.001198
C	-1.589844	0.043146	0.405282	H	-0.821149	0.013384	1.186590
H	-0.684796	-0.581934	0.444794	C	-1.643377	2.731615	-2.055133
C	-0.827915	1.909335	-2.856577	H	-2.174893	3.597142	-1.621148
H	-1.182938	2.950429	-2.775017	H	-0.791632	3.146603	-2.611318
H	0.255142	1.975953	-3.056367	H	-2.317011	2.257311	-2.781582
H	-1.303682	1.460822	-3.743946	B	2.526577	0.371966	0.193927

B	2.531361	0.371966	0.198711
B	1.462021	-1.054975	0.838979
C	3.909155	0.966704	-0.312670
C	5.141990	0.457540	0.127314
C	3.943737	2.020061	-1.242548
C	6.353210	0.961035	-0.350179
H	5.148983	-0.354187	0.861654
C	5.148827	2.539823	-1.714923
H	3.000199	2.444213	-1.601634
C	6.360511	2.005651	-1.274130
H	7.297759	0.540768	0.005329
H	5.144875	3.365388	-2.431586
H	7.306789	2.405835	-1.646163
C	1.143623	-2.533833	1.328668
C	0.079509	-3.277631	0.787304
C	1.879745	-3.140469	2.362053
C	-0.240220	-4.556064	1.248603
H	-0.524961	-2.833364	-0.010376
C	1.577290	-4.421234	2.824013
H	2.714461	-2.591725	2.807093
C	0.512694	-5.134210	2.270502
H	-1.076024	-5.105705	0.806764
H	2.174065	-4.868148	3.623358
H	0.273250	-6.137012	2.632373
P	1.968575	-1.415169	-2.048450
C	2.476526	-0.372554	-3.443513
H	2.762795	-0.985983	-4.309765
H	3.324392	0.252041	-3.128736
H	1.637106	0.282237	-3.715904
C	0.623143	-2.446072	-2.701357
H	-0.276576	-1.819123	-2.792640
H	0.417945	-3.262839	-1.996693
H	0.885594	-2.867049	-3.682424
C	3.366903	-2.536514	-1.739866
H	3.095365	-3.213561	-0.916628
H	4.229661	-1.929966	-1.426362

B	1.462021	-1.054975	0.834195
C	3.909155	0.966704	-0.312670
C	5.141990	0.457540	0.127314
C	3.943737	2.020061	-1.242548
C	6.353210	0.961035	-0.350179
H	5.148983	-0.354187	0.861654
C	5.148827	2.539823	-1.714923
H	3.000199	2.444213	-1.601634
C	6.360511	2.005651	-1.274130
H	7.297759	0.540768	0.005329
H	5.144875	3.365388	-2.431586
H	7.306789	2.405835	-1.646163
C	1.143623	-2.533833	1.328668
C	0.079509	-3.277631	0.787304
C	1.879745	-3.140469	2.362053
C	-0.240220	-4.556064	1.248603
H	-0.524961	-2.833364	-0.010376
C	1.577290	-4.421234	2.824013
H	2.714461	-2.591725	2.807093
C	0.512694	-5.134210	2.270502
H	-1.076024	-5.105705	0.806764
H	2.174065	-4.868148	3.623358
H	0.273250	-6.137012	2.632373
P	1.216937	-1.011418	-2.276528
C	1.394954	0.335577	-3.484230
H	1.198176	-0.020694	-4.505497
H	2.415770	0.737372	-3.416588
H	0.683573	1.132906	-3.227757
C	-0.469908	-1.639623	-2.532298
H	-1.175478	-0.915713	-2.097536
H	-0.579243	-2.600146	-2.011522
H	-0.687279	-1.776594	-3.601215
C	2.337259	-2.327097	-2.848161
H	2.245643	-3.179802	-2.158385
H	3.369842	-1.940615	-2.786607
H	2.123975	-2.651638	-3.877835

H	3.628573	-3.120683	-2.633813
C	1.974514	0.140445	1.562043
C	1.488362	-0.497498	-0.673464
H	0.212750	0.319185	-1.168942
C	2.329950	0.517567	2.963680
H	3.376245	0.858713	3.055423
H	2.185114	-0.307928	3.684107
H	1.708512	1.355969	3.330855

C	1.974514	0.140445	1.566827
C	1.507496	-0.511849	-0.668680
H	-0.119365	1.474821	-1.196643
C	2.329950	0.517567	2.963680
H	3.376245	0.858713	3.060207
H	2.185114	-0.307928	3.684107
H	1.708512	1.355969	3.335639

TS$_{Int \rightarrow D}$

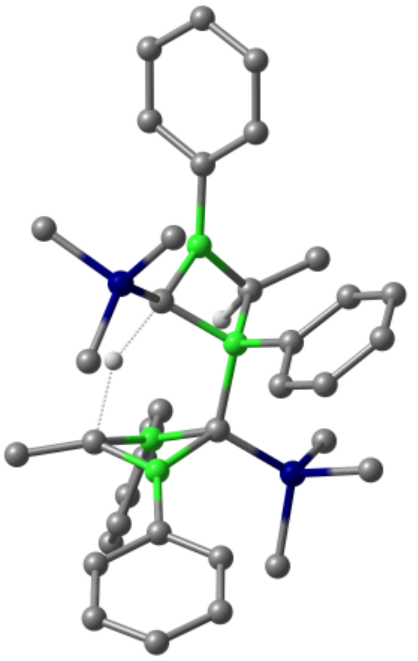

E(scf) = −2179.95973217 a.u.

ν_{min} = −1588.1 cm^{-1}

B	-1.674989	1.316203	0.584757
B	-1.817475	-0.568397	0.782645
C	-2.406543	2.707850	0.397362
C	-3.770400	2.812598	0.722426
C	-1.759072	3.877390	-0.034626
C	-4.465880	4.014123	0.588610
H	-4.299520	1.928126	1.091939
C	-2.442065	5.087821	-0.153592
H	-0.694375	3.844646	-0.282267
C	-3.801784	5.158552	0.148468
H	-5.528532	4.060546	0.839589
H	-1.908907	5.981859	-0.486203

H	-4.339789	6.104137	0.048929
C	-2.693772	-1.886996	0.865525
C	-2.876369	-2.782188	-0.203430
C	-3.312462	-2.229546	2.082480
C	-3.638855	-3.942176	-0.079849
H	-2.392907	-2.577320	-1.160094
C	-4.078516	-3.385946	2.221258
H	-3.189570	-1.571617	2.944697
C	-4.246617	-4.247179	1.137584
H	-3.754588	-4.613689	-0.934083
H	-4.546508	-3.618179	3.181047
H	-4.844731	-5.155477	1.242394
P	-1.906978	0.305461	-2.026664
C	-1.622650	1.882215	-2.877082
H	-2.095811	1.849347	-3.868855
H	-2.054114	2.701594	-2.288671
H	-0.545906	2.051546	-2.988157
C	-1.415998	-0.924560	-3.272123
H	-1.488759	-1.947218	-2.883584
H	-2.065460	-0.810440	-4.152066
H	-0.369803	-0.755187	-3.557605
C	-3.718390	0.192856	-1.920547
H	-4.016853	-0.760344	-1.465411
H	-4.084438	1.017731	-1.293659
H	-4.157365	0.279408	-2.924619
C	-1.601542	0.490878	1.847896
C	-1.124746	0.211665	-0.450009
H	0.527663	2.269480	-0.694308
C	-2.023382	0.758961	3.262930
H	-1.596963	0.038725	3.983733
H	-1.729795	1.767304	3.595122
H	-3.119543	0.697075	3.374570
B	0.627448	0.085129	-0.577301
B	2.235800	1.150001	0.137912
C	1.070977	-1.263589	-1.374820
C	2.051106	-1.293134	-2.380879

C	0.517513	-2.509747	-1.027533
C	2.455670	-2.480788	-2.996707
H	2.514700	-0.360095	-2.704641
C	0.904762	-3.703835	-1.632455
H	-0.252991	-2.546726	-0.255313
C	1.884039	-3.696504	-2.627367
H	3.223622	-2.452975	-3.774454
H	0.440121	-4.644958	-1.325319
H	2.196045	-4.626633	-3.108117
C	3.816044	1.253416	0.233397
C	4.384187	2.115955	1.187101
C	4.699998	0.517226	-0.572643
C	5.766134	2.236747	1.334293
H	3.728137	2.711658	1.830188
C	6.083632	0.629125	-0.428552
H	4.299713	-0.174368	-1.318705
C	6.623265	1.489565	0.526664
H	6.176897	2.918728	2.083268
H	6.745208	0.039207	-1.068030
H	7.706148	1.579391	0.639847
P	1.927613	-1.013772	1.999381
C	0.632776	-2.100112	2.660400
H	1.067926	-2.809410	3.378716
H	0.157967	-2.655662	1.841827
H	-0.137540	-1.490917	3.150062
C	2.710085	-0.265355	3.456838
H	1.979363	0.388870	3.952601
H	3.561176	0.343890	3.120543
H	3.059925	-1.035006	4.159796
C	3.185340	-2.131480	1.310303
H	4.055653	-1.537088	0.999848
H	2.771514	-2.641138	0.428995
H	3.491271	-2.873441	2.062224
C	1.318520	1.587945	-1.035240
C	1.334901	0.197060	0.929162
H	-0.022156	0.655585	1.627594

C	1.912918	2.051353	-2.361666
H	1.405592	1.631796	-3.249914
H	2.976123	1.777754	-2.462600
H	1.871771	3.148490	-2.463138

References

1 G. Doisneau, G. Balavoine and T. Fillebeen-Khan, *J. Organomet. Chem.*, 1992, **425**, 113.

2 A. Hermann, T. Zorn, M. Arrowsmith, F. Fantuzzi, B. Ritschel, B. Engels, I. Krummenacher, K. Radacki and H. Braunschweig, *Angew. Chem. Int. Ed.*, 2020, **59**, 15717.

3 H. Braunschweig, I. Krummenacher, C. Lichtenberg, J. D. Mattock, M. Schäfer, U. Schmidt, C. Schneider, T. Steffenhagen, S. Ullrich and A. Vargas, *Angew. Chem. Int. Ed.*, 2017, **56**, 889

4 H. Braunschweig, R. D. Dewhurst, C. Hörl, A. K. Phukan, F. Pinzner and S. Ullrich, *Angew. Chem. Int. Ed.*, 2014, **53**, 3241.

5 P. Bissinger, H. Braunschweig, M. A. Celik, C. Claes, R. D. Dewhurst, S. Endres, H. Kelch, T. Kramer, I. Krummenacher and C. Schneider, *Chem. Commun.*, 2015, **51**, 15917.

6 T. E. Stennett, J. D. Mattock, I. Vollert, A. Vargas and H. Braunschweig, *Angew. Chem. Int. Ed.*, 2018, **57**, 4098.

7 G. Sheldrick, *Acta Cryst.*, 2015, **A71**, 3.

8 G. Sheldrick, *Acta Cryst.*, 2008, **A64**, 112.

9 A. L Spek, *Acta Cryst.*, 2015, **C71**, 9.

10 M. J. Frisch, G. W. Trucks, H. B. Schlegel, G. E. Scuseria, M. A. Robb, J. R. Cheeseman, G. Scalmani, V. Barone, G. A. Petersson, H. Nakatsuji, X. Li, M. Caricato, A. V. Marenich, J. Bloino, B. G. Janesko, R. Gomperts, B. Mennucci, H. P. Hratchian, J. V. Ortiz, A. F. Izmaylov, J. L. Sonnenberg, D. Williams-Young, F. Ding, F. Lipparini, F. Egidi, J. Goings, B. Peng, A. Petrone, T. Henderson, D. Ranasinghe, V. G. Zakrzewski, J. Gao, N. Rega, G. Zheng, W. Liang, M. Hada, M. Ehara, K. Toyota, R. Fukuda, J. Hasegawa, M. Ishida, T. Nakajima, Y. Honda, O. Kitao, H. Nakai, T. Vreven, K. Throssell, J. A. Montgomery, Jr., J. E. Peralta, F. Ogliaro, M. J. Bearpark, J. J. Heyd, E. N. Brothers, K. N. Kudin, V. N. Staroverov, T. A. Keith, R. Kobayashi, J. Normand, K. Raghavachari, A. P. Rendell, J. C. Burant, S. S. Iyengar, J. Tomasi, M. Cossi, J. M. Millam, M. Klene, C. Adamo, R. Cammi, J. W. Ochterski, R. L. Martin, K. Morokuma, O. Farkas, J. B. Foresman and D. J. Fox, Gaussian 16, Revision B.01, Gaussian, Inc., Wallingford CT, **2016**.

11 J.-D. Chai and M. Head-Gordon, *Phys. Chem. Chem. Phys.*, 2008, **10**, 6615.

12 (a) F. Weigend and R. Ahlrichs, *Phys. Chem. Chem. Phys.*, 2005, **7**, 3297; (b) F. Weigend, *Phys. Chem. Chem. Phys.*, 2006, **8**, 1057.

13 (a) A. V. Marenich, C. J. Cramer and D. G. Truhlar, *J. Phys. Chem. B*, 2009, **113**, 6378; (b) V. Barone and M. Cossi, *J. Phys. Chem. A*, 1998, **102**, 1995; (c) M. Cossi, V. Barone, B. Mennucci and J. Tomasi, *Chem. Phys. Lett.*, 1998, **286**, 253; (d) V. Barone, M. Cossi and J. Tomasi, *J. Comp. Chem.*, 1998, **19**, 404; (e) J. Tomasi, B. Mennucci and R. Cammi, *Chem. Rev.*, 2005, **105**, 2999.

14 C. Y. Legault, CYLview20, Université de Sherbrooke, 2020 (http://www.cylview.org).

15 T. Noro, M. Sekiya and T. Koga, *Theor. Chem. Acc.*, 2012, **131**, 1124.
